# Follow the eyes: gaze and grammaticality

**DOI:** 10.3389/fpsyg.2024.1415590

**Published:** 2024-11-15

**Authors:** Laszlo Hunyadi

**Affiliations:** Hungarian Research Centre for Linguistics, Budapest, Hungary

**Keywords:** grammaticality, eye tracking, fixation, saccades, presentation order effects

## Abstract

This paper studies the role of eye tracking in detecting grammatical violation in reading tasks. It tests the assumption that encountering syntactic violation has its correspondence in the behavioral patterns associated with gazing. Applying the *T-pattern analysis* offered by the research environment *Theme*, it is shown that the observation of grammaticality/agrammaticality in samples of experiments is reflected in the partial correlation of these categories across a number of structural patterns. It is however also shown that their expected difference in the total duration of focusing is not confirmed. It is suggested that associating eye tracking with an additional sound recording or with linguistic ERP studies could widen the categorical spectrum of identifying grammatical violations.

## 1 Introduction

In our text based digital world one of the most widespread tools we use is the spell-checker. We rely on it in case we are unsure about the proper spelling of a given word, however, it also corrects us even in cases we do know the spelling, but the word is still, inadvertently, misspelled. In a way it is something similar to a slip of the tongue, when what we say is different from what is in our thought. It is a kind of difference captured in theoretical linguistics in terms of the distinction between competence and performance (*cf.*, among others, Hauser et al., 2002). Our commonly shared human linguistic competence is realized in our actual, concrete linguistic performance, the latter featuring non-arbitrary, but still individual characteristics. When we are reading, often we rush through some words without noticing a spelling error just because the abstract mechanism of our competence, that we might call predictive understanding, takes the lead over the analysis of some surface observation. We can say that seeing and noticing are two different actions which are not always in synchronicity or even equally manifested. Still, probably the most obvious way to reach closer to one’s cognitive mechanism and learn about its certain functions is through performance, i.e., the observation of some relevant behavior.

This is what we see in a number of important neurophysiological studies aiming to identify certain linguistic processes in the brain such as syntactic or semantic processing (*cf.* Angela, 2002, 2004; Gunter and Friederici, 1999; Jolsvai et al., 2011; Kocsis et al., 2017). As an electrophysiological response of the brain to the linguistic stimulus, especially the one representing a mismatch with respect to the grammar of a given language, the resulted event-related potential (ERP) is strongly associated with the timing of the presentation of the stimulus, and, as a result, the ERP signal can be considered as an indication of the type of the linguistic mismatch in question that, in turn, can lead to a better understanding of the neural basis of the given linguistic property (*cf.* Angela, 2002).

In order to ensure the exact onset time detection of the ERP signal with respect to the presentation of the given stimulus, such experiments rely on the presentation of the linguistic material (usually a sentence) as a sequence of individual words on the computer screen, with the caveat that this mode of presentation does not fully replicate the process of natural, continuous reading. In a listening rather than reading experiment, this issue of properly detecting evoked potential as a response to grammatical mismatches can, however, be avoided. In Szalárdy et al. (2018), the subjects were presented with two continuous and concurrent speech streams, while the manipulation of the variables of attention and task affecting syntactic grammaticality was aimed at eliciting ERPs. As a result, the study argued that syntactic analysis is not automatic, syntactic violation only elicits an ERP response in the task condition.

As for reading tasks, an experimental setting conforming to natural, continuous reading can be supported by means of tracking eye movement while reading. Already in measuring reading comprehension, it was shown that registering eye movement and generating patterns of reading yields a more reliable insight into the formation of the process of linguistic and contextual comprehension than the established, widely used tests without relying on eye tracking (*cf.* Mézière et al., 2023). As for specific error detection tasks, there have been a number of psycholinguistic, communication and computational studies using the eye tracker, capturing data related to fixation and saccades and associating them with errors or other features of the text as stimulus, ultimately offering an insight into various aspects of language processing. A recent research topic *Eye-tracking while reading for psycholinguistic and computational models of language comprehension* in *Frontiers in Psychology* (*cf.* Palmović et al., 2023) represents a number of experimental approaches across a number of languages and different language types for the study of language processing by means of capturing how eye movement can reflect various linguistic errors. Even though capturing the same physical parameters (fixation and saccades), due to the often incompatible typological differences of the languages involved, no single model proved to be applicable to all of them (*cf.* Falcon et al., 2023).

There is an obvious similarity between studying the path the eyes take during reading or while observing a web page. Even though the path is essentially linear in reading a text whereas, at the same time, it is more determined by non-linearly organized, non-textual visual objects on a web page, the two different ways of forming a path of the gaze can appear in both modalities: while reading, the reader (especially the fast reader) can skip certain words or can return to an already passed detail and thus break linearity, whereas the observer of a web page can also turn to reading linearly a piece of text on the page. Techniques of scanpath analysis with eye tracking are compared and evaluated in Sukru et al. (2016), presenting a choice of approaches to capture the temporal and special progress of the eyes while reading/observing.

The aim of the present study is to examine the possible benefits of applying the approach of *T-pattern analysis* (*TPA*), (*cf.* Magnusson, 1996; Magnus et al., 2016) to error detection in reading tasks. TPA differs from previous models of behavioral studies by detecting temporal patterns with the following specific assumptions: (a) while the order of pattern components is important, these components (called events) need not necessarily be adjacent (i.e., there may occur other events in between without them belonging to the given pattern), (b) the time intervals between the events of the pattern should fall within a so-called critical interval (e.g., for events A and B), B following (either or not adjacently) A, they will form a single pattern AB if there is a (predefined) critical interval between A and B, (c) any pattern candidate should occur in the observation period at least a certain (predefined) number of times. These key features of the TPA model may suggest that, for the analysis of reading tasks with focus on the path of the gaze, scanpath analysis can be a more suitable approach. However, if one is interested in capturing the overall characteristics of the process of reading, including the eventual discovery of further, short-timed patterns and larger patterns consisting of these patterns, or the kinds and distribution of them across the whole observation period as well as capture the pattern-wise difference within pairs of grammatical/agrammatical sentences, the holistic approach of TAP may reveal further important information about this kind of behavior. In what follows, we will apply the T-pattern analysis to test the extent to which the global, holistic difference between texts with and without grammatical errors can be identified by observing behavioral patterns associated with eye tracking.

## 2 Material and methods

The stimuli for the experiment consisted of 60 simple Hungarian sentences. They were based on the set of stimuli from a previous experiment on the processing of syntactic violations during listening to two concurrent speech streams (*cf.* Szalárdy et al., 2018). 30 contextually independent grammatical sentences were selected from newspaper news, covering a variety of everyday topics including internet use, sport, career guidance and recent history. Further 30 agrammatical sentences represented the agrammatical counterparts of the above grammatical ones with one of two types of errors each: 20 of them violating subject-object agreement and 10—violating verb-object agreement. In order to maintain the attention of the subjects during the experiment, each of the sentences contained a numeral phrase.

The subject selected for this study was a 26 year old healthy male university graduate.

Each of the sentences was visually presented only once at the center of a computer screen in a randomized order. The subject was asked to read the sentence aloud and judge its grammaticality by saying “correct” or “incorrect.” Reading time was not restricted, and the subject proceeded to the next sentence by pressing ENTER. The voice of the subject was also recorded with the purpose to identify further possible cues of agrammaticality. With Hungarian being an agglutinative language expressing agreement relations by word-final morphology, proper judgment required reading carefully up to the end of the respective words. Each word of each sentence was marked with a corresponding ROI to properly follow and identify the target of eye movement.

For data acquisition we used a ViewPoint 90 fps USB eye tracker from Arrington Research in binocular mode. Two computers were used, an Apple PowerMac for running the presentation software PsyScope v. B77 and a Windows computer for running the data acquisition software (ViewPoint v. 2.9.2), synchronized via the ethernet port. Eye data from the eye tracker hardware to the ViewPoint software traveled via the serial port. For stable viewing position and the restriction of head movement ViewPoint’s HeadLock^™^ Ultra Precision Head Positioner was used. Eye tracking was carried out with two desk mounted cameras and an illuminator for binocular eye tracking.

The overall settings of the experiment, including the positioning of the eyes (45 cm horizontal x 45 cm vertical from a 24 inch monitor) and the pupil based calibration followed the manufacturer’s recommendations. The subject performed the task sitting alone in a sound proof room, with all other equipments being in, and the experiment leader observing from an adjacent room. The eye tracker recorded the resulting fixations and saccades of both eyes. The processing of the raw data was managed using ViewPoint’s built-in Data Analyzer. Final data analysis was performed on data from a single eye.

## 3 Patterns of behavior as indication of variations in grammaticality

Reading takes place across time, accompanied by various markers of the cognitive processes enabling it. One of its noticeable markers is eye movement interpreted in terms of gaze direction, saccades and fixation. Gazing has its own patterns depending on the relative position and content of its object, spread across time. This temporality can be captured both by the simultaneity or consecutiveness of its components, their adjacency or non-adjacency. The patterns are not always obvious to the direct observer; they may often be captured only as a result of a longer observation period. A set of events becomes characteristic of a certain function only if they have multiple occurrences with the same interpretation, such as attention, control or correction. These events include, among others, the starting or stopping of an eye movement, the change in the speed of the movement, the change of the size of the pupil, even blinking. These patterns can be more generic, relating to some human- or culture-specific action, or on the contrary, more specific to an individual. These are all patterns of behavior, constituting the focus of *T-pattern analysis* as offered by the research environment *Theme* (*cf.* Magnus et al., 2016). In this section we are going to find out whether the judgement of grammaticality/agrammaticality can be supported by various patterns of eye movement.

In terms of *Theme* an instance of behavior is represented by an event occurring at a given point of time and specified by the behaviors (called items) taking place at that point of time. As such, an event can consist of a single item or multiple ones. As an example: if a saccade starts at a point of time and it is characterized by its direction (such as forward, backward or to a more remote target), then the two items (the start and the direction of the saccade) make a single event. There is a token-type relation between an event and an event type (ET): any event at a single time is the token, its multiple occurrences across the whole observation period constitute to the given event type.

The fundamental criteria for any two events of the same or different type to form a pattern (called *T-pattern*) in *Theme* is that they should occur within a so-called *critical interval*, at multiple (usually at least 3) times, and at a certain significance level (usually *p* = 0.005 or better). In the case of eye tracking *saccades* and *fixations* serve as the primary event types, saccades associated with direction and fixations with duration. Since *Theme* considers the beginning and the end of an action as two separate events, the set of event types also includes the behaviors *begin* and *end*. In our study we identified the following ET’s: *b* for *begin*, *e* for *end*, f *for* fixation, *s* for *saccade,* as well as the directions of the latter: *dwn* for *down*, *up* for *up*, and *bck* for *backwards.*

The most frequent patterns found in our data include those consisting of two ETs only, such as (e,s b,f) where the end of a saccade forms a pattern with the start of a fixation within the critical interval (each of the samples of the same pattern can have a variable interval within the predefined critical interval, in our case 1,500 ms). Other frequent patterns are (e,f e,s), where the end of the fixation forms a pattern with the end of the saccade, or (b,s,dwn e,s), where the beginning of a downwards saccade forms a pattern with the end of – obviously the same—saccade. The longest patterns found in our recordings include (b,s,up (e,s (b,f b,s,up)) (e,f (e,s b,s,up))): an upwards moving saccade > (its) ending > the beginning of a fixation > the beginning of yet another upwards moving saccade > end of fixation > end of saccade > beginning of yet another upwards moving saccade, or the pattern ((b,s,dwn (e,f e,s)) (b,s,dwn (e,s b,f))): a downwards moving saccade > end of fixation > end of a saccade > beginning of yet another downwards moving saccade > end of a saccade > beginning of a fixation.

Obviously, saccades and fixations are strongly related: considering our total data, out of 243 cases of the ET b,f (the beginning of a fixation) it is followed by b,s (the beginning of a saccade) 91 times, by the end of (the same) fixation 50 times, by the end of a saccade (e,s) 15 times, and there is a high number of occurrences (87) where b,f is the final element of the pattern. The end of a saccade (e,s), occurring altogether 312 times forms a pattern with the following b,f (beginning of fixation) most frequently, 143 times, by the end of a fixation (e,f) 53 times, by the beginning of yet another saccade (b,s) 42 times, and it is the final ET of any pattern in 72 times.

In what follows, instead of characterizing descriptively the gazing behavior of the reading of each of the grammatical/agrammatical pairs of sentences, we will consider parameters which are global to the set of all such pairs, assumed to be characteristic of the way one encounters grammatical issues with a text.

**Figure 1:**
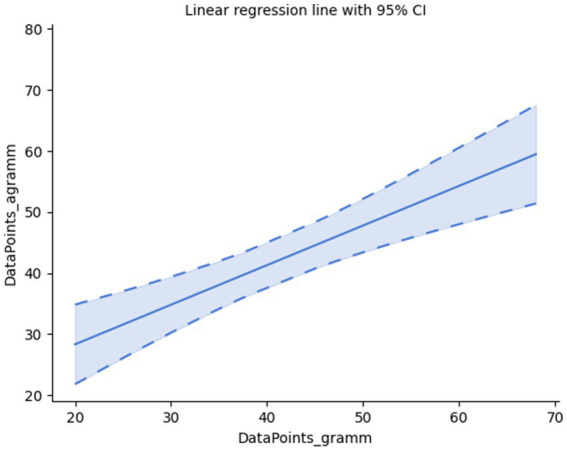
Correlation of the number of data points in grammatical and agrammatical samples.

We might assume that noticing a grammatical violation will change the behavioral pattern associated with gazing as compared to the case when no grammatical violation is encountered. Let us examine this question based on a variety of parameters. First, find out if a difference in the number of registered datapoints within each of the samples can reflect such a distinction (see Figure 1).

It is found that, according to the number of data points (events), the gazing of grammatical and agrammatical samples are somewhat positively correlated but are still not identical: Pearson’s correlation: r(28) = 0.69, *p* < 0.001, Spearman’s rank-order correlation: r_s_(28) = 0.70, *p* < 0.001. Their positive correlation can be attributed to the fact that in each pair of samples (grammatical vs. agrammatical; please note that the members of each pair were randomly, i.e., not adjacently presented among the samples) the gaze follows the same linguistic material, whereas the observation of the violation constitutes to their difference as well.

**Figure 2:**
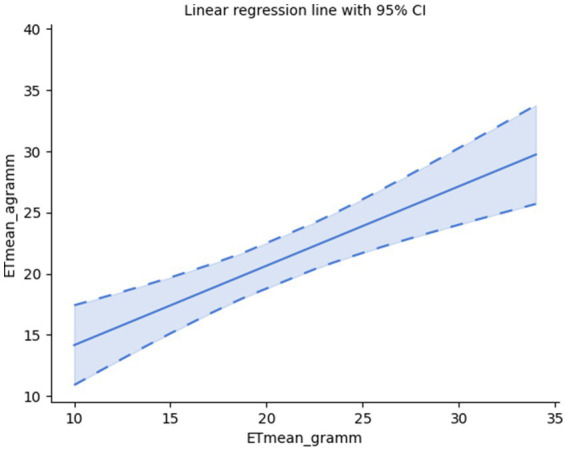
Correlation of the means of ETs in grammatical and agrammatical samples.

The two types of sentences have a similar relation regarding the mean value of the number of event types (ETs); *cf.* Figure 2.

As found, they are similarly correlated: Pearson’s correlation: r(28) = 0.69, *p* < 0.001, Spearman’s rank-order correlation: rs(28) = 0.70, *p* < 0.001.

**Figure 3:**
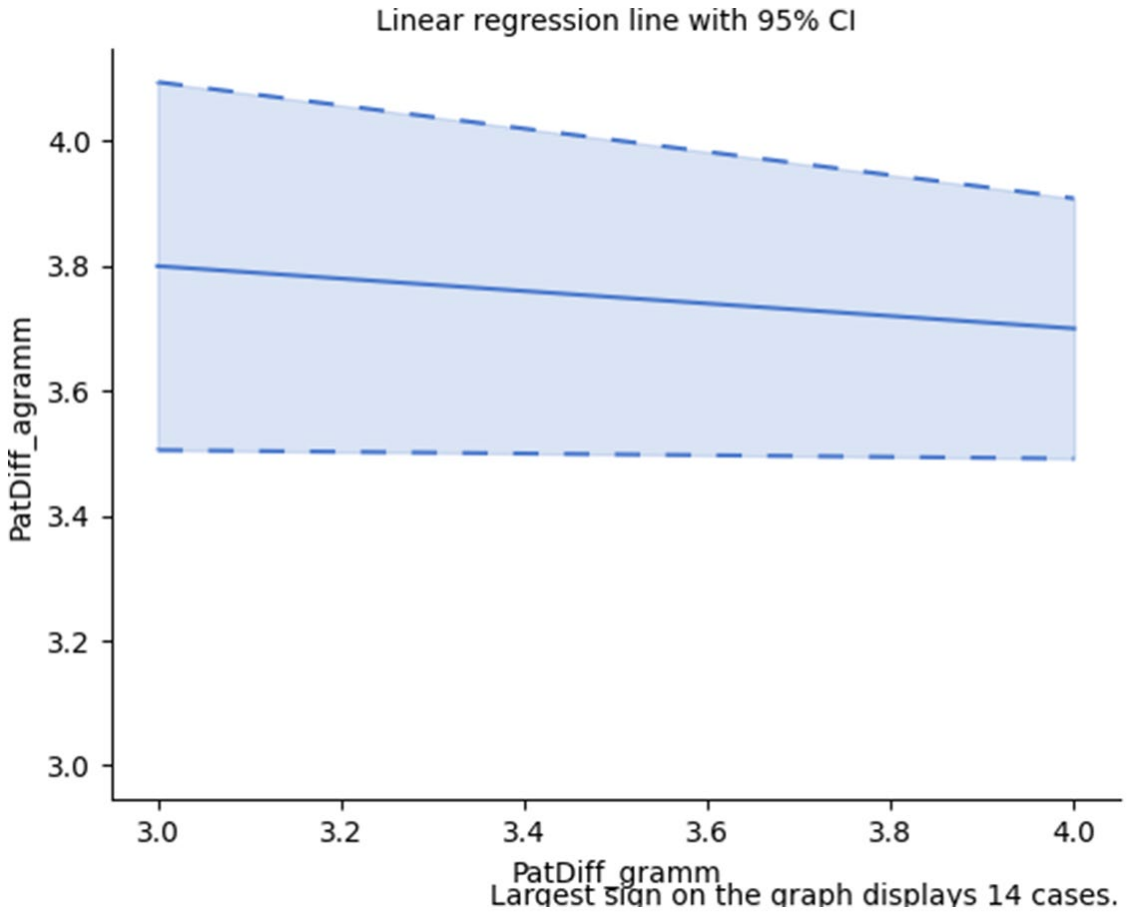
Number of different patterns.

However, if we ask whether grammaticality/agrammaticality also determines significantly different numbers of patterns, Spearman’s rank-order correlation (*r_s_*(28) = −0.11, *p* = 0.575) shows a weak negative correlation, but, actually, this relation is not statistically significant. Accordingly, the actual difference between the number of patterns in grammatical and agrammatical sentences may not be due to their being grammatical or agrammatical, respectively; *cf.* Figure 3.

**Figure 4:**
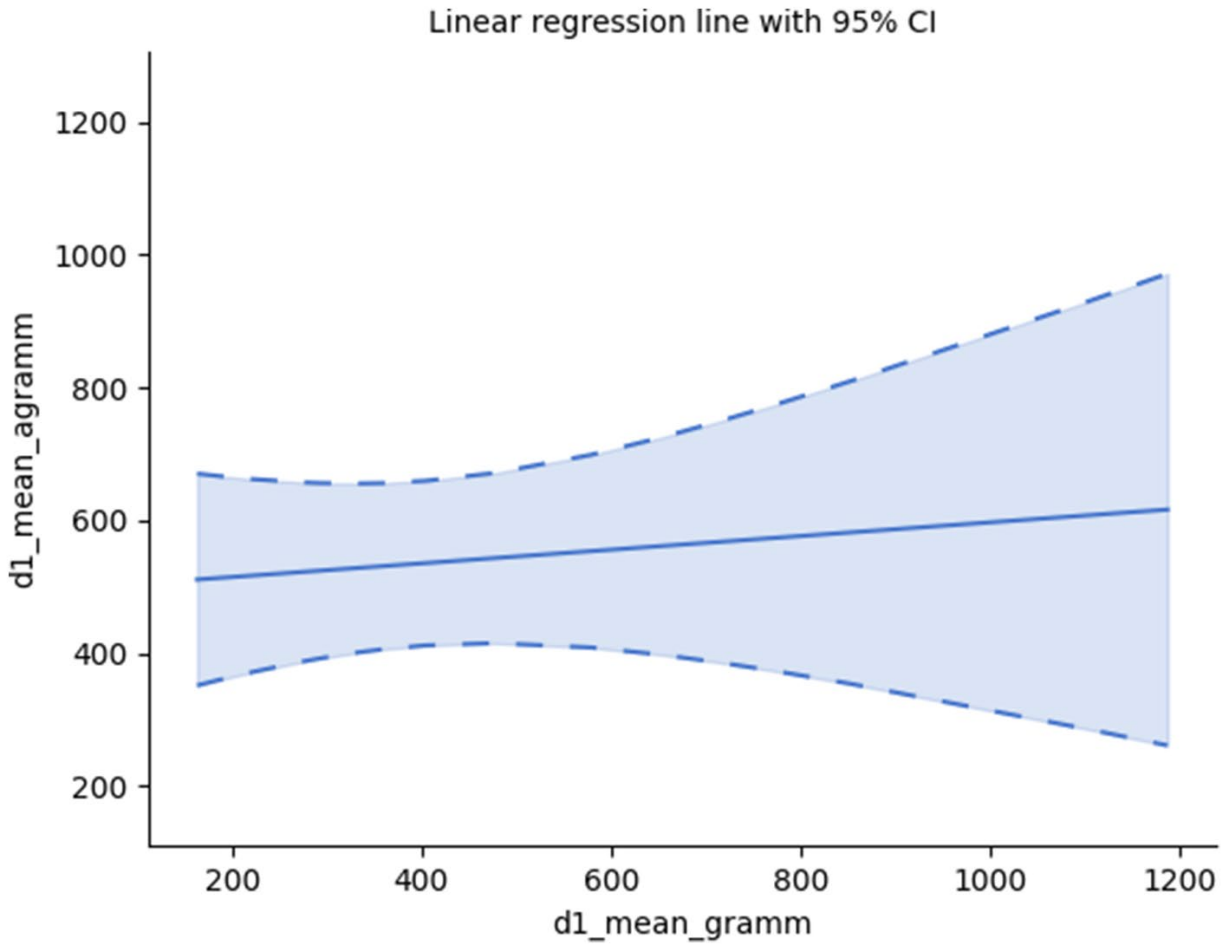
Minimal critical interval.

**Figure 5:**
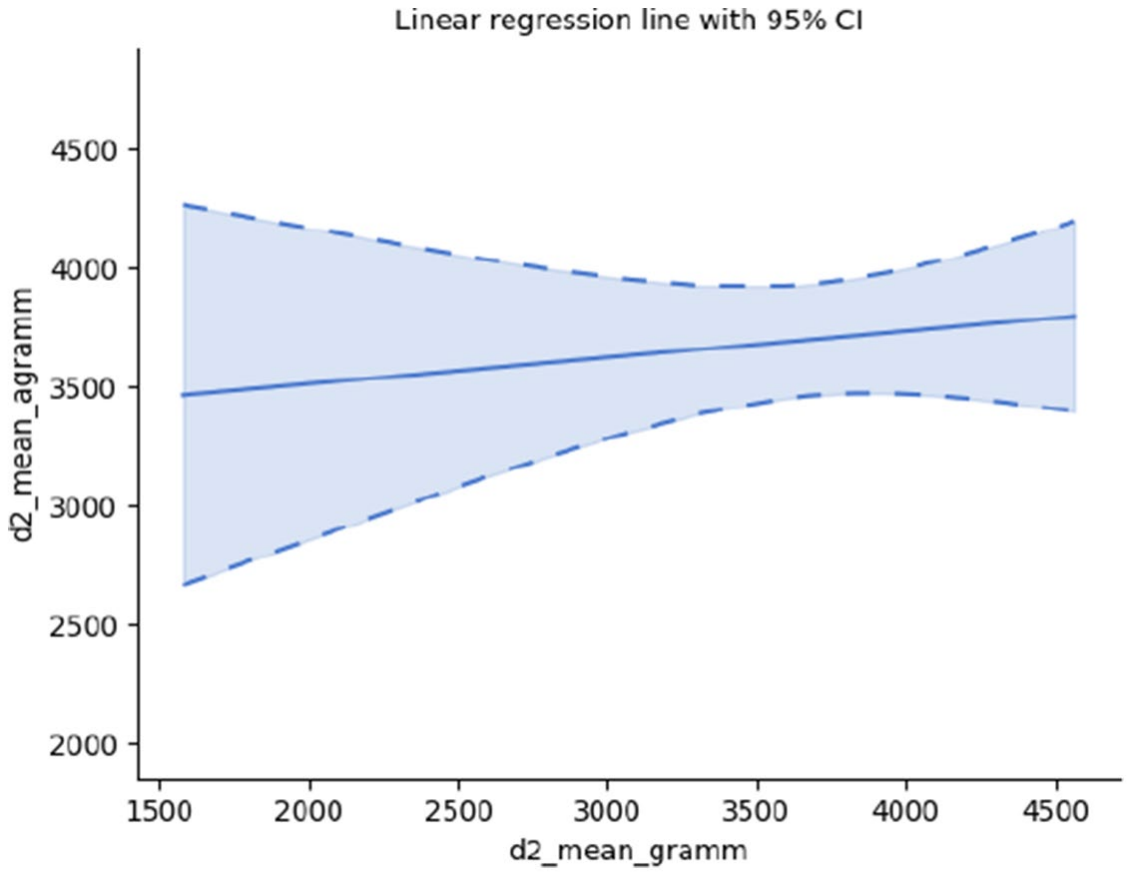
Maximal critical interval.

Further, considering the so-called critical interval, i.e., the minimal and maximal temporal distance within which events form a single pattern, these two parameters for pairs of grammatical and agrammatical samples appear to lack correlation, too: d1 mean: Spearman’s rank-order correlation: r_s_(28) = −0.06, *p* = 0.752, d2 mean: Spearman’s rank-order correlation: r_s_(28) = 0.20, *p* = 0.278; suggesting that the observed differences in the processing time of grammatical and agrammatical sentences may be due to other, non-grammatical factors; *cf.* Figures 4, 5.

**Figure 6:**
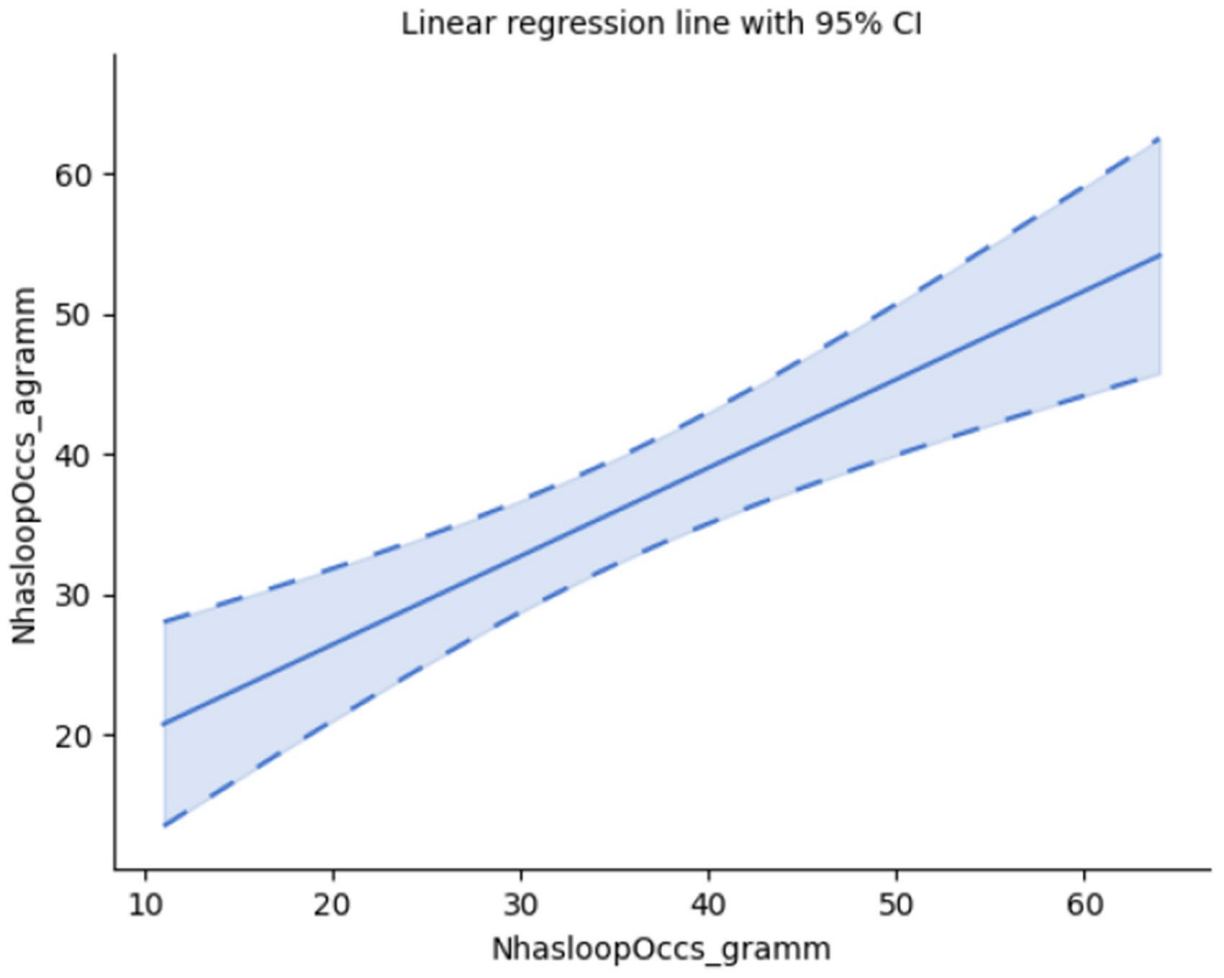
Number of occurrences of loops.

Finally, pairs of samples do not significantly differ with respect to the number of loops within a sample (as such, if an event of focusing is followed by yet another event of focusing, as an expected pattern of repeated attention taking, it creates a loop within the pattern). Accordingly, this recursive formation of loops may be a property of gazing in general, regardless of the grammaticality status of the reading stimulus; *cf.* Pearson’s correlation: r(28) = 0.69, *p* < 0.001, Spearman’s rank-order correlation: r_s_(28) = 0.66, *p* < 0.001; *cf.* Figure 6.

## 4 Fixation as the reflection of grammaticality/agrammaticality

In addition to the global correlation between the eye tracking of grammatical/agrammatical pairs of samples, let us find out whether gaze movement, in particular saccades and fixation reflect this categorical difference in terms of direction and duration.

**Figure 7:**
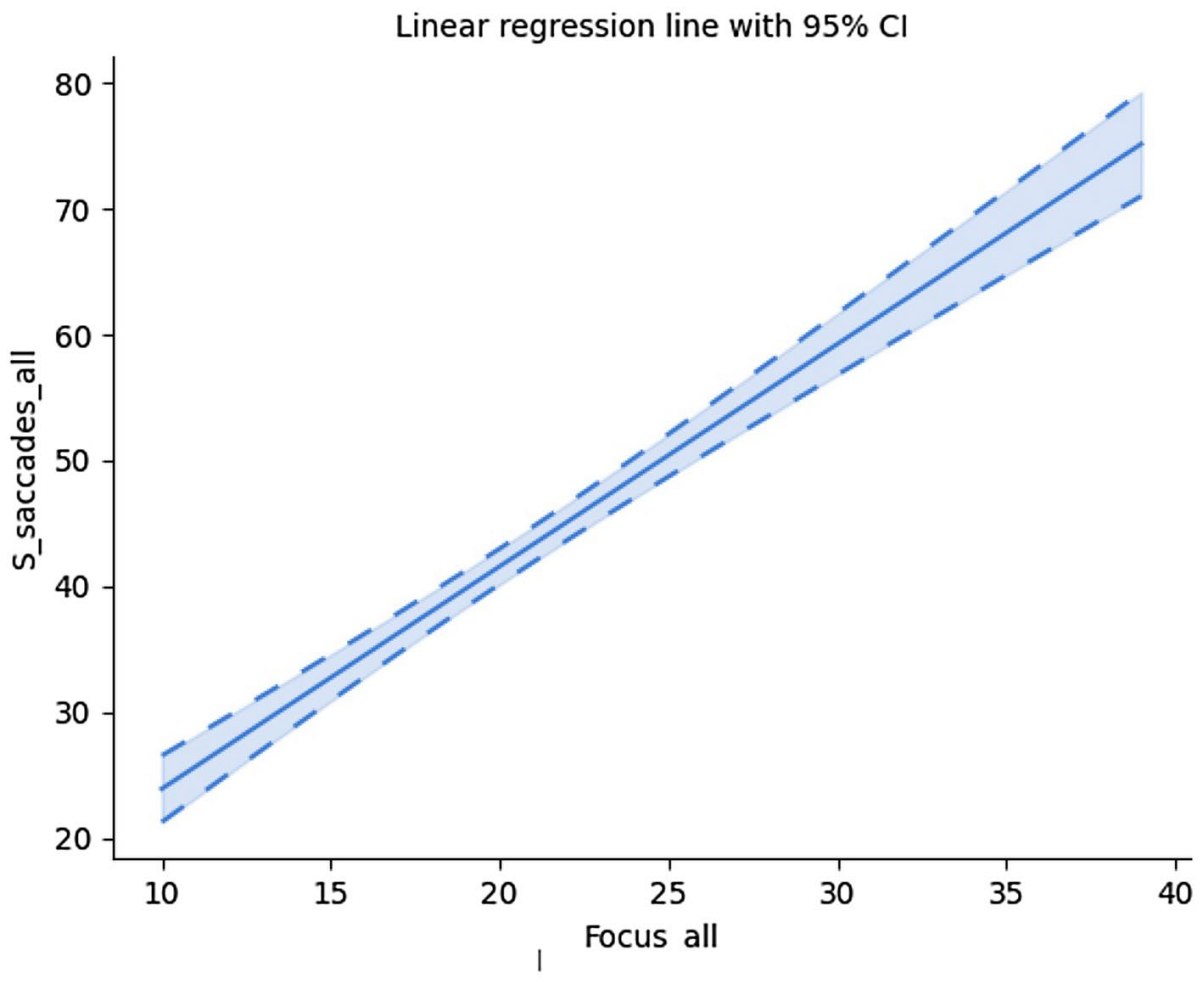
Saccades vs. fixations.

Since saccades and fixations are mutually dependent (the gaze moves to a position in order to fixate there), it is no surprise that the two are correlated (Spearman’s rank-order correlation: *r_s_*(58) = 0.89, *p* < 0.001); *cf.* Figure 7.

**Figure 8:**
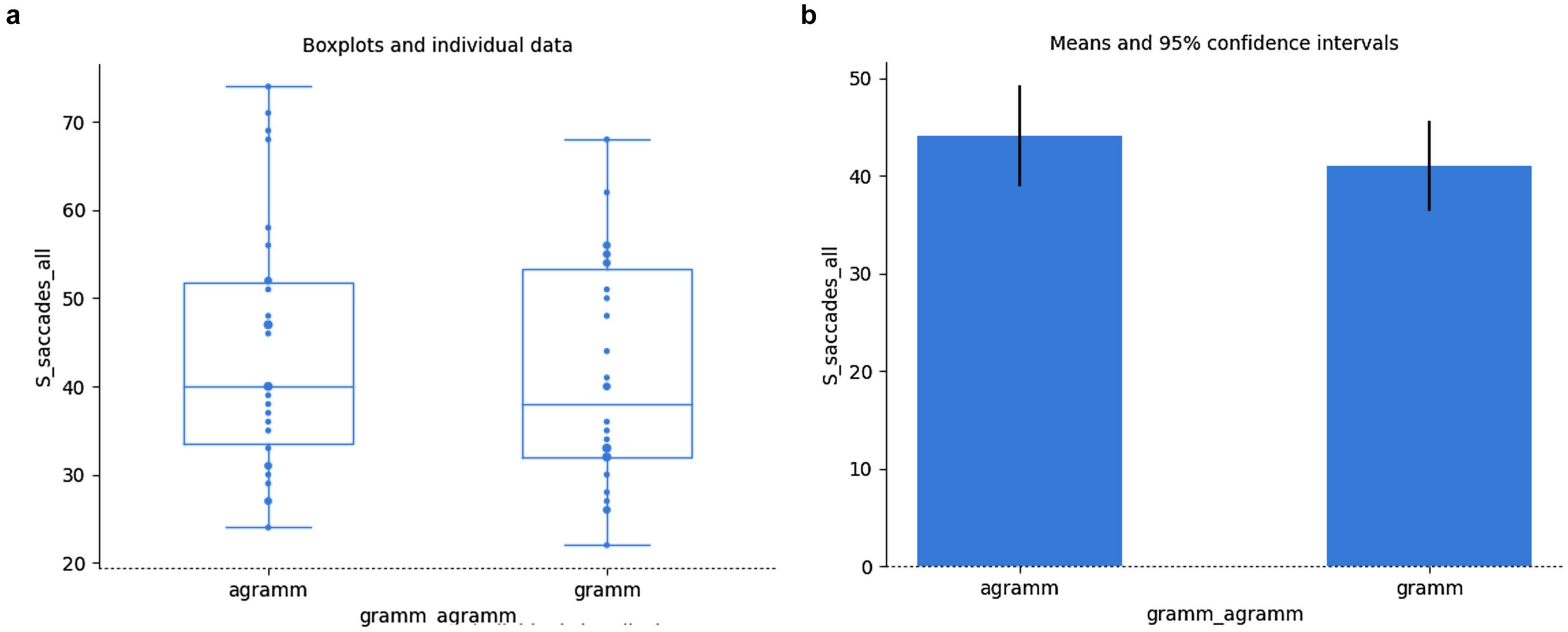
Saccades vs grammaticality: individual data **(a)**, means **(b)**.

Even though there are more saccades in cases of agramamticality, this fact does not amount to showing a significant difference (independent samples t-test: *t*(58) = 0.89, *p* = 0.380, Bayesian independent two-samples *t*-test: BF_10_ = 0.36, BF_01_ = 2.74); *cf.* Figures 8A,B.

**Figure 9:**
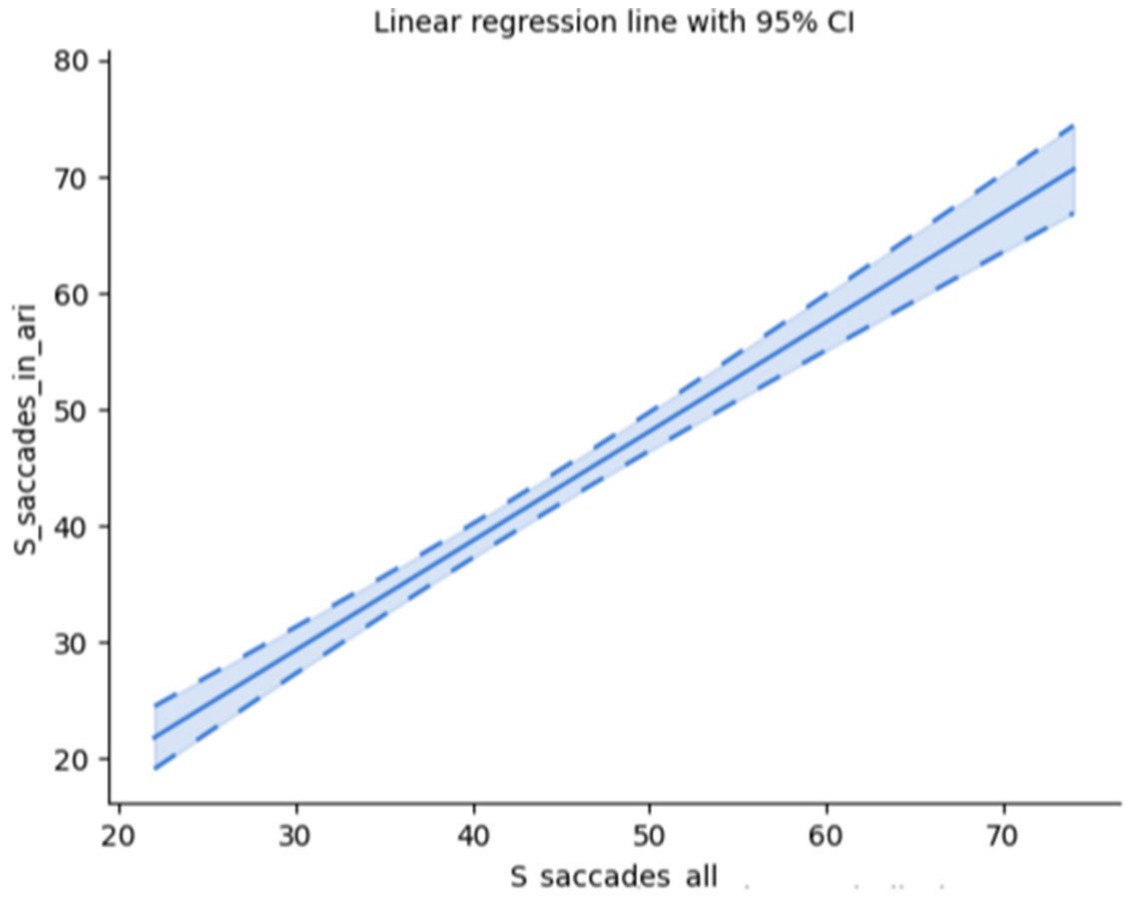
All saccades vs. Saccades in regions of interest.

As also expected, the target of saccades clearly correlates with the position of words in a sentence: Spearman’s rank-order correlation: *r_s_*(58) = 0.89, *p* < 0.001 (ari: region of interest, for eye A); *cf.* Figure 9.

**Figure 10:**
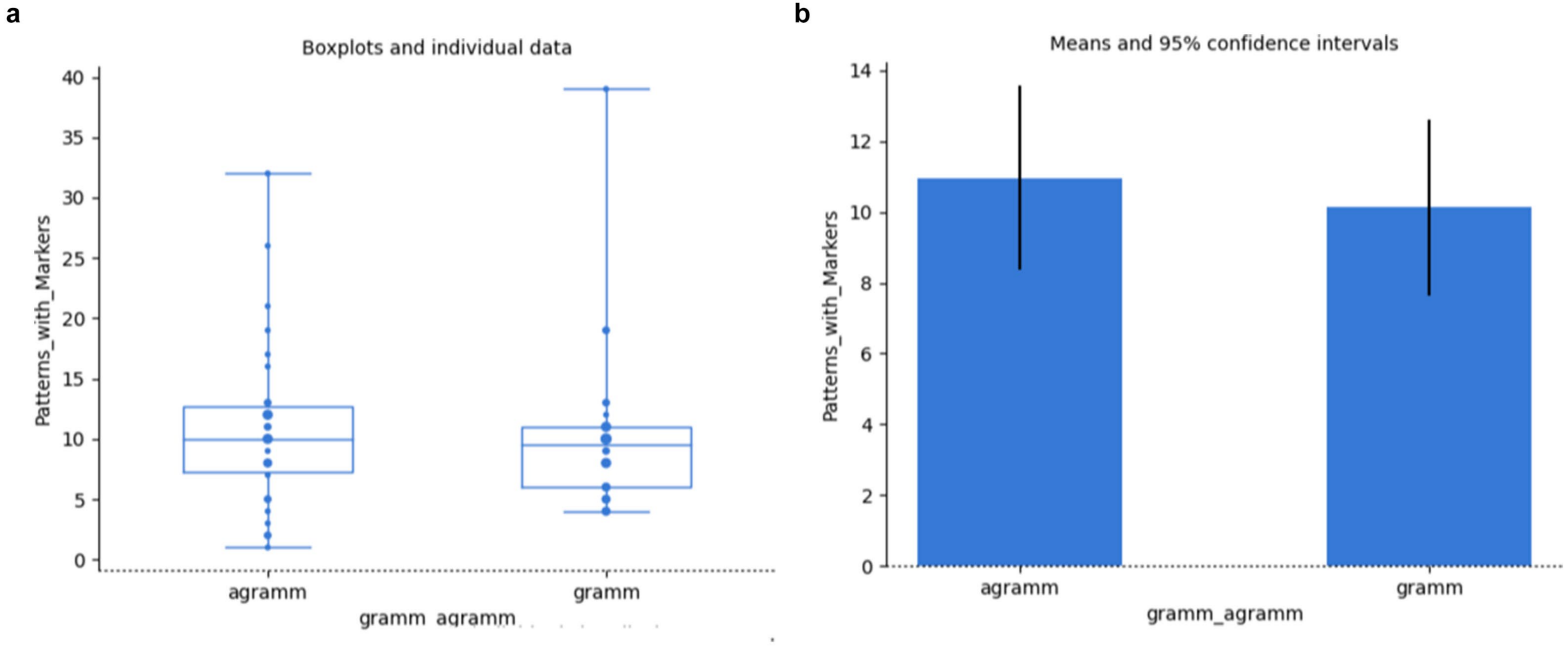
Patterns with markers vs. grammaticality: individual data **(a)**, means **(b)**.

The number of patterns may indicate the complexity of a given behavior. We may generally expect a simpler, more coherent set of behaviors to be reflected by a smaller number of different patterns. Accordingly, we might expect that encountering grammatical errors could result in a larger variety of patterns, too. Systematic, repetitive behavior involves stronger connection between components of a pattern resulting in one or another component becoming the *marker* of that pattern. Since samples with agrammaticality produce a larger number of patterns, we can expect there to be more patterns with markers, too. This is what we see in Figures 10A,B, even though the independent samples Mann–Whitney rank test does not indicate this difference as statistically significant (*U* = 499.50, *p* = 0.467):

As far as the role of fixation in tracking agramamticality is concerned, according to popular opinion, encountering an error generates surprise, which is then reflected by a longer focus on the location of the error and/or an eventual returning/jumping to the source of the agrammaticality (*cf.* Bánréti et al., 2017). Let us now see, if the opinion holds, according to which a longer focusing time should be expected in case agrammaticality is encountered.

**Figure 11:**
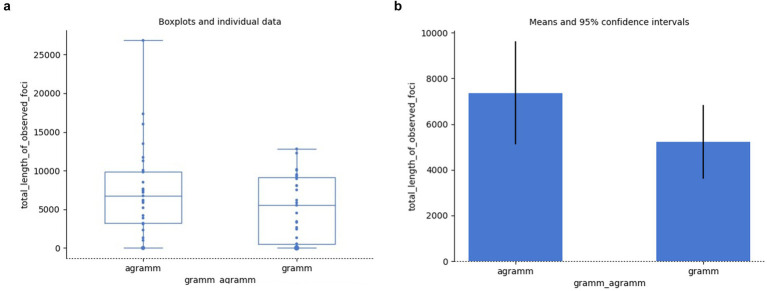
Total duration of fixations relevant to grammaticality: individual data **(a)**, means **(b)**.

Figures 11A, B show the boxplots and individual data regarding the total duration of fixations relevant to grammaticality (i.e., relevant to subject-verb agreement or subject-object differentiation):

Even though we observe higher means of data for the agrammatical samples, this difference is not significant. Result of independent samples Mann–Whitney rank test: *U* = 496.50, *p* = 0.240. In defense of the popular opinion let us then suggest that in each agrammatical sentence the incorrect agreement only concerned a single word, or at most, in case the construction itself was also double checked, two words, with this additional fixation time requiring little extra time from the total duration of the reading.

**Figure 12:**
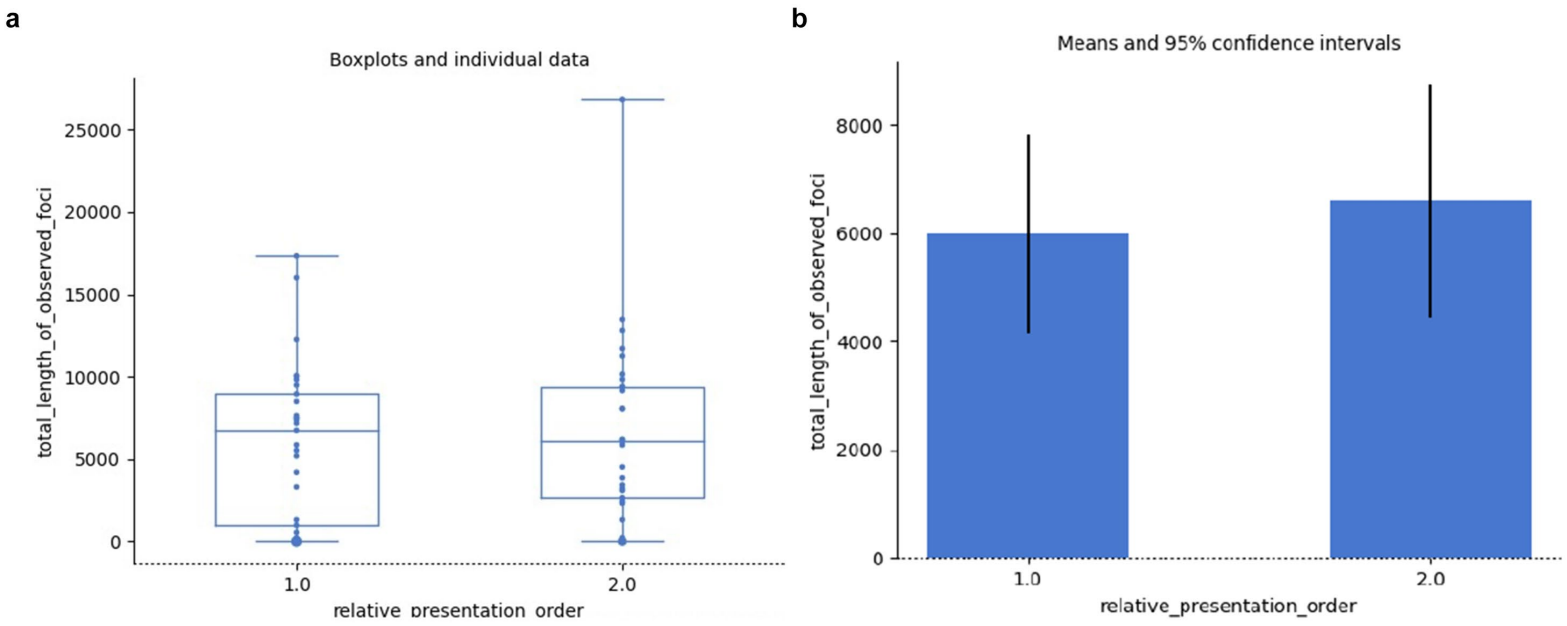
Total duration of fixations. Order of presentation of pairs of samples vs. grammaticality: individual data **(a)**, means **(b)**.

Again, we might assume that eventually the order of presentation (in view of a possible memory effect of whether the grammatical or the agrammatical sentence of the given pair was presented first) could influence the total duration of focusing at the relevant positions; − it turned out it did not, either: Result of independent samples Mann–Whitney rank test: *U* = 396.50, *p* = 0.714; *cf.* Figures 12A,B.

**Figure 13:**
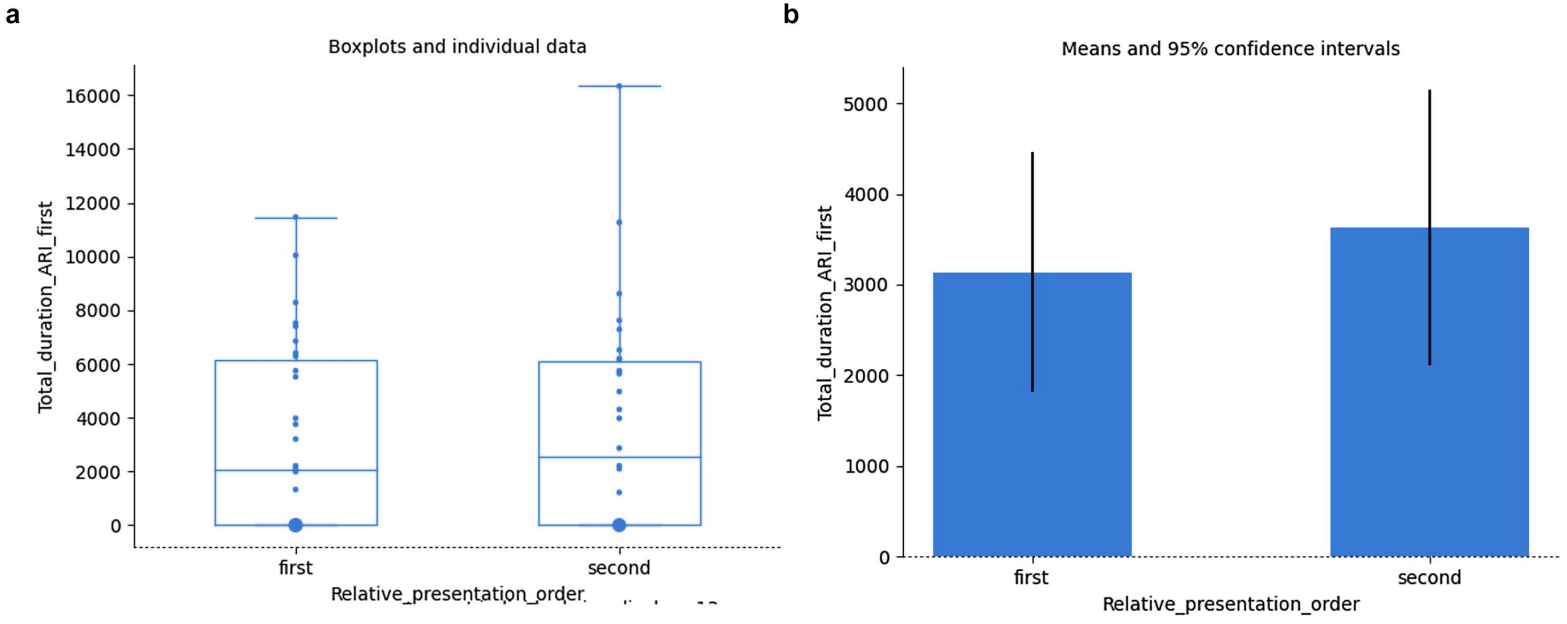
Total duration of fixations on the first words of agreement. Order of presentation vs. grammaticality: individual data **(a)**, means **(b)**.

**Figure 14:**
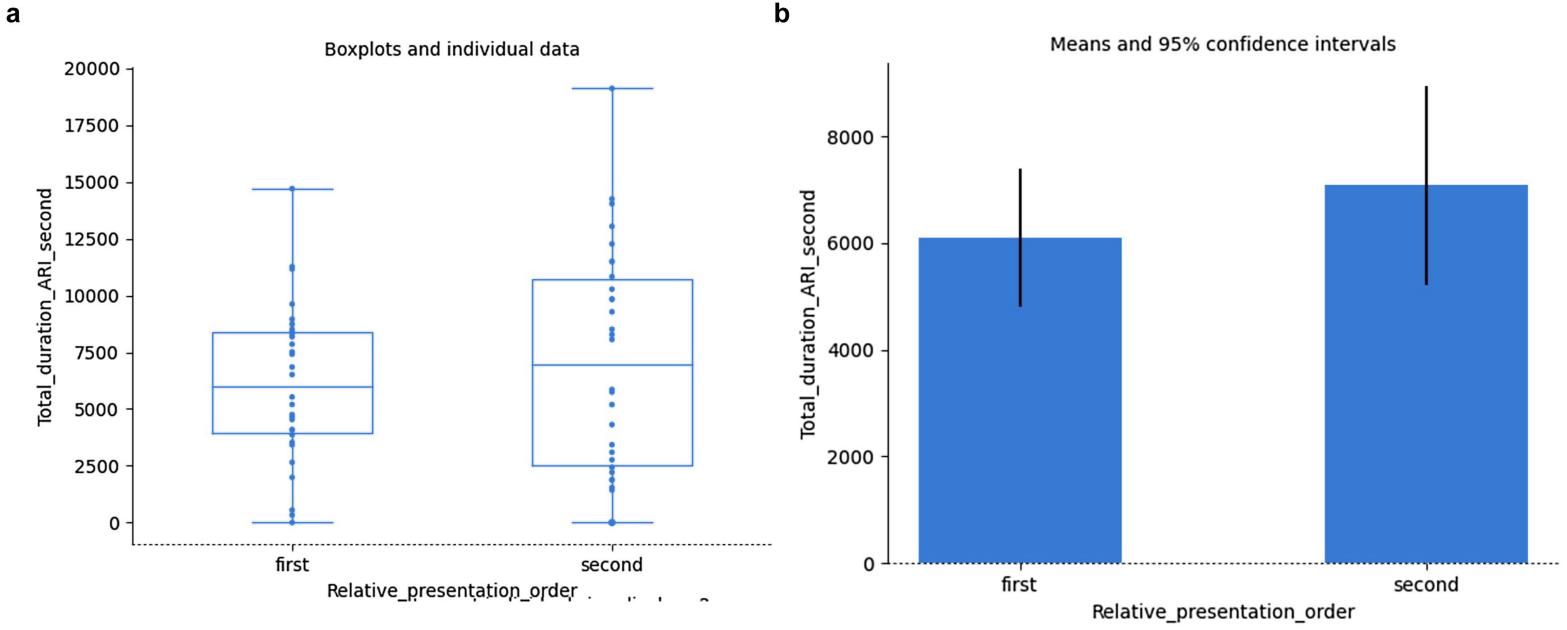
Total duration of fixations on all second words of agreement. Order of presentation vs. grammaticality: individual data **(a)**, means **(b)**.

The same is confirmed by selecting just the two regions of interest (ARI_first and ARI_second) involved in the grammaticality judgement of the given agreement relation within a given sentence. Contrary to the expectation that since the ungrammaticality is only encountered while gazing at the second word of the pair for agreement relation, the total duration of fixation on the first word of the agreement relation was not found to significantly correlate with the total duration of fixation on its second word across all the samples (Spearman’s rank-order correlation: *r_s_*(58) = 0.20, *p* = 0.129). Again, their variation with regard to grammaticality/agrammaticality was not found to be significant either (independent samples *t*-test: *t*(58) = 1.03, *p* = 0.307). The effect of the order of presentation of the grammatical/agrammatical pairs relative to each other, even though the means were not equal, did not prove to be statistically significant either: independent samples Mann–Whitney rank test: *U* = 429.50, *p* = 0.759 for the first word, and Welch’s unequal variances *t*-test for the second word: *t*(56.2) = −0.88, *p* = 0.383; *cf.* Figures 13A,B and 14A,B, respectively:

## 5 Discussion

The results presented above confirmed that the eye tracking data showed only restricted cases of differences in gazing performance between grammatical and agrammatical samples. Such differences are formulated in terms of correlation relation: in most cases demonstrated above we found a certain degree of positive correlation between the pairs of grammatical/agrammatical samples attributed to their similar linguistic material. However, this correlation was only partial, which can be accounted for by the fact that – even though the task was to judge the samples by their grammaticality, syntactic analysis, as found in Szalárdy et al. (2018), is, in general, not automatic. Still, we can attribute the longer fixations observed in certain cases to the fact that grammatical (in our case: syntactic) violation generally require more extensive focusing.

Another possibility for capturing some statistical difference regarding the duration of focusing between the grammatical and agrammatical samples could be attributed to their relative presentation order, due to a possible memory effect. The assumption could be that however random the presentation order of the corresponding grammatical/agrammatical samples of the pairs could be, memory would affect the performance of the observation of the second sample. The results, however, confirmed that relative order does not have a significant effect on error detection while reading, suggesting that presentation order may play a role in individual cases only.

All this leads us to suggest that in the present study it was the experimental task itself that conditioned the observed eye tracking behavior: since the task was to judge in each case the grammaticality of the sample, the subject took this task as given, paying equal attention to each of them, noticing and checking each case of grammatical agreement or the expected morphological case regardless of their presentation order or relying on previous familiarity of the sample. The increased duration of fixation, however, even if proved not to be significant, showed that the agrammaticality of the corresponding samples was noticed and played its proper role in deciding the response as “correct” or “incorrect”.

## 6 Conclusion

The results of this pilot experiment argue for including eye tracking with restricted but certainly useful benefits in the exploration of the behavioral patterns associated with making grammatical judgements through reading. The observed restrictions, however, also point to the necessity of possible further improvements in the experimental paradigm. By considering eye tracking as a component of a more complex experiment, further development could take two additional directions: accompanied by reading aloud, the study of speech performance (including tempo change, restart, repair etc.) could offer additional dimensions for the discovery of behavioral patterns of reading. Furthermore, as a potential direction for future research, synchronizing eye tracking with linguistic ERP studies in reading tasks could widen the categorical spectrum of identifying grammatical violations.

Finally, it has to be noted, that the wider scope of the present conclusions is limited by our study referring to only data from a single subject. The inclusion of the elaboration of the sound recording synchronized with the eye tracking data could also add a further aspect to the detection of behavioral patterns using eye tracking.

## Data Availability

The raw data supporting the conclusions of this article will be made available by the authors, without undue reservation.

## In memoriam

This paper is dedicated to Aron, whose eyes will only be shining in our memories.
