# Supplementary material for: Follow the eyes: gaze and grammaticality
Source: Front Psychol. 2024 Nov 15;15:1415590. doi: 10.3389/fpsyg.2024.1415590 (PMC11616449; doi:10.3389/fpsyg.2024.1415590)
Supplement: Supplementary file 1 [file Table_1.DOC]

PsyScope X B77 started:	 3/26/15   9:45:40
Script file:  Syntax_violation Script
Run on:       cogling
Random Seed: 	232431683

SubjectName: Aron3


Input devices active: Key Mouse Sound 
Timing Device: Macintosh

Trial	Condition	trial start	Stimulus	PutUpBy	Onset	Time	keys	mouse_down		
2	aa1n3s	14476	aa1n3s.bmp	SentenceToRead	325	8788	[SPACE]	0	
3	t1n2s	23590	t1n2s.bmp	SentenceToRead	277	5850	[SPACE]	0	
4	aa1n20	29718	aa1n20.bmp	SentenceToRead	292	9323	[SPACE]	0	
5	aa1n10s	39334	aa1n10s.bmp	SentenceToRead	277	8682	[SPACE]	0	
6	t1n7	48295	t1n7.bmp	SentenceToRead	281	7765	[SPACE]	0	
7	aa1n9s	56342	aa1n9s.bmp	SentenceToRead	275	4900	[SPACE]	0	
8	t1n8s	61518	t1n8s.bmp	SentenceToRead	283	6092	[SPACE]	0	
9	t1n4s	67894	t1n4s.bmp	SentenceToRead	278	5937	[SPACE]	0	
10	t1n7s	74110	t1n7s.bmp	SentenceToRead	282	7661	[SPACE]	0	
11	t1n10s	82054	t1n10s.bmp	SentenceToRead	283	8076	[SPACE]	0	
12	aa1n19	90415	aa1n19.bmp	SentenceToRead	285	10969	[SPACE]	0	
13	aa1n10	101670	aa1n10.bmp	SentenceToRead	291	10108	[SPACE]	0	
14	aa1n14	112070	aa1n14.bmp	SentenceToRead	285	8362	[SPACE]	0	
15	aa1n9	120718	aa1n9.bmp	SentenceToRead	282	7309	[SPACE]	0	
16	aa1n18s	128310	aa1n18s.bmp	SentenceToRead	280	13127	[SPACE]	0	
17	t1n1	141718	t1n1.bmp	SentenceToRead	284	6579	[SPACE]	0	
18	aa1n1s	148584	aa1n1s.bmp	SentenceToRead	285	7160	[SPACE]	0	
19	aa1n7s	156030	aa1n7s.bmp	SentenceToRead	290	8053	[SPACE]	0	
20	aa1n2s	164374	aa1n2s.bmp	SentenceToRead	286	7753	[SPACE]	0	
21	aa1n13s	172414	aa1n13s.bmp	SentenceToRead	282	7157	[SPACE]	0	
22	t1n9	179854	t1n9.bmp	SentenceToRead	278	5801	[SPACE]	0	
23	aa1n6	185934	aa1n6.bmp	SentenceToRead	283	5924	[SPACE]	0	
24	t1n5	192143	t1n5.bmp	SentenceToRead	283	10339	[SPACE]	0	
25	aa1n17	202766	aa1n17.bmp	SentenceToRead	274	5053	[SPACE]	0	
26	aa1n16	208094	aa1n16.bmp	SentenceToRead	290	9349	[SPACE]	0	
27	aa1n7	217734	aa1n7.bmp	SentenceToRead	280	7479	[SPACE]	0	
28	t1n4	225494	t1n4.bmp	SentenceToRead	279	6680	[SPACE]	0	
29	t1n3	232454	t1n3.bmp	SentenceToRead	278	7233	[SPACE]	0	
30	aa1n4s	239968	aa1n4s.bmp	SentenceToRead	282	9707	[SPACE]	0	
31	aa1n8s	249958	aa1n8s.bmp	SentenceToRead	285	20298	[SPACE]	0	
32	t1n3s	270542	t1n3s.bmp	SentenceToRead	279	6264	[SPACE]	0	
33	aa1n1	277086	aa1n1.bmp	SentenceToRead	272	5151	[SPACE]	0	
34	aa1n18	282510	aa1n18.bmp	SentenceToRead	287	11520	[SPACE]	0	
35	t1n1s	294318	t1n1s.bmp	SentenceToRead	283	8116	[SPACE]	0	
36	aa1n20s	302720	aa1n20s.bmp	SentenceToRead	283	10610	[SPACE]	0	
37	t1n2	313614	t1n2.bmp	SentenceToRead	278	6305	[SPACE]	0	
38	aa1n5s	320198	aa1n5s.bmp	SentenceToRead	279	7272	[SPACE]	0	
39	aa1n11	327750	aa1n11.bmp	SentenceToRead	287	5048	[SPACE]	0	
40	aa1n12	333086	aa1n12.bmp	SentenceToRead	274	8821	[SPACE]	0	
41	aa1n16s	342182	aa1n16s.bmp	SentenceToRead	289	8422	[SPACE]	0	
42	aa1n11s	350896	aa1n11s.bmp	SentenceToRead	286	5975	[SPACE]	0	
43	aa1n15	357158	aa1n15.bmp	SentenceToRead	269	7890	[SPACE]	0	
44	aa1n3	365318	aa1n3.bmp	SentenceToRead	286	9481	[SPACE]	0	
45	t1n9s	375086	t1n9s.bmp	SentenceToRead	289	5990	[SPACE]	0	
46	t1n8	381366	t1n8.bmp	SentenceToRead	284	7635	[SPACE]	0	
47	aa1n2	389286	aa1n2.bmp	SentenceToRead	285	6554	[SPACE]	0	
48	t1n6s	396128	t1n6s.bmp	SentenceToRead	288	6229	[SPACE]	0	
49	t1n5s	402646	t1n5s.bmp	SentenceToRead	287	9280	[SPACE]	0	
50	aa1n4	412214	aa1n4.bmp	SentenceToRead	283	10788	[SPACE]	0	
51	t1n6	423286	t1n6.bmp	SentenceToRead	274	5141	[SPACE]	0	
52	aa1n12s	428702	aa1n12s.bmp	SentenceToRead	282	8949	[SPACE]	0	
53	aa1n19s	437934	aa1n19s.bmp	SentenceToRead	281	12254	[SPACE]	0	
54	t1n10	450472	t1n10.bmp	SentenceToRead	282	5051	[SPACE]	0	
55	aa1n14s	455806	aa1n14s.bmp	SentenceToRead	288	7919	[SPACE]	0	
56	aa1n5	464014	aa1n5.bmp	SentenceToRead	275	9084	[SPACE]	0	
57	aa1n8	473374	aa1n8.bmp	SentenceToRead	329	11022	[SPACE]	0	
58	aa1n6s	484726	aa1n6s.bmp	SentenceToRead	297	5398	[SPACE]	0	
59	aa1n15s	490422	aa1n15s.bmp	SentenceToRead	282	8021	[SPACE]	0	
60	aa1n13	498728	aa1n13.bmp	SentenceToRead	276	7361	[SPACE]	0	
61	aa1n17s	506366	aa1n17s.bmp	SentenceToRead	280	5855	[SPACE]	0	
62		512515	NULL	StopRecording	2015	N/A	[N/A]	0	
	
Local system time at start (ms):	1427359540209	
Absolute mac time at start (ms):	3873861441	
=======================================================	
SUMMARY OF TIMING STATISTICS:	
Condition	Event	Actual Onset	Actual Duration	
:	OpenConn	9	750	
:	OpenFile	759	500	
:	Instructions	2259	5900	
:	StartRecording	1509	1250	
Number of trials averaged:	2	
aa1n3s:	SentenceToRead	325	8788	
Number of trials averaged:	1	
t1n2s:	SentenceToRead	277	5850	
Number of trials averaged:	1	
aa1n20:	SentenceToRead	292	9323	
Number of trials averaged:	1	
aa1n10s:	SentenceToRead	277	8682	
Number of trials averaged:	1	
t1n7:	SentenceToRead	281	7765	
Number of trials averaged:	1	
aa1n9s:	SentenceToRead	275	4900	
Number of trials averaged:	1	
t1n8s:	SentenceToRead	283	6092	
Number of trials averaged:	1	
t1n4s:	SentenceToRead	278	5937	
Number of trials averaged:	1	
t1n7s:	SentenceToRead	282	7661	
Number of trials averaged:	1	
t1n10s:	SentenceToRead	283	8076	
Number of trials averaged:	1	
aa1n19:	SentenceToRead	285	10969	
Number of trials averaged:	1	
aa1n10:	SentenceToRead	291	10108	
Number of trials averaged:	1	
aa1n14:	SentenceToRead	285	8362	
Number of trials averaged:	1	
aa1n9:	SentenceToRead	282	7309	
Number of trials averaged:	1	
aa1n18s:	SentenceToRead	280	13127	
Number of trials averaged:	1	
t1n1:	SentenceToRead	284	6579	
Number of trials averaged:	1	
aa1n1s:	SentenceToRead	285	7160	
Number of trials averaged:	1	
aa1n7s:	SentenceToRead	290	8053	
Number of trials averaged:	1	
aa1n2s:	SentenceToRead	286	7753	
Number of trials averaged:	1	
aa1n13s:	SentenceToRead	282	7157	
Number of trials averaged:	1	
t1n9:	SentenceToRead	278	5801	
Number of trials averaged:	1	
aa1n6:	SentenceToRead	283	5924	
Number of trials averaged:	1	
t1n5:	SentenceToRead	283	10339	
Number of trials averaged:	1	
aa1n17:	SentenceToRead	274	5053	
Number of trials averaged:	1	
aa1n16:	SentenceToRead	290	9349	
Number of trials averaged:	1	
aa1n7:	SentenceToRead	280	7479	
Number of trials averaged:	1	
t1n4:	SentenceToRead	279	6680	
Number of trials averaged:	1	
t1n3:	SentenceToRead	278	7233	
Number of trials averaged:	1	
aa1n4s:	SentenceToRead	282	9707	
Number of trials averaged:	1	
aa1n8s:	SentenceToRead	285	20298	
Number of trials averaged:	1	
t1n3s:	SentenceToRead	279	6264	
Number of trials averaged:	1	
aa1n1:	SentenceToRead	272	5151	
Number of trials averaged:	1	
aa1n18:	SentenceToRead	287	11520	
Number of trials averaged:	1	
t1n1s:	SentenceToRead	283	8116	
Number of trials averaged:	1	
aa1n20s:	SentenceToRead	283	10610	
Number of trials averaged:	1	
t1n2:	SentenceToRead	278	6305	
Number of trials averaged:	1	
aa1n5s:	SentenceToRead	279	7272	
Number of trials averaged:	1	
aa1n11:	SentenceToRead	287	5048	
Number of trials averaged:	1	
aa1n12:	SentenceToRead	274	8821	
Number of trials averaged:	1	
aa1n16s:	SentenceToRead	289	8422	
Number of trials averaged:	1	
aa1n11s:	SentenceToRead	286	5975	
Number of trials averaged:	1	
aa1n15:	SentenceToRead	269	7890	
Number of trials averaged:	1	
aa1n3:	SentenceToRead	286	9481	
Number of trials averaged:	1	
t1n9s:	SentenceToRead	289	5990	
Number of trials averaged:	1	
t1n8:	SentenceToRead	284	7635	
Number of trials averaged:	1	
aa1n2:	SentenceToRead	285	6554	
Number of trials averaged:	1	
t1n6s:	SentenceToRead	288	6229	
Number of trials averaged:	1	
t1n5s:	SentenceToRead	287	9280	
Number of trials averaged:	1	
aa1n4:	SentenceToRead	283	10788	
Number of trials averaged:	1	
t1n6:	SentenceToRead	274	5141	
Number of trials averaged:	1	
aa1n12s:	SentenceToRead	282	8949	
Number of trials averaged:	1	
aa1n19s:	SentenceToRead	281	12254	
Number of trials averaged:	1	
t1n10:	SentenceToRead	282	5051	
Number of trials averaged:	1	
aa1n14s:	SentenceToRead	288	7919	
Number of trials averaged:	1	
aa1n5:	SentenceToRead	275	9084	
Number of trials averaged:	1	
aa1n8:	SentenceToRead	329	11022	
Number of trials averaged:	1	
aa1n6s:	SentenceToRead	297	5398	
Number of trials averaged:	1	
aa1n15s:	SentenceToRead	282	8021	
Number of trials averaged:	1	
aa1n13:	SentenceToRead	276	7361	
Number of trials averaged:	1	
aa1n17s:	SentenceToRead	280	5855	
Number of trials averaged:	1	
Total number of trials averaged:	62	
Total number of trials run:	62	
Note: EventDurations reflect actual stimulus durations only for stimuli that are cleared by EndEvent.	
	

=======================================================	
 FULL TIMING STATISTICS:	
Condition		
Trial	START Onset	START Duration	OpenConn Onset	OpenConn Duration	OpenFile Onset	OpenFile Duration	Instructions Onset	Instructions Duration	StartRecording Onset	StartRecording Duration	
1	0	0	3	500	503	500	3004	11301	1003	2000	
62	0	15	15	1000	1015	500	1515	500	2015	500	
Condition	aa1n3s	
Trial	START Onset	START Duration	SentenceToRead Onset	SentenceToRead Duration	
2	0	325	325	8788	
Condition	t1n2s	
Trial	START Onset	START Duration	SentenceToRead Onset	SentenceToRead Duration	
3	0	277	277	5850	
Condition	aa1n20	
Trial	START Onset	START Duration	SentenceToRead Onset	SentenceToRead Duration	
4	0	292	292	9323	
Condition	aa1n10s	
Trial	START Onset	START Duration	SentenceToRead Onset	SentenceToRead Duration	
5	0	277	277	8682	
Condition	t1n7	
Trial	START Onset	START Duration	SentenceToRead Onset	SentenceToRead Duration	
6	0	280	281	7765	
Condition	aa1n9s	
Trial	START Onset	START Duration	SentenceToRead Onset	SentenceToRead Duration	
7	0	275	275	4900	
Condition	t1n8s	
Trial	START Onset	START Duration	SentenceToRead Onset	SentenceToRead Duration	
8	0	283	283	6092	
Condition	t1n4s	
Trial	START Onset	START Duration	SentenceToRead Onset	SentenceToRead Duration	
9	0	277	278	5937	
Condition	t1n7s	
Trial	START Onset	START Duration	SentenceToRead Onset	SentenceToRead Duration	
10	0	282	282	7661	
Condition	t1n10s	
Trial	START Onset	START Duration	SentenceToRead Onset	SentenceToRead Duration	
11	0	283	283	8076	
Condition	aa1n19	
Trial	START Onset	START Duration	SentenceToRead Onset	SentenceToRead Duration	
12	0	284	285	10969	
Condition	aa1n10	
Trial	START Onset	START Duration	SentenceToRead Onset	SentenceToRead Duration	
13	0	291	291	10108	
Condition	aa1n14	
Trial	START Onset	START Duration	SentenceToRead Onset	SentenceToRead Duration	
14	0	284	285	8362	
Condition	aa1n9	
Trial	START Onset	START Duration	SentenceToRead Onset	SentenceToRead Duration	
15	0	281	282	7309	
Condition	aa1n18s	
Trial	START Onset	START Duration	SentenceToRead Onset	SentenceToRead Duration	
16	0	280	280	13127	
Condition	t1n1	
Trial	START Onset	START Duration	SentenceToRead Onset	SentenceToRead Duration	
17	0	284	284	6579	
Condition	aa1n1s	
Trial	START Onset	START Duration	SentenceToRead Onset	SentenceToRead Duration	
18	0	285	285	7160	
Condition	aa1n7s	
Trial	START Onset	START Duration	SentenceToRead Onset	SentenceToRead Duration	
19	0	290	290	8053	
Condition	aa1n2s	
Trial	START Onset	START Duration	SentenceToRead Onset	SentenceToRead Duration	
20	0	286	286	7753	
Condition	aa1n13s	
Trial	START Onset	START Duration	SentenceToRead Onset	SentenceToRead Duration	
21	0	282	282	7157	
Condition	t1n9	
Trial	START Onset	START Duration	SentenceToRead Onset	SentenceToRead Duration	
22	0	278	278	5801	
Condition	aa1n6	
Trial	START Onset	START Duration	SentenceToRead Onset	SentenceToRead Duration	
23	0	283	283	5924	
Condition	t1n5	
Trial	START Onset	START Duration	SentenceToRead Onset	SentenceToRead Duration	
24	0	283	283	10339	
Condition	aa1n17	
Trial	START Onset	START Duration	SentenceToRead Onset	SentenceToRead Duration	
25	0	274	274	5053	
Condition	aa1n16	
Trial	START Onset	START Duration	SentenceToRead Onset	SentenceToRead Duration	
26	0	290	290	9349	
Condition	aa1n7	
Trial	START Onset	START Duration	SentenceToRead Onset	SentenceToRead Duration	
27	0	279	280	7479	
Condition	t1n4	
Trial	START Onset	START Duration	SentenceToRead Onset	SentenceToRead Duration	
28	0	279	279	6680	
Condition	t1n3	
Trial	START Onset	START Duration	SentenceToRead Onset	SentenceToRead Duration	
29	0	278	278	7233	
Condition	aa1n4s	
Trial	START Onset	START Duration	SentenceToRead Onset	SentenceToRead Duration	
30	0	282	282	9707	
Condition	aa1n8s	
Trial	START Onset	START Duration	SentenceToRead Onset	SentenceToRead Duration	
31	0	284	285	20298	
Condition	t1n3s	
Trial	START Onset	START Duration	SentenceToRead Onset	SentenceToRead Duration	
32	0	279	279	6264	
Condition	aa1n1	
Trial	START Onset	START Duration	SentenceToRead Onset	SentenceToRead Duration	
33	0	272	272	5151	
Condition	aa1n18	
Trial	START Onset	START Duration	SentenceToRead Onset	SentenceToRead Duration	
34	0	287	287	11520	
Condition	t1n1s	
Trial	START Onset	START Duration	SentenceToRead Onset	SentenceToRead Duration	
35	0	282	283	8116	
Condition	aa1n20s	
Trial	START Onset	START Duration	SentenceToRead Onset	SentenceToRead Duration	
36	0	283	283	10610	
Condition	t1n2	
Trial	START Onset	START Duration	SentenceToRead Onset	SentenceToRead Duration	
37	0	278	278	6305	
Condition	aa1n5s	
Trial	START Onset	START Duration	SentenceToRead Onset	SentenceToRead Duration	
38	0	278	279	7272	
Condition	aa1n11	
Trial	START Onset	START Duration	SentenceToRead Onset	SentenceToRead Duration	
39	0	287	287	5048	
Condition	aa1n12	
Trial	START Onset	START Duration	SentenceToRead Onset	SentenceToRead Duration	
40	0	274	274	8821	
Condition	aa1n16s	
Trial	START Onset	START Duration	SentenceToRead Onset	SentenceToRead Duration	
41	0	289	289	8422	
Condition	aa1n11s	
Trial	START Onset	START Duration	SentenceToRead Onset	SentenceToRead Duration	
42	0	286	286	5975	
Condition	aa1n15	
Trial	START Onset	START Duration	SentenceToRead Onset	SentenceToRead Duration	
43	0	269	269	7890	
Condition	aa1n3	
Trial	START Onset	START Duration	SentenceToRead Onset	SentenceToRead Duration	
44	0	285	286	9481	
Condition	t1n9s	
Trial	START Onset	START Duration	SentenceToRead Onset	SentenceToRead Duration	
45	0	289	289	5990	
Condition	t1n8	
Trial	START Onset	START Duration	SentenceToRead Onset	SentenceToRead Duration	
46	0	284	284	7635	
Condition	aa1n2	
Trial	START Onset	START Duration	SentenceToRead Onset	SentenceToRead Duration	
47	0	285	285	6554	
Condition	t1n6s	
Trial	START Onset	START Duration	SentenceToRead Onset	SentenceToRead Duration	
48	0	288	288	6229	
Condition	t1n5s	
Trial	START Onset	START Duration	SentenceToRead Onset	SentenceToRead Duration	
49	0	287	287	9280	
Condition	aa1n4	
Trial	START Onset	START Duration	SentenceToRead Onset	SentenceToRead Duration	
50	0	283	283	10788	
Condition	t1n6	
Trial	START Onset	START Duration	SentenceToRead Onset	SentenceToRead Duration	
51	0	274	274	5141	
Condition	aa1n12s	
Trial	START Onset	START Duration	SentenceToRead Onset	SentenceToRead Duration	
52	0	282	282	8949	
Condition	aa1n19s	
Trial	START Onset	START Duration	SentenceToRead Onset	SentenceToRead Duration	
53	0	281	281	12254	
Condition	t1n10	
Trial	START Onset	START Duration	SentenceToRead Onset	SentenceToRead Duration	
54	0	282	282	5051	
Condition	aa1n14s	
Trial	START Onset	START Duration	SentenceToRead Onset	SentenceToRead Duration	
55	0	288	288	7919	
Condition	aa1n5	
Trial	START Onset	START Duration	SentenceToRead Onset	SentenceToRead Duration	
56	0	275	275	9084	
Condition	aa1n8	
Trial	START Onset	START Duration	SentenceToRead Onset	SentenceToRead Duration	
57	0	329	329	11022	
Condition	aa1n6s	
Trial	START Onset	START Duration	SentenceToRead Onset	SentenceToRead Duration	
58	0	297	297	5398	
Condition	aa1n15s	
Trial	START Onset	START Duration	SentenceToRead Onset	SentenceToRead Duration	
59	0	282	282	8021	
Condition	aa1n13	
Trial	START Onset	START Duration	SentenceToRead Onset	SentenceToRead Duration	
60	0	276	276	7361	
Condition	aa1n17s	
Trial	START Onset	START Duration	SentenceToRead Onset	SentenceToRead Duration	
61	0	280	280	5855	
Note: EventDurations reflect actual stimulus durations	
      only for stimuli that are cleared by EndEvent.	
