# Supplementary material for: Follow the eyes: gaze and grammaticality
Source: Front Psychol. 2024 Nov 15;15:1415590. doi: 10.3389/fpsyg.2024.1415590 (PMC11616449; doi:10.3389/fpsyg.2024.1415590)
Supplement: Supplementary file 2 [file Table_2.DOC]

   1	 0.0000	++					DataPoint(0)
   2	 0.0000	STARTUP.BMP				DataPoint(0)
   3	 0.0000	F	0.0446	(0.39, 0.47)		DataPointRange(0 - 3)
   4	 0.0331	S	0.0222	(0.39, 0.46)-(0.42, 0.38)	DataPointRange(4 - 5)	0.0222	0.07	3.02	3.77
   5	 0.0664	F	0.2319	(0.42, 0.37)		DataPointRange(6 - 26)
   6	 0.2873	S	0.1325	(0.42, 0.38)-(0.42, 0.38)	DataPointRange(27 - 38)	0.1325	0.01	0.05	42.96
   7	 0.4309	F	0.3868	(0.43, 0.38)		DataPointRange(39 - 73)
   8	 0.4971	R					DataPoint(45)
   9	 0.8068	S	0.0218	(0.42, 0.39)-(0.48, 0.40)	DataPointRange(74 - 75)	0.0218	0.06	2.71	5.25
  10	 0.8401	F	0.1543	(0.47, 0.39)		DataPointRange(76 - 89)
  11	 0.9835	S	0.0440	(0.47, 0.38)-(0.36, 0.64)	DataPointRange(90 - 93)	0.0440	0.23	5.15	10.07
  12	 1.0388	F	0.2093	(0.35, 0.63)		DataPointRange(94 - 112)
  13	 1.2373	S	0.1877	(0.35, 0.64)-(0.48, 0.55)	DataPointRange(113 - 129)	0.1877	0.15	0.78	74.02
  14	 1.4360	F	0.5525	(0.49, 0.58)		DataPointRange(130 - 179)
  15	 1.9776	S	0.0331	(0.49, 0.57)-(0.39, 0.48)	DataPointRange(180 - 182)	0.0331	0.13	3.79	5.59
  16	 2.0217	F	0.2983	(0.38, 0.43)		DataPointRange(183 - 209)
  17	 2.3090	S	0.0225	(0.39, 0.45)-(0.44, 0.43)	DataPointRange(210 - 211)	0.0225	0.06	2.48	4.14
  18	 2.3419	F	0.4417	(0.47, 0.44)		DataPointRange(212 - 251)
  19	 2.7725	S	0.0331	(0.46, 0.46)-(0.35, 0.41)	DataPointRange(252 - 254)	0.0331	0.12	3.68	7.66
  20	 2.8167	F	0.4200	(0.35, 0.40)		DataPointRange(255 - 292)
  21	 3.2256	S	0.0111	(0.33, 0.37)-(0.35, 0.36)	DataPointRange(293 - 293)	0.0111	0.02	1.47	1.47
  22	 3.2479	F	0.3093	(0.38, 0.39)		DataPointRange(294 - 321)
  23	 3.5460	S	0.0112	(0.39, 0.38)-(0.42, 0.37)	DataPointRange(322 - 322)	0.0112	0.03	3.06	3.06
  24	 3.5681	F	0.2209	(0.45, 0.40)		DataPointRange(323 - 342)
  25	 3.7780	S	0.0222	(0.45, 0.41)-(0.50, 0.40)	DataPointRange(343 - 344)	0.0222	0.05	2.36	4.40
  26	 3.8112	F	0.4420	(0.50, 0.40)		DataPointRange(345 - 384)
  27	 4.2421	S	0.0111	(0.50, 0.38)-(0.52, 0.38)	DataPointRange(385 - 385)	0.0111	0.03	2.49	2.49
  28	 4.2641	F	0.3532	(0.54, 0.39)		DataPointRange(386 - 417)
  29	 4.6064	S	0.0109	(0.55, 0.40)-(0.57, 0.39)	DataPointRange(418 - 418)	0.0109	0.02	2.10	2.10
  30	 4.6286	F	0.4306	(0.62, 0.39)		DataPointRange(419 - 457)
  31	 5.0482	S	0.0551	(0.64, 0.38)-(0.30, 0.38)	DataPointRange(458 - 462)	0.0551	0.33	6.06	8.41
  32	 5.1148	F	0.4196	(0.34, 0.40)		DataPointRange(463 - 500)
  33	 5.5235	S	0.0109	(0.36, 0.40)-(0.38, 0.39)	DataPointRange(501 - 501)	0.0109	0.02	1.68	1.68
  34	 5.5454	F	0.2432	(0.41, 0.40)		DataPointRange(502 - 523)
  35	 5.7774	S	0.0112	(0.41, 0.39)-(0.44, 0.40)	DataPointRange(524 - 524)	0.0112	0.04	3.25	3.25
  36	 5.7995	F	0.2428	(0.45, 0.40)		DataPointRange(525 - 546)
  37	 6.0315	S	0.0219	(0.45, 0.40)-(0.51, 0.41)	DataPointRange(547 - 548)	0.0219	0.07	3.01	5.20
  38	 6.0643	F	0.2764	(0.53, 0.40)		DataPointRange(549 - 573)
  39	 6.3297	S	0.0220	(0.54, 0.40)-(0.60, 0.40)	DataPointRange(574 - 575)	0.0220	0.06	2.66	6.02
  40	 6.3626	F	0.2102	(0.60, 0.41)		DataPointRange(576 - 594)
  41	 6.5615	S	0.0443	(0.60, 0.40)-(0.37, 0.44)	DataPointRange(595 - 598)	0.0443	0.23	5.14	8.55
  42	 6.6168	F	0.2210	(0.34, 0.42)		DataPointRange(599 - 618)
  43	 6.8266	S	0.0112	(0.34, 0.43)-(0.35, 0.45)	DataPointRange(619 - 619)	0.0112	0.02	1.74	1.74
  44	 6.8489	F	0.1657	(0.39, 0.42)		DataPointRange(620 - 634)
  45	 7.0034	S	0.0552	(0.40, 0.43)-(1.87, -0.31)	DataPointRange(635 - 639)	0.0552	1.57	28.43	134.03
  46	 7.0701	F	0.0107	(1.87, -0.31)		DataPointRange(640 - 640)
  47	 7.0701	S	0.0768	(1.87, -0.31)-(0.43, 0.42)	DataPointRange(641 - 647)	0.0768	1.54	20.04	145.75
  48	 7.1579	F	0.0331	(0.43, 0.37)		DataPointRange(648 - 650)
  49	 7.1799	S	0.0111	(0.43, 0.36)-(0.45, 0.42)	DataPointRange(651 - 651)	0.0111	0.05	4.50	4.50
  50	 7.2024	F	0.2205	(0.45, 0.41)		DataPointRange(652 - 671)
  51	 7.4119	S	0.0222	(0.46, 0.41)-(0.53, 0.39)	DataPointRange(672 - 673)	0.0222	0.07	3.23	5.95
  52	 7.4452	F	0.3314	(0.54, 0.41)		DataPointRange(674 - 703)
  53	 7.7656	S	0.0442	(0.54, 0.41)-(0.33, 0.47)	DataPointRange(704 - 707)	0.0442	0.21	4.86	8.41
  54	 7.8208	F	0.2766	(0.33, 0.47)		DataPointRange(708 - 732)
  55	 8.0859	S	0.0221	(0.32, 0.48)-(0.39, 0.44)	DataPointRange(733 - 734)	0.0221	0.08	3.47	4.98
  56	 8.1192	F	0.1544	(0.40, 0.45)		DataPointRange(735 - 748)
  57	 8.2625	S	0.0221	(0.41, 0.45)-(0.36, 0.50)	DataPointRange(749 - 750)	0.0221	0.06	2.60	4.96
  58	 8.2957	F	0.2210	(0.38, 0.52)		DataPointRange(751 - 770)
  59	 8.5058	S	0.0109	(0.37, 0.51)-(0.39, 0.48)	DataPointRange(771 - 771)	0.0109	0.03	2.33	2.33
  60	 8.5278	F	0.2431	(0.39, 0.45)		DataPointRange(772 - 793)
  61	 8.7597	S	0.0112	(0.39, 0.46)-(0.43, 0.45)	DataPointRange(794 - 794)	0.0112	0.04	3.68	3.68
  62	 8.7819	F	0.2539	(0.45, 0.47)		DataPointRange(795 - 817)
  63	 9.0248	S	0.0110	(0.46, 0.46)-(0.49, 0.45)	DataPointRange(818 - 818)	0.0110	0.03	2.76	2.76
  64	 9.0471	F	0.2433	(0.50, 0.46)		DataPointRange(819 - 840)
  65	 9.2792	S	0.0218	(0.49, 0.48)-(0.58, 0.44)	DataPointRange(841 - 842)	0.0218	0.09	4.27	4.59
  66	 9.3122	F	0.2100	(0.58, 0.45)		DataPointRange(843 - 861)
  67	 9.5109	S	0.0332	(0.59, 0.45)-(0.39, 0.48)	DataPointRange(862 - 864)	0.0332	0.20	6.10	10.47
  68	 9.5556	F	0.2979	(0.35, 0.46)		DataPointRange(865 - 891)
  69	 9.8424	S	0.0221	(0.36, 0.48)-(0.43, 0.49)	DataPointRange(892 - 893)	0.0221	0.07	3.09	4.63
  70	 9.8756	F	0.1656	(0.43, 0.48)		DataPointRange(894 - 908)
  71	10.0301	S	0.1437	(0.43, 0.49)-(0.36, 0.43)	DataPointRange(909 - 921)	0.1437	0.08	0.57	17.32
  72	10.1849	F	0.0108	(0.35, 0.38)		DataPointRange(922 - 922)
  73	10.1849	S	0.0108	(0.35, 0.38)-(0.38, 0.40)	DataPointRange(923 - 923)	0.0108	0.03	3.09	3.09
  74	10.2069	F	0.3201	(0.38, 0.47)		DataPointRange(924 - 952)
  75	10.5163	S	0.0218	(0.38, 0.46)-(0.44, 0.47)	DataPointRange(953 - 954)	0.0218	0.07	3.00	4.55
  76	10.5493	F	0.2650	(0.44, 0.48)		DataPointRange(955 - 978)
  77	10.8032	S	0.0111	(0.44, 0.49)-(0.43, 0.49)	DataPointRange(979 - 979)	0.0111	0.01	0.57	0.57
  78	10.8253	F	0.2653	(0.40, 0.46)		DataPointRange(980 - 1003)
  79	11.0796	S	0.0774	(0.40, 0.44)-(0.42, 0.47)	DataPointRange(1004 - 1010)	0.0774	0.03	0.37	8.09
  80	11.1680	F	0.4525	(0.42, 0.47)		DataPointRange(1011 - 1051)
  81	11.6095	S	0.0222	(0.43, 0.48)-(0.48, 0.49)	DataPointRange(1052 - 1053)	0.0222	0.05	2.34	4.04
  82	11.6427	F	0.1768	(0.50, 0.47)		DataPointRange(1054 - 1069)
  83	11.8089	S	0.0770	(0.50, 0.47)-(0.48, 0.46)	DataPointRange(1070 - 1076)	0.0770	0.02	0.28	18.28
  84	11.8969	F	0.1104	(0.51, 0.47)		DataPointRange(1077 - 1086)
  85	11.9963	S	0.1877	(0.51, 0.48)-(0.46, 0.41)	DataPointRange(1087 - 1103)	0.1877	0.08	0.42	137.07
  86	12.1951	F	0.0113	(0.48, 0.38)		DataPointRange(1104 - 1104)
  87	12.1951	S	0.0113	(0.48, 0.38)-(0.46, 0.46)	DataPointRange(1105 - 1105)	0.0113	0.06	5.47	5.47
  88	12.2173	F	0.1878	(0.47, 0.49)		DataPointRange(1106 - 1122)
  89	12.3938	S	0.0222	(0.46, 0.47)-(0.39, 0.46)	DataPointRange(1123 - 1124)	0.0222	0.08	3.55	6.88
  90	12.4270	F	0.3647	(0.37, 0.50)		DataPointRange(1125 - 1157)
  91	12.7806	S	0.0111	(0.37, 0.49)-(0.40, 0.48)	DataPointRange(1158 - 1158)	0.0111	0.03	3.00	3.00
  92	12.8030	F	0.2760	(0.41, 0.47)		DataPointRange(1159 - 1183)
  93	13.0680	S	0.0110	(0.40, 0.48)-(0.38, 0.45)	DataPointRange(1184 - 1184)	0.0110	0.04	3.26	3.26
  94	13.0902	F	0.3643	(0.37, 0.40)		DataPointRange(1185 - 1217)
  95	13.4433	S	0.1548	(0.38, 0.42)-(0.36, 0.41)	DataPointRange(1218 - 1231)	0.1548	0.02	0.15	34.03
  96	13.6090	F	0.8508	(0.38, 0.41)		DataPointRange(1232 - 1308)
  97	14.3161	aa1n3s.bmp				DataPoint(1296)
  98	14.4044	Start					DataPoint(1304)
  99	14.4485	S	0.0447	(0.38, 0.38)-(0.23, 0.20)	DataPointRange(1309 - 1312)	0.0447	0.20	4.48	8.52
 100	14.5037	F	0.1106	(0.25, 0.21)		DataPointRange(1313 - 1322)
 101	14.6031	S	0.0223	(0.24, 0.21)-(0.18, 0.15)	DataPointRange(1323 - 1324)	0.0223	0.07	3.25	7.55
 102	14.6362	F	0.2099	(0.18, 0.13)		DataPointRange(1325 - 1343)
 103	14.8351	S	0.0223	(0.18, 0.13)-(0.23, 0.13)	DataPointRange(1344 - 1345)	0.0223	0.05	2.14	3.48
 104	14.8681	F	0.3537	(0.26, 0.14)		DataPointRange(1346 - 1377)
 105	15.2106	S	0.0222	(0.26, 0.15)-(0.33, 0.15)	DataPointRange(1378 - 1379)	0.0222	0.07	3.16	5.44
 106	15.2441	F	0.2095	(0.35, 0.15)		DataPointRange(1380 - 1398)
 107	15.4425	S	0.0333	(0.34, 0.15)-(0.43, 0.15)	DataPointRange(1399 - 1401)	0.0333	0.09	2.63	4.09
 108	15.4868	F	0.4528	(0.45, 0.16)		DataPointRange(1402 - 1442)
 109	15.9286	S	0.0331	(0.48, 0.15)-(0.62, 0.17)	DataPointRange(1443 - 1445)	0.0331	0.13	4.03	6.25
 110	15.9728	F	0.2211	(0.64, 0.17)		DataPointRange(1446 - 1465)
 111	16.1829	S	0.0220	(0.63, 0.18)-(0.69, 0.15)	DataPointRange(1466 - 1467)	0.0220	0.07	3.13	5.60
 112	16.2163	F	0.4082	(0.72, 0.17)		DataPointRange(1468 - 1504)
 113	16.6136	S	0.0665	(0.74, 0.18)-(0.19, 0.21)	DataPointRange(1505 - 1510)	0.0665	0.54	8.19	11.70
 114	16.6908	F	0.2876	(0.16, 0.27)		DataPointRange(1511 - 1536)
 115	16.9672	S	0.0218	(0.15, 0.28)-(0.23, 0.23)	DataPointRange(1537 - 1538)	0.0218	0.09	4.03	7.14
 116	17.0001	F	0.2540	(0.27, 0.25)		DataPointRange(1539 - 1561)
 117	17.2435	S	0.0330	(0.28, 0.24)-(0.44, 0.25)	DataPointRange(1562 - 1564)	0.0330	0.16	4.77	7.10
 118	17.2875	F	0.2430	(0.49, 0.25)		DataPointRange(1565 - 1586)
 119	17.5194	S	0.0226	(0.49, 0.25)-(0.58, 0.25)	DataPointRange(1587 - 1588)	0.0226	0.09	4.17	6.61
 120	17.5526	F	0.3978	(0.57, 0.24)		DataPointRange(1589 - 1624)
 121	17.9392	S	0.0442	(0.56, 0.24)-(0.73, 0.24)	DataPointRange(1625 - 1628)	0.0442	0.17	3.83	6.09
 122	17.9945	F	0.2096	(0.74, 0.24)		DataPointRange(1629 - 1647)
 123	18.1931	S	0.0222	(0.73, 0.23)-(0.79, 0.24)	DataPointRange(1648 - 1649)	0.0222	0.06	2.53	4.60
 124	18.2263	F	0.1989	(0.82, 0.24)		DataPointRange(1650 - 1667)
 125	18.4139	S	0.0776	(0.82, 0.25)-(0.17, 0.30)	DataPointRange(1668 - 1674)	0.0776	0.65	8.34	12.14
 126	18.5027	F	0.2980	(0.16, 0.33)		DataPointRange(1675 - 1701)
 127	18.7898	S	0.0223	(0.15, 0.35)-(0.25, 0.34)	DataPointRange(1702 - 1703)	0.0223	0.10	4.40	7.01
 128	18.8230	F	0.2097	(0.26, 0.35)		DataPointRange(1704 - 1722)
 129	19.0218	S	0.0219	(0.26, 0.35)-(0.35, 0.35)	DataPointRange(1723 - 1724)	0.0219	0.09	3.93	5.34
 130	19.0548	F	0.3203	(0.36, 0.34)		DataPointRange(1725 - 1753)
 131	19.3641	S	0.0444	(0.36, 0.34)-(0.53, 0.32)	DataPointRange(1754 - 1757)	0.0444	0.18	4.05	7.12
 132	19.4194	F	0.2429	(0.54, 0.34)		DataPointRange(1758 - 1779)
 133	19.6514	S	0.0109	(0.55, 0.32)-(0.58, 0.35)	DataPointRange(1780 - 1780)	0.0109	0.04	3.86	3.86
 134	19.6733	F	0.1768	(0.62, 0.33)		DataPointRange(1781 - 1796)
 135	19.8392	S	0.0220	(0.63, 0.30)-(0.72, 0.31)	DataPointRange(1797 - 1798)	0.0220	0.09	4.10	5.30
 136	19.8722	F	0.2541	(0.74, 0.34)		DataPointRange(1799 - 1821)
 137	20.1152	S	0.0664	(0.74, 0.34)-(0.44, 0.39)	DataPointRange(1822 - 1827)	0.0664	0.30	4.59	16.25
 138	20.1929	F	0.1872	(0.43, 0.39)		DataPointRange(1828 - 1844)
 139	20.3692	S	0.0441	(0.43, 0.41)-(0.18, 0.44)	DataPointRange(1845 - 1848)	0.0441	0.25	5.74	10.05
 140	20.4246	F	0.2209	(0.15, 0.46)		DataPointRange(1849 - 1868)
 141	20.6345	S	0.0331	(0.15, 0.45)-(0.29, 0.43)	DataPointRange(1869 - 1871)	0.0331	0.14	4.26	6.65
 142	20.6787	F	0.2540	(0.32, 0.44)		DataPointRange(1872 - 1894)
 143	20.9216	S	0.0223	(0.32, 0.47)-(0.36, 0.42)	DataPointRange(1895 - 1896)	0.0223	0.06	2.70	4.00
 144	20.9549	F	0.2539	(0.41, 0.42)		DataPointRange(1897 - 1919)
 145	21.1978	S	0.0220	(0.42, 0.43)-(0.46, 0.40)	DataPointRange(1920 - 1921)	0.0220	0.05	2.19	5.07
 146	21.2311	F	0.0994	(0.47, 0.41)		DataPointRange(1922 - 1930)
 147	21.3192	S	0.2210	(0.47, 0.40)-(0.20, 0.49)	DataPointRange(1931 - 1950)	0.2210	0.28	1.26	223.68
 148	21.5514	F	0.0333	(0.21, 0.45)		DataPointRange(1951 - 1953)
 149	21.5734	S	0.0113	(0.22, 0.43)-(0.21, 0.49)	DataPointRange(1954 - 1954)	0.0113	0.05	4.18	4.18
 150	21.5953	F	0.0555	(0.21, 0.49)		DataPointRange(1955 - 1959)
 151	21.6394	S	0.0221	(0.21, 0.48)-(0.16, 0.54)	DataPointRange(1960 - 1961)	0.0221	0.07	3.20	4.89
 152	21.6726	F	0.2322	(0.15, 0.55)		DataPointRange(1962 - 1982)
 153	21.8938	S	0.0225	(0.16, 0.56)-(0.24, 0.53)	DataPointRange(1983 - 1984)	0.0225	0.08	3.51	7.03
 154	21.9270	F	0.2098	(0.27, 0.54)		DataPointRange(1985 - 2003)
 155	22.1258	S	0.0222	(0.27, 0.54)-(0.32, 0.54)	DataPointRange(2004 - 2005)	0.0222	0.05	2.38	4.40
 156	22.1588	F	0.2207	(0.36, 0.53)		DataPointRange(2006 - 2025)
 157	22.3686	S	0.0220	(0.36, 0.52)-(0.42, 0.56)	DataPointRange(2026 - 2027)	0.0220	0.07	2.99	4.80
 158	22.4016	F	0.2984	(0.41, 0.53)		DataPointRange(2028 - 2054)
 159	22.6892	S	0.0220	(0.41, 0.52)-(0.46, 0.54)	DataPointRange(2055 - 2056)	0.0220	0.05	2.05	3.04
 160	22.7223	F	0.1324	(0.46, 0.52)		DataPointRange(2057 - 2068)
 161	22.8437	S	0.0553	(0.45, 0.51)-(0.32, 0.20)	DataPointRange(2069 - 2073)	0.0553	0.27	4.84	8.77
 162	22.9104	F	0.6182	(0.30, 0.15)		DataPointRange(2074 - 2129)
 163	23.0092	End					DataPoint(2083)
 164	23.2986	t1n2s.bmp				DataPoint(2109)
 165	23.3295	Start					DataPoint(2112)
 166	23.5175	S	0.0222	(0.29, 0.15)-(0.19, 0.12)	DataPointRange(2130 - 2131)	0.0222	0.11	4.73	8.31
 167	23.5505	F	0.1656	(0.17, 0.15)		DataPointRange(2132 - 2146)
 168	23.7054	S	0.0220	(0.17, 0.14)-(0.25, 0.15)	DataPointRange(2147 - 2148)	0.0220	0.08	3.64	5.52
 169	23.7384	F	0.2101	(0.27, 0.16)		DataPointRange(2149 - 2167)
 170	23.9376	S	0.0331	(0.26, 0.16)-(0.43, 0.17)	DataPointRange(2168 - 2170)	0.0331	0.17	5.05	8.10
 171	23.9815	F	0.2541	(0.48, 0.17)		DataPointRange(2171 - 2193)
 172	24.2245	S	0.0220	(0.48, 0.16)-(0.56, 0.16)	DataPointRange(2194 - 2195)	0.0220	0.08	3.55	4.86
 173	24.2576	F	0.2763	(0.60, 0.16)		DataPointRange(2196 - 2220)
 174	24.5227	S	0.0552	(0.59, 0.15)-(0.19, 0.21)	DataPointRange(2221 - 2225)	0.0552	0.41	7.45	11.47
 175	24.5890	F	0.2649	(0.18, 0.24)		DataPointRange(2226 - 2249)
 176	24.8429	S	0.0221	(0.19, 0.24)-(0.25, 0.23)	DataPointRange(2250 - 2251)	0.0221	0.06	2.76	4.51
 177	24.8761	F	0.2319	(0.26, 0.25)		DataPointRange(2252 - 2272)
 178	25.0970	S	0.0110	(0.27, 0.25)-(0.29, 0.25)	DataPointRange(2273 - 2273)	0.0110	0.02	2.07	2.07
 179	25.1192	F	0.1439	(0.33, 0.24)		DataPointRange(2274 - 2286)
 180	25.2516	S	0.0334	(0.33, 0.24)-(0.45, 0.25)	DataPointRange(2287 - 2289)	0.0334	0.12	3.54	5.76
 181	25.2958	F	0.2431	(0.46, 0.24)		DataPointRange(2290 - 2311)
 182	25.5284	S	0.0105	(0.46, 0.24)-(0.49, 0.23)	DataPointRange(2312 - 2312)	0.0105	0.03	2.60	2.60
 183	25.5501	F	0.2206	(0.53, 0.24)		DataPointRange(2313 - 2332)
 184	25.7599	S	0.0552	(0.52, 0.22)-(0.14, 0.32)	DataPointRange(2333 - 2337)	0.0552	0.38	6.89	10.39
 185	25.8263	F	0.2206	(0.15, 0.34)		DataPointRange(2338 - 2357)
 186	26.0360	S	0.0331	(0.16, 0.32)-(0.28, 0.33)	DataPointRange(2358 - 2360)	0.0331	0.11	3.42	6.15
 187	26.0800	F	0.4089	(0.28, 0.33)		DataPointRange(2361 - 2397)
 188	26.4777	S	0.0221	(0.29, 0.33)-(0.36, 0.31)	DataPointRange(2398 - 2399)	0.0221	0.07	3.12	4.65
 189	26.5108	F	0.3645	(0.37, 0.33)		DataPointRange(2400 - 2432)
 190	26.8644	S	0.0441	(0.37, 0.33)-(0.57, 0.35)	DataPointRange(2433 - 2436)	0.0441	0.20	4.52	6.85
 191	26.9198	F	0.2100	(0.60, 0.34)		DataPointRange(2437 - 2455)
 192	27.1184	S	0.0333	(0.61, 0.35)-(0.72, 0.34)	DataPointRange(2456 - 2458)	0.0333	0.11	3.37	6.58
 193	27.1627	F	0.3204	(0.75, 0.33)		DataPointRange(2459 - 2487)
 194	27.4720	S	0.0776	(0.76, 0.34)-(0.19, 0.42)	DataPointRange(2488 - 2494)	0.0776	0.57	7.30	13.50
 195	27.5605	F	0.4418	(0.18, 0.44)		DataPointRange(2495 - 2534)
 196	27.9911	S	0.0333	(0.16, 0.42)-(0.35, 0.41)	DataPointRange(2535 - 2537)	0.0333	0.19	5.56	6.89
 197	28.0354	F	0.5191	(0.36, 0.41)		DataPointRange(2538 - 2584)
 198	28.5434	S	0.0221	(0.35, 0.39)-(0.41, 0.41)	DataPointRange(2585 - 2586)	0.0221	0.06	2.67	4.59
 199	28.5768	F	0.2868	(0.41, 0.41)		DataPointRange(2587 - 2612)
 200	28.8527	S	0.0332	(0.42, 0.41)-(0.29, 0.30)	DataPointRange(2613 - 2615)	0.0332	0.15	4.60	8.04
 201	28.8968	F	0.1324	(0.28, 0.29)		DataPointRange(2616 - 2627)
 202	29.0182	S	0.0332	(0.26, 0.29)-(0.21, 0.15)	DataPointRange(2628 - 2630)	0.0332	0.12	3.74	5.09
 203	29.0627	F	0.7069	(0.22, 0.13)		DataPointRange(2631 - 2694)
 204	29.1399	End					DataPoint(2638)
 205	29.4939	aa1n20.bmp				DataPoint(2670)
 206	29.5706	Start					DataPoint(2677)
 207	29.7585	S	0.0222	(0.22, 0.13)-(0.30, 0.15)	DataPointRange(2695 - 2696)	0.0222	0.08	3.56	5.13
 208	29.7917	F	0.1544	(0.31, 0.16)		DataPointRange(2697 - 2710)
 209	29.9352	S	0.0997	(0.31, 0.16)-(0.39, 0.15)	DataPointRange(2711 - 2719)	0.0997	0.08	0.80	13.57
 210	30.0459	F	0.0221	(0.40, 0.14)		DataPointRange(2720 - 2721)
 211	30.0568	S	0.0112	(0.40, 0.13)-(0.40, 0.16)	DataPointRange(2722 - 2722)	0.0112	0.02	1.80	1.80
 212	30.0789	F	0.2761	(0.40, 0.17)		DataPointRange(2723 - 2747)
 213	30.3440	S	0.0110	(0.39, 0.15)-(0.42, 0.16)	DataPointRange(2748 - 2748)	0.0110	0.03	2.80	2.80
 214	30.3663	F	0.2427	(0.46, 0.16)		DataPointRange(2749 - 2770)
 215	30.5978	S	0.0334	(0.46, 0.17)-(0.61, 0.16)	DataPointRange(2771 - 2773)	0.0334	0.15	4.58	6.20
 216	30.6421	F	0.1656	(0.64, 0.16)		DataPointRange(2774 - 2788)
 217	30.7969	S	0.0330	(0.65, 0.16)-(0.73, 0.17)	DataPointRange(2789 - 2791)	0.0330	0.09	2.58	5.03
 218	30.8409	F	0.1767	(0.74, 0.16)		DataPointRange(2792 - 2807)
 219	31.0067	S	0.0774	(0.75, 0.16)-(0.18, 0.21)	DataPointRange(2808 - 2814)	0.0774	0.57	7.32	13.10
 220	31.0952	F	0.3091	(0.20, 0.23)		DataPointRange(2815 - 2842)
 221	31.3933	S	0.0221	(0.20, 0.24)-(0.29, 0.25)	DataPointRange(2843 - 2844)	0.0221	0.09	3.87	5.56
 222	31.4267	F	0.2426	(0.28, 0.24)		DataPointRange(2845 - 2866)
 223	31.6586	S	0.0218	(0.29, 0.23)-(0.35, 0.24)	DataPointRange(2867 - 2868)	0.0218	0.06	2.85	4.86
 224	31.6915	F	0.1657	(0.40, 0.24)		DataPointRange(2869 - 2883)
 225	31.8462	S	0.0220	(0.40, 0.25)-(0.45, 0.24)	DataPointRange(2884 - 2885)	0.0220	0.05	2.08	4.25
 226	31.8792	F	0.3204	(0.47, 0.25)		DataPointRange(2886 - 2914)
 227	32.1887	S	0.0332	(0.47, 0.24)-(0.55, 0.22)	DataPointRange(2915 - 2917)	0.0332	0.08	2.49	4.07
 228	32.2328	F	0.2874	(0.60, 0.23)		DataPointRange(2918 - 2943)
 229	32.5089	S	0.0221	(0.60, 0.22)-(0.69, 0.23)	DataPointRange(2944 - 2945)	0.0221	0.09	4.09	4.99
 230	32.5422	F	0.3536	(0.70, 0.24)		DataPointRange(2946 - 2977)
 231	32.8850	S	0.0328	(0.68, 0.25)-(0.79, 0.25)	DataPointRange(2978 - 2980)	0.0328	0.12	3.52	5.71
 232	32.9289	F	0.1767	(0.81, 0.25)		DataPointRange(2981 - 2996)
 233	33.0947	S	0.0968	(0.81, 0.25)-(0.18, 0.33)	DataPointRange(2997 - 3003)	0.0968	0.63	6.46	13.85
 234	33.1961	F	0.2959	(0.17, 0.35)		DataPointRange(3004 - 3030)
 235	33.4810	S	0.0331	(0.17, 0.36)-(0.31, 0.33)	DataPointRange(3031 - 3033)	0.0331	0.14	4.33	6.94
 236	33.5252	F	0.3427	(0.34, 0.33)		DataPointRange(3034 - 3064)
 237	33.8571	S	0.0218	(0.33, 0.33)-(0.42, 0.34)	DataPointRange(3065 - 3066)	0.0218	0.09	4.15	4.90
 238	33.8901	F	0.2430	(0.42, 0.35)		DataPointRange(3067 - 3088)
 239	34.1219	S	0.0332	(0.41, 0.34)-(0.55, 0.33)	DataPointRange(3089 - 3091)	0.0332	0.14	4.08	6.48
 240	34.1662	F	0.2536	(0.59, 0.35)		DataPointRange(3092 - 3114)
 241	34.4089	S	0.0223	(0.59, 0.34)-(0.66, 0.37)	DataPointRange(3115 - 3116)	0.0223	0.07	3.28	5.94
 242	34.4421	F	0.1988	(0.66, 0.34)		DataPointRange(3117 - 3134)
 243	34.6297	S	0.0112	(0.65, 0.34)-(0.69, 0.34)	DataPointRange(3135 - 3135)	0.0112	0.04	3.59	3.59
 244	34.6522	F	0.1765	(0.72, 0.34)		DataPointRange(3136 - 3151)
 245	34.8177	S	0.0668	(0.73, 0.36)-(0.19, 0.40)	DataPointRange(3152 - 3157)	0.0668	0.54	8.06	11.89
 246	34.8950	F	0.2875	(0.19, 0.44)		DataPointRange(3158 - 3183)
 247	35.1713	S	0.0220	(0.19, 0.42)-(0.25, 0.45)	DataPointRange(3184 - 3185)	0.0220	0.06	2.93	4.70
 248	35.2045	F	0.3092	(0.25, 0.45)		DataPointRange(3186 - 3213)
 249	35.5026	S	0.0221	(0.25, 0.44)-(0.30, 0.41)	DataPointRange(3214 - 3215)	0.0221	0.06	2.59	4.58
 250	35.5358	F	0.2762	(0.30, 0.42)		DataPointRange(3216 - 3240)
 251	35.8009	S	0.0331	(0.30, 0.43)-(0.40, 0.42)	DataPointRange(3241 - 3243)	0.0331	0.11	3.24	5.61
 252	35.8454	F	0.2317	(0.43, 0.42)		DataPointRange(3244 - 3264)
 253	36.0661	S	0.0220	(0.43, 0.43)-(0.54, 0.40)	DataPointRange(3265 - 3266)	0.0220	0.11	4.96	6.29
 254	36.0991	F	0.2541	(0.56, 0.42)		DataPointRange(3267 - 3289)
 255	36.3422	S	0.0221	(0.57, 0.43)-(0.64, 0.41)	DataPointRange(3290 - 3291)	0.0221	0.07	3.21	4.69
 256	36.3753	F	0.1328	(0.62, 0.40)		DataPointRange(3292 - 3303)
 257	36.4967	S	0.0222	(0.61, 0.41)-(0.69, 0.40)	DataPointRange(3304 - 3305)	0.0222	0.08	3.42	3.99
 258	36.5301	F	0.0660	(0.71, 0.41)		DataPointRange(3306 - 3311)
 259	36.5851	S	0.0663	(0.72, 0.41)-(0.20, 0.49)	DataPointRange(3312 - 3317)	0.0663	0.52	7.90	13.21
 260	36.6624	F	0.2097	(0.20, 0.52)		DataPointRange(3318 - 3336)
 261	36.8611	S	0.0110	(0.21, 0.56)-(0.19, 0.53)	DataPointRange(3337 - 3337)	0.0110	0.03	2.64	2.64
 262	36.8831	F	0.2210	(0.16, 0.53)		DataPointRange(3338 - 3357)
 263	37.0932	S	0.0220	(0.15, 0.52)-(0.25, 0.49)	DataPointRange(3358 - 3359)	0.0220	0.11	4.96	6.48
 264	37.1263	F	0.1441	(0.27, 0.51)		DataPointRange(3360 - 3372)
 265	37.2590	S	0.0221	(0.26, 0.52)-(0.35, 0.51)	DataPointRange(3373 - 3374)	0.0221	0.09	4.03	6.85
 266	37.2921	F	0.2652	(0.40, 0.51)		DataPointRange(3375 - 3398)
 267	37.5461	S	0.0444	(0.39, 0.51)-(0.59, 0.50)	DataPointRange(3399 - 3402)	0.0444	0.20	4.48	7.86
 268	37.6015	F	1.0822	(0.66, 0.52)		DataPointRange(3403 - 3500)
 269	38.6727	S	0.0663	(0.64, 0.49)-(0.33, 0.14)	DataPointRange(3501 - 3506)	0.0663	0.41	6.11	11.05
 270	38.7500	End					DataPoint(3507)
 271	38.7500	F	0.2322	(0.33, 0.15)		DataPointRange(3507 - 3527)
 272	38.9711	S	0.0221	(0.34, 0.16)-(0.24, 0.14)	DataPointRange(3528 - 3529)	0.0221	0.10	4.35	5.92
 273	39.0043	F	0.4309	(0.23, 0.15)		DataPointRange(3530 - 3568)
 274	39.0815	aa1n10s.bmp				DataPoint(3537)
 275	39.1618	Start					DataPoint(3544)
 276	39.4243	S	0.0218	(0.22, 0.14)-(0.31, 0.14)	DataPointRange(3569 - 3570)	0.0218	0.09	3.94	4.95
 277	39.4571	F	0.2321	(0.30, 0.15)		DataPointRange(3571 - 3591)
 278	39.6781	S	0.0222	(0.31, 0.16)-(0.37, 0.15)	DataPointRange(3592 - 3593)	0.0222	0.06	2.79	4.52
 279	39.7113	F	0.2319	(0.38, 0.16)		DataPointRange(3594 - 3614)
 280	39.9322	S	0.0439	(0.39, 0.15)-(0.53, 0.17)	DataPointRange(3615 - 3618)	0.0439	0.14	3.28	5.82
 281	39.9879	F	0.1322	(0.55, 0.17)		DataPointRange(3619 - 3630)
 282	40.1087	S	0.0552	(0.55, 0.18)-(0.15, 0.24)	DataPointRange(3631 - 3635)	0.0552	0.40	7.27	9.95
 283	40.1752	F	0.2436	(0.14, 0.24)		DataPointRange(3636 - 3657)
 284	40.4072	S	0.0221	(0.14, 0.25)-(0.22, 0.24)	DataPointRange(3658 - 3659)	0.0221	0.07	3.32	6.26
 285	40.4400	F	0.3316	(0.22, 0.25)		DataPointRange(3660 - 3689)
 286	40.7607	S	0.0223	(0.23, 0.25)-(0.28, 0.24)	DataPointRange(3690 - 3691)	0.0223	0.05	2.11	5.04
 287	40.7939	F	0.3090	(0.32, 0.23)		DataPointRange(3692 - 3719)
 288	41.0917	S	0.0221	(0.32, 0.22)-(0.42, 0.25)	DataPointRange(3720 - 3721)	0.0221	0.10	4.63	6.98
 289	41.1250	F	0.1769	(0.42, 0.24)		DataPointRange(3722 - 3736)
 290	41.2913	S	0.0217	(0.42, 0.24)-(0.52, 0.23)	DataPointRange(3737 - 3738)	0.0217	0.10	4.62	4.88
 291	41.3244	F	0.1873	(0.54, 0.25)		DataPointRange(3739 - 3755)
 292	41.5008	S	0.0330	(0.54, 0.25)-(0.68, 0.24)	DataPointRange(3756 - 3758)	0.0330	0.14	4.19	7.04
 293	41.5449	F	0.2432	(0.72, 0.25)		DataPointRange(3759 - 3780)
 294	41.7769	S	0.0221	(0.72, 0.24)-(0.79, 0.25)	DataPointRange(3781 - 3782)	0.0221	0.07	3.33	4.79
 295	41.8099	F	0.0553	(0.78, 0.25)		DataPointRange(3783 - 3787)
 296	41.8539	S	0.0773	(0.79, 0.26)-(0.18, 0.32)	DataPointRange(3788 - 3794)	0.0773	0.60	7.82	15.59
 297	41.9423	F	0.3426	(0.19, 0.32)		DataPointRange(3795 - 3825)
 298	42.2740	S	0.0331	(0.19, 0.32)-(0.33, 0.33)	DataPointRange(3826 - 3828)	0.0331	0.14	4.16	6.75
 299	42.3180	F	0.2653	(0.35, 0.33)		DataPointRange(3829 - 3852)
 300	42.5723	S	0.0444	(0.35, 0.32)-(0.53, 0.35)	DataPointRange(3853 - 3856)	0.0444	0.18	4.02	8.18
 301	42.6274	F	0.2870	(0.56, 0.35)		DataPointRange(3857 - 3882)
 302	42.9033	S	0.0222	(0.56, 0.34)-(0.63, 0.33)	DataPointRange(3883 - 3884)	0.0222	0.08	3.49	7.11
 303	42.9365	F	0.2212	(0.64, 0.34)		DataPointRange(3885 - 3904)
 304	43.1469	S	0.4353	(0.63, 0.33)-(0.19, 0.43)	DataPointRange(3905 - 3906)	0.4353	0.45	1.04	1.07
 305	43.5903	F	0.0863	(0.19, 0.44)		DataPointRange(3907 - 3914)
 306	43.6657	S	0.0224	(0.19, 0.44)-(0.28, 0.42)	DataPointRange(3915 - 3916)	0.0224	0.08	3.76	5.45
 307	43.6988	F	0.2319	(0.28, 0.41)		DataPointRange(3917 - 3937)
 308	43.9198	S	0.0219	(0.27, 0.42)-(0.34, 0.40)	DataPointRange(3938 - 3939)	0.0219	0.07	3.15	4.69
 309	43.9528	F	0.1877	(0.35, 0.41)		DataPointRange(3940 - 3956)
 310	44.1294	S	0.0331	(0.36, 0.41)-(0.46, 0.42)	DataPointRange(3957 - 3959)	0.0331	0.10	3.06	6.00
 311	44.1737	F	0.1656	(0.49, 0.40)		DataPointRange(3960 - 3974)
 312	44.3283	S	0.0224	(0.50, 0.42)-(0.39, 0.42)	DataPointRange(3975 - 3976)	0.0224	0.11	4.78	6.45
 313	44.3618	F	0.2097	(0.37, 0.40)		DataPointRange(3977 - 3995)
 314	44.5606	S	0.0331	(0.37, 0.40)-(0.51, 0.40)	DataPointRange(3996 - 3998)	0.0331	0.14	4.17	8.30
 315	44.6046	F	0.3534	(0.55, 0.40)		DataPointRange(3999 - 4030)
 316	44.9472	S	0.0330	(0.55, 0.41)-(0.71, 0.40)	DataPointRange(4031 - 4033)	0.0330	0.15	4.70	8.43
 317	44.9911	F	0.2653	(0.71, 0.41)		DataPointRange(4034 - 4057)
 318	45.2453	S	0.0666	(0.72, 0.40)-(0.18, 0.49)	DataPointRange(4058 - 4063)	0.0666	0.55	8.20	11.78
 319	45.3225	F	0.3206	(0.19, 0.52)		DataPointRange(4064 - 4092)
 320	45.6320	S	0.0219	(0.18, 0.52)-(0.24, 0.49)	DataPointRange(4093 - 4094)	0.0219	0.07	2.99	7.13
 321	45.6650	F	0.2872	(0.25, 0.52)		DataPointRange(4095 - 4120)
 322	45.9414	S	0.0440	(0.25, 0.51)-(0.43, 0.52)	DataPointRange(4121 - 4124)	0.0440	0.18	4.09	6.52
 323	45.9966	F	0.2760	(0.46, 0.52)		DataPointRange(4125 - 4149)
 324	46.2616	S	0.0221	(0.47, 0.52)-(0.53, 0.52)	DataPointRange(4150 - 4151)	0.0221	0.06	2.94	3.51
 325	46.2948	F	0.3976	(0.57, 0.51)		DataPointRange(4152 - 4187)
 326	46.6814	S	0.0330	(0.55, 0.51)-(0.68, 0.51)	DataPointRange(4188 - 4190)	0.0330	0.12	3.75	7.15
 327	46.7254	F	0.1656	(0.73, 0.52)		DataPointRange(4191 - 4205)
 328	46.8801	S	0.0772	(0.74, 0.51)-(0.13, 0.63)	DataPointRange(4206 - 4212)	0.0772	0.62	7.98	12.63
 329	46.9686	F	0.4639	(0.14, 0.65)		DataPointRange(4213 - 4254)
 330	47.4213	S	0.0996	(0.13, 0.68)-(1.87, -0.30)	DataPointRange(4255 - 4263)	0.0996	1.89	18.96	280.18
 331	47.5324	F	0.0216	(1.86, -0.32)		DataPointRange(4264 - 4265)
 332	47.5430	S	0.0110	(1.86, -0.32)-(1.86, -0.32)	DataPointRange(4266 - 4266)	0.0110	0.00	0.00	0.00
 333	47.5652	F	0.0108	(-0.82, 0.21)		DataPointRange(4267 - 4267)
 334	47.5652	S	0.0108	(-0.82, 0.21)-(1.87, -0.28)	DataPointRange(4268 - 4268)	0.0108	2.71	251.11	251.11
 335	47.5869	F	0.0331	(1.87, -0.30)		DataPointRange(4269 - 4271)
 336	47.6092	S	0.0993	(1.86, -0.32)-(0.22, 0.15)	DataPointRange(4272 - 4280)	0.0993	1.68	16.93	213.97
 337	47.7085	End					DataPoint(4280)
 338	47.7198	F	0.0218	(0.22, 0.13)		DataPointRange(4281 - 4282)
 339	47.7311	S	0.0105	(0.23, 0.13)-(0.23, 0.18)	DataPointRange(4283 - 4283)	0.0105	0.04	3.74	3.74
 340	47.7527	F	0.4639	(0.23, 0.20)		DataPointRange(4284 - 4325)
 341	48.1395	t1n7.bmp					DataPoint(4319)
 342	48.2055	S	0.0111	(0.23, 0.20)-(0.23, 0.18)	DataPointRange(4326 - 4326)	0.0111	0.01	1.23	1.23
 343	48.2419	F	0.1514	(0.21, 0.15)		DataPointRange(4327 - 4340)
 344	48.2496	Start					DataPoint(4328)
 345	48.3823	S	0.0221	(0.22, 0.15)-(0.28, 0.16)	DataPointRange(4341 - 4342)	0.0221	0.06	2.93	5.53
 346	48.4155	F	0.2319	(0.28, 0.15)		DataPointRange(4343 - 4363)
 347	48.6364	S	0.0331	(0.27, 0.15)-(0.46, 0.15)	DataPointRange(4364 - 4366)	0.0331	0.19	5.67	7.03
 348	48.6805	F	0.3316	(0.50, 0.17)		DataPointRange(4367 - 4396)
 349	49.0012	S	0.0441	(0.51, 0.18)-(0.69, 0.18)	DataPointRange(4397 - 4400)	0.0441	0.18	4.08	6.46
 350	49.0563	F	0.2430	(0.71, 0.17)		DataPointRange(4401 - 4422)
 351	49.2882	S	0.0221	(0.71, 0.15)-(0.79, 0.17)	DataPointRange(4423 - 4424)	0.0221	0.08	3.71	5.42
 352	49.3212	F	0.2100	(0.80, 0.17)		DataPointRange(4425 - 4443)
 353	49.5201	S	0.0664	(0.80, 0.15)-(0.24, 0.22)	DataPointRange(4444 - 4449)	0.0664	0.56	8.48	11.31
 354	49.5977	F	0.2127	(0.22, 0.24)		DataPointRange(4450 - 4468)
 355	49.8009	S	0.0173	(0.22, 0.21)-(0.19, 0.26)	DataPointRange(4469 - 4470)	0.0173	0.05	2.70	3.04
 356	49.8292	F	0.0774	(0.17, 0.25)		DataPointRange(4471 - 4477)
 357	49.8958	S	0.8497	(0.18, 0.23)-(0.52, 0.23)	DataPointRange(4478 - 4479)	0.8497	0.35	0.41	1.89
 358	50.7527	F	0.2069	(0.53, 0.25)		DataPointRange(4480 - 4494)
 359	50.9452	S	0.0335	(0.54, 0.24)-(0.66, 0.25)	DataPointRange(4495 - 4497)	0.0335	0.12	3.57	11.25
 360	50.9894	F	0.1656	(0.67, 0.24)		DataPointRange(4498 - 4512)
 361	51.1440	S	0.0220	(0.67, 0.23)-(0.74, 0.26)	DataPointRange(4513 - 4514)	0.0220	0.07	3.41	4.95
 362	51.1772	F	0.2649	(0.73, 0.25)		DataPointRange(4515 - 4538)
 363	51.4310	S	0.0222	(0.73, 0.24)-(0.80, 0.25)	DataPointRange(4539 - 4540)	0.0222	0.07	3.14	5.18
 364	51.4642	F	0.2210	(0.84, 0.26)		DataPointRange(4541 - 4560)
 365	51.6740	S	0.0665	(0.84, 0.27)-(0.30, 0.30)	DataPointRange(4561 - 4566)	0.0665	0.55	8.20	13.53
 366	51.7515	F	0.0993	(0.28, 0.33)		DataPointRange(4567 - 4575)
 367	51.8399	S	0.0330	(0.28, 0.32)-(0.17, 0.35)	DataPointRange(4576 - 4578)	0.0330	0.11	3.38	5.64
 368	51.8842	F	0.3092	(0.16, 0.36)		DataPointRange(4579 - 4606)
 369	52.1824	S	0.0330	(0.16, 0.36)-(0.34, 0.35)	DataPointRange(4607 - 4609)	0.0330	0.18	5.43	7.26
 370	52.2264	F	0.3092	(0.37, 0.34)		DataPointRange(4610 - 4637)
 371	52.5246	S	0.2099	(0.36, 0.33)-(0.48, 0.33)	DataPointRange(4638 - 4656)	0.2099	0.12	0.55	232.45
 372	52.7455	F	0.0111	(0.45, 0.22)		DataPointRange(4657 - 4657)
 373	52.7455	S	0.0111	(0.45, 0.22)-(0.46, 0.27)	DataPointRange(4658 - 4658)	0.0111	0.05	4.09	4.09
 374	52.7676	F	0.1989	(0.48, 0.33)		DataPointRange(4659 - 4676)
 375	52.9553	S	0.0224	(0.48, 0.33)-(0.53, 0.33)	DataPointRange(4677 - 4678)	0.0224	0.05	2.10	4.22
 376	52.9886	F	0.1435	(0.54, 0.33)		DataPointRange(4679 - 4691)
 377	53.1211	S	0.0331	(0.54, 0.35)-(0.64, 0.34)	DataPointRange(4692 - 4694)	0.0331	0.10	2.94	5.95
 378	53.1651	F	0.2102	(0.65, 0.34)		DataPointRange(4695 - 4713)
 379	53.3641	S	0.0222	(0.65, 0.34)-(0.74, 0.34)	DataPointRange(4714 - 4715)	0.0222	0.09	3.84	5.20
 380	53.3973	F	0.1435	(0.77, 0.33)		DataPointRange(4716 - 4728)
 381	53.5299	S	0.0777	(0.76, 0.33)-(0.19, 0.45)	DataPointRange(4729 - 4735)	0.0777	0.58	7.41	12.89
 382	53.6183	F	0.3424	(0.18, 0.47)		DataPointRange(4736 - 4766)
 383	53.9497	S	0.0222	(0.19, 0.48)-(0.25, 0.42)	DataPointRange(4767 - 4768)	0.0222	0.08	3.61	4.99
 384	53.9828	F	0.2429	(0.25, 0.43)		DataPointRange(4769 - 4790)
 385	54.2146	S	0.0443	(0.26, 0.42)-(0.45, 0.42)	DataPointRange(4791 - 4794)	0.0443	0.19	4.18	7.12
 386	54.2699	F	0.2763	(0.47, 0.42)		DataPointRange(4795 - 4819)
 387	54.5352	S	0.0221	(0.47, 0.42)-(0.56, 0.41)	DataPointRange(4820 - 4821)	0.0221	0.09	3.95	7.02
 388	54.5688	F	0.2867	(0.59, 0.41)		DataPointRange(4822 - 4847)
 389	54.8446	S	0.0223	(0.59, 0.42)-(0.68, 0.43)	DataPointRange(4848 - 4849)	0.0223	0.09	4.25	5.28
 390	54.8777	F	0.2649	(0.71, 0.42)		DataPointRange(4850 - 4873)
 391	55.1318	S	0.0330	(0.72, 0.45)-(0.79, 0.44)	DataPointRange(4874 - 4876)	0.0330	0.08	2.37	6.03
 392	55.1758	F	0.2539	(0.80, 0.41)		DataPointRange(4877 - 4899)
 393	55.4187	S	0.0664	(0.81, 0.41)-(0.43, 0.24)	DataPointRange(4900 - 4905)	0.0664	0.40	6.00	9.94
 394	55.4966	F	0.1764	(0.42, 0.21)		DataPointRange(4906 - 4921)
 395	55.6619	S	0.0219	(0.42, 0.22)-(0.36, 0.18)	DataPointRange(4922 - 4923)	0.0219	0.07	3.32	5.31
 396	55.6948	F	0.3535	(0.34, 0.16)		DataPointRange(4924 - 4955)
 397	55.7614	End					DataPoint(4930)
 398	56.0373	S	0.0222	(0.34, 0.17)-(0.24, 0.17)	DataPointRange(4956 - 4957)	0.0222	0.09	4.25	5.46
 399	56.0705	F	0.1988	(0.25, 0.18)		DataPointRange(4958 - 4975)
 400	56.0943	aa1n9s.bmp				DataPoint(4960)
 401	56.1809	Start					DataPoint(4968)
 402	56.2582	S	0.0111	(0.25, 0.18)-(0.22, 0.17)	DataPointRange(4976 - 4976)	0.0111	0.04	3.18	3.18
 403	56.2804	F	0.0552	(0.19, 0.15)		DataPointRange(4977 - 4981)
 404	56.3246	S	0.0110	(0.19, 0.16)-(0.19, 0.16)	DataPointRange(4982 - 4982)	0.0110	0.00	0.14	0.14
 405	56.3466	F	0.0111	(0.14, 0.20)		DataPointRange(4983 - 4983)
 406	56.3466	S	0.0111	(0.14, 0.20)-(0.19, 0.15)	DataPointRange(4984 - 4984)	0.0111	0.06	5.41	5.41
 407	56.3687	F	0.0883	(0.19, 0.15)		DataPointRange(4985 - 4992)
 408	56.4460	S	0.0220	(0.19, 0.14)-(0.28, 0.16)	DataPointRange(4993 - 4994)	0.0220	0.09	4.10	6.37
 409	56.4793	F	0.1545	(0.28, 0.16)		DataPointRange(4995 - 5008)
 410	56.6227	S	0.0221	(0.28, 0.16)-(0.35, 0.15)	DataPointRange(5009 - 5010)	0.0221	0.08	3.41	4.62
 411	56.6561	F	0.1765	(0.40, 0.16)		DataPointRange(5011 - 5026)
 412	56.8218	S	0.1764	(0.40, 0.18)-(0.50, 0.20)	DataPointRange(5027 - 5042)	0.1764	0.10	0.57	146.28
 413	57.0093	F	0.2320	(0.49, 0.17)		DataPointRange(5043 - 5063)
 414	57.2303	S	0.0223	(0.49, 0.16)-(0.56, 0.18)	DataPointRange(5064 - 5065)	0.0223	0.08	3.65	6.58
 415	57.2634	F	0.0441	(0.57, 0.17)		DataPointRange(5066 - 5069)
 416	57.2965	S	0.0110	(0.58, 0.18)-(0.58, 0.19)	DataPointRange(5070 - 5070)	0.0110	0.01	0.77	0.77
 417	57.3191	F	0.0107	(0.54, 0.22)		DataPointRange(5071 - 5071)
 418	57.3191	S	0.0107	(0.54, 0.22)-(0.59, 0.18)	DataPointRange(5072 - 5072)	0.0107	0.06	5.86	5.86
 419	57.3408	F	0.1657	(0.58, 0.17)		DataPointRange(5073 - 5087)
 420	57.4954	S	0.0111	(0.58, 0.18)-(0.60, 0.16)	DataPointRange(5088 - 5088)	0.0111	0.02	1.73	1.73
 421	57.5177	F	0.1214	(0.64, 0.17)		DataPointRange(5089 - 5099)
 422	57.6280	S	0.0111	(0.64, 0.16)-(0.64, 0.17)	DataPointRange(5100 - 5100)	0.0111	0.00	0.44	0.44
 423	57.6500	F	0.0331	(0.61, 0.19)		DataPointRange(5101 - 5103)
 424	57.6721	S	0.0110	(0.60, 0.20)-(0.64, 0.17)	DataPointRange(5104 - 5104)	0.0110	0.04	3.96	3.96
 425	57.6942	F	0.0332	(0.63, 0.17)		DataPointRange(5105 - 5107)
 426	57.7165	S	0.0109	(0.63, 0.17)-(0.63, 0.16)	DataPointRange(5108 - 5108)	0.0109	0.01	0.78	0.78
 427	57.7384	F	0.0111	(0.59, 0.21)		DataPointRange(5109 - 5109)
 428	57.7384	S	0.0111	(0.59, 0.21)-(0.64, 0.16)	DataPointRange(5110 - 5110)	0.0111	0.06	5.09	5.09
 429	57.7605	F	0.0773	(0.64, 0.16)		DataPointRange(5111 - 5117)
 430	57.8269	S	0.0330	(0.65, 0.16)-(0.75, 0.17)	DataPointRange(5118 - 5120)	0.0330	0.11	3.25	5.36
 431	57.8709	F	0.1327	(0.79, 0.17)		DataPointRange(5121 - 5132)
 432	57.9924	S	0.0775	(0.79, 0.17)-(0.15, 0.26)	DataPointRange(5133 - 5139)	0.0775	0.64	8.32	12.87
 433	58.0810	F	0.2320	(0.15, 0.27)		DataPointRange(5140 - 5160)
 434	58.3018	S	0.0443	(0.15, 0.28)-(0.33, 0.25)	DataPointRange(5161 - 5164)	0.0443	0.18	3.99	7.26
 435	58.3570	F	0.2761	(0.34, 0.25)		DataPointRange(5165 - 5189)
 436	58.6221	S	0.0220	(0.35, 0.26)-(0.43, 0.23)	DataPointRange(5190 - 5191)	0.0220	0.08	3.84	6.41
 437	58.6552	F	0.0332	(0.45, 0.25)		DataPointRange(5192 - 5194)
 438	58.6773	S	0.0111	(0.46, 0.25)-(0.46, 0.25)	DataPointRange(5195 - 5195)	0.0111	0.01	0.69	0.69
 439	58.6994	F	0.0331	(0.43, 0.28)		DataPointRange(5196 - 5198)
 440	58.7215	S	0.0110	(0.41, 0.31)-(0.46, 0.24)	DataPointRange(5199 - 5199)	0.0110	0.07	6.20	6.20
 441	58.7439	F	0.1544	(0.46, 0.24)		DataPointRange(5200 - 5213)
 442	58.8872	S	0.0220	(0.46, 0.23)-(0.54, 0.23)	DataPointRange(5214 - 5215)	0.0220	0.09	3.96	7.12
 443	58.9204	F	0.2318	(0.56, 0.24)		DataPointRange(5216 - 5236)
 444	59.1415	S	0.0332	(0.55, 0.24)-(0.74, 0.25)	DataPointRange(5237 - 5239)	0.0332	0.19	5.75	7.23
 445	59.1853	F	0.3208	(0.77, 0.25)		DataPointRange(5240 - 5268)
 446	59.4949	S	0.0664	(0.78, 0.25)-(0.22, 0.30)	DataPointRange(5269 - 5274)	0.0664	0.56	8.48	11.67
 447	59.5724	F	0.1875	(0.20, 0.31)		DataPointRange(5275 - 5291)
 448	59.7488	S	0.0111	(0.21, 0.32)-(0.20, 0.31)	DataPointRange(5292 - 5292)	0.0111	0.02	1.63	1.63
 449	59.7709	F	0.1768	(0.17, 0.33)		DataPointRange(5293 - 5308)
 450	59.9367	S	0.0444	(0.18, 0.31)-(0.35, 0.31)	DataPointRange(5309 - 5312)	0.0444	0.18	3.97	7.16
 451	59.9921	F	0.2428	(0.37, 0.32)		DataPointRange(5313 - 5334)
 452	60.2239	S	0.0110	(0.37, 0.33)-(0.37, 0.33)	DataPointRange(5335 - 5335)	0.0110	0.01	0.56	0.56
 453	60.2458	F	0.1327	(0.43, 0.33)		DataPointRange(5336 - 5347)
 454	60.3673	S	0.0336	(0.43, 0.32)-(0.31, 0.34)	DataPointRange(5348 - 5350)	0.0336	0.12	3.64	5.35
 455	60.4116	F	0.2651	(0.30, 0.33)		DataPointRange(5351 - 5374)
 456	60.6660	S	0.0217	(0.30, 0.33)-(0.38, 0.33)	DataPointRange(5375 - 5376)	0.0217	0.08	3.53	5.48
 457	60.6987	F	0.1437	(0.41, 0.33)		DataPointRange(5377 - 5389)
 458	60.8313	S	0.0442	(0.40, 0.33)-(0.25, 0.20)	DataPointRange(5390 - 5393)	0.0442	0.18	4.13	7.34
 459	60.8867	F	0.5745	(0.22, 0.18)		DataPointRange(5394 - 5445)
 460	60.9419	End					DataPoint(5399)
 461	61.3293	t1n8s.bmp				DataPoint(5434)
 462	61.3837	Start					DataPoint(5439)
 463	61.4501	S	0.0111	(0.22, 0.20)-(0.21, 0.16)	DataPointRange(5446 - 5446)	0.0111	0.04	3.25	3.25
 464	61.4721	F	0.1327	(0.21, 0.15)		DataPointRange(5447 - 5458)
 465	61.5940	S	0.0331	(0.21, 0.14)-(0.30, 0.16)	DataPointRange(5459 - 5461)	0.0331	0.09	2.65	3.88
 466	61.6379	F	0.1988	(0.31, 0.16)		DataPointRange(5462 - 5479)
 467	61.8260	S	0.0217	(0.31, 0.16)-(0.36, 0.15)	DataPointRange(5480 - 5481)	0.0217	0.06	2.57	3.99
 468	61.8588	F	0.2651	(0.38, 0.17)		DataPointRange(5482 - 5505)
 469	62.1129	S	0.0442	(0.38, 0.15)-(0.57, 0.17)	DataPointRange(5506 - 5509)	0.0442	0.19	4.29	5.94
 470	62.1682	F	0.3201	(0.58, 0.17)		DataPointRange(5510 - 5538)
 471	62.4772	S	0.0332	(0.57, 0.19)-(0.65, 0.17)	DataPointRange(5539 - 5541)	0.0332	0.08	2.46	4.43
 472	62.5214	F	0.1992	(0.65, 0.18)		DataPointRange(5542 - 5559)
 473	62.7093	S	0.0221	(0.66, 0.17)-(0.73, 0.15)	DataPointRange(5560 - 5561)	0.0221	0.07	3.28	4.29
 474	62.7423	F	0.2871	(0.77, 0.17)		DataPointRange(5562 - 5587)
 475	63.0184	S	0.0665	(0.76, 0.16)-(0.20, 0.26)	DataPointRange(5588 - 5593)	0.0665	0.57	8.52	12.86
 476	63.0958	F	0.1438	(0.16, 0.27)		DataPointRange(5594 - 5606)
 477	63.2285	S	0.0111	(0.17, 0.28)-(0.15, 0.24)	DataPointRange(5607 - 5607)	0.0111	0.03	2.96	2.96
 478	63.2505	F	0.2875	(0.14, 0.25)		DataPointRange(5608 - 5633)
 479	63.5269	S	0.0331	(0.16, 0.26)-(0.31, 0.24)	DataPointRange(5634 - 5636)	0.0331	0.15	4.62	7.11
 480	63.5709	F	0.3093	(0.35, 0.25)		DataPointRange(5637 - 5664)
 481	63.8692	S	0.0219	(0.36, 0.25)-(0.41, 0.25)	DataPointRange(5665 - 5666)	0.0219	0.05	2.32	4.81
 482	63.9025	F	0.2427	(0.43, 0.24)		DataPointRange(5667 - 5688)
 483	64.1342	S	0.0330	(0.42, 0.24)-(0.57, 0.25)	DataPointRange(5689 - 5691)	0.0330	0.15	4.52	5.17
 484	64.1785	F	0.4640	(0.59, 0.25)		DataPointRange(5692 - 5733)
 485	64.6311	S	0.0114	(0.59, 0.23)-(0.60, 0.24)	DataPointRange(5734 - 5734)	0.0114	0.02	1.48	1.48
 486	64.6532	F	0.4863	(0.64, 0.24)		DataPointRange(5735 - 5778)
 487	65.1283	S	0.0331	(0.65, 0.24)-(0.75, 0.23)	DataPointRange(5779 - 5781)	0.0331	0.10	3.06	5.28
 488	65.2051	F	0.1109	(0.77, 0.23)		DataPointRange(5782 - 5792)
 489	65.3051	S	0.0773	(0.77, 0.21)-(0.20, 0.20)	DataPointRange(5793 - 5799)	0.0773	0.57	7.35	21.57
 490	65.3937	F	0.0108	(0.14, 0.20)		DataPointRange(5800 - 5800)
 491	65.3937	S	0.0108	(0.14, 0.20)-(0.17, 0.21)	DataPointRange(5801 - 5801)	0.0108	0.02	2.21	2.21
 492	65.4156	F	0.0115	(0.10, 0.18)		DataPointRange(5802 - 5802)
 493	65.4156	S	0.0221	(0.10, 0.18)-(0.17, 0.32)	DataPointRange(5803 - 5804)	0.0221	0.13	5.80	6.76
 494	65.4488	F	0.2652	(0.17, 0.34)		DataPointRange(5805 - 5828)
 495	65.7029	S	0.0221	(0.17, 0.34)-(0.26, 0.32)	DataPointRange(5829 - 5830)	0.0221	0.09	4.24	6.06
 496	65.7358	F	0.4088	(0.28, 0.33)		DataPointRange(5831 - 5867)
 497	66.1336	S	0.1876	(0.28, 0.33)-(0.49, 0.28)	DataPointRange(5868 - 5884)	0.1876	0.21	1.14	281.27
 498	66.3323	F	0.0111	(0.46, 0.24)		DataPointRange(5885 - 5885)
 499	66.3323	S	0.0331	(0.46, 0.24)-(0.50, 0.32)	DataPointRange(5886 - 5888)	0.0331	0.07	2.22	5.45
 500	66.3765	F	0.1880	(0.50, 0.34)		DataPointRange(5889 - 5905)
 501	66.5535	S	0.0220	(0.51, 0.32)-(0.56, 0.33)	DataPointRange(5906 - 5907)	0.0220	0.06	2.53	5.85
 502	66.5869	F	0.2869	(0.58, 0.34)		DataPointRange(5908 - 5933)
 503	66.8628	S	0.0218	(0.58, 0.34)-(0.63, 0.34)	DataPointRange(5934 - 5935)	0.0218	0.05	2.20	6.13
 504	66.8957	F	0.2650	(0.65, 0.34)		DataPointRange(5936 - 5959)
 505	67.1497	S	0.0553	(0.65, 0.32)-(0.33, 0.20)	DataPointRange(5960 - 5964)	0.0553	0.33	6.04	9.42
 506	67.2163	F	0.5079	(0.33, 0.19)		DataPointRange(5965 - 6010)
 507	67.3154	End					DataPoint(5974)
 508	67.7132	S	0.0110	(0.33, 0.20)-(0.30, 0.19)	DataPointRange(6011 - 6011)	0.0110	0.04	3.32	3.32
 509	67.7351	F	0.1107	(0.27, 0.18)		DataPointRange(6012 - 6021)
 510	67.7686	t1n4s.bmp				DataPoint(6015)
 511	67.8016	Start					DataPoint(6018)
 512	67.8348	S	0.0219	(0.27, 0.17)-(0.19, 0.15)	DataPointRange(6022 - 6023)	0.0219	0.09	3.92	5.90
 513	67.8677	F	0.2323	(0.17, 0.14)		DataPointRange(6024 - 6044)
 514	68.0887	S	0.0221	(0.16, 0.14)-(0.23, 0.13)	DataPointRange(6045 - 6046)	0.0221	0.07	3.11	5.02
 515	68.1220	F	0.3091	(0.26, 0.15)		DataPointRange(6047 - 6074)
 516	68.4201	S	0.0332	(0.26, 0.15)-(0.42, 0.17)	DataPointRange(6075 - 6077)	0.0332	0.16	4.86	6.84
 517	68.4643	F	0.2429	(0.46, 0.17)		DataPointRange(6078 - 6099)
 518	68.6962	S	0.0220	(0.47, 0.17)-(0.54, 0.16)	DataPointRange(6100 - 6101)	0.0220	0.08	3.51	4.79
 519	68.7295	F	0.2981	(0.57, 0.17)		DataPointRange(6102 - 6128)
 520	69.0166	S	0.0220	(0.58, 0.16)-(0.66, 0.19)	DataPointRange(6129 - 6130)	0.0220	0.08	3.75	5.90
 521	69.0498	F	0.1987	(0.66, 0.17)		DataPointRange(6131 - 6148)
 522	69.2376	S	0.0221	(0.66, 0.18)-(0.75, 0.17)	DataPointRange(6149 - 6150)	0.0221	0.09	4.07	5.95
 523	69.2708	F	0.2429	(0.76, 0.16)		DataPointRange(6151 - 6172)
 524	69.5026	S	0.0668	(0.76, 0.15)-(0.23, 0.22)	DataPointRange(6173 - 6178)	0.0668	0.53	8.01	13.35
 525	69.5800	F	0.3093	(0.19, 0.25)		DataPointRange(6179 - 6206)
 526	69.8781	S	0.0222	(0.19, 0.25)-(0.26, 0.23)	DataPointRange(6207 - 6208)	0.0222	0.07	3.15	5.34
 527	69.9113	F	0.2541	(0.29, 0.24)		DataPointRange(6209 - 6231)
 528	70.1548	S	0.0106	(0.31, 0.24)-(0.33, 0.23)	DataPointRange(6232 - 6232)	0.0106	0.02	1.90	1.90
 529	70.1766	F	0.1546	(0.38, 0.24)		DataPointRange(6233 - 6246)
 530	70.3202	S	0.0331	(0.37, 0.22)-(0.48, 0.25)	DataPointRange(6247 - 6249)	0.0331	0.11	3.31	5.65
 531	70.3645	F	0.2316	(0.53, 0.25)		DataPointRange(6250 - 6270)
 532	70.5853	S	0.0219	(0.53, 0.26)-(0.61, 0.24)	DataPointRange(6271 - 6272)	0.0219	0.09	4.03	6.84
 533	70.6182	F	0.2434	(0.65, 0.25)		DataPointRange(6273 - 6294)
 534	70.8502	S	0.0776	(0.66, 0.25)-(0.17, 0.32)	DataPointRange(6295 - 6301)	0.0776	0.48	6.25	11.31
 535	70.9388	F	0.2874	(0.19, 0.35)		DataPointRange(6302 - 6327)
 536	71.2148	S	0.0220	(0.19, 0.34)-(0.29, 0.33)	DataPointRange(6328 - 6329)	0.0220	0.10	4.72	7.79
 537	71.2478	F	0.3204	(0.28, 0.33)		DataPointRange(6330 - 6358)
 538	71.5576	S	0.0106	(0.27, 0.35)-(0.30, 0.35)	DataPointRange(6359 - 6359)	0.0106	0.04	3.41	3.41
 539	71.5793	F	0.2098	(0.34, 0.33)		DataPointRange(6360 - 6378)
 540	71.7781	S	0.0331	(0.34, 0.32)-(0.45, 0.34)	DataPointRange(6379 - 6381)	0.0331	0.11	3.22	6.46
 541	71.8224	F	0.2098	(0.47, 0.33)		DataPointRange(6382 - 6400)
 542	72.0211	S	0.0442	(0.49, 0.33)-(0.18, 0.43)	DataPointRange(6401 - 6404)	0.0442	0.32	7.20	10.88
 543	72.0763	F	0.2872	(0.18, 0.42)		DataPointRange(6405 - 6430)
 544	72.3526	S	0.0330	(0.18, 0.42)-(0.28, 0.44)	DataPointRange(6431 - 6433)	0.0330	0.10	3.07	5.16
 545	72.3966	F	0.2099	(0.29, 0.43)		DataPointRange(6434 - 6452)
 546	72.5954	S	0.0332	(0.29, 0.43)-(0.40, 0.39)	DataPointRange(6453 - 6455)	0.0332	0.11	3.42	4.60
 547	72.6398	F	0.2651	(0.40, 0.42)		DataPointRange(6456 - 6479)
 548	72.8938	S	0.0220	(0.42, 0.43)-(0.48, 0.42)	DataPointRange(6480 - 6481)	0.0220	0.06	2.67	6.25
 549	72.9269	F	0.5193	(0.50, 0.43)		DataPointRange(6482 - 6528)
 550	73.4352	S	0.0220	(0.51, 0.42)-(0.45, 0.44)	DataPointRange(6529 - 6530)	0.0220	0.06	2.68	5.05
 551	73.4685	F	0.1986	(0.45, 0.41)		DataPointRange(6531 - 6548)
 552	73.5347	End					DataPoint(6537)
 553	73.6561	S	0.0221	(0.45, 0.41)-(0.38, 0.32)	DataPointRange(6549 - 6550)	0.0221	0.10	4.68	6.97
 554	73.6893	F	0.1103	(0.39, 0.33)		DataPointRange(6551 - 6560)
 555	73.7885	S	0.0663	(0.39, 0.32)-(0.19, 0.17)	DataPointRange(6561 - 6566)	0.0663	0.23	3.41	6.92
 556	73.8658	F	0.2651	(0.18, 0.14)		DataPointRange(6567 - 6590)
 557	73.8768	t1n7s.bmp				DataPoint(6568)
 558	73.9652	Start					DataPoint(6576)
 559	74.1199	S	0.0223	(0.19, 0.13)-(0.24, 0.16)	DataPointRange(6591 - 6592)	0.0223	0.05	2.43	4.72
 560	74.1531	F	0.2098	(0.27, 0.15)		DataPointRange(6593 - 6611)
 561	74.3520	S	0.0334	(0.28, 0.17)-(0.36, 0.15)	DataPointRange(6612 - 6614)	0.0334	0.09	2.58	4.63
 562	74.3962	F	0.1439	(0.38, 0.15)		DataPointRange(6615 - 6627)
 563	74.5286	S	0.0223	(0.38, 0.15)-(0.46, 0.15)	DataPointRange(6628 - 6629)	0.0223	0.08	3.75	6.01
 564	74.5618	F	0.0886	(0.50, 0.16)		DataPointRange(6630 - 6637)
 565	74.6391	S	0.1330	(0.50, 0.19)-(0.55, 0.15)	DataPointRange(6638 - 6649)	0.1330	0.05	0.41	227.29
 566	74.7828	F	0.1767	(0.56, 0.17)		DataPointRange(6650 - 6665)
 567	74.9484	S	0.0332	(0.57, 0.17)-(0.70, 0.18)	DataPointRange(6666 - 6668)	0.0332	0.13	3.97	6.84
 568	74.9926	F	0.2540	(0.72, 0.17)		DataPointRange(6669 - 6691)
 569	75.2357	S	0.0221	(0.73, 0.17)-(0.80, 0.17)	DataPointRange(6692 - 6693)	0.0221	0.07	3.23	4.29
 570	75.2691	F	0.1653	(0.80, 0.17)		DataPointRange(6694 - 6708)
 571	75.4235	S	0.0777	(0.80, 0.16)-(0.21, 0.21)	DataPointRange(6709 - 6715)	0.0777	0.60	7.72	12.27
 572	75.5116	F	0.2983	(0.20, 0.25)		DataPointRange(6716 - 6742)
 573	75.7989	S	0.0221	(0.21, 0.26)-(0.28, 0.25)	DataPointRange(6743 - 6744)	0.0221	0.07	3.23	5.48
 574	75.8321	F	0.3203	(0.28, 0.24)		DataPointRange(6745 - 6773)
 575	76.1412	S	0.0333	(0.28, 0.25)-(0.43, 0.25)	DataPointRange(6774 - 6776)	0.0333	0.16	4.68	6.58
 576	76.1855	F	0.2984	(0.47, 0.25)		DataPointRange(6777 - 6803)
 577	76.4729	S	0.0222	(0.48, 0.26)-(0.55, 0.24)	DataPointRange(6804 - 6805)	0.0222	0.06	2.90	4.54
 578	76.5060	F	0.2983	(0.56, 0.25)		DataPointRange(6806 - 6832)
 579	76.7932	S	0.0111	(0.58, 0.27)-(0.60, 0.24)	DataPointRange(6833 - 6833)	0.0111	0.03	2.66	2.66
 580	76.8154	F	0.2760	(0.64, 0.24)		DataPointRange(6834 - 6858)
 581	77.0802	S	0.0331	(0.65, 0.23)-(0.75, 0.25)	DataPointRange(6859 - 6861)	0.0331	0.10	3.04	4.98
 582	77.1247	F	0.2869	(0.77, 0.24)		DataPointRange(6862 - 6887)
 583	77.4006	S	0.0663	(0.77, 0.25)-(0.26, 0.28)	DataPointRange(6888 - 6893)	0.0663	0.51	7.64	11.76
 584	77.4780	F	0.1546	(0.25, 0.31)		DataPointRange(6894 - 6907)
 585	77.6217	S	0.0219	(0.26, 0.30)-(0.21, 0.32)	DataPointRange(6908 - 6909)	0.0219	0.05	2.08	3.39
 586	77.6548	F	0.4420	(0.18, 0.35)		DataPointRange(6910 - 6949)
 587	78.0856	S	0.0441	(0.17, 0.34)-(0.37, 0.36)	DataPointRange(6950 - 6953)	0.0441	0.19	4.42	6.63
 588	78.1409	F	0.2981	(0.41, 0.34)		DataPointRange(6954 - 6980)
 589	78.4279	S	0.0224	(0.41, 0.35)-(0.46, 0.32)	DataPointRange(6981 - 6982)	0.0224	0.05	2.30	3.81
 590	78.4610	F	0.2652	(0.50, 0.33)		DataPointRange(6983 - 7006)
 591	78.7154	S	0.0334	(0.50, 0.33)-(0.63, 0.34)	DataPointRange(7007 - 7009)	0.0334	0.14	4.06	6.28
 592	78.7592	F	0.2322	(0.67, 0.33)		DataPointRange(7010 - 7030)
 593	78.9805	S	0.0220	(0.68, 0.32)-(0.74, 0.31)	DataPointRange(7031 - 7032)	0.0220	0.06	2.93	4.10
 594	79.0139	F	0.1433	(0.76, 0.32)		DataPointRange(7033 - 7045)
 595	79.1466	S	0.1165	(0.76, 0.32)-(0.21, 0.42)	DataPointRange(7046 - 7049)	0.1165	0.55	4.69	12.32
 596	79.2677	F	0.2979	(0.20, 0.42)		DataPointRange(7050 - 7076)
 597	79.5546	S	0.0224	(0.19, 0.42)-(0.27, 0.41)	DataPointRange(7077 - 7078)	0.0224	0.07	3.30	3.57
 598	79.5879	F	0.1879	(0.29, 0.41)		DataPointRange(7079 - 7095)
 599	79.7646	S	0.0330	(0.31, 0.39)-(0.45, 0.41)	DataPointRange(7096 - 7098)	0.0330	0.14	4.31	9.34
 600	79.8087	F	0.2540	(0.48, 0.41)		DataPointRange(7099 - 7121)
 601	80.0517	S	0.0331	(0.48, 0.40)-(0.63, 0.42)	DataPointRange(7122 - 7124)	0.0331	0.15	4.47	6.02
 602	80.0959	F	0.2982	(0.63, 0.41)		DataPointRange(7125 - 7151)
 603	80.3831	S	0.0221	(0.63, 0.41)-(0.71, 0.40)	DataPointRange(7152 - 7153)	0.0221	0.08	3.57	5.77
 604	80.4163	F	0.2428	(0.73, 0.41)		DataPointRange(7154 - 7175)
 605	80.6481	S	0.0222	(0.73, 0.42)-(0.79, 0.43)	DataPointRange(7176 - 7177)	0.0222	0.07	2.94	5.52
 606	80.6813	F	0.3868	(0.83, 0.42)		DataPointRange(7178 - 7212)
 607	81.0570	S	0.0111	(0.83, 0.40)-(0.83, 0.42)	DataPointRange(7213 - 7213)	0.0111	0.01	1.20	1.20
 608	81.0789	F	0.3313	(0.78, 0.42)		DataPointRange(7214 - 7243)
 609	81.3994	S	0.0441	(0.79, 0.40)-(0.50, 0.32)	DataPointRange(7244 - 7247)	0.0441	0.29	6.59	10.14
 610	81.4545	F	0.2209	(0.47, 0.33)		DataPointRange(7248 - 7267)
 611	81.4768	End					DataPoint(7250)
 612	81.6645	S	0.1992	(0.47, 0.34)-(0.19, 0.18)	DataPointRange(7268 - 7285)	0.1992	0.31	1.55	241.44
 613	81.8637	t1n10s.bmp				DataPoint(7285)
 614	81.8744	F	0.2211	(0.21, 0.23)		DataPointRange(7286 - 7305)
 615	81.9074	Start					DataPoint(7289)
 616	82.0844	S	0.0220	(0.22, 0.23)-(0.19, 0.17)	DataPointRange(7306 - 7307)	0.0220	0.06	2.60	4.60
 617	82.1175	F	0.7289	(0.21, 0.17)		DataPointRange(7308 - 7373)
 618	82.8352	S	0.0222	(0.22, 0.16)-(0.27, 0.17)	DataPointRange(7374 - 7375)	0.0222	0.05	2.11	4.90
 619	82.8684	F	0.1657	(0.27, 0.17)		DataPointRange(7376 - 7390)
 620	83.0233	S	0.0331	(0.26, 0.17)-(0.16, 0.18)	DataPointRange(7391 - 7393)	0.0331	0.11	3.22	10.11
 621	83.0672	F	0.0111	(0.06, 0.24)		DataPointRange(7394 - 7394)
 622	83.0672	S	0.0111	(0.06, 0.24)-(0.20, 0.17)	DataPointRange(7395 - 7395)	0.0111	0.15	13.38	13.38
 623	83.0897	F	0.0219	(0.21, 0.14)		DataPointRange(7396 - 7397)
 624	83.1005	S	0.0111	(0.21, 0.15)-(0.23, 0.11)	DataPointRange(7398 - 7398)	0.0111	0.03	2.91	2.91
 625	83.1227	F	0.0108	(0.22, 0.08)		DataPointRange(7399 - 7399)
 626	83.1227	S	0.0108	(0.22, 0.08)-(0.25, 0.16)	DataPointRange(7400 - 7400)	0.0108	0.06	5.68	5.68
 627	83.1449	F	0.3314	(0.24, 0.17)		DataPointRange(7401 - 7430)
 628	83.4652	S	0.0111	(0.24, 0.16)-(0.29, 0.16)	DataPointRange(7431 - 7431)	0.0111	0.05	4.49	4.49
 629	83.4872	F	0.2209	(0.33, 0.16)		DataPointRange(7432 - 7451)
 630	83.6971	S	0.0332	(0.32, 0.16)-(0.45, 0.17)	DataPointRange(7452 - 7454)	0.0332	0.14	4.16	7.17
 631	83.7414	F	0.2429	(0.48, 0.16)		DataPointRange(7455 - 7476)
 632	83.9730	S	0.0444	(0.48, 0.17)-(0.68, 0.15)	DataPointRange(7477 - 7480)	0.0444	0.20	4.41	7.39
 633	84.0282	F	0.2762	(0.70, 0.16)		DataPointRange(7481 - 7505)
 634	84.2934	S	0.0223	(0.71, 0.16)-(0.78, 0.17)	DataPointRange(7506 - 7507)	0.0223	0.07	2.98	6.22
 635	84.3265	F	0.1989	(0.79, 0.16)		DataPointRange(7508 - 7525)
 636	84.5143	S	0.0220	(0.78, 0.15)-(0.83, 0.15)	DataPointRange(7526 - 7527)	0.0220	0.05	2.30	4.14
 637	84.5477	F	0.1327	(0.83, 0.14)		DataPointRange(7528 - 7539)
 638	84.6691	S	0.0773	(0.82, 0.14)-(0.24, 0.23)	DataPointRange(7540 - 7546)	0.0773	0.59	7.59	11.94
 639	84.7575	F	0.1435	(0.23, 0.22)		DataPointRange(7547 - 7559)
 640	84.8900	S	0.0110	(0.22, 0.21)-(0.20, 0.22)	DataPointRange(7560 - 7560)	0.0110	0.03	2.56	2.56
 641	84.9122	F	0.2318	(0.19, 0.24)		DataPointRange(7561 - 7581)
 642	85.1329	S	0.0331	(0.19, 0.24)-(0.34, 0.22)	DataPointRange(7582 - 7584)	0.0331	0.15	4.68	7.26
 643	85.1898	F	0.2524	(0.35, 0.24)		DataPointRange(7585 - 7607)
 644	85.4312	S	0.0220	(0.36, 0.26)-(0.43, 0.25)	DataPointRange(7608 - 7609)	0.0220	0.07	3.14	4.71
 645	85.4643	F	0.2321	(0.42, 0.24)		DataPointRange(7610 - 7630)
 646	85.6852	S	0.0335	(0.42, 0.25)-(0.53, 0.28)	DataPointRange(7631 - 7633)	0.0335	0.12	3.48	6.30
 647	85.7293	F	0.2100	(0.56, 0.24)		DataPointRange(7634 - 7652)
 648	85.9284	S	0.0330	(0.56, 0.25)-(0.69, 0.25)	DataPointRange(7653 - 7655)	0.0330	0.13	3.80	5.43
 649	85.9724	F	0.2542	(0.69, 0.23)		DataPointRange(7656 - 7678)
 650	86.2164	S	0.0212	(0.70, 0.24)-(0.74, 0.25)	DataPointRange(7679 - 7680)	0.0212	0.05	2.25	5.53
 651	86.2486	F	0.1993	(0.77, 0.23)		DataPointRange(7681 - 7698)
 652	86.4365	S	0.0778	(0.77, 0.23)-(0.19, 0.30)	DataPointRange(7699 - 7705)	0.0778	0.59	7.52	21.01
 653	86.5249	F	0.3425	(0.20, 0.33)		DataPointRange(7706 - 7736)
 654	86.8563	S	0.0221	(0.19, 0.32)-(0.24, 0.32)	DataPointRange(7737 - 7738)	0.0221	0.05	2.34	4.17
 655	86.8894	F	0.1877	(0.28, 0.34)		DataPointRange(7739 - 7755)
 656	87.0660	S	0.0662	(0.27, 0.30)-(0.36, -0.11)	DataPointRange(7756 - 7761)	0.0662	0.32	4.80	137.18
 657	87.1437	F	0.0107	(0.35, -0.12)		DataPointRange(7762 - 7762)
 658	87.1437	S	0.1433	(0.35, -0.12)-(0.25, 0.33)	DataPointRange(7763 - 7775)	0.1433	0.35	2.46	183.83
 659	87.2982	F	0.5188	(0.28, 0.34)		DataPointRange(7776 - 7822)
 660	87.8063	S	0.0439	(0.29, 0.33)-(0.48, 0.30)	DataPointRange(7823 - 7826)	0.0439	0.20	4.52	6.92
 661	87.8613	F	0.1548	(0.49, 0.28)		DataPointRange(7827 - 7840)
 662	88.0051	S	0.0334	(0.48, 0.28)-(0.61, 0.27)	DataPointRange(7841 - 7843)	0.0334	0.13	3.91	5.46
 663	88.0491	F	0.2099	(0.63, 0.26)		DataPointRange(7844 - 7862)
 664	88.2480	S	0.0332	(0.64, 0.26)-(0.48, 0.24)	DataPointRange(7863 - 7865)	0.0332	0.15	4.60	7.41
 665	88.2922	F	0.2099	(0.47, 0.24)		DataPointRange(7866 - 7884)
 666	88.4910	S	0.0332	(0.49, 0.25)-(0.35, 0.21)	DataPointRange(7885 - 7887)	0.0332	0.14	4.28	7.02
 667	88.5355	F	0.3531	(0.34, 0.20)		DataPointRange(7888 - 7919)
 668	88.8776	S	0.0221	(0.33, 0.20)-(0.26, 0.29)	DataPointRange(7920 - 7921)	0.0221	0.10	4.35	6.23
 669	88.9106	F	0.2099	(0.27, 0.33)		DataPointRange(7922 - 7940)
 670	89.1095	S	0.0663	(0.27, 0.35)-(1.86, -0.32)	DataPointRange(7941 - 7946)	0.0663	1.67	25.20	169.33
 671	89.1868	F	0.0221	(1.86, -0.32)		DataPointRange(7947 - 7948)
 672	89.1982	S	0.1543	(1.86, -0.32)-(0.21, 0.34)	DataPointRange(7949 - 7962)	0.1543	1.72	11.17	211.90
 673	89.3637	F	0.0663	(0.24, 0.30)		DataPointRange(7963 - 7968)
 674	89.4188	S	0.0112	(0.24, 0.26)-(0.25, 0.32)	DataPointRange(7969 - 7969)	0.0112	0.05	4.27	4.27
 675	89.4410	F	0.0772	(0.25, 0.33)		DataPointRange(7970 - 7976)
 676	89.5072	S	0.0332	(0.26, 0.33)-(0.21, 0.20)	DataPointRange(7977 - 7979)	0.0332	0.11	3.44	4.81
 677	89.5515	F	0.9721	(0.22, 0.17)		DataPointRange(7980 - 8067)
 678	89.8385	End					DataPoint(8006)
 679	90.2363	aa1n19.bmp				DataPoint(8042)
 680	90.3136	Start					DataPoint(8049)
 681	90.5124	S	0.0221	(0.20, 0.13)-(0.28, 0.16)	DataPointRange(8068 - 8069)	0.0221	0.08	3.64	4.86
 682	90.5455	F	0.1878	(0.29, 0.16)		DataPointRange(8070 - 8086)
 683	90.7227	S	0.0217	(0.29, 0.16)-(0.36, 0.16)	DataPointRange(8087 - 8088)	0.0217	0.07	3.20	5.20
 684	90.7554	F	0.1989	(0.38, 0.15)		DataPointRange(8089 - 8106)
 685	90.9431	S	0.0223	(0.38, 0.16)-(0.46, 0.15)	DataPointRange(8107 - 8108)	0.0223	0.08	3.76	5.60
 686	90.9763	F	0.1879	(0.49, 0.15)		DataPointRange(8109 - 8125)
 687	91.1533	S	0.0329	(0.50, 0.15)-(0.62, 0.17)	DataPointRange(8126 - 8128)	0.0329	0.13	3.80	7.20
 688	91.1975	F	0.1876	(0.64, 0.16)		DataPointRange(8129 - 8145)
 689	91.3741	S	0.0219	(0.64, 0.16)-(0.71, 0.15)	DataPointRange(8146 - 8147)	0.0219	0.07	3.15	4.45
 690	91.4072	F	0.2540	(0.75, 0.15)		DataPointRange(8148 - 8170)
 691	91.6501	S	0.0111	(0.75, 0.16)-(0.77, 0.17)	DataPointRange(8171 - 8171)	0.0111	0.03	2.39	2.39
 692	91.6722	F	0.0663	(0.80, 0.16)		DataPointRange(8172 - 8177)
 693	91.7275	S	0.0772	(0.80, 0.16)-(0.21, 0.22)	DataPointRange(8178 - 8184)	0.0772	0.59	7.60	11.02
 694	91.8163	F	0.3090	(0.21, 0.23)		DataPointRange(8185 - 8212)
 695	92.1143	S	0.0221	(0.21, 0.22)-(0.29, 0.24)	DataPointRange(8213 - 8214)	0.0221	0.08	3.71	6.49
 696	92.1476	F	0.2427	(0.31, 0.23)		DataPointRange(8215 - 8236)
 697	92.3797	S	0.0329	(0.30, 0.23)-(0.46, 0.22)	DataPointRange(8237 - 8239)	0.0329	0.16	4.87	8.98
 698	92.4235	F	0.4751	(0.48, 0.25)		DataPointRange(8240 - 8282)
 699	92.8875	S	0.0220	(0.48, 0.25)-(0.55, 0.24)	DataPointRange(8283 - 8284)	0.0220	0.07	3.24	4.48
 700	92.9207	F	0.2539	(0.56, 0.23)		DataPointRange(8285 - 8307)
 701	93.1635	S	0.0111	(0.55, 0.23)-(0.57, 0.24)	DataPointRange(8308 - 8308)	0.0111	0.02	2.24	2.24
 702	93.1857	F	0.3316	(0.62, 0.24)		DataPointRange(8309 - 8338)
 703	93.5062	S	0.0221	(0.62, 0.24)-(0.68, 0.25)	DataPointRange(8339 - 8340)	0.0221	0.06	2.70	3.57
 704	93.5391	F	0.1548	(0.68, 0.23)		DataPointRange(8341 - 8354)
 705	93.6828	S	0.0225	(0.68, 0.23)-(0.76, 0.24)	DataPointRange(8355 - 8356)	0.0225	0.08	3.63	4.65
 706	93.7159	F	0.2650	(0.79, 0.23)		DataPointRange(8357 - 8380)
 707	93.9700	S	0.0772	(0.79, 0.23)-(0.18, 0.30)	DataPointRange(8381 - 8387)	0.0772	0.61	7.93	11.29
 708	94.0583	F	0.2654	(0.18, 0.31)		DataPointRange(8388 - 8411)
 709	94.3125	S	0.0993	(0.18, 0.34)-(0.20, 0.33)	DataPointRange(8412 - 8420)	0.0993	0.02	0.22	10.55
 710	94.4229	F	0.4754	(0.20, 0.34)		DataPointRange(8421 - 8463)
 711	94.8871	S	0.0328	(0.21, 0.31)-(0.33, 0.34)	DataPointRange(8464 - 8466)	0.0328	0.12	3.70	6.23
 712	94.9311	F	0.3535	(0.37, 0.34)		DataPointRange(8467 - 8498)
 713	95.2736	S	0.0442	(0.38, 0.34)-(0.59, 0.36)	DataPointRange(8499 - 8502)	0.0442	0.20	4.58	7.80
 714	95.3291	F	0.2428	(0.60, 0.35)		DataPointRange(8503 - 8524)
 715	95.5607	S	0.0222	(0.59, 0.35)-(0.67, 0.36)	DataPointRange(8525 - 8526)	0.0222	0.08	3.62	5.52
 716	95.5943	F	0.3200	(0.69, 0.34)		DataPointRange(8527 - 8555)
 717	95.9032	S	0.0442	(0.70, 0.34)-(0.38, 0.38)	DataPointRange(8556 - 8559)	0.0442	0.31	7.10	9.87
 718	95.9587	F	0.1100	(0.40, 0.37)		DataPointRange(8560 - 8569)
 719	96.0577	S	0.0440	(0.42, 0.37)-(0.19, 0.42)	DataPointRange(8570 - 8573)	0.0440	0.23	5.12	8.30
 720	96.1128	F	0.2654	(0.19, 0.43)		DataPointRange(8574 - 8597)
 721	96.3671	S	0.0223	(0.20, 0.41)-(0.27, 0.43)	DataPointRange(8598 - 8599)	0.0223	0.07	3.18	5.50
 722	96.4002	F	0.2545	(0.27, 0.42)		DataPointRange(8600 - 8622)
 723	96.6434	S	0.0440	(0.27, 0.43)-(0.49, 0.42)	DataPointRange(8623 - 8626)	0.0440	0.21	4.84	7.60
 724	96.6987	F	0.2648	(0.51, 0.42)		DataPointRange(8627 - 8650)
 725	96.9525	S	0.0110	(0.51, 0.41)-(0.55, 0.43)	DataPointRange(8651 - 8651)	0.0110	0.04	3.72	3.72
 726	96.9747	F	0.2206	(0.59, 0.42)		DataPointRange(8652 - 8671)
 727	97.1843	S	0.0220	(0.57, 0.41)-(0.65, 0.41)	DataPointRange(8672 - 8673)	0.0220	0.08	3.61	4.59
 728	97.2174	F	0.1438	(0.68, 0.41)		DataPointRange(8674 - 8686)
 729	97.3505	S	0.0771	(0.67, 0.43)-(0.13, 0.42)	DataPointRange(8687 - 8693)	0.0771	0.55	7.10	12.36
 730	97.4385	F	0.0114	(0.08, 0.40)		DataPointRange(8694 - 8694)
 731	97.4385	S	0.0448	(0.08, 0.40)-(0.15, 0.57)	DataPointRange(8695 - 8698)	0.0448	0.14	3.20	7.01
 732	97.4939	F	0.1879	(0.15, 0.55)		DataPointRange(8699 - 8715)
 733	97.6706	S	0.0222	(0.15, 0.54)-(0.24, 0.54)	DataPointRange(8716 - 8717)	0.0222	0.09	3.95	4.97
 734	97.7037	F	0.1658	(0.26, 0.53)		DataPointRange(8718 - 8732)
 735	97.8581	S	0.0223	(0.26, 0.52)-(0.36, 0.52)	DataPointRange(8733 - 8734)	0.0223	0.11	4.78	6.49
 736	97.8912	F	0.2542	(0.41, 0.53)		DataPointRange(8735 - 8757)
 737	98.1345	S	0.0442	(0.42, 0.52)-(0.63, 0.51)	DataPointRange(8758 - 8761)	0.0442	0.22	4.87	7.58
 738	98.1898	F	0.2542	(0.62, 0.52)		DataPointRange(8762 - 8784)
 739	98.4327	S	0.0113	(0.62, 0.51)-(0.61, 0.52)	DataPointRange(8785 - 8785)	0.0113	0.01	1.17	1.17
 740	98.4547	F	0.3426	(0.58, 0.51)		DataPointRange(8786 - 8816)
 741	98.7862	S	0.0219	(0.58, 0.51)-(0.66, 0.52)	DataPointRange(8817 - 8818)	0.0219	0.08	3.54	3.87
 742	98.8193	F	0.3534	(0.68, 0.52)		DataPointRange(8819 - 8850)
 743	99.1616	S	0.0331	(0.68, 0.51)-(0.55, 0.48)	DataPointRange(8851 - 8853)	0.0331	0.13	3.89	7.16
 744	99.2058	F	0.0222	(0.55, 0.48)		DataPointRange(8854 - 8855)
 745	99.2169	S	0.2322	(0.55, 0.47)-(0.44, 0.41)	DataPointRange(8856 - 8876)	0.2322	0.12	0.53	171.12
 746	99.4601	F	0.0444	(0.42, 0.38)		DataPointRange(8877 - 8880)
 747	99.4931	S	0.0114	(0.41, 0.36)-(0.44, 0.42)	DataPointRange(8881 - 8881)	0.0114	0.05	4.37	4.37
 748	99.5153	F	0.2433	(0.45, 0.43)		DataPointRange(8882 - 8903)
 749	99.7472	S	0.0331	(0.44, 0.45)-(0.37, 0.36)	DataPointRange(8904 - 8906)	0.0331	0.09	2.82	6.16
 750	99.7913	F	0.2873	(0.36, 0.32)		DataPointRange(8907 - 8932)
 751	100.0674	S	0.0335	(0.37, 0.32)-(0.30, 0.22)	DataPointRange(8933 - 8935)	0.0335	0.10	2.98	6.01
 752	100.1116	F	0.5743	(0.31, 0.19)		DataPointRange(8936 - 8987)
 753	100.6749	S	0.0223	(0.31, 0.18)-(0.23, 0.20)	DataPointRange(8988 - 8989)	0.0223	0.07	3.27	4.49
 754	100.7080	F	0.3095	(0.22, 0.20)		DataPointRange(8990 - 9017)
 755	101.0064	S	0.0111	(0.22, 0.21)-(0.20, 0.16)	DataPointRange(9018 - 9018)	0.0111	0.05	4.36	4.36
 756	101.0289	F	0.6625	(0.21, 0.16)		DataPointRange(9019 - 9078)
 757	101.0949	End					DataPoint(9025)
 758	101.5366	aa1n10.bmp				DataPoint(9065)
 759	101.6359	Start					DataPoint(9074)
 760	101.6803	S	0.0220	(0.20, 0.17)-(0.26, 0.14)	DataPointRange(9079 - 9080)	0.0220	0.06	2.69	3.41
 761	101.7134	F	0.1878	(0.28, 0.17)		DataPointRange(9081 - 9097)
 762	101.8905	S	0.0107	(0.28, 0.16)-(0.31, 0.16)	DataPointRange(9098 - 9098)	0.0107	0.03	3.03	3.03
 763	101.9123	F	0.2761	(0.35, 0.17)		DataPointRange(9099 - 9123)
 764	102.1776	S	0.0108	(0.35, 0.18)-(0.40, 0.16)	DataPointRange(9124 - 9124)	0.0108	0.05	4.77	4.77
 765	102.1994	F	0.1438	(0.41, 0.17)		DataPointRange(9125 - 9137)
 766	102.3322	S	0.0331	(0.41, 0.19)-(0.53, 0.17)	DataPointRange(9138 - 9140)	0.0331	0.12	3.64	6.74
 767	102.3763	F	0.1323	(0.55, 0.19)		DataPointRange(9141 - 9152)
 768	102.4976	S	0.0556	(0.55, 0.18)-(0.18, 0.25)	DataPointRange(9153 - 9157)	0.0556	0.37	6.70	12.95
 769	102.5639	F	0.2649	(0.15, 0.26)		DataPointRange(9158 - 9181)
 770	102.8180	S	0.0220	(0.14, 0.24)-(0.20, 0.28)	DataPointRange(9182 - 9183)	0.0220	0.07	3.02	4.80
 771	102.8510	F	0.1993	(0.24, 0.25)		DataPointRange(9184 - 9201)
 772	103.0388	S	0.0222	(0.26, 0.26)-(0.33, 0.25)	DataPointRange(9202 - 9203)	0.0222	0.07	3.36	6.87
 773	103.0720	F	0.1988	(0.35, 0.25)		DataPointRange(9204 - 9221)
 774	103.2602	S	0.0326	(0.36, 0.27)-(0.48, 0.26)	DataPointRange(9222 - 9224)	0.0326	0.12	3.79	6.38
 775	103.3038	F	0.2214	(0.51, 0.25)		DataPointRange(9225 - 9244)
 776	103.5142	S	0.0330	(0.51, 0.26)-(0.64, 0.24)	DataPointRange(9245 - 9247)	0.0330	0.13	4.01	6.13
 777	103.5582	F	0.2649	(0.67, 0.24)		DataPointRange(9248 - 9271)
 778	103.8122	S	0.0219	(0.68, 0.24)-(0.76, 0.24)	DataPointRange(9272 - 9273)	0.0219	0.08	3.47	3.77
 779	103.8451	F	0.1879	(0.78, 0.24)		DataPointRange(9274 - 9290)
 780	104.0219	S	0.1218	(0.77, 0.24)-(0.19, 0.33)	DataPointRange(9291 - 9301)	0.1218	0.58	4.78	18.87
 781	104.1545	F	0.2651	(0.20, 0.33)		DataPointRange(9302 - 9325)
 782	104.4086	S	0.0330	(0.20, 0.34)-(0.32, 0.33)	DataPointRange(9326 - 9328)	0.0330	0.13	3.90	6.12
 783	104.4527	F	0.3646	(0.37, 0.33)		DataPointRange(9329 - 9361)
 784	104.8063	S	0.0333	(0.37, 0.33)-(0.53, 0.33)	DataPointRange(9362 - 9364)	0.0333	0.16	4.92	6.07
 785	104.8504	F	0.3203	(0.54, 0.34)		DataPointRange(9365 - 9393)
 786	105.1596	S	0.0221	(0.54, 0.35)-(0.60, 0.33)	DataPointRange(9394 - 9395)	0.0221	0.06	2.72	3.73
 787	105.1929	F	0.2100	(0.61, 0.34)		DataPointRange(9396 - 9414)
 788	105.3916	S	0.0552	(0.61, 0.32)-(0.21, 0.40)	DataPointRange(9415 - 9419)	0.0552	0.40	7.27	10.24
 789	105.4579	F	0.2542	(0.20, 0.42)		DataPointRange(9420 - 9442)
 790	105.7011	S	0.0220	(0.19, 0.42)-(0.27, 0.41)	DataPointRange(9443 - 9444)	0.0220	0.08	3.80	6.05
 791	105.7345	F	0.3200	(0.29, 0.42)		DataPointRange(9445 - 9473)
 792	106.0434	S	0.0330	(0.30, 0.42)-(0.43, 0.42)	DataPointRange(9474 - 9476)	0.0330	0.13	4.00	7.81
 793	106.0875	F	0.1547	(0.45, 0.43)		DataPointRange(9477 - 9490)
 794	106.2311	S	0.0221	(0.44, 0.43)-(0.50, 0.41)	DataPointRange(9491 - 9492)	0.0221	0.06	2.50	5.12
 795	106.2642	F	0.1992	(0.53, 0.42)		DataPointRange(9493 - 9510)
 796	106.4522	S	0.0330	(0.54, 0.42)-(0.65, 0.43)	DataPointRange(9511 - 9513)	0.0330	0.12	3.52	6.12
 797	106.4966	F	0.3089	(0.68, 0.42)		DataPointRange(9514 - 9541)
 798	106.7945	S	0.0665	(0.67, 0.41)-(0.18, 0.50)	DataPointRange(9542 - 9547)	0.0665	0.49	7.44	11.62
 799	106.8718	F	0.5303	(0.20, 0.51)		DataPointRange(9548 - 9595)
 800	107.3910	S	0.0442	(0.21, 0.53)-(0.45, 0.55)	DataPointRange(9596 - 9599)	0.0442	0.25	5.59	9.44
 801	107.4464	F	0.2098	(0.46, 0.52)		DataPointRange(9600 - 9618)
 802	107.6450	S	0.1109	(0.45, 0.55)-(1.87, -0.26)	DataPointRange(9619 - 9628)	0.1109	1.54	13.92	190.11
 803	107.7665	F	0.0332	(1.87, -0.26)		DataPointRange(9629 - 9631)
 804	107.7889	S	0.0994	(1.87, -0.25)-(0.60, 0.47)	DataPointRange(9632 - 9640)	0.0994	1.38	13.89	130.24
 805	107.8991	F	0.1878	(0.59, 0.49)		DataPointRange(9641 - 9657)
 806	108.0759	S	0.0221	(0.60, 0.49)-(0.69, 0.53)	DataPointRange(9658 - 9659)	0.0221	0.09	4.20	5.23
 807	108.1090	F	0.2209	(0.72, 0.50)		DataPointRange(9660 - 9679)
 808	108.3189	S	0.0664	(0.71, 0.50)-(0.16, 0.64)	DataPointRange(9680 - 9685)	0.0664	0.56	8.44	11.59
 809	108.3962	F	0.4198	(0.15, 0.65)		DataPointRange(9686 - 9723)
 810	108.8050	S	0.0776	(0.13, 0.64)-(0.74, 0.53)	DataPointRange(9724 - 9730)	0.0776	0.62	8.00	13.22
 811	108.8932	F	0.3977	(0.74, 0.52)		DataPointRange(9731 - 9766)
 812	109.2800	S	0.0331	(0.74, 0.53)-(0.56, 0.47)	DataPointRange(9767 - 9769)	0.0331	0.18	5.45	9.20
 813	109.3240	F	0.1989	(0.52, 0.45)		DataPointRange(9770 - 9787)
 814	109.5118	S	0.0333	(0.52, 0.45)-(0.41, 0.41)	DataPointRange(9788 - 9790)	0.0333	0.11	3.29	5.96
 815	109.5562	F	0.3643	(0.40, 0.39)		DataPointRange(9791 - 9823)
 816	109.9097	S	0.0329	(0.41, 0.39)-(0.53, 0.39)	DataPointRange(9824 - 9826)	0.0329	0.13	3.82	6.26
 817	109.9537	F	0.1988	(0.54, 0.41)		DataPointRange(9827 - 9844)
 818	110.1415	S	0.1215	(0.55, 0.42)-(1.86, -0.32)	DataPointRange(9845 - 9855)	0.1215	1.43	11.76	168.99
 819	110.2742	F	0.0108	(1.86, -0.32)		DataPointRange(9856 - 9856)
 820	110.2742	S	0.0993	(1.86, -0.32)-(0.44, 0.36)	DataPointRange(9857 - 9865)	0.0993	1.51	15.19	238.50
 821	110.3845	F	0.0442	(0.44, 0.30)		DataPointRange(9866 - 9869)
 822	110.4176	S	0.0111	(0.44, 0.27)-(0.44, 0.34)	DataPointRange(9870 - 9870)	0.0111	0.05	4.43	4.43
 823	110.4400	F	0.2206	(0.45, 0.34)		DataPointRange(9871 - 9890)
 824	110.6497	S	0.0330	(0.45, 0.35)-(0.34, 0.28)	DataPointRange(9891 - 9893)	0.0330	0.13	3.81	6.12
 825	110.6939	F	0.3975	(0.34, 0.28)		DataPointRange(9894 - 9929)
 826	111.0805	S	0.0222	(0.34, 0.30)-(0.27, 0.26)	DataPointRange(9930 - 9931)	0.0222	0.08	3.48	3.87
 827	111.1135	F	0.3207	(0.23, 0.24)		DataPointRange(9932 - 9960)
 828	111.4229	S	0.0113	(0.24, 0.22)-(0.22, 0.20)	DataPointRange(9961 - 9961)	0.0113	0.02	2.06	2.06
 829	111.4449	F	0.6298	(0.23, 0.17)		DataPointRange(9962 - 10018)
 830	111.4892	End					DataPoint(9966)
 831	111.8427	aa1n14.bmp				DataPoint(9998)
 832	111.9531	Start					DataPoint(10008)
 833	112.0636	S	0.0111	(0.23, 0.17)-(0.27, 0.16)	DataPointRange(10019 - 10019)	0.0111	0.04	3.61	3.61
 834	112.0858	F	0.2099	(0.27, 0.17)		DataPointRange(10020 - 10038)
 835	112.2846	S	0.0219	(0.26, 0.17)-(0.33, 0.16)	DataPointRange(10039 - 10040)	0.0219	0.07	2.99	3.88
 836	112.3178	F	0.0992	(0.36, 0.16)		DataPointRange(10041 - 10049)
 837	112.4060	S	0.0773	(0.37, 0.16)-(1.86, -0.32)	DataPointRange(10050 - 10056)	0.0773	1.54	19.93	172.02
 838	112.4946	F	0.0217	(1.86, -0.32)		DataPointRange(10057 - 10058)
 839	112.5054	S	0.0554	(1.86, -0.32)-(0.51, 0.15)	DataPointRange(10059 - 10063)	0.0554	1.40	25.33	129.46
 840	112.5718	F	0.2540	(0.53, 0.17)		DataPointRange(10064 - 10086)
 841	112.8147	S	0.0220	(0.52, 0.17)-(0.59, 0.17)	DataPointRange(10087 - 10088)	0.0220	0.07	3.09	4.75
 842	112.8478	F	0.2100	(0.61, 0.17)		DataPointRange(10089 - 10107)
 843	113.0467	S	0.0222	(0.61, 0.17)-(0.71, 0.17)	DataPointRange(10108 - 10109)	0.0222	0.09	4.18	4.97
 844	113.0799	F	0.2433	(0.75, 0.17)		DataPointRange(10110 - 10131)
 845	113.3122	S	0.0220	(0.74, 0.16)-(0.82, 0.17)	DataPointRange(10132 - 10133)	0.0220	0.08	3.81	4.01
 846	113.3453	F	0.2868	(0.83, 0.15)		DataPointRange(10134 - 10159)
 847	113.6210	S	0.0672	(0.82, 0.15)-(0.26, 0.20)	DataPointRange(10160 - 10165)	0.0672	0.57	8.44	14.66
 848	113.6983	F	0.1438	(0.23, 0.21)		DataPointRange(10166 - 10178)
 849	113.8308	S	0.0226	(0.23, 0.22)-(0.14, 0.25)	DataPointRange(10179 - 10180)	0.0226	0.10	4.35	6.92
 850	113.8643	F	0.2212	(0.14, 0.25)		DataPointRange(10181 - 10200)
 851	114.0742	S	0.0219	(0.16, 0.24)-(0.27, 0.24)	DataPointRange(10201 - 10202)	0.0219	0.11	5.03	8.49
 852	114.1074	F	0.2205	(0.29, 0.24)		DataPointRange(10203 - 10222)
 853	114.3170	S	0.0219	(0.29, 0.23)-(0.38, 0.24)	DataPointRange(10223 - 10224)	0.0219	0.09	3.99	5.52
 854	114.3501	F	0.1877	(0.38, 0.24)		DataPointRange(10225 - 10241)
 855	114.5268	S	0.0441	(0.39, 0.24)-(0.56, 0.26)	DataPointRange(10242 - 10245)	0.0441	0.18	3.97	6.52
 856	114.5823	F	0.1545	(0.59, 0.25)		DataPointRange(10246 - 10259)
 857	114.7257	S	0.0223	(0.58, 0.26)-(0.51, 0.25)	DataPointRange(10260 - 10261)	0.0223	0.08	3.52	7.01
 858	114.7589	F	0.1218	(0.48, 0.25)		DataPointRange(10262 - 10272)
 859	114.8693	S	0.0222	(0.48, 0.26)-(0.55, 0.25)	DataPointRange(10273 - 10274)	0.0222	0.06	2.84	5.41
 860	114.9025	F	0.2098	(0.58, 0.24)		DataPointRange(10275 - 10293)
 861	115.1015	S	0.0330	(0.57, 0.24)-(0.71, 0.24)	DataPointRange(10294 - 10296)	0.0330	0.14	4.13	5.83
 862	115.1458	F	0.1325	(0.74, 0.25)		DataPointRange(10297 - 10308)
 863	115.2672	S	0.0663	(0.74, 0.24)-(0.19, 0.33)	DataPointRange(10309 - 10314)	0.0663	0.55	8.34	12.14
 864	115.3456	F	0.2860	(0.20, 0.34)		DataPointRange(10315 - 10340)
 865	115.6205	S	0.0333	(0.19, 0.35)-(0.29, 0.32)	DataPointRange(10341 - 10343)	0.0333	0.10	3.07	4.25
 866	115.6647	F	0.3203	(0.31, 0.33)		DataPointRange(10344 - 10372)
 867	115.9741	S	0.0330	(0.31, 0.33)-(0.49, 0.34)	DataPointRange(10373 - 10375)	0.0330	0.18	5.41	8.67
 868	116.0181	F	0.2873	(0.51, 0.34)		DataPointRange(10376 - 10401)
 869	116.2943	S	0.0222	(0.52, 0.35)-(0.58, 0.32)	DataPointRange(10402 - 10403)	0.0222	0.06	2.78	5.54
 870	116.3275	F	0.1876	(0.60, 0.33)		DataPointRange(10404 - 10420)
 871	116.5040	S	0.0553	(0.60, 0.34)-(0.19, 0.39)	DataPointRange(10421 - 10425)	0.0553	0.41	7.47	11.64
 872	116.5706	F	0.2871	(0.18, 0.44)		DataPointRange(10426 - 10451)
 873	116.8465	S	0.0441	(0.18, 0.45)-(0.37, 0.42)	DataPointRange(10452 - 10455)	0.0441	0.19	4.27	6.06
 874	116.9020	F	0.2428	(0.38, 0.42)		DataPointRange(10456 - 10477)
 875	117.1339	S	0.0331	(0.38, 0.42)-(0.55, 0.42)	DataPointRange(10478 - 10480)	0.0331	0.17	5.26	7.45
 876	117.1782	F	0.3313	(0.58, 0.41)		DataPointRange(10481 - 10510)
 877	117.4983	S	0.0220	(0.58, 0.40)-(0.64, 0.41)	DataPointRange(10511 - 10512)	0.0220	0.07	3.19	5.26
 878	117.5316	F	0.1659	(0.65, 0.41)		DataPointRange(10513 - 10527)
 879	117.6860	S	0.0443	(0.66, 0.42)-(0.79, 0.41)	DataPointRange(10528 - 10531)	0.0443	0.14	3.12	5.13
 880	117.7414	F	0.2209	(0.82, 0.43)		DataPointRange(10532 - 10551)
 881	117.9511	S	0.0995	(0.82, 0.42)-(1.86, -0.32)	DataPointRange(10552 - 10560)	0.0995	1.18	11.90	190.56
 882	118.0620	F	0.0332	(1.87, -0.31)		DataPointRange(10561 - 10563)
 883	118.0836	S	0.0996	(1.87, -0.29)-(0.53, 0.38)	DataPointRange(10564 - 10572)	0.0996	1.43	14.37	270.90
 884	118.1944	F	0.0439	(0.52, 0.34)		DataPointRange(10573 - 10576)
 885	118.2275	S	0.0108	(0.50, 0.31)-(0.53, 0.36)	DataPointRange(10577 - 10577)	0.0108	0.04	4.07	4.07
 886	118.2494	F	0.4421	(0.53, 0.40)		DataPointRange(10578 - 10617)
 887	118.6801	S	0.0448	(0.52, 0.38)-(0.76, 0.44)	DataPointRange(10618 - 10621)	0.0448	0.24	5.43	10.11
 888	118.7354	F	0.3977	(0.76, 0.43)		DataPointRange(10622 - 10657)
 889	119.1221	S	0.0334	(0.77, 0.41)-(0.63, 0.35)	DataPointRange(10658 - 10660)	0.0334	0.15	4.47	7.63
 890	119.1662	F	0.2543	(0.59, 0.33)		DataPointRange(10661 - 10683)
 891	119.4093	S	0.0445	(0.61, 0.34)-(0.82, 0.44)	DataPointRange(10684 - 10687)	0.0445	0.22	5.04	7.48
 892	119.4645	F	0.1657	(0.82, 0.45)		DataPointRange(10688 - 10702)
 893	119.6191	S	0.0111	(0.81, 0.45)-(0.79, 0.44)	DataPointRange(10703 - 10703)	0.0111	0.02	1.89	1.89
 894	119.6412	F	0.2100	(0.79, 0.41)		DataPointRange(10704 - 10722)
 895	119.8400	S	0.0553	(0.80, 0.42)-(0.45, 0.34)	DataPointRange(10723 - 10727)	0.0553	0.35	6.30	11.75
 896	119.9063	F	0.2099	(0.44, 0.32)		DataPointRange(10728 - 10746)
 897	120.1052	S	0.0331	(0.43, 0.31)-(0.29, 0.27)	DataPointRange(10747 - 10749)	0.0331	0.14	4.31	8.08
 898	120.1383	End					DataPoint(10749)
 899	120.1496	F	0.3532	(0.25, 0.26)		DataPointRange(10750 - 10781)
 900	120.5028	aa1n9.bmp				DataPoint(10782)
 901	120.4920	S	0.0234	(0.25, 0.27)-(0.21, 0.22)	DataPointRange(10782 - 10783)	0.0234	0.06	2.36	3.70
 902	120.5250	F	0.3425	(0.20, 0.19)		DataPointRange(10784 - 10814)
 903	120.5801	Start					DataPoint(10789)
 904	120.8564	S	0.0222	(0.20, 0.15)-(0.28, 0.15)	DataPointRange(10815 - 10816)	0.0222	0.08	3.44	4.86
 905	120.8894	F	0.3869	(0.30, 0.15)		DataPointRange(10817 - 10851)
 906	121.2651	S	0.0221	(0.29, 0.16)-(0.40, 0.17)	DataPointRange(10852 - 10853)	0.0221	0.11	4.91	5.39
 907	121.2981	F	0.6848	(0.43, 0.17)		DataPointRange(10854 - 10915)
 908	121.9722	S	0.0329	(0.43, 0.17)-(0.57, 0.19)	DataPointRange(10916 - 10918)	0.0329	0.14	4.11	6.41
 909	122.0161	F	0.2982	(0.61, 0.19)		DataPointRange(10919 - 10945)
 910	122.3035	S	0.0219	(0.60, 0.20)-(0.65, 0.19)	DataPointRange(10946 - 10947)	0.0219	0.05	2.28	3.32
 911	122.3364	F	0.2874	(0.68, 0.17)		DataPointRange(10948 - 10973)
 912	122.6126	S	0.0112	(0.67, 0.20)-(0.70, 0.18)	DataPointRange(10974 - 10974)	0.0112	0.03	3.03	3.03
 913	122.6348	F	0.0110	(0.70, 0.15)		DataPointRange(10975 - 10975)
 914	122.6348	S	0.0337	(0.70, 0.15)-(0.80, 0.16)	DataPointRange(10976 - 10978)	0.0337	0.10	2.85	4.55
 915	122.6788	F	0.1327	(0.82, 0.18)		DataPointRange(10979 - 10990)
 916	122.8004	S	0.0773	(0.82, 0.15)-(0.23, 0.25)	DataPointRange(10991 - 10997)	0.0773	0.59	7.68	13.12
 917	122.8888	F	0.1436	(0.23, 0.25)		DataPointRange(10998 - 11010)
 918	123.0214	S	0.0110	(0.22, 0.24)-(0.22, 0.24)	DataPointRange(11011 - 11011)	0.0110	0.00	0.37	0.37
 919	123.0435	F	0.1437	(0.18, 0.26)		DataPointRange(11012 - 11024)
 920	123.1762	S	0.0772	(0.17, 0.29)-(0.32, 0.25)	DataPointRange(11025 - 11031)	0.0772	0.15	1.93	16.99
 921	123.2644	F	0.1876	(0.35, 0.26)		DataPointRange(11032 - 11048)
 922	123.4410	S	0.0332	(0.35, 0.27)-(0.47, 0.26)	DataPointRange(11049 - 11051)	0.0332	0.12	3.53	8.04
 923	123.4853	F	0.2319	(0.48, 0.25)		DataPointRange(11052 - 11072)
 924	123.7062	S	0.0221	(0.48, 0.27)-(0.57, 0.25)	DataPointRange(11073 - 11074)	0.0221	0.09	3.88	5.43
 925	123.7393	F	0.1768	(0.58, 0.25)		DataPointRange(11075 - 11090)
 926	123.9051	S	0.0331	(0.58, 0.24)-(0.71, 0.25)	DataPointRange(11091 - 11093)	0.0331	0.14	4.10	6.98
 927	123.9492	F	0.2984	(0.73, 0.26)		DataPointRange(11094 - 11120)
 928	124.2366	S	0.0663	(0.73, 0.27)-(0.19, 0.33)	DataPointRange(11121 - 11126)	0.0663	0.53	8.06	12.80
 929	124.3139	F	0.1546	(0.20, 0.34)		DataPointRange(11127 - 11140)
 930	124.4573	S	0.0223	(0.22, 0.34)-(0.16, 0.35)	DataPointRange(11141 - 11142)	0.0223	0.05	2.31	4.78
 931	124.4906	F	0.1434	(0.16, 0.35)		DataPointRange(11143 - 11155)
 932	124.6231	S	0.0333	(0.17, 0.35)-(0.35, 0.37)	DataPointRange(11156 - 11158)	0.0333	0.18	5.42	8.28
 933	124.6672	F	0.2651	(0.37, 0.34)		DataPointRange(11159 - 11182)
 934	124.9213	S	0.0220	(0.37, 0.32)-(0.44, 0.34)	DataPointRange(11183 - 11184)	0.0220	0.07	3.15	6.10
 935	124.9544	F	0.3537	(0.45, 0.34)		DataPointRange(11185 - 11216)
 936	125.2968	S	0.0221	(0.44, 0.33)-(0.39, 0.32)	DataPointRange(11217 - 11218)	0.0221	0.05	2.08	3.96
 937	125.3301	F	0.1879	(0.38, 0.32)		DataPointRange(11219 - 11235)
 938	125.5066	S	0.0995	(0.38, 0.32)-(1.87, -0.29)	DataPointRange(11236 - 11244)	0.0995	1.56	15.68	256.45
 939	125.6174	F	0.0995	(1.86, -0.32)		DataPointRange(11245 - 11253)
 940	125.7057	S	0.0992	(1.86, -0.32)-(0.40, 0.30)	DataPointRange(11254 - 11262)	0.0992	1.54	15.51	234.23
 941	125.8163	F	0.0332	(0.39, 0.25)		DataPointRange(11263 - 11265)
 942	125.8381	S	0.0114	(0.38, 0.25)-(0.39, 0.28)	DataPointRange(11266 - 11266)	0.0114	0.03	2.65	2.65
 943	125.8603	F	0.5302	(0.40, 0.31)		DataPointRange(11267 - 11314)
 944	126.3795	S	0.0220	(0.40, 0.31)-(0.47, 0.32)	DataPointRange(11315 - 11316)	0.0220	0.07	3.22	5.16
 945	126.4126	F	0.1988	(0.48, 0.32)		DataPointRange(11317 - 11334)
 946	126.6004	S	0.0332	(0.48, 0.32)-(0.36, 0.23)	DataPointRange(11335 - 11337)	0.0332	0.14	4.33	7.06
 947	126.6445	F	0.3426	(0.36, 0.22)		DataPointRange(11338 - 11368)
 948	126.9762	S	0.0220	(0.36, 0.23)-(0.30, 0.20)	DataPointRange(11369 - 11370)	0.0220	0.07	3.13	3.97
 949	127.0095	F	0.2537	(0.29, 0.21)		DataPointRange(11371 - 11393)
 950	127.2521	S	0.0334	(0.28, 0.22)-(0.41, 0.31)	DataPointRange(11394 - 11396)	0.0334	0.14	4.21	6.58
 951	127.2964	F	0.2097	(0.44, 0.32)		DataPointRange(11397 - 11415)
 952	127.4951	S	0.0331	(0.45, 0.33)-(0.30, 0.23)	DataPointRange(11416 - 11418)	0.0331	0.17	5.16	8.37
 953	127.5396	F	0.4417	(0.30, 0.20)		DataPointRange(11419 - 11458)
 954	127.7272	End					DataPoint(11436)
 955	127.9705	S	0.0219	(0.31, 0.21)-(0.22, 0.19)	DataPointRange(11459 - 11460)	0.0219	0.09	4.07	6.11
 956	128.0039	F	0.2424	(0.24, 0.19)		DataPointRange(11461 - 11482)
 957	128.0838	aa1n18s.bmp				DataPoint(11468)
 958	128.1578	Start					DataPoint(11475)
 959	128.2353	S	0.0222	(0.24, 0.19)-(0.16, 0.14)	DataPointRange(11483 - 11484)	0.0222	0.09	4.01	5.09
 960	128.2689	F	0.2320	(0.17, 0.15)		DataPointRange(11485 - 11505)
 961	128.4893	S	0.0334	(0.17, 0.15)-(0.35, 0.16)	DataPointRange(11506 - 11508)	0.0334	0.19	5.60	9.85
 962	128.5336	F	0.2427	(0.38, 0.16)		DataPointRange(11509 - 11530)
 963	128.7653	S	0.0334	(0.38, 0.16)-(0.51, 0.16)	DataPointRange(11531 - 11533)	0.0334	0.13	3.80	6.42
 964	128.8095	F	0.2544	(0.56, 0.17)		DataPointRange(11534 - 11556)
 965	129.0527	S	0.0112	(0.57, 0.17)-(0.60, 0.17)	DataPointRange(11557 - 11557)	0.0112	0.03	2.45	2.45
 966	129.0748	F	0.1437	(0.65, 0.16)		DataPointRange(11558 - 11570)
 967	129.2075	S	0.0219	(0.65, 0.16)-(0.75, 0.16)	DataPointRange(11571 - 11572)	0.0219	0.10	4.37	7.19
 968	129.2406	F	0.1556	(0.78, 0.17)		DataPointRange(11573 - 11586)
 969	129.3842	S	0.0663	(0.78, 0.18)-(0.19, 0.21)	DataPointRange(11587 - 11592)	0.0663	0.59	8.88	13.38
 970	129.4618	F	0.2427	(0.18, 0.25)		DataPointRange(11593 - 11614)
 971	129.6934	S	0.0331	(0.17, 0.25)-(0.31, 0.26)	DataPointRange(11615 - 11617)	0.0331	0.14	4.20	6.10
 972	129.7377	F	0.3643	(0.35, 0.25)		DataPointRange(11618 - 11650)
 973	130.0910	S	0.0330	(0.36, 0.25)-(0.51, 0.27)	DataPointRange(11651 - 11653)	0.0330	0.15	4.60	6.54
 974	130.1350	F	0.3319	(0.52, 0.25)		DataPointRange(11654 - 11683)
 975	130.4558	S	0.0220	(0.51, 0.26)-(0.58, 0.26)	DataPointRange(11684 - 11685)	0.0220	0.07	3.31	4.49
 976	130.4886	F	0.2542	(0.62, 0.26)		DataPointRange(11686 - 11708)
 977	130.7316	S	0.0222	(0.62, 0.26)-(0.67, 0.26)	DataPointRange(11709 - 11710)	0.0222	0.05	2.18	4.09
 978	130.7649	F	0.2540	(0.69, 0.26)		DataPointRange(11711 - 11733)
 979	131.0080	S	0.0662	(0.70, 0.29)-(0.23, 0.35)	DataPointRange(11734 - 11739)	0.0662	0.47	7.06	11.48
 980	131.0854	F	0.3755	(0.21, 0.35)		DataPointRange(11740 - 11773)
 981	131.4499	S	0.0220	(0.20, 0.35)-(0.32, 0.35)	DataPointRange(11774 - 11775)	0.0220	0.13	5.74	6.62
 982	131.4829	F	0.2542	(0.35, 0.33)		DataPointRange(11776 - 11798)
 983	131.7261	S	0.0329	(0.37, 0.33)-(0.48, 0.34)	DataPointRange(11799 - 11801)	0.0329	0.12	3.65	6.43
 984	131.7702	F	0.2650	(0.53, 0.33)		DataPointRange(11802 - 11825)
 985	132.0242	S	0.0110	(0.54, 0.32)-(0.58, 0.34)	DataPointRange(11826 - 11826)	0.0110	0.05	4.17	4.17
 986	132.0463	F	0.2318	(0.60, 0.34)		DataPointRange(11827 - 11847)
 987	132.2669	S	0.0443	(0.59, 0.36)-(0.77, 0.34)	DataPointRange(11848 - 11851)	0.0443	0.18	4.04	5.93
 988	132.3222	F	0.3760	(0.78, 0.35)		DataPointRange(11852 - 11885)
 989	132.6871	S	0.0771	(0.78, 0.34)-(0.18, 0.45)	DataPointRange(11886 - 11892)	0.0771	0.60	7.76	11.79
 990	132.7754	F	0.1659	(0.17, 0.44)		DataPointRange(11893 - 11907)
 991	132.9300	S	0.0113	(0.18, 0.45)-(0.16, 0.45)	DataPointRange(11908 - 11908)	0.0113	0.02	1.66	1.66
 992	132.9521	F	0.1989	(0.14, 0.46)		DataPointRange(11909 - 11926)
 993	133.1399	S	0.0345	(0.15, 0.46)-(0.30, 0.45)	DataPointRange(11927 - 11929)	0.0345	0.16	4.57	8.68
 994	133.1842	F	0.4305	(0.33, 0.44)		DataPointRange(11930 - 11968)
 995	133.6043	S	0.0217	(0.34, 0.43)-(0.24, 0.41)	DataPointRange(11969 - 11970)	0.0217	0.10	4.57	6.19
 996	133.6369	F	0.1547	(0.21, 0.44)		DataPointRange(11971 - 11984)
 997	133.7807	S	0.0548	(0.22, 0.45)-(0.53, 0.44)	DataPointRange(11985 - 11989)	0.0548	0.31	5.72	8.67
 998	133.8467	F	0.3537	(0.52, 0.43)		DataPointRange(11990 - 12021)
 999	134.1893	S	0.0330	(0.51, 0.41)-(0.63, 0.43)	DataPointRange(12022 - 12024)	0.0330	0.13	3.80	6.33
1000	134.2335	F	0.2538	(0.66, 0.41)		DataPointRange(12025 - 12047)
1001	134.4762	S	0.0664	(0.67, 0.40)-(0.18, 0.50)	DataPointRange(12048 - 12053)	0.0664	0.49	7.34	10.68
1002	134.5536	F	0.3205	(0.18, 0.53)		DataPointRange(12054 - 12082)
1003	134.8633	S	0.0329	(0.18, 0.56)-(0.31, 0.53)	DataPointRange(12083 - 12085)	0.0329	0.13	3.87	6.60
1004	134.9073	F	0.2430	(0.32, 0.52)		DataPointRange(12086 - 12107)
1005	135.1394	S	0.0218	(0.32, 0.51)-(0.43, 0.52)	DataPointRange(12108 - 12109)	0.0218	0.11	4.96	7.34
1006	135.1898	F	0.2149	(0.47, 0.52)		DataPointRange(12110 - 12129)
1007	135.3934	S	0.0221	(0.47, 0.50)-(0.54, 0.52)	DataPointRange(12130 - 12131)	0.0221	0.07	3.04	5.90
1008	135.4265	F	0.4528	(0.57, 0.51)		DataPointRange(12132 - 12172)
1009	135.8687	S	0.0332	(0.56, 0.51)-(0.70, 0.50)	DataPointRange(12173 - 12175)	0.0332	0.14	4.26	8.24
1010	135.9126	F	0.1879	(0.74, 0.52)		DataPointRange(12176 - 12192)
1011	136.0892	S	0.0221	(0.73, 0.53)-(0.83, 0.54)	DataPointRange(12193 - 12194)	0.0221	0.10	4.63	7.24
1012	136.1223	F	0.1879	(0.83, 0.52)		DataPointRange(12195 - 12211)
1013	136.2992	S	0.0773	(0.82, 0.50)-(0.19, 0.61)	DataPointRange(12212 - 12218)	0.0773	0.64	8.25	13.73
1014	136.3874	F	0.2099	(0.17, 0.64)		DataPointRange(12219 - 12237)
1015	136.5861	S	0.0112	(0.17, 0.64)-(0.17, 0.63)	DataPointRange(12238 - 12238)	0.0112	0.01	1.01	1.01
1016	136.6082	F	0.1656	(0.14, 0.65)		DataPointRange(12239 - 12253)
1017	136.7628	S	0.0335	(0.13, 0.68)-(0.27, 0.61)	DataPointRange(12254 - 12256)	0.0335	0.15	4.47	6.97
1018	136.8071	F	0.2100	(0.28, 0.60)		DataPointRange(12257 - 12275)
1019	137.0061	S	0.0222	(0.30, 0.62)-(0.41, 0.60)	DataPointRange(12276 - 12277)	0.0222	0.11	4.74	7.93
1020	137.0390	F	0.2101	(0.44, 0.61)		DataPointRange(12278 - 12296)
1021	137.2381	S	0.0331	(0.45, 0.60)-(0.61, 0.60)	DataPointRange(12297 - 12299)	0.0331	0.16	4.85	8.21
1022	137.2822	F	0.2873	(0.66, 0.61)		DataPointRange(12300 - 12325)
1023	137.5584	S	0.0221	(0.66, 0.61)-(0.77, 0.57)	DataPointRange(12326 - 12327)	0.0221	0.12	5.21	5.35
1024	137.5919	F	0.2755	(0.78, 0.60)		DataPointRange(12328 - 12352)
1025	137.8564	S	0.0884	(0.78, 0.58)-(1.86, -0.36)	DataPointRange(12353 - 12360)	0.0884	1.29	14.58	130.58
1026	137.9563	F	0.0107	(1.86, -0.36)		DataPointRange(12361 - 12361)
1027	137.9563	S	0.0218	(1.86, -0.36)-(1.87, -0.28)	DataPointRange(12362 - 12363)	0.0218	0.06	2.89	6.00
1028	137.9894	F	0.0108	(1.87, -0.28)		DataPointRange(12364 - 12364)
1029	137.9894	S	0.0218	(1.87, -0.28)-(1.86, -0.36)	DataPointRange(12365 - 12366)	0.0218	0.06	2.89	5.73
1030	138.0225	F	0.0109	(-0.07, 0.23)		DataPointRange(12367 - 12367)
1031	138.0225	S	0.0772	(-0.07, 0.23)-(0.52, 0.33)	DataPointRange(12368 - 12374)	0.0772	0.59	7.64	181.34
1032	138.1105	F	0.0117	(0.51, 0.33)		DataPointRange(12375 - 12375)
1033	138.1105	S	0.0117	(0.51, 0.33)-(0.51, 0.29)	DataPointRange(12376 - 12376)	0.0117	0.03	2.66	2.66
1034	138.1328	F	0.0110	(0.51, 0.27)		DataPointRange(12377 - 12377)
1035	138.1328	S	0.0110	(0.51, 0.27)-(0.51, 0.31)	DataPointRange(12378 - 12378)	0.0110	0.03	3.05	3.05
1036	138.1552	F	0.3420	(0.51, 0.34)		DataPointRange(12379 - 12409)
1037	138.4866	S	0.0438	(0.52, 0.34)-(0.66, 0.59)	DataPointRange(12410 - 12413)	0.0438	0.24	5.39	7.61
1038	138.5415	F	0.3863	(0.67, 0.58)		DataPointRange(12414 - 12448)
1039	138.9167	S	0.0222	(0.69, 0.58)-(0.59, 0.54)	DataPointRange(12449 - 12450)	0.0222	0.11	4.77	7.48
1040	138.9502	F	0.0111	(0.59, 0.50)		DataPointRange(12451 - 12451)
1041	138.9502	S	0.1434	(0.59, 0.50)-(0.53, 0.55)	DataPointRange(12452 - 12464)	0.1434	0.07	0.49	17.24
1042	139.1049	F	0.3427	(0.53, 0.52)		DataPointRange(12465 - 12495)
1043	139.4362	S	0.0332	(0.54, 0.52)-(0.68, 0.56)	DataPointRange(12496 - 12498)	0.0332	0.14	4.25	6.33
1044	139.4808	F	0.3089	(0.68, 0.58)		DataPointRange(12499 - 12526)
1045	139.7789	S	0.0329	(0.69, 0.59)-(0.54, 0.59)	DataPointRange(12527 - 12529)	0.0329	0.14	4.39	8.30
1046	139.8229	F	0.2760	(0.51, 0.57)		DataPointRange(12530 - 12554)
1047	140.0879	S	0.0332	(0.51, 0.56)-(0.35, 0.52)	DataPointRange(12555 - 12557)	0.0332	0.16	4.69	6.86
1048	140.1320	F	0.2543	(0.37, 0.51)		DataPointRange(12558 - 12580)
1049	140.3755	S	0.0439	(0.37, 0.52)-(0.26, 0.32)	DataPointRange(12581 - 12584)	0.0439	0.18	4.07	7.52
1050	140.4304	F	0.2320	(0.28, 0.29)		DataPointRange(12585 - 12605)
1051	140.6514	S	0.0331	(0.28, 0.28)-(0.40, 0.26)	DataPointRange(12606 - 12608)	0.0331	0.12	3.54	7.86
1052	140.6956	F	0.2647	(0.41, 0.26)		DataPointRange(12609 - 12632)
1053	140.9493	S	0.0888	(0.42, 0.26)-(-0.35, 1.25)	DataPointRange(12633 - 12640)	0.0888	1.07	12.05	127.49
1054	141.0487	F	0.0111	(-0.39, 1.33)		DataPointRange(12641 - 12641)
1055	141.0487	S	0.1219	(-0.39, 1.33)-(0.35, 0.29)	DataPointRange(12642 - 12652)	0.1219	1.08	8.83	41.12
1056	141.1373	End					DataPoint(12649)
1057	141.1813	F	0.2764	(0.36, 0.26)		DataPointRange(12653 - 12677)
1058	141.4467	S	0.0222	(0.37, 0.27)-(0.25, 0.21)	DataPointRange(12678 - 12679)	0.0222	0.13	5.91	7.73
1059	141.4796	F	0.1769	(0.25, 0.23)		DataPointRange(12680 - 12695)
1060	141.5127	t1n1.bmp					DataPoint(12683)
1061	141.5568	Start					DataPoint(12687)
1062	141.6454	S	0.0222	(0.25, 0.23)-(0.21, 0.19)	DataPointRange(12696 - 12697)	0.0222	0.05	2.46	4.44
1063	141.6786	F	0.4196	(0.18, 0.15)		DataPointRange(12698 - 12735)
1064	142.0870	S	0.0445	(0.18, 0.14)-(0.40, 0.16)	DataPointRange(12736 - 12739)	0.0445	0.23	5.08	7.38
1065	142.1425	F	0.2544	(0.39, 0.16)		DataPointRange(12740 - 12762)
1066	142.3857	S	0.0220	(0.39, 0.16)-(0.46, 0.16)	DataPointRange(12763 - 12764)	0.0220	0.07	3.20	6.19
1067	142.4187	F	0.2210	(0.51, 0.16)		DataPointRange(12765 - 12784)
1068	142.6285	S	0.0222	(0.50, 0.18)-(0.58, 0.16)	DataPointRange(12785 - 12786)	0.0222	0.08	3.63	5.00
1069	142.6617	F	0.1877	(0.62, 0.16)		DataPointRange(12787 - 12803)
1070	142.8384	S	0.0222	(0.62, 0.17)-(0.71, 0.17)	DataPointRange(12804 - 12805)	0.0222	0.09	3.92	6.36
1071	142.8716	F	0.1768	(0.72, 0.17)		DataPointRange(12806 - 12821)
1072	143.0375	S	0.0219	(0.72, 0.17)-(0.78, 0.16)	DataPointRange(12822 - 12823)	0.0219	0.06	2.62	4.87
1073	143.0706	F	0.1652	(0.83, 0.16)		DataPointRange(12824 - 12838)
1074	143.2249	S	0.0774	(0.83, 0.15)-(0.18, 0.27)	DataPointRange(12839 - 12845)	0.0774	0.66	8.51	12.47
1075	143.3135	F	0.3092	(0.17, 0.27)		DataPointRange(12846 - 12873)
1076	143.6117	S	0.0332	(0.15, 0.26)-(0.28, 0.24)	DataPointRange(12874 - 12876)	0.0332	0.13	3.99	6.61
1077	143.6564	F	0.1873	(0.32, 0.25)		DataPointRange(12877 - 12893)
1078	143.8325	S	0.0774	(0.33, 0.26)-(0.35, 0.22)	DataPointRange(12894 - 12900)	0.0774	0.04	0.50	13.74
1079	143.9214	F	0.1429	(0.37, 0.26)		DataPointRange(12901 - 12913)
1080	144.0533	S	0.0221	(0.37, 0.26)-(0.46, 0.25)	DataPointRange(12914 - 12915)	0.0221	0.10	4.39	6.88
1081	144.0866	F	0.2097	(0.50, 0.25)		DataPointRange(12916 - 12934)
1082	144.2855	S	0.0439	(0.50, 0.25)-(0.67, 0.26)	DataPointRange(12935 - 12938)	0.0439	0.16	3.75	6.07
1083	144.3404	F	0.2876	(0.68, 0.25)		DataPointRange(12939 - 12964)
1084	144.6171	S	0.0109	(0.69, 0.26)-(0.66, 0.24)	DataPointRange(12965 - 12965)	0.0109	0.03	3.02	3.02
1085	144.6389	F	0.2767	(0.64, 0.25)		DataPointRange(12966 - 12990)
1086	144.9040	S	0.0663	(0.63, 0.25)-(0.15, 0.36)	DataPointRange(12991 - 12996)	0.0663	0.49	7.46	12.39
1087	144.9816	F	0.2320	(0.14, 0.35)		DataPointRange(12997 - 13017)
1088	145.2023	S	0.0222	(0.15, 0.36)-(0.22, 0.35)	DataPointRange(13018 - 13019)	0.0222	0.07	3.25	5.07
1089	145.2353	F	0.1766	(0.24, 0.35)		DataPointRange(13020 - 13035)
1090	145.4010	S	0.0220	(0.24, 0.38)-(0.33, 0.32)	DataPointRange(13036 - 13037)	0.0220	0.10	4.51	5.25
1091	145.4340	F	0.2764	(0.36, 0.34)		DataPointRange(13038 - 13062)
1092	145.6994	S	0.0331	(0.36, 0.34)-(0.51, 0.35)	DataPointRange(13063 - 13065)	0.0331	0.15	4.43	7.68
1093	145.7437	F	0.2981	(0.54, 0.35)		DataPointRange(13066 - 13092)
1094	146.0307	S	0.0111	(0.55, 0.36)-(0.57, 0.36)	DataPointRange(13093 - 13093)	0.0111	0.02	1.81	1.81
1095	146.0529	F	0.2872	(0.61, 0.34)		DataPointRange(13094 - 13119)
1096	146.3289	S	0.0995	(0.60, 0.34)-(1.86, -0.32)	DataPointRange(13120 - 13128)	0.0995	1.36	13.66	121.67
1097	146.4397	F	0.0107	(-0.19, 1.88)		DataPointRange(13129 - 13129)
1098	146.4397	S	0.0992	(-0.19, 1.88)-(0.55, 0.34)	DataPointRange(13130 - 13138)	0.0992	1.37	13.84	246.60
1099	146.5500	F	0.4864	(0.55, 0.35)		DataPointRange(13139 - 13182)
1100	147.0249	S	0.0115	(0.55, 0.35)-(0.52, 0.35)	DataPointRange(13183 - 13183)	0.0115	0.02	2.03	2.03
1101	147.0469	F	0.3211	(0.51, 0.34)		DataPointRange(13184 - 13212)
1102	147.3564	S	0.0220	(0.51, 0.33)-(0.58, 0.33)	DataPointRange(13213 - 13214)	0.0220	0.08	3.45	3.74
1103	147.3897	F	0.3756	(0.60, 0.34)		DataPointRange(13215 - 13248)
1104	147.7540	S	0.0222	(0.60, 0.33)-(0.51, 0.35)	DataPointRange(13249 - 13250)	0.0222	0.10	4.29	7.45
1105	147.7872	F	0.2430	(0.48, 0.33)		DataPointRange(13251 - 13272)
1106	148.0083	End					DataPoint(13271)
1107	148.0192	S	0.0330	(0.49, 0.31)-(0.35, 0.33)	DataPointRange(13273 - 13275)	0.0330	0.13	4.04	6.07
1108	148.0638	F	0.1874	(0.36, 0.32)		DataPointRange(13276 - 13292)
1109	148.2400	S	0.0331	(0.35, 0.32)-(0.23, 0.26)	DataPointRange(13293 - 13295)	0.0331	0.13	3.95	8.13
1110	148.2842	F	0.2319	(0.20, 0.24)		DataPointRange(13296 - 13316)
1111	148.3836	aa1n1s.bmp				DataPoint(13305)
1112	148.4276	Start					DataPoint(13309)
1113	148.5051	S	0.0221	(0.20, 0.24)-(0.13, 0.18)	DataPointRange(13317 - 13318)	0.0221	0.08	3.64	5.79
1114	148.5382	F	0.1435	(0.16, 0.16)		DataPointRange(13319 - 13331)
1115	148.6708	S	0.0224	(0.16, 0.17)-(0.22, 0.16)	DataPointRange(13332 - 13333)	0.0224	0.06	2.81	5.39
1116	148.7039	F	0.2100	(0.24, 0.16)		DataPointRange(13334 - 13352)
1117	148.9028	S	0.0333	(0.23, 0.18)-(0.37, 0.16)	DataPointRange(13353 - 13355)	0.0333	0.14	4.11	7.95
1118	148.9469	F	0.1876	(0.42, 0.16)		DataPointRange(13356 - 13372)
1119	149.1237	S	0.0108	(0.43, 0.15)-(0.38, 0.15)	DataPointRange(13373 - 13373)	0.0108	0.05	4.47	4.47
1120	149.1457	F	0.2432	(0.35, 0.15)		DataPointRange(13374 - 13395)
1121	149.3777	S	0.0333	(0.34, 0.14)-(0.50, 0.18)	DataPointRange(13396 - 13398)	0.0333	0.16	4.77	7.95
1122	149.4225	F	0.3639	(0.52, 0.18)		DataPointRange(13399 - 13431)
1123	149.7752	S	0.0331	(0.51, 0.17)-(0.60, 0.17)	DataPointRange(13432 - 13434)	0.0331	0.09	2.68	4.18
1124	149.8197	F	0.2319	(0.60, 0.16)		DataPointRange(13435 - 13455)
1125	150.0406	S	0.0331	(0.62, 0.15)-(0.49, 0.15)	DataPointRange(13456 - 13458)	0.0331	0.12	3.69	6.87
1126	150.0847	F	0.2762	(0.49, 0.16)		DataPointRange(13459 - 13483)
1127	150.3503	S	0.0547	(0.49, 0.17)-(0.15, 0.23)	DataPointRange(13484 - 13488)	0.0547	0.34	6.29	9.10
1128	150.4164	F	0.2648	(0.15, 0.24)		DataPointRange(13489 - 13512)
1129	150.6700	S	0.0223	(0.14, 0.25)-(0.24, 0.25)	DataPointRange(13513 - 13514)	0.0223	0.10	4.68	6.91
1130	150.7033	F	0.2207	(0.26, 0.24)		DataPointRange(13515 - 13534)
1131	150.9132	S	0.0441	(0.25, 0.23)-(0.44, 0.26)	DataPointRange(13535 - 13538)	0.0441	0.20	4.47	6.50
1132	150.9684	F	0.2651	(0.47, 0.25)		DataPointRange(13539 - 13562)
1133	151.2224	S	0.0332	(0.47, 0.25)-(0.55, 0.27)	DataPointRange(13563 - 13565)	0.0332	0.09	2.60	3.72
1134	151.2667	F	0.1768	(0.56, 0.25)		DataPointRange(13566 - 13581)
1135	151.4323	S	0.0333	(0.56, 0.25)-(0.68, 0.25)	DataPointRange(13582 - 13584)	0.0333	0.12	3.49	6.15
1136	151.4770	F	0.2320	(0.72, 0.24)		DataPointRange(13585 - 13605)
1137	151.6975	S	0.0662	(0.71, 0.23)-(0.17, 0.34)	DataPointRange(13606 - 13611)	0.0662	0.55	8.28	12.06
1138	151.7751	F	0.3089	(0.18, 0.34)		DataPointRange(13612 - 13639)
1139	152.0734	S	0.0106	(0.18, 0.34)-(0.22, 0.35)	DataPointRange(13640 - 13640)	0.0106	0.04	3.40	3.40
1140	152.0952	F	0.1546	(0.25, 0.33)		DataPointRange(13641 - 13654)
1141	152.2391	S	0.0440	(0.26, 0.32)-(0.42, 0.33)	DataPointRange(13655 - 13658)	0.0440	0.16	3.69	5.54
1142	152.2940	F	0.1988	(0.43, 0.34)		DataPointRange(13659 - 13676)
1143	152.4818	S	0.0439	(0.43, 0.34)-(0.61, 0.35)	DataPointRange(13677 - 13680)	0.0439	0.18	4.05	5.83
1144	152.5372	F	0.2761	(0.60, 0.34)		DataPointRange(13681 - 13705)
1145	152.8022	S	0.0219	(0.60, 0.34)-(0.66, 0.36)	DataPointRange(13706 - 13707)	0.0219	0.06	2.91	6.44
1146	152.8358	F	0.8500	(0.66, 0.34)		DataPointRange(13708 - 13784)
1147	153.6747	S	0.1104	(0.65, 0.34)-(1.86, -0.33)	DataPointRange(13785 - 13794)	0.1104	1.31	11.86	217.29
1148	153.7966	F	0.0438	(1.87, -0.31)		DataPointRange(13795 - 13798)
1149	153.8297	S	0.0880	(1.87, -0.29)-(0.68, 0.31)	DataPointRange(13799 - 13806)	0.0880	1.27	14.46	119.86
1150	153.9291	F	0.0331	(0.68, 0.29)		DataPointRange(13807 - 13809)
1151	153.9509	S	0.0113	(0.67, 0.28)-(0.68, 0.34)	DataPointRange(13810 - 13810)	0.0113	0.05	4.19	4.19
1152	153.9730	F	0.3645	(0.68, 0.33)		DataPointRange(13811 - 13843)
1153	154.3269	S	0.0216	(0.67, 0.33)-(0.58, 0.34)	DataPointRange(13844 - 13845)	0.0216	0.09	4.13	7.76
1154	154.3600	F	0.5851	(0.55, 0.34)		DataPointRange(13846 - 13898)
1155	154.9340	S	0.0332	(0.55, 0.33)-(0.44, 0.32)	DataPointRange(13899 - 13901)	0.0332	0.11	3.21	6.18
1156	154.9780	F	0.1878	(0.44, 0.32)		DataPointRange(13902 - 13918)
1157	155.1547	S	0.0332	(0.44, 0.31)-(0.28, 0.20)	DataPointRange(13919 - 13921)	0.0332	0.18	5.43	7.81
1158	155.1989	F	0.2323	(0.27, 0.18)		DataPointRange(13922 - 13942)
1159	155.4204	S	0.0108	(0.27, 0.16)-(0.25, 0.15)	DataPointRange(13943 - 13943)	0.0108	0.03	2.32	2.32
1160	155.4422	F	0.5191	(0.24, 0.17)		DataPointRange(13944 - 13990)
1161	155.4531	End					DataPoint(13945)
1162	155.8287	aa1n7s.bmp				DataPoint(13979)
1163	155.9061	Start					DataPoint(13986)
1164	155.9502	S	0.0331	(0.24, 0.18)-(0.14, 0.14)	DataPointRange(13991 - 13993)	0.0331	0.10	3.03	5.01
1165	155.9944	F	0.1326	(0.16, 0.14)		DataPointRange(13994 - 14005)
1166	156.1160	S	0.0335	(0.15, 0.15)-(0.27, 0.15)	DataPointRange(14006 - 14008)	0.0335	0.12	3.44	5.52
1167	156.1600	F	0.1767	(0.27, 0.16)		DataPointRange(14009 - 14024)
1168	156.3256	S	0.0221	(0.27, 0.16)-(0.37, 0.15)	DataPointRange(14025 - 14026)	0.0221	0.10	4.54	8.01
1169	156.3588	F	0.2323	(0.43, 0.15)		DataPointRange(14027 - 14047)
1170	156.5803	S	0.0108	(0.44, 0.14)-(0.45, 0.17)	DataPointRange(14048 - 14048)	0.0108	0.03	2.49	2.49
1171	156.6019	F	0.2321	(0.49, 0.18)		DataPointRange(14049 - 14069)
1172	156.8229	S	0.0111	(0.50, 0.18)-(0.53, 0.17)	DataPointRange(14070 - 14070)	0.0111	0.04	3.39	3.39
1173	156.8453	F	0.1873	(0.55, 0.17)		DataPointRange(14071 - 14087)
1174	157.0217	S	0.0332	(0.56, 0.17)-(0.68, 0.15)	DataPointRange(14088 - 14090)	0.0332	0.12	3.72	7.14
1175	157.0658	F	0.2650	(0.70, 0.17)		DataPointRange(14091 - 14114)
1176	157.3200	S	0.0219	(0.70, 0.16)-(0.75, 0.16)	DataPointRange(14115 - 14116)	0.0219	0.05	2.36	4.03
1177	157.3528	F	0.2545	(0.78, 0.16)		DataPointRange(14117 - 14139)
1178	157.5962	S	0.0778	(0.77, 0.14)-(0.18, 0.19)	DataPointRange(14140 - 14146)	0.0778	0.59	7.62	12.12
1179	157.6843	F	0.3206	(0.15, 0.25)		DataPointRange(14147 - 14175)
1180	157.9939	S	0.0332	(0.16, 0.24)-(0.29, 0.24)	DataPointRange(14176 - 14178)	0.0332	0.14	4.14	8.13
1181	158.0382	F	0.5963	(0.32, 0.25)		DataPointRange(14179 - 14232)
1182	158.6232	S	0.0223	(0.33, 0.25)-(0.37, 0.22)	DataPointRange(14233 - 14234)	0.0223	0.05	2.37	4.56
1183	158.6565	F	0.1991	(0.39, 0.24)		DataPointRange(14235 - 14252)
1184	158.8444	S	0.0221	(0.39, 0.25)-(0.50, 0.26)	DataPointRange(14253 - 14254)	0.0221	0.11	5.13	7.36
1185	158.8776	F	0.8945	(0.53, 0.25)		DataPointRange(14255 - 14335)
1186	159.7610	S	0.0336	(0.53, 0.25)-(0.66, 0.24)	DataPointRange(14336 - 14338)	0.0336	0.13	3.83	6.82
1187	159.8053	F	0.2431	(0.69, 0.25)		DataPointRange(14339 - 14360)
1188	160.0373	S	0.0111	(0.71, 0.23)-(0.74, 0.24)	DataPointRange(14361 - 14361)	0.0111	0.03	2.62	2.62
1189	160.0599	F	0.1431	(0.76, 0.25)		DataPointRange(14362 - 14374)
1190	160.1922	S	0.0771	(0.74, 0.24)-(0.15, 0.36)	DataPointRange(14375 - 14381)	0.0771	0.59	7.70	12.31
1191	160.2802	F	0.3427	(0.16, 0.36)		DataPointRange(14382 - 14412)
1192	160.6118	S	0.0329	(0.15, 0.34)-(0.26, 0.34)	DataPointRange(14413 - 14415)	0.0329	0.11	3.26	5.29
1193	160.6560	F	0.1767	(0.28, 0.34)		DataPointRange(14416 - 14431)
1194	160.8217	S	0.0332	(0.28, 0.34)-(0.41, 0.32)	DataPointRange(14432 - 14434)	0.0332	0.13	3.99	6.84
1195	160.8658	F	0.2209	(0.41, 0.33)		DataPointRange(14435 - 14454)
1196	161.0757	S	0.0332	(0.41, 0.33)-(0.54, 0.32)	DataPointRange(14455 - 14457)	0.0332	0.14	4.11	5.84
1197	161.1201	F	0.2654	(0.55, 0.33)		DataPointRange(14458 - 14481)
1198	161.3739	S	0.0221	(0.55, 0.31)-(0.61, 0.32)	DataPointRange(14482 - 14483)	0.0221	0.05	2.33	5.00
1199	161.4071	F	0.5192	(0.61, 0.32)		DataPointRange(14484 - 14530)
1200	161.9150	S	0.0336	(0.61, 0.32)-(0.40, 0.28)	DataPointRange(14531 - 14533)	0.0336	0.21	6.30	9.35
1201	161.9594	F	0.3425	(0.35, 0.26)		DataPointRange(14534 - 14564)
1202	162.2908	S	0.0889	(0.34, 0.25)-(1.86, -0.33)	DataPointRange(14565 - 14572)	0.0889	1.58	17.81	219.23
1203	162.3903	F	0.0111	(1.86, -0.32)		DataPointRange(14573 - 14573)
1204	162.3903	S	0.0111	(1.86, -0.32)-(1.86, -0.32)	DataPointRange(14574 - 14574)	0.0111	0.01	0.51	0.51
1205	162.4127	F	0.0107	(1.87, -0.22)		DataPointRange(14575 - 14575)
1206	162.4127	S	0.0879	(1.87, -0.22)-(0.66, 0.31)	DataPointRange(14576 - 14583)	0.0879	1.28	14.53	116.65
1207	162.5119	F	0.5743	(0.66, 0.35)		DataPointRange(14584 - 14635)
1208	163.0754	S	0.0328	(0.65, 0.34)-(0.54, 0.33)	DataPointRange(14636 - 14638)	0.0328	0.11	3.32	5.59
1209	163.1194	F	0.3422	(0.54, 0.33)		DataPointRange(14639 - 14669)
1210	163.4506	S	0.0331	(0.55, 0.32)-(0.66, 0.35)	DataPointRange(14670 - 14672)	0.0331	0.12	3.52	6.93
1211	163.4947	F	0.1769	(0.67, 0.34)		DataPointRange(14673 - 14688)
1212	163.6605	S	0.0445	(0.68, 0.34)-(0.38, 0.26)	DataPointRange(14689 - 14692)	0.0445	0.30	6.79	10.83
1213	163.7157	F	0.1658	(0.37, 0.24)		DataPointRange(14693 - 14707)
1214	163.7929	End					DataPoint(14700)
1215	163.8708	S	0.0330	(0.37, 0.25)-(0.24, 0.21)	DataPointRange(14708 - 14710)	0.0330	0.13	4.04	6.52
1216	163.9146	F	0.3754	(0.25, 0.20)		DataPointRange(14711 - 14744)
1217	164.1463	aa1n2s.bmp				DataPoint(14732)
1218	164.2022	Start					DataPoint(14737)
1219	164.2789	S	0.0224	(0.24, 0.22)-(0.20, 0.15)	DataPointRange(14745 - 14746)	0.0224	0.06	2.85	4.65
1220	164.3121	F	0.2876	(0.18, 0.13)		DataPointRange(14747 - 14772)
1221	164.5883	S	0.0114	(0.18, 0.12)-(0.19, 0.12)	DataPointRange(14773 - 14773)	0.0114	0.01	1.10	1.10
1222	164.6106	F	0.2651	(0.24, 0.15)		DataPointRange(14774 - 14797)
1223	164.8645	S	0.0329	(0.23, 0.14)-(0.42, 0.15)	DataPointRange(14798 - 14800)	0.0329	0.19	5.68	7.80
1224	164.9087	F	0.2729	(0.43, 0.16)		DataPointRange(14801 - 14824)
1225	165.1625	S	0.0332	(0.41, 0.14)-(0.54, 0.17)	DataPointRange(14825 - 14827)	0.0332	0.13	3.91	8.80
1226	165.2068	F	0.3756	(0.57, 0.17)		DataPointRange(14828 - 14861)
1227	165.5715	S	0.0221	(0.56, 0.16)-(0.62, 0.16)	DataPointRange(14862 - 14863)	0.0221	0.06	2.56	2.97
1228	165.6044	F	0.2547	(0.65, 0.16)		DataPointRange(14864 - 14886)
1229	165.8478	S	0.0551	(0.65, 0.16)-(0.19, 0.23)	DataPointRange(14887 - 14891)	0.0551	0.46	8.29	12.23
1230	165.9138	F	0.2872	(0.18, 0.26)		DataPointRange(14892 - 14917)
1231	166.1903	S	0.0219	(0.19, 0.24)-(0.24, 0.26)	DataPointRange(14918 - 14919)	0.0219	0.06	2.82	5.94
1232	166.2232	F	0.2319	(0.28, 0.25)		DataPointRange(14920 - 14940)
1233	166.4441	S	0.0331	(0.28, 0.25)-(0.43, 0.25)	DataPointRange(14941 - 14943)	0.0331	0.15	4.51	6.69
1234	166.4887	F	0.2205	(0.45, 0.25)		DataPointRange(14944 - 14963)
1235	166.6982	S	0.0332	(0.44, 0.21)-(0.60, 0.25)	DataPointRange(14964 - 14966)	0.0332	0.16	4.95	8.64
1236	166.7423	F	0.1658	(0.63, 0.22)		DataPointRange(14967 - 14981)
1237	166.8970	S	0.0227	(0.64, 0.18)-(0.64, 0.25)	DataPointRange(14982 - 14983)	0.0227	0.06	2.53	4.53
1238	166.9302	F	0.1547	(0.63, 0.24)		DataPointRange(14984 - 14997)
1239	167.0738	S	0.0221	(0.63, 0.23)-(0.69, 0.25)	DataPointRange(14998 - 14999)	0.0221	0.06	2.54	3.81
1240	167.1070	F	0.4197	(0.74, 0.26)		DataPointRange(15000 - 15037)
1241	167.5156	S	0.0222	(0.77, 0.28)-(0.82, 0.25)	DataPointRange(15038 - 15039)	0.0222	0.05	2.47	4.88
1242	167.5488	F	0.0443	(0.83, 0.26)		DataPointRange(15040 - 15043)
1243	167.5822	S	0.0770	(0.83, 0.27)-(0.01, -0.42)	DataPointRange(15044 - 15050)	0.0770	0.97	12.55	63.38
1244	167.6702	F	0.0333	(-0.01, -0.44)		DataPointRange(15051 - 15053)
1245	167.6923	S	0.0224	(-0.01, -0.46)-(-0.00, -0.52)	DataPointRange(15054 - 15055)	0.0224	0.05	2.10	2.81
1246	167.7255	F	0.1105	(-0.00, -0.60)		DataPointRange(15056 - 15065)
1247	167.8251	S	0.0221	(-0.01, -0.60)-(0.21, 0.40)	DataPointRange(15066 - 15067)	0.0221	0.78	35.33	68.81
1248	167.8581	F	0.0224	(0.21, 0.39)		DataPointRange(15068 - 15069)
1249	167.8692	S	0.0113	(0.22, 0.37)-(0.20, 0.35)	DataPointRange(15070 - 15070)	0.0113	0.03	2.29	2.29
1250	167.8913	F	0.0552	(0.17, 0.37)		DataPointRange(15071 - 15075)
1251	167.9354	S	0.0111	(0.17, 0.39)-(0.18, 0.36)	DataPointRange(15076 - 15076)	0.0111	0.03	2.63	2.63
1252	167.9575	F	0.1106	(0.19, 0.34)		DataPointRange(15077 - 15086)
1253	168.0569	S	0.0112	(0.18, 0.32)-(0.16, 0.34)	DataPointRange(15087 - 15087)	0.0112	0.02	2.20	2.20
1254	168.0791	F	0.0111	(0.13, 0.39)		DataPointRange(15088 - 15088)
1255	168.0791	S	0.0111	(0.13, 0.39)-(0.18, 0.35)	DataPointRange(15089 - 15089)	0.0111	0.05	4.85	4.85
1256	168.1012	F	0.0329	(0.18, 0.33)		DataPointRange(15090 - 15092)
1257	168.1230	S	0.0222	(0.18, 0.33)-(0.27, 0.28)	DataPointRange(15093 - 15094)	0.0222	0.10	4.61	9.85
1258	168.1564	F	0.0329	(0.27, 0.29)		DataPointRange(15095 - 15097)
1259	168.1783	S	0.0110	(0.27, 0.28)-(0.27, 0.34)	DataPointRange(15098 - 15098)	0.0110	0.05	4.14	4.14
1260	168.2003	F	0.1988	(0.26, 0.35)		DataPointRange(15099 - 15116)
1261	168.3881	S	0.0551	(0.27, 0.34)-(0.39, 0.24)	DataPointRange(15117 - 15121)	0.0551	0.14	2.63	6.29
1262	168.4543	F	0.0442	(0.40, 0.25)		DataPointRange(15122 - 15125)
1263	168.4875	S	0.0110	(0.40, 0.25)-(0.39, 0.25)	DataPointRange(15126 - 15126)	0.0110	0.00	0.19	0.19
1264	168.5100	F	0.0107	(0.40, 0.32)		DataPointRange(15127 - 15127)
1265	168.5100	S	0.0107	(0.40, 0.32)-(0.41, 0.26)	DataPointRange(15128 - 15128)	0.0107	0.05	4.69	4.69
1266	168.5317	F	0.0446	(0.41, 0.24)		DataPointRange(15129 - 15132)
1267	168.5649	S	0.0220	(0.40, 0.25)-(0.40, 0.34)	DataPointRange(15133 - 15134)	0.0220	0.06	2.85	7.24
1268	168.5981	F	0.0110	(0.40, 0.34)		DataPointRange(15135 - 15135)
1269	168.5981	S	0.0110	(0.40, 0.34)-(0.40, 0.32)	DataPointRange(15136 - 15136)	0.0110	0.01	1.23	1.23
1270	168.6201	F	0.0222	(0.40, 0.29)		DataPointRange(15137 - 15138)
1271	168.6313	S	0.0110	(0.40, 0.33)-(0.40, 0.23)	DataPointRange(15139 - 15139)	0.0110	0.08	6.84	6.84
1272	168.6533	F	0.0222	(0.40, 0.24)		DataPointRange(15140 - 15141)
1273	168.6644	S	0.0442	(0.40, 0.23)-(0.54, 0.32)	DataPointRange(15142 - 15145)	0.0442	0.15	3.42	7.63
1274	168.7197	F	0.2320	(0.57, 0.34)		DataPointRange(15146 - 15166)
1275	168.9406	S	0.0338	(0.57, 0.33)-(0.73, 0.28)	DataPointRange(15167 - 15169)	0.0338	0.16	4.81	6.49
1276	168.9849	F	0.1656	(0.73, 0.32)		DataPointRange(15170 - 15184)
1277	169.1396	S	0.0109	(0.73, 0.29)-(0.73, 0.30)	DataPointRange(15185 - 15185)	0.0109	0.01	1.14	1.14
1278	169.1613	F	0.0224	(0.74, 0.35)		DataPointRange(15186 - 15187)
1279	169.1730	S	0.0216	(0.73, 0.35)-(0.78, 0.31)	DataPointRange(15188 - 15189)	0.0216	0.05	2.52	4.17
1280	169.2058	F	0.4306	(0.82, 0.34)		DataPointRange(15190 - 15228)
1281	169.6253	S	0.0885	(0.81, 0.37)-(0.22, 0.46)	DataPointRange(15229 - 15236)	0.0885	0.60	6.74	13.60
1282	169.7248	F	0.0994	(0.23, 0.44)		DataPointRange(15237 - 15245)
1283	169.8132	S	0.0110	(0.21, 0.44)-(0.22, 0.45)	DataPointRange(15246 - 15246)	0.0110	0.01	1.02	1.02
1284	169.8357	F	0.0107	(0.17, 0.56)		DataPointRange(15247 - 15247)
1285	169.8357	S	0.0107	(0.17, 0.56)-(0.21, 0.46)	DataPointRange(15248 - 15248)	0.0107	0.09	8.56	8.56
1286	169.8575	F	0.0221	(0.22, 0.47)		DataPointRange(15249 - 15250)
1287	169.8690	S	0.0437	(0.22, 0.46)-(0.19, 0.46)	DataPointRange(15251 - 15254)	0.0437	0.03	0.70	11.90
1288	169.9237	F	0.1768	(0.19, 0.44)		DataPointRange(15255 - 15270)
1289	170.0894	S	0.0332	(0.18, 0.46)-(0.29, 0.44)	DataPointRange(15271 - 15273)	0.0332	0.11	3.34	4.88
1290	170.1339	F	0.2980	(0.31, 0.41)		DataPointRange(15274 - 15300)
1291	170.4209	S	0.0329	(0.31, 0.42)-(0.41, 0.31)	DataPointRange(15301 - 15303)	0.0329	0.13	4.02	5.53
1292	170.4651	F	0.4312	(0.43, 0.29)		DataPointRange(15304 - 15342)
1293	170.8847	S	0.0223	(0.43, 0.29)-(0.42, 0.21)	DataPointRange(15343 - 15344)	0.0223	0.06	2.85	3.43
1294	170.9179	F	0.1215	(0.41, 0.17)		DataPointRange(15345 - 15355)
1295	171.0283	S	0.1989	(0.40, 0.17)-(0.44, 0.14)	DataPointRange(15356 - 15373)	0.1989	0.05	0.24	160.10
1296	171.2382	F	0.0225	(0.47, 0.13)		DataPointRange(15374 - 15375)
1297	171.2493	S	0.0114	(0.47, 0.14)-(0.47, 0.19)	DataPointRange(15376 - 15376)	0.0114	0.04	3.18	3.18
1298	171.2710	F	0.1886	(0.48, 0.21)		DataPointRange(15377 - 15393)
1299	171.4481	S	0.0330	(0.47, 0.21)-(0.34, 0.25)	DataPointRange(15394 - 15396)	0.0330	0.13	4.05	7.42
1300	171.4924	F	0.1764	(0.33, 0.27)		DataPointRange(15397 - 15412)
1301	171.6576	S	0.0443	(0.33, 0.27)-(0.15, 0.21)	DataPointRange(15413 - 15416)	0.0443	0.18	4.15	7.27
1302	171.7129	F	0.1990	(0.15, 0.19)		DataPointRange(15417 - 15434)
1303	171.8345	End					DataPoint(15428)
1304	171.9009	S	0.0222	(0.16, 0.20)-(0.21, 0.16)	DataPointRange(15435 - 15436)	0.0222	0.06	2.51	5.19
1305	171.9342	F	0.4085	(0.24, 0.18)		DataPointRange(15437 - 15473)
1306	172.1881	aa1n13s.bmp				DataPoint(15460)
1307	172.2542	Start					DataPoint(15466)
1308	172.3317	S	0.0225	(0.24, 0.19)-(0.16, 0.13)	DataPointRange(15474 - 15475)	0.0225	0.09	3.85	5.53
1309	172.3649	F	0.1328	(0.16, 0.13)		DataPointRange(15476 - 15487)
1310	172.4867	S	0.0440	(0.16, 0.13)-(0.35, 0.17)	DataPointRange(15488 - 15491)	0.0440	0.18	4.20	6.25
1311	172.5416	F	0.2872	(0.35, 0.17)		DataPointRange(15492 - 15517)
1312	172.8175	S	0.0335	(0.35, 0.17)-(0.27, 0.13)	DataPointRange(15518 - 15520)	0.0335	0.09	2.71	3.76
1313	172.8619	F	0.0332	(0.26, 0.15)		DataPointRange(15521 - 15523)
1314	172.8840	S	0.1658	(0.27, 0.15)-(0.43, 0.15)	DataPointRange(15524 - 15538)	0.1658	0.16	0.97	124.08
1315	173.0608	F	0.0227	(0.44, 0.12)		DataPointRange(15539 - 15540)
1316	173.0719	S	0.0116	(0.43, 0.11)-(0.42, 0.14)	DataPointRange(15541 - 15541)	0.0116	0.03	2.20	2.20
1317	173.0939	F	0.1989	(0.45, 0.17)		DataPointRange(15542 - 15559)
1318	173.2817	S	0.0222	(0.44, 0.16)-(0.50, 0.16)	DataPointRange(15560 - 15561)	0.0222	0.06	2.64	4.82
1319	173.3149	F	0.2431	(0.52, 0.17)		DataPointRange(15562 - 15583)
1320	173.5471	S	0.0440	(0.53, 0.17)-(0.73, 0.15)	DataPointRange(15584 - 15587)	0.0440	0.20	4.54	6.75
1321	173.6021	F	0.2649	(0.72, 0.16)		DataPointRange(15588 - 15611)
1322	173.8560	S	0.0223	(0.71, 0.17)-(0.79, 0.17)	DataPointRange(15612 - 15613)	0.0223	0.08	3.74	6.19
1323	173.8892	F	0.2873	(0.81, 0.14)		DataPointRange(15614 - 15639)
1324	174.1653	S	0.0778	(0.81, 0.14)-(0.17, 0.24)	DataPointRange(15640 - 15646)	0.0778	0.64	8.23	12.00
1325	174.2537	F	0.3202	(0.18, 0.24)		DataPointRange(15647 - 15675)
1326	174.5629	S	0.0331	(0.18, 0.23)-(0.29, 0.24)	DataPointRange(15676 - 15678)	0.0331	0.11	3.28	6.12
1327	174.6072	F	0.1768	(0.31, 0.24)		DataPointRange(15679 - 15694)
1328	174.7733	S	0.0218	(0.31, 0.25)-(0.26, 0.25)	DataPointRange(15695 - 15696)	0.0218	0.05	2.48	4.46
1329	174.8061	F	0.1987	(0.27, 0.24)		DataPointRange(15697 - 15714)
1330	174.9936	S	0.0222	(0.27, 0.24)-(0.34, 0.25)	DataPointRange(15715 - 15716)	0.0222	0.07	3.23	6.24
1331	175.0268	F	0.2874	(0.38, 0.24)		DataPointRange(15717 - 15742)
1332	175.3033	S	0.0220	(0.38, 0.25)-(0.49, 0.25)	DataPointRange(15743 - 15744)	0.0220	0.11	5.22	7.18
1333	175.3364	F	0.2098	(0.50, 0.24)		DataPointRange(15745 - 15763)
1334	175.5352	S	0.0221	(0.49, 0.23)-(0.55, 0.23)	DataPointRange(15764 - 15765)	0.0221	0.06	2.73	5.87
1335	175.5683	F	0.2540	(0.60, 0.23)		DataPointRange(15766 - 15788)
1336	175.8112	S	0.0331	(0.59, 0.24)-(0.74, 0.24)	DataPointRange(15789 - 15791)	0.0331	0.15	4.54	7.48
1337	175.8554	F	0.3979	(0.77, 0.24)		DataPointRange(15792 - 15827)
1338	176.2422	S	0.0773	(0.76, 0.24)-(0.15, 0.38)	DataPointRange(15828 - 15834)	0.0773	0.62	7.98	13.64
1339	176.3306	F	0.2655	(0.15, 0.36)		DataPointRange(15835 - 15858)
1340	176.5846	S	0.0220	(0.15, 0.34)-(0.26, 0.33)	DataPointRange(15859 - 15860)	0.0220	0.11	5.03	8.33
1341	176.6177	F	0.2209	(0.27, 0.34)		DataPointRange(15861 - 15880)
1342	176.8276	S	0.0442	(0.28, 0.32)-(0.49, 0.36)	DataPointRange(15881 - 15884)	0.0442	0.21	4.69	8.29
1343	176.8828	F	0.2429	(0.51, 0.34)		DataPointRange(15885 - 15906)
1344	177.1145	S	0.0333	(0.52, 0.36)-(0.63, 0.34)	DataPointRange(15907 - 15909)	0.0333	0.12	3.54	5.25
1345	177.1590	F	0.3976	(0.63, 0.34)		DataPointRange(15910 - 15945)
1346	177.5454	S	0.0332	(0.63, 0.35)-(0.51, 0.28)	DataPointRange(15946 - 15948)	0.0332	0.13	3.96	7.18
1347	177.5897	F	0.2211	(0.50, 0.26)		DataPointRange(15949 - 15968)
1348	177.7996	S	0.0333	(0.51, 0.24)-(0.35, 0.22)	DataPointRange(15969 - 15971)	0.0333	0.16	4.67	6.73
1349	177.8439	F	0.2981	(0.35, 0.24)		DataPointRange(15972 - 15998)
1350	178.1311	S	0.0884	(0.35, 0.25)-(0.24, 1.03)	DataPointRange(15999 - 16006)	0.0884	0.60	6.76	23.34
1351	178.2303	F	0.0109	(0.27, 1.17)		DataPointRange(16007 - 16007)
1352	178.2303	S	0.0995	(0.27, 1.17)-(0.50, 0.29)	DataPointRange(16008 - 16016)	0.0995	0.70	7.04	26.50
1353	178.3408	F	0.2873	(0.52, 0.28)		DataPointRange(16017 - 16042)
1354	178.6175	S	0.0325	(0.51, 0.29)-(0.64, 0.34)	DataPointRange(16043 - 16045)	0.0325	0.14	4.21	5.22
1355	178.6612	F	0.2100	(0.65, 0.35)		DataPointRange(16046 - 16064)
1356	178.8601	S	0.0559	(0.65, 0.36)-(0.34, 0.24)	DataPointRange(16065 - 16069)	0.0559	0.32	5.81	10.71
1357	178.9264	F	0.1878	(0.34, 0.23)		DataPointRange(16070 - 16086)
1358	179.1030	S	0.0332	(0.34, 0.24)-(0.22, 0.21)	DataPointRange(16087 - 16089)	0.0332	0.11	3.45	7.06
1359	179.1474	F	0.2316	(0.23, 0.19)		DataPointRange(16090 - 16110)
1360	179.2796	End					DataPoint(16102)
1361	179.3680	S	0.0110	(0.23, 0.19)-(0.24, 0.17)	DataPointRange(16111 - 16111)	0.0110	0.01	0.86	0.86
1362	179.3903	F	0.3975	(0.28, 0.17)		DataPointRange(16112 - 16147)
1363	179.6332	t1n9.bmp					DataPoint(16134)
1364	179.6661	Start					DataPoint(16137)
1365	179.7768	S	0.0222	(0.29, 0.19)-(0.18, 0.15)	DataPointRange(16148 - 16149)	0.0222	0.11	4.92	6.58
1366	179.8102	F	0.3864	(0.16, 0.15)		DataPointRange(16150 - 16184)
1367	180.1857	S	0.0223	(0.16, 0.14)-(0.26, 0.14)	DataPointRange(16185 - 16186)	0.0223	0.10	4.31	6.98
1368	180.2188	F	0.1654	(0.27, 0.15)		DataPointRange(16187 - 16201)
1369	180.3731	S	0.0226	(0.27, 0.15)-(0.36, 0.16)	DataPointRange(16202 - 16203)	0.0226	0.09	4.06	5.18
1370	180.4065	F	0.2210	(0.37, 0.16)		DataPointRange(16204 - 16223)
1371	180.6164	S	0.0224	(0.37, 0.16)-(0.44, 0.17)	DataPointRange(16224 - 16225)	0.0224	0.07	3.13	5.21
1372	180.6496	F	0.3203	(0.48, 0.17)		DataPointRange(16226 - 16254)
1373	180.9590	S	0.0331	(0.48, 0.16)-(0.66, 0.17)	DataPointRange(16255 - 16257)	0.0331	0.18	5.39	6.17
1374	181.0035	F	0.2425	(0.67, 0.17)		DataPointRange(16258 - 16279)
1375	181.2349	S	0.0223	(0.66, 0.15)-(0.74, 0.17)	DataPointRange(16280 - 16281)	0.0223	0.08	3.46	3.62
1376	181.2681	F	0.2098	(0.76, 0.17)		DataPointRange(16282 - 16300)
1377	181.4668	S	0.0667	(0.75, 0.16)-(0.21, 0.22)	DataPointRange(16301 - 16306)	0.0667	0.54	8.17	11.71
1378	181.5442	F	0.2652	(0.20, 0.24)		DataPointRange(16307 - 16330)
1379	181.7985	S	0.0221	(0.20, 0.25)-(0.14, 0.28)	DataPointRange(16331 - 16332)	0.0221	0.07	3.21	5.02
1380	181.8316	F	0.1990	(0.14, 0.26)		DataPointRange(16333 - 16350)
1381	182.0192	S	0.0444	(0.14, 0.26)-(0.32, 0.24)	DataPointRange(16351 - 16354)	0.0444	0.19	4.18	6.96
1382	182.0745	F	0.2651	(0.35, 0.26)		DataPointRange(16355 - 16378)
1383	182.3290	S	0.0217	(0.34, 0.26)-(0.45, 0.25)	DataPointRange(16379 - 16380)	0.0217	0.12	5.47	7.20
1384	182.3622	F	0.3307	(0.47, 0.25)		DataPointRange(16381 - 16410)
1385	182.6818	S	0.0331	(0.46, 0.25)-(0.61, 0.27)	DataPointRange(16411 - 16413)	0.0331	0.14	4.29	6.73
1386	182.7263	F	0.3866	(0.63, 0.25)		DataPointRange(16414 - 16448)
1387	183.1018	S	0.0111	(0.64, 0.25)-(0.66, 0.26)	DataPointRange(16449 - 16449)	0.0111	0.02	1.59	1.59
1388	183.1242	F	0.3532	(0.70, 0.25)		DataPointRange(16450 - 16481)
1389	183.4664	S	0.0220	(0.70, 0.26)-(0.60, 0.25)	DataPointRange(16482 - 16483)	0.0220	0.11	4.93	6.27
1390	183.4994	F	0.3203	(0.58, 0.24)		DataPointRange(16484 - 16512)
1391	183.8087	S	0.0332	(0.58, 0.26)-(0.70, 0.23)	DataPointRange(16513 - 16515)	0.0332	0.12	3.60	5.35
1392	183.8529	F	0.3867	(0.70, 0.25)		DataPointRange(16516 - 16550)
1393	184.2286	S	0.0110	(0.70, 0.24)-(0.69, 0.27)	DataPointRange(16551 - 16551)	0.0110	0.02	2.17	2.17
1394	184.2506	F	0.0110	(0.63, 0.12)		DataPointRange(16552 - 16552)
1395	184.2506	S	0.0884	(0.63, 0.12)-(1.87, -0.29)	DataPointRange(16553 - 16560)	0.0884	1.27	14.39	118.86
1396	184.3502	F	0.0332	(1.87, -0.31)		DataPointRange(16561 - 16563)
1397	184.3720	S	0.0994	(1.86, -0.32)-(0.74, 0.26)	DataPointRange(16564 - 16572)	0.0994	1.21	12.15	119.30
1398	184.4829	F	0.2865	(0.73, 0.25)		DataPointRange(16573 - 16598)
1399	184.7584	S	0.0332	(0.72, 0.25)-(0.53, 0.23)	DataPointRange(16599 - 16601)	0.0332	0.19	5.82	8.86
1400	184.8026	F	0.2876	(0.50, 0.22)		DataPointRange(16602 - 16627)
1401	185.0795	S	0.0328	(0.50, 0.21)-(0.38, 0.23)	DataPointRange(16628 - 16630)	0.0328	0.12	3.75	6.43
1402	185.1232	F	0.1215	(0.37, 0.24)		DataPointRange(16631 - 16641)
1403	185.2336	S	0.0444	(0.37, 0.23)-(0.19, 0.20)	DataPointRange(16642 - 16645)	0.0444	0.18	4.03	7.65
1404	185.2889	F	0.5789	(0.19, 0.20)		DataPointRange(16646 - 16696)
1405	185.3552	End					DataPoint(16652)
1406	185.7307	aa1n6.bmp				DataPoint(16686)
1407	185.8629	S	0.0185	(0.20, 0.23)-(0.17, 0.17)	DataPointRange(16697 - 16698)	0.0185	0.05	2.47	3.62
1408	185.8855	F	0.3650	(0.16, 0.16)		DataPointRange(16699 - 16731)
1409	185.8962	Start					DataPoint(16700)
1410	186.2389	S	0.0222	(0.18, 0.16)-(0.25, 0.16)	DataPointRange(16732 - 16733)	0.0222	0.08	3.47	8.10
1411	186.2720	F	0.1878	(0.28, 0.16)		DataPointRange(16734 - 16750)
1412	186.4491	S	0.0329	(0.28, 0.16)-(0.39, 0.17)	DataPointRange(16751 - 16753)	0.0329	0.11	3.27	5.46
1413	186.4929	F	0.2874	(0.40, 0.16)		DataPointRange(16754 - 16779)
1414	186.7693	S	0.0331	(0.40, 0.17)-(0.54, 0.16)	DataPointRange(16780 - 16782)	0.0331	0.14	4.12	7.10
1415	186.8137	F	0.4416	(0.58, 0.16)		DataPointRange(16783 - 16822)
1416	187.2445	S	0.0548	(0.58, 0.15)-(0.20, 0.22)	DataPointRange(16823 - 16827)	0.0548	0.39	7.03	10.72
1417	187.3108	F	0.2983	(0.20, 0.23)		DataPointRange(16828 - 16854)
1418	187.5974	S	0.0117	(0.21, 0.22)-(0.25, 0.22)	DataPointRange(16855 - 16855)	0.0117	0.04	3.55	3.55
1419	187.6198	F	0.2649	(0.30, 0.23)		DataPointRange(16856 - 16879)
1420	187.8740	S	0.0439	(0.31, 0.23)-(0.49, 0.26)	DataPointRange(16880 - 16883)	0.0439	0.18	4.07	7.05
1421	187.9291	F	0.2208	(0.50, 0.24)		DataPointRange(16884 - 16903)
1422	188.1387	S	0.0336	(0.50, 0.25)-(0.63, 0.25)	DataPointRange(16904 - 16906)	0.0336	0.14	4.06	6.41
1423	188.1829	F	0.3426	(0.67, 0.24)		DataPointRange(16907 - 16937)
1424	188.5145	S	0.0331	(0.67, 0.23)-(0.77, 0.23)	DataPointRange(16938 - 16940)	0.0331	0.10	2.90	4.95
1425	188.5587	F	0.2209	(0.78, 0.24)		DataPointRange(16941 - 16960)
1426	188.7690	S	0.0769	(0.78, 0.25)-(0.21, 0.32)	DataPointRange(16961 - 16967)	0.0769	0.57	7.43	12.81
1427	188.8568	F	0.2870	(0.21, 0.32)		DataPointRange(16968 - 16993)
1428	189.1330	S	0.0219	(0.21, 0.32)-(0.16, 0.34)	DataPointRange(16994 - 16995)	0.0219	0.05	2.33	4.69
1429	189.1662	F	0.2651	(0.14, 0.35)		DataPointRange(16996 - 17019)
1430	189.4202	S	0.0221	(0.13, 0.33)-(0.24, 0.33)	DataPointRange(17020 - 17021)	0.0221	0.11	4.94	7.27
1431	189.4533	F	0.1990	(0.30, 0.33)		DataPointRange(17022 - 17039)
1432	189.6412	S	0.0224	(0.30, 0.32)-(0.33, 0.35)	DataPointRange(17040 - 17041)	0.0224	0.04	1.97	2.96
1433	189.6743	F	0.6736	(0.35, 0.34)		DataPointRange(17042 - 17102)
1434	190.3367	S	0.0775	(0.35, 0.35)-(0.20, 0.73)	DataPointRange(17103 - 17109)	0.0775	0.32	4.17	13.49
1435	190.4255	F	0.0108	(0.26, 0.83)		DataPointRange(17110 - 17110)
1436	190.4255	S	0.0551	(0.26, 0.83)-(0.31, 0.34)	DataPointRange(17111 - 17115)	0.0551	0.37	6.72	14.85
1437	190.4919	F	0.3752	(0.35, 0.33)		DataPointRange(17116 - 17149)
1438	190.8562	S	0.0218	(0.34, 0.35)-(0.29, 0.31)	DataPointRange(17150 - 17151)	0.0218	0.06	2.67	4.87
1439	190.8895	F	0.0994	(0.29, 0.30)		DataPointRange(17152 - 17160)
1440	190.9776	S	0.1108	(0.29, 0.32)-(1.86, -0.32)	DataPointRange(17161 - 17170)	0.1108	1.64	14.82	172.14
1441	191.0993	F	0.0109	(1.86, -0.32)		DataPointRange(17171 - 17171)
1442	191.0993	S	0.1105	(1.86, -0.32)-(0.31, 0.28)	DataPointRange(17172 - 17181)	0.1105	1.61	14.60	240.08
1443	191.2212	F	0.0547	(0.34, 0.25)		DataPointRange(17182 - 17186)
1444	191.2649	S	0.0110	(0.33, 0.23)-(0.36, 0.27)	DataPointRange(17187 - 17187)	0.0110	0.04	3.27	3.27
1445	191.2874	F	0.0215	(0.37, 0.31)		DataPointRange(17188 - 17189)
1446	191.2980	S	0.0330	(0.37, 0.31)-(0.27, 0.26)	DataPointRange(17190 - 17192)	0.0330	0.10	3.10	5.03
1447	191.3420	F	0.2874	(0.26, 0.25)		DataPointRange(17193 - 17218)
1448	191.5630	End					DataPoint(17213)
1449	191.6184	S	0.0221	(0.25, 0.26)-(0.24, 0.17)	DataPointRange(17219 - 17220)	0.0221	0.06	2.88	6.43
1450	191.6518	F	0.4304	(0.25, 0.18)		DataPointRange(17221 - 17259)
1451	191.9276	t1n5.bmp					DataPoint(17246)
1452	192.0600	Start					DataPoint(17258)
1453	192.0710	S	0.0222	(0.25, 0.19)-(0.16, 0.15)	DataPointRange(17260 - 17261)	0.0222	0.09	4.09	7.82
1454	192.1043	F	0.1438	(0.17, 0.15)		DataPointRange(17262 - 17274)
1455	192.2370	S	0.0332	(0.16, 0.13)-(0.28, 0.16)	DataPointRange(17275 - 17277)	0.0332	0.12	3.75	4.76
1456	192.2815	F	0.1761	(0.29, 0.16)		DataPointRange(17278 - 17293)
1457	192.4469	S	0.0329	(0.28, 0.17)-(0.40, 0.16)	DataPointRange(17294 - 17296)	0.0329	0.11	3.48	6.14
1458	192.4908	F	0.2102	(0.44, 0.16)		DataPointRange(17297 - 17315)
1459	192.6899	S	0.0331	(0.45, 0.15)-(0.54, 0.18)	DataPointRange(17316 - 17318)	0.0331	0.10	2.92	4.54
1460	192.7340	F	0.1989	(0.54, 0.17)		DataPointRange(17319 - 17336)
1461	192.9219	S	0.0221	(0.55, 0.17)-(0.62, 0.17)	DataPointRange(17337 - 17338)	0.0221	0.08	3.51	4.49
1462	192.9550	F	0.1658	(0.62, 0.16)		DataPointRange(17339 - 17353)
1463	193.1096	S	0.0222	(0.62, 0.17)-(0.71, 0.16)	DataPointRange(17354 - 17355)	0.0222	0.08	3.75	5.17
1464	193.1428	F	0.2651	(0.73, 0.16)		DataPointRange(17356 - 17379)
1465	193.3969	S	0.0222	(0.73, 0.17)-(0.80, 0.16)	DataPointRange(17380 - 17381)	0.0222	0.06	2.85	4.74
1466	193.4299	F	0.1103	(0.81, 0.16)		DataPointRange(17382 - 17391)
1467	193.5291	S	0.0664	(0.81, 0.15)-(0.22, 0.26)	DataPointRange(17392 - 17397)	0.0664	0.60	9.07	12.52
1468	193.6069	F	0.3203	(0.18, 0.25)		DataPointRange(17398 - 17426)
1469	193.9160	S	0.0226	(0.18, 0.25)-(0.27, 0.25)	DataPointRange(17427 - 17428)	0.0226	0.09	4.12	4.96
1470	193.9491	F	0.2763	(0.29, 0.24)		DataPointRange(17429 - 17453)
1471	194.2141	S	0.0334	(0.29, 0.25)-(0.15, 0.25)	DataPointRange(17454 - 17456)	0.0334	0.14	4.25	6.91
1472	194.2584	F	0.1988	(0.14, 0.24)		DataPointRange(17457 - 17474)
1473	194.4462	S	0.0443	(0.14, 0.24)-(0.35, 0.24)	DataPointRange(17475 - 17478)	0.0443	0.21	4.67	8.62
1474	194.5015	F	0.3756	(0.35, 0.24)		DataPointRange(17479 - 17512)
1475	194.8660	S	0.0336	(0.34, 0.24)-(0.48, 0.25)	DataPointRange(17513 - 17515)	0.0336	0.14	4.11	7.01
1476	194.9103	F	0.2211	(0.52, 0.25)		DataPointRange(17516 - 17535)
1477	195.1200	S	0.0332	(0.52, 0.26)-(0.61, 0.24)	DataPointRange(17536 - 17538)	0.0332	0.10	2.87	4.32
1478	195.1640	F	0.1664	(0.64, 0.26)		DataPointRange(17539 - 17553)
1479	195.3189	S	0.0551	(0.65, 0.26)-(0.22, 0.33)	DataPointRange(17554 - 17558)	0.0551	0.43	7.89	12.46
1480	195.3851	F	0.2435	(0.19, 0.36)		DataPointRange(17559 - 17580)
1481	195.6171	S	0.0333	(0.18, 0.37)-(0.28, 0.34)	DataPointRange(17581 - 17583)	0.0333	0.10	3.02	4.77
1482	195.6614	F	0.3092	(0.28, 0.33)		DataPointRange(17584 - 17611)
1483	195.9594	S	0.0444	(0.28, 0.33)-(0.50, 0.34)	DataPointRange(17612 - 17615)	0.0444	0.22	4.89	7.37
1484	196.0149	F	0.2102	(0.51, 0.34)		DataPointRange(17616 - 17634)
1485	196.2138	S	0.0330	(0.52, 0.33)-(0.65, 0.34)	DataPointRange(17635 - 17637)	0.0330	0.13	3.93	6.51
1486	196.2581	F	0.2538	(0.68, 0.34)		DataPointRange(17638 - 17660)
1487	196.5007	S	0.0331	(0.68, 0.34)-(0.78, 0.34)	DataPointRange(17661 - 17663)	0.0331	0.11	3.23	6.01
1488	196.5449	F	0.3423	(0.80, 0.34)		DataPointRange(17664 - 17694)
1489	196.8762	S	0.0663	(0.81, 0.33)-(0.25, 0.41)	DataPointRange(17695 - 17700)	0.0663	0.56	8.41	11.19
1490	196.9537	F	0.1989	(0.22, 0.44)		DataPointRange(17701 - 17718)
1491	197.1416	S	0.0220	(0.23, 0.42)-(0.15, 0.43)	DataPointRange(17719 - 17720)	0.0220	0.07	3.37	6.65
1492	197.1747	F	0.2100	(0.16, 0.45)		DataPointRange(17721 - 17739)
1493	197.3735	S	0.0222	(0.17, 0.46)-(0.27, 0.44)	DataPointRange(17740 - 17741)	0.0222	0.10	4.59	6.28
1494	197.4066	F	0.2874	(0.29, 0.43)		DataPointRange(17742 - 17767)
1495	197.6829	S	0.0111	(0.29, 0.40)-(0.30, 0.42)	DataPointRange(17768 - 17768)	0.0111	0.02	1.68	1.68
1496	197.7051	F	0.0991	(0.30, 0.44)		DataPointRange(17769 - 17777)
1497	197.7934	S	0.0441	(0.29, 0.44)-(0.46, 0.42)	DataPointRange(17778 - 17781)	0.0441	0.17	3.92	5.31
1498	197.8486	F	0.2870	(0.46, 0.43)		DataPointRange(17782 - 17807)
1499	198.1247	S	0.0221	(0.45, 0.42)-(0.51, 0.42)	DataPointRange(17808 - 17809)	0.0221	0.06	2.81	4.39
1500	198.1577	F	0.1989	(0.53, 0.43)		DataPointRange(17810 - 17827)
1501	198.3460	S	0.0216	(0.54, 0.41)-(0.64, 0.42)	DataPointRange(17828 - 17829)	0.0216	0.10	4.77	6.59
1502	198.3785	F	0.3867	(0.70, 0.42)		DataPointRange(17830 - 17864)
1503	198.7542	S	0.0226	(0.69, 0.41)-(0.78, 0.42)	DataPointRange(17865 - 17866)	0.0226	0.09	3.81	4.84
1504	198.7875	F	0.2426	(0.79, 0.42)		DataPointRange(17867 - 17888)
1505	199.0191	S	0.0663	(0.78, 0.42)-(0.24, 0.49)	DataPointRange(17889 - 17894)	0.0663	0.55	8.30	12.24
1506	199.0964	F	0.4312	(0.20, 0.54)		DataPointRange(17895 - 17933)
1507	199.5164	S	0.0226	(0.20, 0.54)-(0.27, 0.52)	DataPointRange(17934 - 17935)	0.0226	0.07	3.08	4.07
1508	199.5496	F	0.1766	(0.30, 0.52)		DataPointRange(17936 - 17951)
1509	199.7152	S	0.0227	(0.30, 0.51)-(0.38, 0.52)	DataPointRange(17952 - 17953)	0.0227	0.08	3.71	4.86
1510	199.7484	F	0.2760	(0.39, 0.50)		DataPointRange(17954 - 17978)
1511	200.0134	S	0.0221	(0.39, 0.52)-(0.44, 0.52)	DataPointRange(17979 - 17980)	0.0221	0.05	2.25	4.13
1512	200.0464	F	0.0224	(0.46, 0.51)		DataPointRange(17981 - 17982)
1513	200.0575	S	0.1215	(0.46, 0.52)-(0.66, -0.06)	DataPointRange(17983 - 17993)	0.1215	0.47	3.89	135.97
1514	200.1901	F	0.0111	(0.67, -0.06)		DataPointRange(17994 - 17994)
1515	200.1901	S	0.0223	(0.67, -0.06)-(1.86, -0.36)	DataPointRange(17995 - 17996)	0.0223	1.21	54.06	108.01
1516	200.2233	F	0.0446	(1.86, -0.31)		DataPointRange(17997 - 18000)
1517	200.2565	S	0.1326	(1.86, -0.32)-(0.43, 0.36)	DataPointRange(18001 - 18012)	0.1326	1.53	11.53	270.28
1518	200.4005	F	0.0217	(0.42, 0.35)		DataPointRange(18013 - 18014)
1519	200.4111	S	0.0111	(0.43, 0.35)-(0.44, 0.36)	DataPointRange(18015 - 18015)	0.0111	0.01	0.95	0.95
1520	200.4337	F	0.2867	(0.44, 0.41)		DataPointRange(18016 - 18041)
1521	200.7093	S	0.0222	(0.46, 0.44)-(0.51, 0.35)	DataPointRange(18042 - 18043)	0.0222	0.08	3.73	4.95
1522	200.7427	F	0.2319	(0.52, 0.36)		DataPointRange(18044 - 18064)
1523	200.9637	S	0.0330	(0.53, 0.37)-(0.62, 0.33)	DataPointRange(18065 - 18067)	0.0330	0.09	2.74	4.30
1524	201.0076	F	0.2097	(0.64, 0.32)		DataPointRange(18068 - 18086)
1525	201.2062	S	0.0334	(0.63, 0.32)-(0.53, 0.28)	DataPointRange(18087 - 18089)	0.0334	0.10	3.13	6.18
1526	201.2507	F	0.3645	(0.53, 0.29)		DataPointRange(18090 - 18122)
1527	201.6042	S	0.0225	(0.54, 0.30)-(0.47, 0.28)	DataPointRange(18123 - 18124)	0.0225	0.06	2.88	4.87
1528	201.6374	F	0.2099	(0.44, 0.31)		DataPointRange(18125 - 18143)
1529	201.8363	S	0.0992	(0.44, 0.33)-(1.86, -0.32)	DataPointRange(18144 - 18152)	0.0992	1.50	15.14	132.84
1530	201.9465	F	0.0115	(0.95, 1.45)		DataPointRange(18153 - 18153)
1531	201.9465	S	0.0222	(0.95, 1.45)-(1.87, -0.28)	DataPointRange(18154 - 18155)	0.0222	1.59	71.58	138.32
1532	201.9797	F	0.0114	(0.68, -0.07)		DataPointRange(18156 - 18156)
1533	201.9797	S	0.0114	(0.68, -0.07)-(1.87, -0.28)	DataPointRange(18157 - 18157)	0.0114	1.20	105.64	105.64
1534	202.0019	F	0.0331	(1.87, -0.30)		DataPointRange(18158 - 18160)
1535	202.0242	S	0.1215	(1.87, -0.28)-(0.28, 0.23)	DataPointRange(18161 - 18171)	0.1215	1.63	13.41	196.33
1536	202.1569	F	0.1322	(0.29, 0.24)		DataPointRange(18172 - 18183)
1537	202.1898	End					DataPoint(18175)
1538	202.2777	S	0.0222	(0.31, 0.26)-(0.23, 0.21)	DataPointRange(18184 - 18185)	0.0222	0.09	3.93	6.85
1539	202.3108	F	0.3869	(0.19, 0.21)		DataPointRange(18186 - 18220)
1540	202.5210	aa1n17.bmp				DataPoint(18205)
1541	202.5761	Start					DataPoint(18210)
1542	202.6870	S	0.0107	(0.18, 0.20)-(0.18, 0.19)	DataPointRange(18221 - 18221)	0.0107	0.01	0.62	0.62
1543	202.7088	F	0.1548	(0.20, 0.15)		DataPointRange(18222 - 18235)
1544	202.8524	S	0.0221	(0.20, 0.16)-(0.26, 0.16)	DataPointRange(18236 - 18237)	0.0221	0.06	2.85	6.02
1545	202.8860	F	0.2646	(0.29, 0.16)		DataPointRange(18238 - 18261)
1546	203.1397	S	0.0330	(0.30, 0.16)-(0.48, 0.18)	DataPointRange(18262 - 18264)	0.0330	0.17	5.25	7.37
1547	203.1840	F	0.2758	(0.53, 0.17)		DataPointRange(18265 - 18289)
1548	203.4487	S	0.0334	(0.52, 0.17)-(0.66, 0.18)	DataPointRange(18290 - 18292)	0.0334	0.14	4.18	7.86
1549	203.4930	F	0.2320	(0.71, 0.18)		DataPointRange(18293 - 18313)
1550	203.7139	S	0.0335	(0.71, 0.17)-(0.80, 0.16)	DataPointRange(18314 - 18316)	0.0335	0.09	2.74	4.40
1551	203.7583	F	0.2429	(0.82, 0.17)		DataPointRange(18317 - 18338)
1552	203.9902	S	0.0662	(0.82, 0.17)-(0.23, 0.25)	DataPointRange(18339 - 18344)	0.0662	0.60	9.08	13.29
1553	204.0673	F	0.1440	(0.21, 0.25)		DataPointRange(18345 - 18357)
1554	204.2001	S	0.0219	(0.21, 0.26)-(0.16, 0.25)	DataPointRange(18358 - 18359)	0.0219	0.05	2.24	4.62
1555	204.2331	F	0.2431	(0.15, 0.26)		DataPointRange(18360 - 18381)
1556	204.4648	S	0.0333	(0.14, 0.28)-(0.32, 0.22)	DataPointRange(18382 - 18384)	0.0333	0.18	5.52	9.57
1557	204.5091	F	0.2541	(0.33, 0.24)		DataPointRange(18385 - 18407)
1558	204.7524	S	0.0441	(0.33, 0.24)-(0.55, 0.22)	DataPointRange(18408 - 18411)	0.0441	0.21	4.82	7.50
1559	204.8076	F	0.3202	(0.56, 0.23)		DataPointRange(18412 - 18440)
1560	205.1169	S	0.0328	(0.56, 0.24)-(0.69, 0.23)	DataPointRange(18441 - 18443)	0.0328	0.13	3.81	6.00
1561	205.1608	F	0.2323	(0.70, 0.24)		DataPointRange(18444 - 18464)
1562	205.3820	S	0.0219	(0.71, 0.23)-(0.75, 0.26)	DataPointRange(18465 - 18466)	0.0219	0.05	2.13	4.68
1563	205.4150	F	0.1215	(0.80, 0.26)		DataPointRange(18467 - 18477)
1564	205.5255	S	0.0661	(0.80, 0.25)-(0.20, 0.33)	DataPointRange(18478 - 18483)	0.0661	0.60	9.08	13.58
1565	205.6032	F	0.1765	(0.21, 0.34)		DataPointRange(18484 - 18499)
1566	205.7688	S	0.0109	(0.21, 0.34)-(0.19, 0.36)	DataPointRange(18500 - 18500)	0.0109	0.03	2.77	2.77
1567	205.7906	F	0.2101	(0.17, 0.36)		DataPointRange(18501 - 18519)
1568	205.9896	S	0.0220	(0.18, 0.37)-(0.26, 0.33)	DataPointRange(18520 - 18521)	0.0220	0.09	3.97	6.51
1569	206.0226	F	0.2984	(0.27, 0.33)		DataPointRange(18522 - 18548)
1570	206.3098	S	0.0221	(0.27, 0.32)-(0.33, 0.33)	DataPointRange(18549 - 18550)	0.0221	0.06	2.64	5.58
1571	206.3431	F	0.1434	(0.36, 0.33)		DataPointRange(18551 - 18563)
1572	206.4757	S	0.0883	(0.36, 0.34)-(0.38, 0.53)	DataPointRange(18564 - 18571)	0.0883	0.15	1.66	9.26
1573	206.5748	F	0.0222	(0.38, 0.55)		DataPointRange(18572 - 18573)
1574	206.5861	S	0.0769	(0.40, 0.56)-(0.41, 0.27)	DataPointRange(18574 - 18580)	0.0769	0.22	2.89	3.93
1575	206.6741	F	0.7625	(0.42, 0.26)		DataPointRange(18581 - 18649)
1576	207.4255	S	0.0222	(0.43, 0.26)-(0.35, 0.23)	DataPointRange(18650 - 18651)	0.0222	0.08	3.65	4.51
1577	207.4592	F	0.3416	(0.32, 0.21)		DataPointRange(18652 - 18682)
1578	207.5139	End					DataPoint(18657)
1579	207.7898	S	0.0221	(0.32, 0.21)-(0.25, 0.19)	DataPointRange(18683 - 18684)	0.0221	0.07	3.05	5.22
1580	207.8233	F	0.2207	(0.26, 0.19)		DataPointRange(18685 - 18704)
1581	207.8672	aa1n16.bmp				DataPoint(18689)
1582	207.9334	Start					DataPoint(18695)
1583	208.0328	S	0.0225	(0.26, 0.18)-(0.16, 0.16)	DataPointRange(18705 - 18706)	0.0225	0.10	4.36	4.95
1584	208.0660	F	0.1438	(0.17, 0.15)		DataPointRange(18707 - 18719)
1585	208.1990	S	0.0223	(0.16, 0.16)-(0.26, 0.16)	DataPointRange(18720 - 18721)	0.0223	0.10	4.45	5.34
1586	208.2319	F	0.2431	(0.26, 0.16)		DataPointRange(18722 - 18743)
1587	208.4638	S	0.0222	(0.26, 0.15)-(0.36, 0.16)	DataPointRange(18744 - 18745)	0.0222	0.10	4.33	5.14
1588	208.4971	F	0.3641	(0.36, 0.17)		DataPointRange(18746 - 18778)
1589	208.8502	S	0.0442	(0.34, 0.17)-(0.53, 0.17)	DataPointRange(18779 - 18782)	0.0442	0.19	4.22	7.20
1590	208.9058	F	0.2734	(0.53, 0.17)		DataPointRange(18783 - 18806)
1591	209.1600	S	0.0329	(0.54, 0.16)-(0.44, 0.16)	DataPointRange(18807 - 18809)	0.0329	0.10	3.01	20.88
1592	209.2039	F	0.2103	(0.41, 0.17)		DataPointRange(18810 - 18828)
1593	209.4028	S	0.0442	(0.41, 0.17)-(0.62, 0.17)	DataPointRange(18829 - 18832)	0.0442	0.22	4.90	6.28
1594	209.4580	F	0.2213	(0.63, 0.17)		DataPointRange(18833 - 18852)
1595	209.6679	S	0.0332	(0.63, 0.17)-(0.76, 0.18)	DataPointRange(18853 - 18855)	0.0332	0.13	3.89	6.78
1596	209.7126	F	0.1431	(0.79, 0.17)		DataPointRange(18856 - 18868)
1597	209.8447	S	0.0770	(0.79, 0.16)-(0.17, 0.25)	DataPointRange(18869 - 18875)	0.0770	0.62	8.02	13.12
1598	209.9327	F	0.2874	(0.17, 0.26)		DataPointRange(18876 - 18901)
1599	210.2092	S	0.0220	(0.16, 0.26)-(0.24, 0.25)	DataPointRange(18902 - 18903)	0.0220	0.08	3.62	4.73
1600	210.2420	F	0.2985	(0.29, 0.26)		DataPointRange(18904 - 18930)
1601	210.5295	S	0.0331	(0.29, 0.25)-(0.45, 0.26)	DataPointRange(18931 - 18933)	0.0331	0.16	4.84	6.85
1602	210.5741	F	0.2757	(0.50, 0.26)		DataPointRange(18934 - 18958)
1603	210.8387	S	0.0221	(0.50, 0.25)-(0.61, 0.25)	DataPointRange(18959 - 18960)	0.0221	0.11	5.08	7.68
1604	210.8724	F	0.3971	(0.65, 0.25)		DataPointRange(18961 - 18995)
1605	211.2586	S	0.0662	(0.64, 0.25)-(0.20, 0.34)	DataPointRange(18996 - 19001)	0.0662	0.44	6.67	11.15
1606	211.3358	F	0.2983	(0.20, 0.35)		DataPointRange(19002 - 19028)
1607	211.6231	S	0.0225	(0.20, 0.35)-(0.27, 0.34)	DataPointRange(19029 - 19030)	0.0225	0.07	3.27	5.90
1608	211.6562	F	0.3093	(0.29, 0.34)		DataPointRange(19031 - 19058)
1609	211.9545	S	0.0221	(0.30, 0.34)-(0.37, 0.33)	DataPointRange(19059 - 19060)	0.0221	0.08	3.56	5.43
1610	211.9875	F	0.3538	(0.40, 0.34)		DataPointRange(19061 - 19092)
1611	212.3299	S	0.0221	(0.39, 0.33)-(0.46, 0.37)	DataPointRange(19093 - 19094)	0.0221	0.08	3.53	8.09
1612	212.3631	F	0.2208	(0.50, 0.34)		DataPointRange(19095 - 19114)
1613	212.5733	S	0.0880	(0.51, 0.34)-(0.66, 0.78)	DataPointRange(19115 - 19122)	0.0880	0.36	4.10	30.36
1614	212.6727	F	0.0108	(0.69, 0.79)		DataPointRange(19123 - 19123)
1615	212.6727	S	0.0661	(0.69, 0.79)-(0.70, 0.40)	DataPointRange(19124 - 19129)	0.0661	0.30	4.47	7.41
1616	212.7498	F	0.0109	(0.69, 0.37)		DataPointRange(19130 - 19130)
1617	212.7498	S	0.0109	(0.69, 0.37)-(0.68, 0.36)	DataPointRange(19131 - 19131)	0.0109	0.01	1.38	1.38
1618	212.7722	F	0.2868	(0.68, 0.34)		DataPointRange(19132 - 19157)
1619	213.0482	S	0.0218	(0.68, 0.34)-(0.75, 0.35)	DataPointRange(19158 - 19159)	0.0218	0.06	2.88	3.40
1620	213.0812	F	0.2097	(0.77, 0.34)		DataPointRange(19160 - 19178)
1621	213.2796	S	0.0665	(0.77, 0.35)-(0.21, 0.44)	DataPointRange(19179 - 19184)	0.0665	0.56	8.39	12.73
1622	213.3571	F	0.2433	(0.22, 0.44)		DataPointRange(19185 - 19206)
1623	213.5894	S	0.0110	(0.22, 0.41)-(0.21, 0.45)	DataPointRange(19207 - 19207)	0.0110	0.03	2.78	2.78
1624	213.6114	F	0.4087	(0.16, 0.45)		DataPointRange(19208 - 19244)
1625	214.0091	S	0.0331	(0.16, 0.46)-(0.24, 0.43)	DataPointRange(19245 - 19247)	0.0331	0.08	2.53	4.24
1626	214.0532	F	0.2760	(0.26, 0.43)		DataPointRange(19248 - 19272)
1627	214.3180	S	0.0332	(0.25, 0.42)-(0.44, 0.43)	DataPointRange(19273 - 19275)	0.0332	0.20	5.99	7.84
1628	214.3626	F	0.2872	(0.49, 0.41)		DataPointRange(19276 - 19301)
1629	214.6387	S	0.0330	(0.50, 0.42)-(0.66, 0.41)	DataPointRange(19302 - 19304)	0.0330	0.17	5.14	7.11
1630	214.6828	F	0.2541	(0.71, 0.42)		DataPointRange(19305 - 19327)
1631	214.9260	S	0.0221	(0.70, 0.41)-(0.76, 0.42)	DataPointRange(19328 - 19329)	0.0221	0.06	2.76	4.93
1632	214.9593	F	0.5850	(0.79, 0.42)		DataPointRange(19330 - 19382)
1633	215.5334	S	0.0331	(0.78, 0.41)-(0.66, 0.39)	DataPointRange(19383 - 19385)	0.0331	0.12	3.62	6.22
1634	215.5775	F	0.3537	(0.63, 0.40)		DataPointRange(19386 - 19417)
1635	215.9206	S	0.0106	(0.64, 0.43)-(0.62, 0.41)	DataPointRange(19418 - 19418)	0.0106	0.02	1.72	1.72
1636	215.9421	F	0.0113	(0.60, 0.33)		DataPointRange(19419 - 19419)
1637	215.9421	S	0.0885	(0.60, 0.33)-(1.87, -0.28)	DataPointRange(19420 - 19427)	0.0885	1.35	15.24	126.03
1638	216.0415	F	0.0114	(1.87, -0.28)		DataPointRange(19428 - 19428)
1639	216.0415	S	0.1658	(1.87, -0.28)-(0.76, 0.33)	DataPointRange(19429 - 19443)	0.1658	1.20	7.22	162.20
1640	216.2187	F	0.0332	(0.78, 0.34)		DataPointRange(19444 - 19446)
1641	216.2405	S	0.0220	(0.78, 0.32)-(0.80, 0.41)	DataPointRange(19447 - 19448)	0.0220	0.07	3.12	5.85
1642	216.2736	F	0.2096	(0.79, 0.41)		DataPointRange(19449 - 19467)
1643	216.4722	S	0.0443	(0.80, 0.40)-(0.59, 0.36)	DataPointRange(19468 - 19471)	0.0443	0.21	4.72	7.47
1644	216.5272	F	0.1992	(0.59, 0.34)		DataPointRange(19472 - 19489)
1645	216.7157	S	0.0440	(0.59, 0.34)-(0.39, 0.30)	DataPointRange(19490 - 19493)	0.0440	0.20	4.52	7.80
1646	216.7708	F	0.1544	(0.39, 0.29)		DataPointRange(19494 - 19507)
1647	216.9141	S	0.0442	(0.40, 0.31)-(0.23, 0.22)	DataPointRange(19508 - 19511)	0.0442	0.18	4.14	5.58
1648	216.9695	F	0.1436	(0.22, 0.22)		DataPointRange(19512 - 19524)
1649	217.1020	S	0.0111	(0.22, 0.23)-(0.22, 0.20)	DataPointRange(19525 - 19525)	0.0111	0.03	2.51	2.51
1650	217.1240	F	0.6297	(0.21, 0.16)		DataPointRange(19526 - 19582)
1651	217.1570	End					DataPoint(19529)
1652	217.4994	aa1n7.bmp				DataPoint(19560)
1653	217.5987	Start					DataPoint(19569)
1654	217.7426	S	0.0221	(0.21, 0.15)-(0.28, 0.16)	DataPointRange(19583 - 19584)	0.0221	0.07	3.15	5.14
1655	217.7758	F	0.1659	(0.29, 0.16)		DataPointRange(19585 - 19599)
1656	217.9305	S	0.0332	(0.28, 0.16)-(0.41, 0.16)	DataPointRange(19600 - 19602)	0.0332	0.13	3.79	6.63
1657	217.9751	F	0.2094	(0.42, 0.17)		DataPointRange(19603 - 19621)
1658	218.1736	S	0.0219	(0.41, 0.17)-(0.49, 0.16)	DataPointRange(19622 - 19623)	0.0219	0.08	3.75	4.56
1659	218.2069	F	0.3200	(0.52, 0.17)		DataPointRange(19624 - 19652)
1660	218.5159	S	0.0443	(0.52, 0.18)-(0.71, 0.17)	DataPointRange(19653 - 19656)	0.0443	0.19	4.37	5.77
1661	218.5712	F	0.3091	(0.74, 0.18)		DataPointRange(19657 - 19684)
1662	218.8692	S	0.0223	(0.74, 0.17)-(0.80, 0.18)	DataPointRange(19685 - 19686)	0.0223	0.06	2.54	4.92
1663	218.9025	F	0.2430	(0.82, 0.17)		DataPointRange(19687 - 19708)
1664	219.1346	S	0.2761	(0.84, 0.17)-(0.14, 0.16)	DataPointRange(19709 - 19733)	0.2761	0.70	2.55	255.46
1665	219.4218	F	0.0113	(0.15, 0.14)		DataPointRange(19734 - 19734)
1666	219.4218	S	0.0218	(0.15, 0.14)-(0.17, 0.24)	DataPointRange(19735 - 19736)	0.0218	0.08	3.56	5.97
1667	219.4548	F	0.2762	(0.17, 0.24)		DataPointRange(19737 - 19761)
1668	219.7200	S	0.0330	(0.15, 0.24)-(0.32, 0.25)	DataPointRange(19762 - 19764)	0.0330	0.17	5.06	7.38
1669	219.7640	F	0.3093	(0.34, 0.24)		DataPointRange(19765 - 19792)
1670	220.0627	S	0.0328	(0.34, 0.24)-(0.45, 0.23)	DataPointRange(19793 - 19795)	0.0328	0.11	3.37	5.62
1671	220.1067	F	0.4195	(0.45, 0.24)		DataPointRange(19796 - 19833)
1672	220.5151	S	0.0111	(0.45, 0.24)-(0.41, 0.23)	DataPointRange(19834 - 19834)	0.0111	0.04	3.51	3.51
1673	220.5371	F	0.1659	(0.40, 0.24)		DataPointRange(19835 - 19849)
1674	220.6923	S	0.0329	(0.40, 0.25)-(0.53, 0.23)	DataPointRange(19850 - 19852)	0.0329	0.13	4.02	8.25
1675	220.7362	F	0.2429	(0.57, 0.24)		DataPointRange(19853 - 19874)
1676	220.9680	S	0.1220	(0.57, 0.25)-(1.86, -0.36)	DataPointRange(19875 - 19885)	0.1220	1.36	11.18	781.35
1677	221.1007	F	0.0109	(1.86, -0.36)		DataPointRange(19886 - 19886)
1678	221.1007	S	0.1325	(1.86, -0.36)-(0.79, 0.27)	DataPointRange(19887 - 19898)	0.1325	1.16	8.79	281.17
1679	221.2444	F	0.2870	(0.77, 0.26)		DataPointRange(19899 - 19924)
1680	221.5204	S	0.0110	(0.77, 0.27)-(0.79, 0.25)	DataPointRange(19925 - 19925)	0.0110	0.03	2.32	2.32
1681	221.5425	F	0.0994	(0.81, 0.26)		DataPointRange(19926 - 19934)
1682	221.6309	S	0.0773	(0.82, 0.27)-(0.16, 0.24)	DataPointRange(19935 - 19941)	0.0773	0.66	8.53	12.55
1683	221.7197	F	0.2976	(0.18, 0.24)		DataPointRange(19942 - 19968)
1684	222.0061	S	0.0333	(0.19, 0.24)-(0.10, 0.36)	DataPointRange(19969 - 19971)	0.0333	0.13	3.86	7.14
1685	222.0505	F	0.1546	(0.12, 0.39)		DataPointRange(19972 - 19985)
1686	222.1942	S	0.0219	(0.11, 0.38)-(0.18, 0.33)	DataPointRange(19986 - 19987)	0.0219	0.08	3.46	5.26
1687	222.2273	F	0.0996	(0.19, 0.35)		DataPointRange(19988 - 19996)
1688	222.3161	S	0.0549	(0.20, 0.34)-(0.23, 0.28)	DataPointRange(19997 - 20001)	0.0549	0.06	1.06	15.04
1689	222.3821	F	0.0110	(0.23, 0.23)		DataPointRange(20002 - 20002)
1690	222.3821	S	0.0553	(0.23, 0.23)-(0.33, 0.33)	DataPointRange(20003 - 20007)	0.0553	0.13	2.35	11.63
1691	222.4482	F	0.2432	(0.36, 0.36)		DataPointRange(20008 - 20029)
1692	222.6804	S	0.0333	(0.37, 0.36)-(0.53, 0.36)	DataPointRange(20030 - 20032)	0.0333	0.17	5.00	7.47
1693	222.7244	F	0.5191	(0.56, 0.36)		DataPointRange(20033 - 20079)
1694	223.2324	S	0.0223	(0.56, 0.34)-(0.60, 0.34)	DataPointRange(20080 - 20081)	0.0223	0.05	2.09	3.67
1695	223.2658	F	0.4754	(0.63, 0.34)		DataPointRange(20082 - 20124)
1696	223.7297	S	0.0332	(0.63, 0.34)-(0.53, 0.31)	DataPointRange(20125 - 20127)	0.0332	0.10	2.96	4.81
1697	223.7739	F	0.0333	(0.53, 0.32)		DataPointRange(20128 - 20130)
1698	223.7959	S	0.2430	(0.52, 0.31)-(0.55, 0.40)	DataPointRange(20131 - 20152)	0.2430	0.07	0.30	198.55
1699	224.0500	F	0.0109	(0.52, 0.34)		DataPointRange(20153 - 20153)
1700	224.0500	S	0.0109	(0.52, 0.34)-(0.56, 0.35)	DataPointRange(20154 - 20154)	0.0109	0.04	3.46	3.46
1701	224.0726	F	0.1429	(0.56, 0.33)		DataPointRange(20155 - 20167)
1702	224.2046	S	0.0773	(0.55, 0.35)-(0.14, 0.33)	DataPointRange(20168 - 20174)	0.0773	0.41	5.28	11.02
1703	224.2930	F	0.2766	(0.14, 0.37)		DataPointRange(20175 - 20199)
1704	224.5582	S	0.0551	(0.14, 0.36)-(0.42, 0.35)	DataPointRange(20200 - 20204)	0.0551	0.28	5.03	8.12
1705	224.6243	F	0.2322	(0.43, 0.34)		DataPointRange(20205 - 20225)
1706	224.8455	S	0.0329	(0.44, 0.34)-(0.30, 0.23)	DataPointRange(20226 - 20228)	0.0329	0.16	4.84	7.24
1707	224.8896	F	0.1877	(0.27, 0.21)		DataPointRange(20229 - 20245)
1708	224.9226	End					DataPoint(20232)
1709	225.0663	S	0.0110	(0.27, 0.23)-(0.24, 0.20)	DataPointRange(20246 - 20246)	0.0110	0.04	3.56	3.56
1710	225.0883	F	0.3424	(0.21, 0.16)		DataPointRange(20247 - 20277)
1711	225.2992	t1n4.bmp					DataPoint(20266)
1712	225.3315	Start					DataPoint(20269)
1713	225.4196	S	0.0222	(0.22, 0.15)-(0.17, 0.14)	DataPointRange(20278 - 20279)	0.0222	0.05	2.03	4.09
1714	225.4527	F	0.1768	(0.15, 0.14)		DataPointRange(20280 - 20295)
1715	225.6185	S	0.0220	(0.16, 0.15)-(0.22, 0.13)	DataPointRange(20296 - 20297)	0.0220	0.06	2.81	5.31
1716	225.6518	F	0.1772	(0.25, 0.16)		DataPointRange(20298 - 20313)
1717	225.8174	S	0.0446	(0.25, 0.18)-(0.42, 0.15)	DataPointRange(20314 - 20317)	0.0446	0.17	3.74	6.49
1718	225.8727	F	0.2099	(0.45, 0.17)		DataPointRange(20318 - 20336)
1719	226.0716	S	0.0220	(0.45, 0.17)-(0.52, 0.16)	DataPointRange(20337 - 20338)	0.0220	0.07	3.11	6.19
1720	226.1048	F	0.3204	(0.56, 0.17)		DataPointRange(20339 - 20367)
1721	226.4136	S	0.0332	(0.55, 0.18)-(0.66, 0.16)	DataPointRange(20368 - 20370)	0.0332	0.10	3.15	5.50
1722	226.4582	F	0.2097	(0.67, 0.16)		DataPointRange(20371 - 20389)
1723	226.6567	S	0.0222	(0.64, 0.18)-(0.75, 0.17)	DataPointRange(20390 - 20391)	0.0222	0.11	5.17	7.48
1724	226.6900	F	0.1988	(0.75, 0.17)		DataPointRange(20392 - 20409)
1725	226.8779	S	0.0663	(0.76, 0.17)-(0.22, 0.23)	DataPointRange(20410 - 20415)	0.0663	0.54	8.16	33.81
1726	226.9557	F	0.1430	(0.21, 0.23)		DataPointRange(20416 - 20428)
1727	227.0878	S	0.0109	(0.22, 0.23)-(0.19, 0.25)	DataPointRange(20429 - 20429)	0.0109	0.03	2.71	2.71
1728	227.1099	F	0.1658	(0.16, 0.25)		DataPointRange(20430 - 20444)
1729	227.2646	S	0.0330	(0.16, 0.26)-(0.28, 0.24)	DataPointRange(20445 - 20447)	0.0330	0.13	3.91	6.29
1730	227.3088	F	0.2426	(0.29, 0.24)		DataPointRange(20448 - 20469)
1731	227.5405	S	0.0221	(0.29, 0.25)-(0.34, 0.25)	DataPointRange(20470 - 20471)	0.0221	0.06	2.60	4.89
1732	227.5736	F	0.1549	(0.36, 0.26)		DataPointRange(20472 - 20485)
1733	227.7176	S	0.0440	(0.37, 0.25)-(0.53, 0.26)	DataPointRange(20486 - 20489)	0.0440	0.17	3.76	8.10
1734	227.7726	F	0.2209	(0.56, 0.27)		DataPointRange(20490 - 20509)
1735	227.9827	S	0.0221	(0.56, 0.26)-(0.64, 0.26)	DataPointRange(20510 - 20511)	0.0221	0.08	3.64	3.96
1736	228.0157	F	0.2652	(0.64, 0.26)		DataPointRange(20512 - 20535)
1737	228.2697	S	0.0552	(0.64, 0.26)-(0.21, 0.32)	DataPointRange(20536 - 20540)	0.0552	0.43	7.79	11.85
1738	228.3361	F	0.2759	(0.18, 0.35)		DataPointRange(20541 - 20565)
1739	228.6009	S	0.0111	(0.19, 0.35)-(0.23, 0.33)	DataPointRange(20566 - 20566)	0.0111	0.04	3.61	3.61
1740	228.6229	F	0.1990	(0.27, 0.34)		DataPointRange(20567 - 20584)
1741	228.8109	S	0.0334	(0.27, 0.35)-(0.42, 0.35)	DataPointRange(20585 - 20587)	0.0334	0.15	4.45	7.31
1742	228.8550	F	0.2548	(0.45, 0.37)		DataPointRange(20588 - 20610)
1743	229.0982	S	0.0444	(0.45, 0.37)-(0.17, 0.45)	DataPointRange(20611 - 20614)	0.0444	0.29	6.49	12.19
1744	229.1533	F	0.2762	(0.16, 0.46)		DataPointRange(20615 - 20639)
1745	229.4184	S	0.0444	(0.16, 0.46)-(0.36, 0.42)	DataPointRange(20640 - 20643)	0.0444	0.21	4.63	7.08
1746	229.4738	F	0.0998	(0.37, 0.42)		DataPointRange(20644 - 20652)
1747	229.5620	S	0.0551	(0.38, 0.42)-(0.78, -0.08)	DataPointRange(20653 - 20657)	0.0551	0.55	10.02	97.89
1748	229.6281	F	0.0221	(0.80, -0.06)		DataPointRange(20658 - 20659)
1749	229.6393	S	0.0219	(0.77, -0.04)-(1.87, -0.31)	DataPointRange(20660 - 20661)	0.0219	1.12	51.08	96.45
1750	229.6726	F	0.0108	(1.87, -0.26)		DataPointRange(20662 - 20662)
1751	229.6726	S	0.0330	(1.87, -0.26)-(1.86, -0.34)	DataPointRange(20663 - 20665)	0.0330	0.06	1.83	190.31
1752	229.7165	F	0.0109	(1.87, -0.25)		DataPointRange(20666 - 20666)
1753	229.7165	S	0.1329	(1.87, -0.25)-(0.46, 0.31)	DataPointRange(20667 - 20678)	0.1329	1.47	11.09	208.86
1754	229.8603	F	0.0331	(0.45, 0.30)		DataPointRange(20679 - 20681)
1755	229.8825	S	0.0109	(0.44, 0.30)-(0.47, 0.34)	DataPointRange(20682 - 20682)	0.0109	0.04	3.26	3.26
1756	229.9046	F	0.2098	(0.46, 0.35)		DataPointRange(20683 - 20701)
1757	230.1034	S	0.0221	(0.47, 0.33)-(0.49, 0.40)	DataPointRange(20702 - 20703)	0.0221	0.06	2.49	3.74
1758	230.1369	F	0.5516	(0.49, 0.41)		DataPointRange(20704 - 20753)
1759	230.6775	S	0.1217	(0.49, 0.42)-(0.68, 1.64)	DataPointRange(20754 - 20764)	0.1217	0.94	7.70	40.66
1760	230.8101	F	0.0887	(0.69, 1.66)		DataPointRange(20765 - 20772)
1761	230.8874	S	0.0114	(0.68, 1.72)-(0.70, 1.66)	DataPointRange(20773 - 20773)	0.0114	0.04	3.90	3.90
1762	230.9096	F	0.0112	(0.67, 1.64)		DataPointRange(20774 - 20774)
1763	230.9096	S	0.0112	(0.67, 1.64)-(0.70, 1.67)	DataPointRange(20775 - 20775)	0.0112	0.03	2.90	2.90
1764	230.9318	F	0.0221	(0.71, 1.69)		DataPointRange(20776 - 20777)
1765	230.9426	S	0.0113	(0.72, 1.71)-(0.68, 1.67)	DataPointRange(20778 - 20778)	0.0113	0.05	4.30	4.30
1766	230.9649	F	0.0111	(0.67, 1.61)		DataPointRange(20779 - 20779)
1767	230.9649	S	0.0111	(0.67, 1.61)-(0.70, 1.67)	DataPointRange(20780 - 20780)	0.0111	0.06	5.08	5.08
1768	230.9869	F	0.3094	(0.70, 1.69)		DataPointRange(20781 - 20808)
1769	231.2853	S	0.0110	(0.69, 1.68)-(0.70, 1.70)	DataPointRange(20809 - 20809)	0.0110	0.01	1.30	1.30
1770	231.3074	F	0.0773	(0.68, 1.68)		DataPointRange(20810 - 20816)
1771	231.3736	S	0.1769	(0.68, 1.70)-(0.37, 0.31)	DataPointRange(20817 - 20832)	0.1769	1.09	6.18	193.72
1772	231.5617	F	0.1101	(0.37, 0.37)		DataPointRange(20833 - 20842)
1773	231.6610	S	0.0219	(0.38, 0.38)-(0.44, 0.42)	DataPointRange(20843 - 20844)	0.0219	0.07	2.99	6.07
1774	231.6942	F	0.1433	(0.48, 0.42)		DataPointRange(20845 - 20857)
1775	231.8266	S	0.0553	(0.48, 0.44)-(0.21, 0.20)	DataPointRange(20858 - 20862)	0.0553	0.32	5.83	9.88
1776	231.8819	End					DataPoint(20862)
1777	231.8929	F	0.3866	(0.22, 0.20)		DataPointRange(20863 - 20897)
1778	232.2686	S	0.0109	(0.22, 0.22)-(0.21, 0.22)	DataPointRange(20898 - 20898)	0.0109	0.01	0.56	0.56
1779	232.2907	F	0.1544	(0.18, 0.18)		DataPointRange(20899 - 20912)
1780	232.4341	S	0.0221	(0.19, 0.19)-(0.22, 0.13)	DataPointRange(20913 - 20914)	0.0221	0.05	2.47	5.56
1781	232.4671	F	0.2101	(0.24, 0.16)		DataPointRange(20915 - 20933)
1782	232.6662	S	0.0331	(0.25, 0.18)-(0.40, 0.16)	DataPointRange(20934 - 20936)	0.0331	0.15	4.63	7.37
1783	232.7105	F	0.2649	(0.44, 0.17)		DataPointRange(20937 - 20960)
1784	232.9644	S	0.0221	(0.43, 0.16)-(0.52, 0.16)	DataPointRange(20961 - 20962)	0.0221	0.08	3.73	4.28
1785	232.9975	F	0.3977	(0.55, 0.16)		DataPointRange(20963 - 20998)
1786	233.3841	S	0.0552	(0.58, 0.15)-(0.24, 0.22)	DataPointRange(20999 - 21003)	0.0552	0.34	6.19	12.35
1787	233.4507	F	0.1432	(0.23, 0.23)		DataPointRange(21004 - 21016)
1788	233.5833	S	0.0218	(0.24, 0.23)-(0.17, 0.26)	DataPointRange(21017 - 21018)	0.0218	0.07	3.12	3.74
1789	233.6160	F	0.1655	(0.15, 0.26)		DataPointRange(21019 - 21033)
1790	233.7264	t1n3.bmp					DataPoint(21029)
1791	233.7594	Start					DataPoint(21032)
1792	233.7704	S	0.0331	(0.15, 0.26)-(0.27, 0.24)	DataPointRange(21034 - 21036)	0.0331	0.12	3.63	6.81
1793	233.8147	F	0.1658	(0.31, 0.24)		DataPointRange(21037 - 21051)
1794	233.9696	S	0.0222	(0.31, 0.23)-(0.41, 0.24)	DataPointRange(21052 - 21053)	0.0222	0.11	4.84	9.06
1795	234.0025	F	0.2431	(0.45, 0.24)		DataPointRange(21054 - 21075)
1796	234.2345	S	0.0222	(0.45, 0.23)-(0.50, 0.25)	DataPointRange(21076 - 21077)	0.0222	0.05	2.12	3.42
1797	234.2678	F	0.2104	(0.52, 0.24)		DataPointRange(21078 - 21096)
1798	234.4666	S	0.0446	(0.52, 0.24)-(0.71, 0.26)	DataPointRange(21097 - 21100)	0.0446	0.19	4.24	6.84
1799	234.5219	F	0.4199	(0.71, 0.24)		DataPointRange(21101 - 21138)
1800	234.9305	S	0.0662	(0.71, 0.25)-(0.24, 0.33)	DataPointRange(21139 - 21144)	0.0662	0.47	7.16	10.89
1801	235.0082	F	0.3422	(0.21, 0.33)		DataPointRange(21145 - 21175)
1802	235.3396	S	0.0327	(0.21, 0.32)-(0.33, 0.33)	DataPointRange(21176 - 21178)	0.0327	0.13	3.94	7.75
1803	235.3833	F	0.1660	(0.36, 0.32)		DataPointRange(21179 - 21193)
1804	235.5381	S	0.0220	(0.36, 0.31)-(0.44, 0.32)	DataPointRange(21194 - 21195)	0.0220	0.08	3.59	6.59
1805	235.5712	F	0.2207	(0.45, 0.32)		DataPointRange(21196 - 21215)
1806	235.7811	S	0.0441	(0.44, 0.34)-(0.63, 0.34)	DataPointRange(21216 - 21219)	0.0441	0.19	4.22	6.39
1807	235.8364	F	0.2210	(0.65, 0.33)		DataPointRange(21220 - 21239)
1808	236.0463	S	0.0222	(0.64, 0.34)-(0.73, 0.31)	DataPointRange(21240 - 21241)	0.0222	0.09	4.13	6.00
1809	236.0794	F	0.3644	(0.75, 0.32)		DataPointRange(21242 - 21274)
1810	236.4328	S	0.0664	(0.74, 0.31)-(0.25, 0.45)	DataPointRange(21275 - 21280)	0.0664	0.50	7.50	11.73
1811	236.5102	F	0.3643	(0.24, 0.43)		DataPointRange(21281 - 21313)
1812	236.8636	S	0.0333	(0.22, 0.42)-(0.39, 0.41)	DataPointRange(21314 - 21316)	0.0333	0.17	5.08	6.89
1813	236.9075	F	0.1108	(0.41, 0.41)		DataPointRange(21317 - 21326)
1814	237.0072	S	0.0220	(0.43, 0.41)-(0.49, 0.34)	DataPointRange(21327 - 21328)	0.0220	0.08	3.42	8.06
1815	237.0402	F	0.0110	(0.38, 0.30)		DataPointRange(21329 - 21329)
1816	237.0402	S	0.1217	(0.38, 0.30)-(0.49, 0.33)	DataPointRange(21330 - 21340)	0.1217	0.11	0.89	9.01
1817	237.1729	F	0.0223	(0.51, 0.30)		DataPointRange(21341 - 21342)
1818	237.1839	S	0.0113	(0.50, 0.28)-(0.54, 0.33)	DataPointRange(21343 - 21343)	0.0113	0.06	5.53	5.53
1819	237.2059	F	0.1549	(0.54, 0.36)		DataPointRange(21344 - 21357)
1820	237.3496	S	0.0222	(0.54, 0.35)-(0.47, 0.39)	DataPointRange(21358 - 21359)	0.0222	0.08	3.50	5.67
1821	237.3828	F	0.2651	(0.45, 0.37)		DataPointRange(21360 - 21383)
1822	237.6367	S	0.0332	(0.46, 0.37)-(0.38, 0.27)	DataPointRange(21384 - 21386)	0.0332	0.11	3.37	6.25
1823	237.6809	F	0.2761	(0.39, 0.26)		DataPointRange(21387 - 21411)
1824	237.9459	S	0.0221	(0.40, 0.26)-(0.43, 0.37)	DataPointRange(21412 - 21413)	0.0221	0.09	4.25	8.02
1825	237.9791	F	0.3980	(0.44, 0.42)		DataPointRange(21414 - 21449)
1826	238.3659	S	0.0333	(0.44, 0.40)-(0.39, 0.27)	DataPointRange(21450 - 21452)	0.0333	0.11	3.32	6.00
1827	238.4101	F	0.0110	(0.38, 0.28)		DataPointRange(21453 - 21453)
1828	238.4101	S	0.0221	(0.38, 0.28)-(0.31, 0.28)	DataPointRange(21454 - 21455)	0.0221	0.07	3.20	3.90
1829	238.4433	F	0.2542	(0.36, 0.26)		DataPointRange(21456 - 21478)
1830	238.6862	S	0.1877	(0.35, 0.22)-(1.86, -0.32)	DataPointRange(21479 - 21495)	0.1877	1.56	8.34	263.81
1831	238.8854	F	0.0107	(1.86, -0.32)		DataPointRange(21496 - 21496)
1832	238.8854	S	0.0880	(1.86, -0.32)-(0.60, 0.33)	DataPointRange(21497 - 21504)	0.0880	1.35	15.39	132.16
1833	238.9849	F	0.0108	(0.57, 0.28)		DataPointRange(21505 - 21505)
1834	238.9849	S	0.0218	(0.57, 0.28)-(0.61, 0.32)	DataPointRange(21506 - 21507)	0.0218	0.05	2.25	3.59
1835	239.0180	F	0.0659	(0.61, 0.36)		DataPointRange(21508 - 21513)
1836	239.0728	S	0.0443	(0.60, 0.35)-(0.41, 0.34)	DataPointRange(21514 - 21517)	0.0443	0.19	4.33	7.91
1837	239.1278	F	0.1770	(0.41, 0.35)		DataPointRange(21518 - 21533)
1838	239.2936	S	0.0333	(0.41, 0.35)-(0.24, 0.22)	DataPointRange(21534 - 21536)	0.0333	0.19	5.82	10.06
1839	239.3380	F	0.5632	(0.27, 0.18)		DataPointRange(21537 - 21587)
1840	239.3932	End					DataPoint(21542)
1841	239.8901	aa1n4s.bmp				DataPoint(21587)
1842	239.8901	S	0.0225	(0.28, 0.18)-(0.19, 0.14)	DataPointRange(21588 - 21589)	0.0225	0.09	4.14	7.42
1843	239.9233	F	0.2763	(0.16, 0.15)		DataPointRange(21590 - 21614)
1844	239.9784	Start					DataPoint(21595)
1845	240.1884	S	0.0442	(0.17, 0.15)-(0.34, 0.16)	DataPointRange(21615 - 21618)	0.0442	0.18	4.05	7.68
1846	240.2439	F	0.3204	(0.38, 0.16)		DataPointRange(21619 - 21647)
1847	240.5530	S	0.0332	(0.38, 0.15)-(0.53, 0.17)	DataPointRange(21648 - 21650)	0.0332	0.15	4.56	6.86
1848	240.5973	F	0.3310	(0.58, 0.16)		DataPointRange(21651 - 21680)
1849	240.9173	S	0.0224	(0.59, 0.17)-(0.66, 0.16)	DataPointRange(21681 - 21682)	0.0224	0.08	3.47	5.21
1850	240.9505	F	0.1657	(0.70, 0.16)		DataPointRange(21683 - 21697)
1851	241.1052	S	0.0664	(0.69, 0.18)-(0.23, 0.22)	DataPointRange(21698 - 21703)	0.0664	0.47	7.02	10.66
1852	241.1824	F	0.1326	(0.20, 0.23)		DataPointRange(21704 - 21715)
1853	241.3042	S	0.0108	(0.20, 0.24)-(0.19, 0.25)	DataPointRange(21716 - 21716)	0.0108	0.01	1.10	1.10
1854	241.3261	F	0.2100	(0.16, 0.25)		DataPointRange(21717 - 21735)
1855	241.5251	S	0.0442	(0.16, 0.25)-(0.40, 0.25)	DataPointRange(21736 - 21739)	0.0442	0.23	5.25	7.63
1856	241.5803	F	0.2982	(0.40, 0.25)		DataPointRange(21740 - 21766)
1857	241.8675	S	0.0222	(0.40, 0.26)-(0.47, 0.24)	DataPointRange(21767 - 21768)	0.0222	0.07	3.04	5.71
1858	241.9009	F	0.2870	(0.49, 0.25)		DataPointRange(21769 - 21794)
1859	242.1769	S	0.0221	(0.48, 0.24)-(0.59, 0.24)	DataPointRange(21795 - 21796)	0.0221	0.10	4.66	5.15
1860	242.2098	F	0.2650	(0.62, 0.25)		DataPointRange(21797 - 21820)
1861	242.4638	S	0.0554	(0.62, 0.25)-(0.26, 0.30)	DataPointRange(21821 - 21825)	0.0554	0.36	6.45	10.11
1862	242.5304	F	0.1214	(0.27, 0.29)		DataPointRange(21826 - 21836)
1863	242.6407	S	0.0332	(0.28, 0.29)-(0.16, 0.32)	DataPointRange(21837 - 21839)	0.0332	0.12	3.64	6.67
1864	242.6850	F	0.2650	(0.16, 0.33)		DataPointRange(21840 - 21863)
1865	242.9390	S	0.0227	(0.15, 0.34)-(0.26, 0.34)	DataPointRange(21864 - 21865)	0.0227	0.10	4.46	5.34
1866	242.9721	F	0.2098	(0.31, 0.32)		DataPointRange(21866 - 21884)
1867	243.1711	S	0.1655	(0.31, 0.28)-(0.49, 0.33)	DataPointRange(21885 - 21899)	0.1655	0.19	1.13	148.72
1868	243.3478	F	0.2430	(0.51, 0.32)		DataPointRange(21900 - 21921)
1869	243.5794	S	0.0222	(0.50, 0.31)-(0.55, 0.33)	DataPointRange(21922 - 21923)	0.0222	0.05	2.33	4.49
1870	243.6127	F	0.3978	(0.59, 0.33)		DataPointRange(21924 - 21959)
1871	243.9993	S	0.0332	(0.58, 0.34)-(0.76, 0.32)	DataPointRange(21960 - 21962)	0.0332	0.17	5.25	7.07
1872	244.0437	F	0.3535	(0.75, 0.34)		DataPointRange(21963 - 21994)
1873	244.3859	S	0.0113	(0.75, 0.32)-(0.78, 0.35)	DataPointRange(21995 - 21995)	0.0113	0.04	3.56	3.56
1874	244.4081	F	0.0663	(0.82, 0.34)		DataPointRange(21996 - 22001)
1875	244.4633	S	0.0884	(0.82, 0.34)-(0.14, 0.44)	DataPointRange(22002 - 22009)	0.0884	0.69	7.80	13.49
1876	244.5625	F	0.2322	(0.14, 0.45)		DataPointRange(22010 - 22030)
1877	244.7835	S	0.0442	(0.15, 0.45)-(0.32, 0.43)	DataPointRange(22031 - 22034)	0.0442	0.18	4.04	6.72
1878	244.8390	F	0.2540	(0.34, 0.43)		DataPointRange(22035 - 22057)
1879	245.0821	S	0.0330	(0.33, 0.43)-(0.52, 0.42)	DataPointRange(22058 - 22060)	0.0330	0.19	5.80	8.65
1880	245.1262	F	0.2429	(0.53, 0.41)		DataPointRange(22061 - 22082)
1881	245.3581	S	0.0442	(0.53, 0.40)-(0.72, 0.44)	DataPointRange(22083 - 22086)	0.0442	0.19	4.24	7.30
1882	245.4134	F	0.2427	(0.73, 0.42)		DataPointRange(22087 - 22108)
1883	245.6451	S	0.0110	(0.74, 0.41)-(0.77, 0.41)	DataPointRange(22109 - 22109)	0.0110	0.03	3.18	3.18
1884	245.6671	F	0.1329	(0.82, 0.42)		DataPointRange(22110 - 22121)
1885	245.7889	S	0.0663	(0.83, 0.42)-(0.22, 0.51)	DataPointRange(22122 - 22127)	0.0663	0.61	9.21	14.27
1886	245.8663	F	0.4754	(0.17, 0.53)		DataPointRange(22128 - 22170)
1887	246.3302	S	0.0220	(0.17, 0.55)-(0.14, 0.61)	DataPointRange(22171 - 22172)	0.0220	0.05	2.45	4.82
1888	246.3633	F	0.1989	(0.16, 0.63)		DataPointRange(22173 - 22190)
1889	246.5511	S	0.0331	(0.17, 0.65)-(0.28, 0.61)	DataPointRange(22191 - 22193)	0.0331	0.12	3.62	7.42
1890	246.5954	F	0.2429	(0.30, 0.62)		DataPointRange(22194 - 22215)
1891	246.8270	S	0.0113	(0.30, 0.64)-(0.26, 0.63)	DataPointRange(22216 - 22216)	0.0113	0.05	4.09	4.09
1892	246.8491	F	0.1549	(0.25, 0.64)		DataPointRange(22217 - 22230)
1893	246.9930	S	0.2208	(0.24, 0.65)-(1.87, -0.29)	DataPointRange(22231 - 22250)	0.2208	1.77	8.03	194.36
1894	247.2250	F	0.0221	(1.86, -0.34)		DataPointRange(22251 - 22252)
1895	247.2364	S	0.0107	(1.86, -0.32)-(1.86, -0.31)	DataPointRange(22253 - 22253)	0.0107	0.00	0.43	0.43
1896	247.2581	F	0.0114	(0.08, -0.06)		DataPointRange(22254 - 22254)
1897	247.2581	S	0.0114	(0.08, -0.06)-(1.87, -0.29)	DataPointRange(22255 - 22255)	0.0114	1.79	157.43	157.43
1898	247.2804	F	0.0554	(1.86, -0.32)		DataPointRange(22256 - 22260)
1899	247.3244	S	0.1326	(1.86, -0.32)-(0.38, 0.63)	DataPointRange(22261 - 22272)	0.1326	1.65	12.46	207.60
1900	247.4682	F	0.0107	(0.34, 0.59)		DataPointRange(22273 - 22273)
1901	247.4682	S	0.0107	(0.34, 0.59)-(0.40, 0.61)	DataPointRange(22274 - 22274)	0.0107	0.06	5.59	5.59
1902	247.4901	F	0.0445	(0.40, 0.59)		DataPointRange(22275 - 22278)
1903	247.5232	S	0.0553	(0.40, 0.59)-(0.27, 0.63)	DataPointRange(22279 - 22283)	0.0553	0.13	2.31	8.03
1904	247.5895	F	0.1546	(0.27, 0.64)		DataPointRange(22284 - 22297)
1905	247.7334	S	0.0219	(0.26, 0.64)-(0.39, 0.61)	DataPointRange(22298 - 22299)	0.0219	0.13	6.01	9.31
1906	247.7664	F	0.2652	(0.42, 0.61)		DataPointRange(22300 - 22323)
1907	248.0202	S	0.0221	(0.41, 0.59)-(0.53, 0.63)	DataPointRange(22324 - 22325)	0.0221	0.12	5.53	6.50
1908	248.0534	F	0.2762	(0.56, 0.59)		DataPointRange(22326 - 22350)
1909	248.3184	S	0.0222	(0.58, 0.60)-(0.49, 0.59)	DataPointRange(22351 - 22352)	0.0222	0.09	4.19	7.09
1910	248.3517	F	0.5522	(0.51, 0.58)		DataPointRange(22353 - 22402)
1911	248.8929	S	0.0772	(0.50, 0.60)-(1.86, -0.32)	DataPointRange(22403 - 22409)	0.0772	1.53	19.76	147.81
1912	248.9811	F	0.0114	(1.86, -0.32)		DataPointRange(22410 - 22410)
1913	248.9811	S	0.0114	(1.86, -0.32)-(1.86, -0.32)	DataPointRange(22411 - 22411)	0.0114	0.00	0.00	0.00
1914	249.0032	F	0.0112	(0.53, -0.07)		DataPointRange(22412 - 22412)
1915	249.0032	S	0.0112	(0.53, -0.07)-(1.86, -0.32)	DataPointRange(22413 - 22413)	0.0112	1.35	120.33	120.33
1916	249.0256	F	0.0108	(-0.23, 0.40)		DataPointRange(22414 - 22414)
1917	249.0256	S	0.0996	(-0.23, 0.40)-(0.48, 0.53)	DataPointRange(22415 - 22423)	0.0996	0.71	7.17	200.51
1918	249.1359	F	0.0111	(0.46, 0.46)		DataPointRange(22424 - 22424)
1919	249.1359	S	0.0111	(0.46, 0.46)-(0.49, 0.57)	DataPointRange(22425 - 22425)	0.0111	0.09	7.77	7.77
1920	249.1582	F	0.1877	(0.50, 0.62)		DataPointRange(22426 - 22442)
1921	249.3348	S	0.0552	(0.50, 0.62)-(0.35, 0.35)	DataPointRange(22443 - 22447)	0.0552	0.26	4.63	8.44
1922	249.3788	End					DataPoint(22446)
1923	249.4009	F	0.2322	(0.36, 0.32)		DataPointRange(22448 - 22468)
1924	249.6220	S	0.0440	(0.36, 0.33)-(0.19, 0.26)	DataPointRange(22469 - 22472)	0.0440	0.17	3.94	6.07
1925	249.6772	F	0.1875	(0.18, 0.23)		DataPointRange(22473 - 22489)
1926	249.7544	aa1n8s.bmp				DataPoint(22480)
1927	249.8647	Start					DataPoint(22490)
1928	249.8552	S	0.0207	(0.17, 0.23)-(0.14, 0.16)	DataPointRange(22490 - 22491)	0.0207	0.06	2.97	4.46
1929	249.8869	F	0.2209	(0.14, 0.13)		DataPointRange(22492 - 22511)
1930	250.0967	S	0.0224	(0.14, 0.14)-(0.22, 0.14)	DataPointRange(22512 - 22513)	0.0224	0.08	3.43	5.29
1931	250.1298	F	0.4867	(0.25, 0.16)		DataPointRange(22514 - 22557)
1932	250.6050	S	0.0331	(0.26, 0.17)-(0.43, 0.18)	DataPointRange(22558 - 22560)	0.0331	0.17	5.02	8.63
1933	250.6492	F	0.3535	(0.45, 0.17)		DataPointRange(22561 - 22592)
1934	250.9916	S	0.0224	(0.46, 0.16)-(0.51, 0.16)	DataPointRange(22593 - 22594)	0.0224	0.06	2.62	4.72
1935	251.0247	F	0.1434	(0.53, 0.17)		DataPointRange(22595 - 22607)
1936	251.1571	S	0.0335	(0.54, 0.18)-(0.65, 0.19)	DataPointRange(22608 - 22610)	0.0335	0.11	3.38	6.42
1937	251.2014	F	0.1546	(0.65, 0.16)		DataPointRange(22611 - 22624)
1938	251.3455	S	0.0327	(0.65, 0.17)-(0.80, 0.17)	DataPointRange(22625 - 22627)	0.0327	0.15	4.47	6.73
1939	251.3895	F	0.1985	(0.82, 0.16)		DataPointRange(22628 - 22645)
1940	251.5774	S	0.0771	(0.82, 0.16)-(0.16, 0.27)	DataPointRange(22646 - 22652)	0.0771	0.67	8.73	12.32
1941	251.6656	F	0.2207	(0.16, 0.27)		DataPointRange(22653 - 22672)
1942	251.8757	S	0.0217	(0.15, 0.28)-(0.22, 0.27)	DataPointRange(22673 - 22674)	0.0217	0.07	3.26	5.44
1943	251.9088	F	0.1323	(0.25, 0.25)		DataPointRange(22675 - 22686)
1944	252.0299	S	0.0222	(0.25, 0.25)-(0.34, 0.24)	DataPointRange(22687 - 22688)	0.0222	0.10	4.42	7.16
1945	252.0630	F	0.3314	(0.35, 0.25)		DataPointRange(22689 - 22718)
1946	252.3834	S	0.0333	(0.36, 0.26)-(0.50, 0.25)	DataPointRange(22719 - 22721)	0.0333	0.14	4.27	6.71
1947	252.4275	F	0.3977	(0.55, 0.25)		DataPointRange(22722 - 22757)
1948	252.8141	S	0.0334	(0.56, 0.25)-(0.67, 0.25)	DataPointRange(22758 - 22760)	0.0334	0.11	3.34	5.03
1949	252.8585	F	0.2652	(0.67, 0.25)		DataPointRange(22761 - 22784)
1950	253.1125	S	0.0225	(0.67, 0.25)-(0.60, 0.25)	DataPointRange(22785 - 22786)	0.0225	0.07	2.93	5.73
1951	253.1457	F	0.1105	(0.60, 0.26)		DataPointRange(22787 - 22796)
1952	253.2451	S	0.0552	(0.60, 0.27)-(0.18, 0.33)	DataPointRange(22797 - 22801)	0.0552	0.42	7.57	11.55
1953	253.3111	F	0.1881	(0.19, 0.31)		DataPointRange(22802 - 22818)
1954	253.4881	S	0.0111	(0.19, 0.31)-(0.16, 0.34)	DataPointRange(22819 - 22819)	0.0111	0.03	2.99	2.99
1955	253.5103	F	0.2320	(0.15, 0.34)		DataPointRange(22820 - 22840)
1956	253.7310	S	0.0221	(0.14, 0.35)-(0.23, 0.35)	DataPointRange(22841 - 22842)	0.0221	0.08	3.72	5.62
1957	253.7645	F	0.4637	(0.23, 0.34)		DataPointRange(22843 - 22884)
1958	254.2171	S	0.0221	(0.23, 0.33)-(0.33, 0.35)	DataPointRange(22885 - 22886)	0.0221	0.10	4.67	9.17
1959	254.2504	F	0.3424	(0.37, 0.34)		DataPointRange(22887 - 22917)
1960	254.5815	S	0.0220	(0.35, 0.33)-(0.42, 0.36)	DataPointRange(22918 - 22919)	0.0220	0.07	3.27	5.45
1961	254.6148	F	0.2539	(0.46, 0.34)		DataPointRange(22920 - 22942)
1962	254.8581	S	0.0106	(0.44, 0.33)-(0.44, 0.35)	DataPointRange(22943 - 22943)	0.0106	0.02	1.73	1.73
1963	254.8799	F	0.1545	(0.40, 0.34)		DataPointRange(22944 - 22957)
1964	255.0236	S	0.0440	(0.39, 0.34)-(0.16, 0.42)	DataPointRange(22958 - 22961)	0.0440	0.24	5.39	8.19
1965	255.0786	F	0.2322	(0.16, 0.43)		DataPointRange(22962 - 22982)
1966	255.2999	S	0.0223	(0.16, 0.42)-(0.20, 0.41)	DataPointRange(22983 - 22984)	0.0223	0.05	2.07	3.99
1967	255.3329	F	0.2870	(0.25, 0.44)		DataPointRange(22985 - 23010)
1968	255.6088	S	0.0221	(0.24, 0.44)-(0.18, 0.44)	DataPointRange(23011 - 23012)	0.0221	0.06	2.89	4.65
1969	255.6419	F	0.3978	(0.17, 0.45)		DataPointRange(23013 - 23048)
1970	256.0287	S	0.0332	(0.17, 0.44)-(0.32, 0.44)	DataPointRange(23049 - 23051)	0.0332	0.15	4.50	6.01
1971	256.0728	F	0.3097	(0.32, 0.42)		DataPointRange(23052 - 23079)
1972	256.3712	S	0.0222	(0.33, 0.41)-(0.42, 0.43)	DataPointRange(23080 - 23081)	0.0222	0.09	4.13	6.93
1973	256.4044	F	0.2537	(0.47, 0.42)		DataPointRange(23082 - 23104)
1974	256.6471	S	0.0221	(0.46, 0.44)-(0.52, 0.41)	DataPointRange(23105 - 23106)	0.0221	0.06	2.74	5.23
1975	256.6804	F	0.1989	(0.53, 0.43)		DataPointRange(23107 - 23124)
1976	256.8682	S	0.0331	(0.52, 0.43)-(0.64, 0.45)	DataPointRange(23125 - 23127)	0.0331	0.12	3.66	6.23
1977	256.9129	F	0.2425	(0.65, 0.43)		DataPointRange(23128 - 23149)
1978	257.1444	S	0.0333	(0.66, 0.43)-(0.78, 0.42)	DataPointRange(23150 - 23152)	0.0333	0.13	3.80	4.90
1979	257.1886	F	0.1105	(0.78, 0.42)		DataPointRange(23153 - 23162)
1980	257.2881	S	0.0773	(0.79, 0.42)-(0.18, 0.54)	DataPointRange(23163 - 23169)	0.0773	0.62	8.02	12.68
1981	257.3769	F	0.3196	(0.18, 0.53)		DataPointRange(23170 - 23198)
1982	257.6854	S	0.0443	(0.18, 0.53)-(0.43, 0.54)	DataPointRange(23199 - 23202)	0.0443	0.25	5.54	8.95
1983	257.7410	F	0.3867	(0.44, 0.52)		DataPointRange(23203 - 23237)
1984	258.1165	S	0.0220	(0.43, 0.51)-(0.52, 0.51)	DataPointRange(23238 - 23239)	0.0220	0.09	4.02	5.43
1985	258.1495	F	0.4751	(0.53, 0.53)		DataPointRange(23240 - 23282)
1986	258.6136	S	0.0551	(0.54, 0.55)-(0.48, 0.59)	DataPointRange(23283 - 23287)	0.0551	0.06	1.14	12.27
1987	258.6797	F	0.0112	(0.51, 0.60)		DataPointRange(23288 - 23288)
1988	258.6797	S	0.0663	(0.51, 0.60)-(0.52, 0.54)	DataPointRange(23289 - 23294)	0.0663	0.05	0.77	9.84
1989	258.7570	F	0.1768	(0.52, 0.57)		DataPointRange(23295 - 23310)
1990	258.9228	S	0.0551	(0.51, 0.59)-(0.54, 1.20)	DataPointRange(23311 - 23315)	0.0551	0.46	8.39	11.28
1991	258.9891	F	0.3203	(0.54, 1.20)		DataPointRange(23316 - 23343)
1992	259.2982	S	0.0223	(0.53, 1.17)-(0.50, 1.31)	DataPointRange(23344 - 23345)	0.0223	0.11	5.03	6.06
1993	259.3314	F	0.5302	(0.52, 1.30)		DataPointRange(23346 - 23393)
1994	259.8505	S	0.0553	(0.53, 1.28)-(0.39, 0.64)	DataPointRange(23394 - 23398)	0.0553	0.49	8.95	17.08
1995	259.9170	F	0.1326	(0.42, 0.66)		DataPointRange(23399 - 23410)
1996	260.0386	S	0.0335	(0.41, 0.67)-(0.49, 0.54)	DataPointRange(23411 - 23413)	0.0335	0.12	3.69	5.11
1997	260.0827	F	0.2211	(0.53, 0.56)		DataPointRange(23414 - 23433)
1998	260.2924	S	0.1768	(0.54, 0.56)-(0.65, 0.73)	DataPointRange(23434 - 23449)	0.1768	0.17	0.95	10.09
1999	260.4803	F	0.1437	(0.67, 0.74)		DataPointRange(23450 - 23462)
2000	260.6128	S	0.0444	(0.68, 0.77)-(0.50, 1.35)	DataPointRange(23463 - 23466)	0.0444	0.46	10.44	14.56
2001	260.6683	F	0.0995	(0.53, 1.41)		DataPointRange(23467 - 23475)
2002	260.7563	S	0.0115	(0.53, 1.44)-(0.54, 1.41)	DataPointRange(23476 - 23476)	0.0115	0.02	1.89	1.89
2003	260.7785	F	0.0332	(0.53, 1.39)		DataPointRange(23477 - 23479)
2004	260.8008	S	0.0109	(0.52, 1.39)-(0.52, 1.38)	DataPointRange(23480 - 23480)	0.0109	0.01	0.83	0.83
2005	260.8226	F	0.0115	(0.53, 1.45)		DataPointRange(23481 - 23481)
2006	260.8226	S	0.0115	(0.53, 1.45)-(0.52, 1.38)	DataPointRange(23482 - 23482)	0.0115	0.06	4.97	4.97
2007	260.8449	F	0.0330	(0.52, 1.36)		DataPointRange(23483 - 23485)
2008	260.8670	S	0.0883	(0.51, 1.33)-(0.16, 0.34)	DataPointRange(23486 - 23493)	0.0883	0.82	9.29	28.69
2009	260.9663	F	0.0108	(0.17, 0.31)		DataPointRange(23494 - 23494)
2010	260.9663	S	0.0108	(0.17, 0.31)-(0.17, 0.31)	DataPointRange(23495 - 23495)	0.0108	0.00	0.20	0.20
2011	260.9883	F	0.5193	(0.19, 0.39)		DataPointRange(23496 - 23542)
2012	261.4971	S	0.0328	(0.19, 0.38)-(0.42, 0.45)	DataPointRange(23543 - 23545)	0.0328	0.24	7.34	12.09
2013	261.5407	F	0.2320	(0.46, 0.48)		DataPointRange(23546 - 23566)
2014	261.7617	S	0.0995	(0.46, 0.47)-(0.60, 1.00)	DataPointRange(23567 - 23575)	0.0995	0.42	4.22	12.48
2015	261.8721	F	0.0220	(0.60, 1.03)		DataPointRange(23576 - 23577)
2016	261.8831	S	0.0333	(0.60, 1.04)-(0.62, 1.14)	DataPointRange(23578 - 23580)	0.0333	0.08	2.47	12.31
2017	261.9273	F	0.0997	(0.63, 1.16)		DataPointRange(23581 - 23589)
2018	262.0157	S	0.0551	(0.62, 1.18)-(0.67, 1.70)	DataPointRange(23590 - 23594)	0.0551	0.40	7.17	15.56
2019	262.0818	F	0.0221	(0.67, 1.68)		DataPointRange(23595 - 23596)
2020	262.0931	S	0.0108	(0.65, 1.66)-(0.67, 1.69)	DataPointRange(23597 - 23597)	0.0108	0.03	2.82	2.82
2021	262.1150	F	0.0221	(0.70, 1.67)		DataPointRange(23598 - 23599)
2022	262.1261	S	0.0110	(0.70, 1.65)-(0.70, 1.71)	DataPointRange(23600 - 23600)	0.0110	0.05	4.17	4.17
2023	262.1482	F	0.3537	(0.68, 1.71)		DataPointRange(23601 - 23632)
2024	262.4910	S	0.0218	(0.67, 1.73)-(0.55, 1.72)	DataPointRange(23633 - 23634)	0.0218	0.12	5.34	7.63
2025	262.5237	F	0.1331	(0.55, 1.73)		DataPointRange(23635 - 23646)
2026	262.6454	S	0.1989	(0.53, 1.69)-(0.50, 0.54)	DataPointRange(23647 - 23664)	0.1989	0.86	4.32	167.37
2027	262.8555	F	0.0330	(0.52, 0.51)		DataPointRange(23665 - 23667)
2028	262.8775	S	0.0110	(0.52, 0.50)-(0.51, 0.46)	DataPointRange(23668 - 23668)	0.0110	0.03	2.73	2.73
2029	262.8995	F	0.0110	(0.52, 0.44)		DataPointRange(23669 - 23669)
2030	262.8995	S	0.0224	(0.52, 0.44)-(0.52, 0.52)	DataPointRange(23670 - 23671)	0.0224	0.06	2.66	3.93
2031	262.9325	F	0.1326	(0.53, 0.51)		DataPointRange(23672 - 23683)
2032	263.0539	S	0.1769	(0.53, 0.49)-(0.61, 1.77)	DataPointRange(23684 - 23699)	0.1769	0.96	5.44	139.90
2033	263.2417	F	0.0778	(0.62, 1.78)		DataPointRange(23700 - 23706)
2034	263.3082	S	0.0113	(0.61, 1.75)-(0.61, 1.80)	DataPointRange(23707 - 23707)	0.0113	0.04	3.51	3.51
2035	263.3303	F	0.1215	(0.60, 1.78)		DataPointRange(23708 - 23718)
2036	263.4408	S	0.0110	(0.61, 1.79)-(0.58, 1.76)	DataPointRange(23719 - 23719)	0.0110	0.04	3.36	3.36
2037	263.4629	F	0.0551	(0.59, 1.77)		DataPointRange(23720 - 23724)
2038	263.5070	S	0.0110	(0.61, 1.79)-(0.58, 1.78)	DataPointRange(23725 - 23725)	0.0110	0.03	3.01	3.01
2039	263.5288	F	0.3317	(0.57, 1.78)		DataPointRange(23726 - 23755)
2040	263.8494	S	0.0111	(0.59, 1.84)-(0.58, 1.79)	DataPointRange(23756 - 23756)	0.0111	0.04	3.63	3.63
2041	263.8717	F	0.0551	(0.57, 1.80)		DataPointRange(23757 - 23761)
2042	263.9160	S	0.0332	(0.57, 1.82)-(0.72, 1.81)	DataPointRange(23762 - 23764)	0.0332	0.15	4.42	8.82
2043	263.9601	F	0.1325	(0.73, 1.80)		DataPointRange(23765 - 23776)
2044	264.0817	S	0.0109	(0.75, 1.81)-(0.71, 1.76)	DataPointRange(23777 - 23777)	0.0109	0.06	5.45	5.45
2045	264.1035	F	0.1212	(0.72, 1.78)		DataPointRange(23778 - 23788)
2046	264.2138	S	0.0220	(0.71, 1.78)-(0.72, 1.86)	DataPointRange(23789 - 23790)	0.0220	0.06	2.92	5.68
2047	264.2468	F	0.0112	(0.73, 1.88)		DataPointRange(23791 - 23791)
2048	264.2468	S	0.0112	(0.73, 1.88)-(0.71, 1.86)	DataPointRange(23792 - 23792)	0.0112	0.03	2.86	2.86
2049	264.2690	F	0.0110	(0.72, 1.80)		DataPointRange(23793 - 23793)
2050	264.2690	S	0.0110	(0.72, 1.80)-(0.73, 1.84)	DataPointRange(23794 - 23794)	0.0110	0.03	2.48	2.48
2051	264.2911	F	0.0110	(0.75, 1.84)		DataPointRange(23795 - 23795)
2052	264.2911	S	0.0110	(0.75, 1.84)-(0.75, 1.88)	DataPointRange(23796 - 23796)	0.0110	0.03	2.38	2.38
2053	264.3133	F	0.0110	(0.72, 1.80)		DataPointRange(23797 - 23797)
2054	264.3133	S	0.0110	(0.72, 1.80)-(0.72, 1.84)	DataPointRange(23798 - 23798)	0.0110	0.03	3.08	3.08
2055	264.3353	F	0.0885	(0.73, 1.87)		DataPointRange(23799 - 23806)
2056	264.4130	S	0.0108	(0.71, 1.88)-(0.73, 1.84)	DataPointRange(23807 - 23807)	0.0108	0.04	3.72	3.72
2057	264.4350	F	0.0108	(0.31, 1.27)		DataPointRange(23808 - 23808)
2058	264.4350	S	0.0108	(0.31, 1.27)-(0.72, 1.89)	DataPointRange(23809 - 23809)	0.0108	0.62	57.43	57.43
2059	264.4571	F	0.0221	(0.71, 1.90)		DataPointRange(23810 - 23811)
2060	264.4682	S	0.0332	(0.71, 1.89)-(0.28, 1.25)	DataPointRange(23812 - 23814)	0.0332	0.65	19.51	58.20
2061	264.5128	F	0.0216	(0.49, 1.55)		DataPointRange(23815 - 23816)
2062	264.5231	S	0.0113	(0.29, 1.25)-(0.74, 1.84)	DataPointRange(23817 - 23817)	0.0113	0.63	55.96	55.96
2063	264.5453	F	0.0113	(0.69, 1.84)		DataPointRange(23818 - 23818)
2064	264.5453	S	0.0113	(0.69, 1.84)-(0.70, 1.85)	DataPointRange(23819 - 23819)	0.0113	0.01	0.59	0.59
2065	264.5676	F	0.1211	(0.70, 1.85)		DataPointRange(23820 - 23830)
2066	264.6778	S	0.0109	(0.67, 1.86)-(0.73, 1.86)	DataPointRange(23831 - 23831)	0.0109	0.06	5.13	5.13
2067	264.7000	F	0.1435	(0.73, 1.85)		DataPointRange(23832 - 23844)
2068	264.8323	S	0.0112	(0.71, 1.84)-(0.73, 1.84)	DataPointRange(23845 - 23845)	0.0112	0.02	1.35	1.35
2069	264.8544	F	0.0223	(0.52, 1.54)		DataPointRange(23846 - 23847)
2070	264.8656	S	0.0220	(0.74, 1.89)-(0.34, 1.19)	DataPointRange(23848 - 23849)	0.0220	0.66	29.78	59.88
2071	264.8987	F	0.0220	(0.55, 1.54)		DataPointRange(23850 - 23851)
2072	264.9100	S	0.0107	(0.34, 1.19)-(0.75, 1.88)	DataPointRange(23852 - 23852)	0.0107	0.66	62.01	62.01
2073	264.9319	F	0.0111	(0.77, 1.88)		DataPointRange(23853 - 23853)
2074	264.9319	S	0.0111	(0.77, 1.88)-(0.74, 1.87)	DataPointRange(23854 - 23854)	0.0111	0.03	3.05	3.05
2075	264.9538	F	0.0226	(0.73, 1.84)		DataPointRange(23855 - 23856)
2076	264.9648	S	0.0116	(0.73, 1.84)-(0.77, 1.83)	DataPointRange(23857 - 23857)	0.0116	0.04	3.46	3.46
2077	264.9869	F	0.0112	(0.75, 1.79)		DataPointRange(23858 - 23858)
2078	264.9869	S	0.0112	(0.75, 1.79)-(0.79, 1.82)	DataPointRange(23859 - 23859)	0.0112	0.04	3.98	3.98
2079	265.0091	F	0.0112	(0.74, 1.86)		DataPointRange(23860 - 23860)
2080	265.0091	S	0.0112	(0.74, 1.86)-(0.77, 1.78)	DataPointRange(23861 - 23861)	0.0112	0.06	5.72	5.72
2081	265.0313	F	0.0219	(0.76, 1.80)		DataPointRange(23862 - 23863)
2082	265.0422	S	0.0110	(0.74, 1.79)-(0.76, 1.85)	DataPointRange(23864 - 23864)	0.0110	0.04	3.89	3.89
2083	265.0645	F	0.2103	(0.75, 1.82)		DataPointRange(23865 - 23883)
2084	265.2633	S	0.0115	(0.76, 1.85)-(0.70, 1.84)	DataPointRange(23884 - 23884)	0.0115	0.06	5.64	5.64
2085	265.2855	F	0.3865	(0.73, 1.84)		DataPointRange(23885 - 23919)
2086	265.6612	S	0.0218	(0.73, 1.85)-(0.65, 1.87)	DataPointRange(23920 - 23921)	0.0218	0.08	3.73	6.59
2087	265.6942	F	0.2210	(0.66, 1.84)		DataPointRange(23922 - 23941)
2088	265.9040	S	0.0112	(0.68, 1.82)-(0.66, 1.87)	DataPointRange(23942 - 23942)	0.0112	0.05	4.11	4.11
2089	265.9262	F	0.0993	(0.66, 1.85)		DataPointRange(23943 - 23951)
2090	266.0146	S	0.0109	(0.65, 1.83)-(0.68, 1.84)	DataPointRange(23952 - 23952)	0.0109	0.02	2.24	2.24
2091	266.0365	F	0.0111	(0.28, 1.14)		DataPointRange(23953 - 23953)
2092	266.0365	S	0.0111	(0.28, 1.14)-(0.65, 1.87)	DataPointRange(23954 - 23954)	0.0111	0.67	60.28	60.28
2093	266.0588	F	0.0220	(0.67, 1.88)		DataPointRange(23955 - 23956)
2094	266.0697	S	0.0331	(0.67, 1.86)-(0.76, 1.73)	DataPointRange(23957 - 23959)	0.0331	0.13	4.02	6.88
2095	266.1139	F	0.0663	(0.77, 1.76)		DataPointRange(23960 - 23965)
2096	266.1693	S	0.0109	(0.77, 1.77)-(0.74, 1.74)	DataPointRange(23966 - 23966)	0.0109	0.04	3.83	3.83
2097	266.1913	F	0.0218	(0.73, 1.73)		DataPointRange(23967 - 23968)
2098	266.2025	S	0.0106	(0.75, 1.72)-(0.72, 1.71)	DataPointRange(23969 - 23969)	0.0106	0.03	2.78	2.78
2099	266.2244	F	0.0110	(0.78, 1.78)		DataPointRange(23970 - 23970)
2100	266.2244	S	0.0110	(0.78, 1.78)-(0.75, 1.73)	DataPointRange(23971 - 23971)	0.0110	0.05	4.24	4.24
2101	266.2463	F	0.0441	(0.76, 1.73)		DataPointRange(23972 - 23975)
2102	266.2793	S	0.0224	(0.75, 1.69)-(0.71, 1.71)	DataPointRange(23976 - 23977)	0.0224	0.04	1.98	6.52
2103	266.3126	F	0.1219	(0.73, 1.70)		DataPointRange(23978 - 23988)
2104	266.4229	S	0.0221	(0.71, 1.70)-(0.76, 1.68)	DataPointRange(23989 - 23990)	0.0221	0.05	2.35	4.44
2105	266.4560	F	0.0664	(0.71, 1.71)		DataPointRange(23991 - 23996)
2106	266.5113	S	0.0111	(0.71, 1.68)-(0.72, 1.69)	DataPointRange(23997 - 23997)	0.0111	0.01	1.05	1.05
2107	266.5334	F	0.0444	(0.73, 1.72)		DataPointRange(23998 - 24001)
2108	266.5666	S	0.0112	(0.70, 1.69)-(0.77, 1.77)	DataPointRange(24002 - 24002)	0.0112	0.09	7.75	7.75
2109	266.5889	F	0.0110	(0.76, 1.71)		DataPointRange(24003 - 24003)
2110	266.5889	S	0.0110	(0.76, 1.71)-(0.77, 1.74)	DataPointRange(24004 - 24004)	0.0110	0.03	2.68	2.68
2111	266.6111	F	0.0885	(0.74, 1.72)		DataPointRange(24005 - 24012)
2112	266.6883	S	0.0553	(0.76, 1.76)-(0.10, 1.88)	DataPointRange(24013 - 24017)	0.0553	0.66	12.01	31.83
2113	266.7546	F	0.0113	(0.10, 1.90)		DataPointRange(24018 - 24018)
2114	266.7546	S	0.0113	(0.10, 1.90)-(0.06, 1.91)	DataPointRange(24019 - 24019)	0.0113	0.03	3.01	3.01
2115	266.7767	F	0.0108	(0.36, 1.87)		DataPointRange(24020 - 24020)
2116	266.7767	S	0.0223	(0.36, 1.87)-(0.30, 1.80)	DataPointRange(24021 - 24022)	0.0223	0.08	3.58	26.27
2117	266.8097	F	0.0111	(0.27, 1.83)		DataPointRange(24023 - 24023)
2118	266.8097	S	0.0226	(0.27, 1.83)-(0.32, 1.85)	DataPointRange(24024 - 24025)	0.0226	0.05	2.20	2.81
2119	266.8427	F	0.0227	(0.33, 1.86)		DataPointRange(24026 - 24027)
2120	266.8541	S	0.0113	(0.31, 1.85)-(0.36, 1.86)	DataPointRange(24028 - 24028)	0.0113	0.05	4.53	4.53
2121	266.8760	F	0.0112	(0.37, 1.87)		DataPointRange(24029 - 24029)
2122	266.8760	S	0.0112	(0.37, 1.87)-(0.38, 1.89)	DataPointRange(24030 - 24030)	0.0112	0.02	2.10	2.10
2123	266.8984	F	0.0107	(0.25, 1.82)		DataPointRange(24031 - 24031)
2124	266.8984	S	0.0107	(0.25, 1.82)-(0.34, 1.89)	DataPointRange(24032 - 24032)	0.0107	0.11	10.44	10.44
2125	266.9202	F	0.1327	(0.36, 1.88)		DataPointRange(24033 - 24044)
2126	267.0419	S	0.0110	(0.34, 1.87)-(0.35, 1.88)	DataPointRange(24045 - 24045)	0.0110	0.01	0.83	0.83
2127	267.0640	F	0.0110	(0.33, 1.81)		DataPointRange(24046 - 24046)
2128	267.0640	S	0.0110	(0.33, 1.81)-(0.39, 1.88)	DataPointRange(24047 - 24047)	0.0110	0.09	7.95	7.95
2129	267.0860	F	0.1216	(0.39, 1.85)		DataPointRange(24048 - 24058)
2130	267.1968	S	0.0993	(0.38, 1.84)-(0.78, 1.71)	DataPointRange(24059 - 24067)	0.0993	0.41	4.13	12.66
2131	267.3068	F	0.1217	(0.76, 1.72)		DataPointRange(24068 - 24078)
2132	267.4174	S	0.0111	(0.79, 1.77)-(0.76, 1.72)	DataPointRange(24079 - 24079)	0.0111	0.04	3.96	3.96
2133	267.4393	F	0.1768	(0.74, 1.71)		DataPointRange(24080 - 24095)
2134	267.6051	S	0.0110	(0.75, 1.72)-(0.72, 1.74)	DataPointRange(24096 - 24096)	0.0110	0.04	3.51	3.51
2135	267.6276	F	0.1434	(0.73, 1.73)		DataPointRange(24097 - 24109)
2136	267.7599	S	0.0330	(0.74, 1.71)-(0.70, 1.75)	DataPointRange(24110 - 24112)	0.0330	0.05	1.46	4.70
2137	267.8041	F	0.0109	(0.70, 1.74)		DataPointRange(24113 - 24113)
2138	267.8041	S	0.0109	(0.70, 1.74)-(0.73, 1.72)	DataPointRange(24114 - 24114)	0.0109	0.03	3.08	3.08
2139	267.8260	F	0.0552	(0.73, 1.74)		DataPointRange(24115 - 24119)
2140	267.8704	S	0.0108	(0.71, 1.71)-(0.74, 1.75)	DataPointRange(24120 - 24120)	0.0108	0.04	3.96	3.96
2141	267.8925	F	0.0109	(0.73, 1.76)		DataPointRange(24121 - 24121)
2142	267.8925	S	0.0109	(0.73, 1.76)-(0.73, 1.75)	DataPointRange(24122 - 24122)	0.0109	0.01	1.21	1.21
2143	267.9143	F	0.0666	(0.74, 1.67)		DataPointRange(24123 - 24128)
2144	267.9696	S	0.0113	(0.73, 1.65)-(0.75, 1.67)	DataPointRange(24129 - 24129)	0.0113	0.02	2.06	2.06
2145	267.9919	F	0.0109	(0.75, 1.72)		DataPointRange(24130 - 24130)
2146	267.9919	S	0.0109	(0.75, 1.72)-(0.75, 1.63)	DataPointRange(24131 - 24131)	0.0109	0.06	5.96	5.96
2147	268.0140	F	0.0108	(0.73, 1.65)		DataPointRange(24132 - 24132)
2148	268.0140	S	0.0218	(0.73, 1.65)-(0.75, 1.73)	DataPointRange(24133 - 24134)	0.0218	0.06	2.85	4.68
2149	268.0469	F	0.0221	(0.75, 1.76)		DataPointRange(24135 - 24136)
2150	268.0581	S	0.1992	(0.73, 1.74)-(0.25, 0.33)	DataPointRange(24137 - 24154)	0.1992	1.16	5.82	187.42
2151	268.2680	F	0.0993	(0.28, 0.32)		DataPointRange(24155 - 24163)
2152	268.3567	S	0.0218	(0.27, 0.33)-(0.30, 0.39)	DataPointRange(24164 - 24165)	0.0218	0.05	2.24	7.20
2153	268.3898	F	0.0992	(0.29, 0.38)		DataPointRange(24166 - 24174)
2154	268.4777	S	0.0333	(0.29, 0.39)-(0.15, 0.19)	DataPointRange(24175 - 24177)	0.0333	0.20	6.03	10.97
2155	268.5224	F	0.2756	(0.15, 0.15)		DataPointRange(24178 - 24202)
2156	268.7870	S	0.0442	(0.14, 0.15)-(0.39, 0.45)	DataPointRange(24203 - 24206)	0.0442	0.33	7.50	9.84
2157	268.8422	F	0.3867	(0.41, 0.47)		DataPointRange(24207 - 24241)
2158	269.2179	S	0.0333	(0.41, 0.45)-(0.56, 0.51)	DataPointRange(24242 - 24244)	0.0333	0.15	4.63	8.67
2159	269.2621	F	0.3207	(0.58, 0.51)		DataPointRange(24245 - 24273)
2160	269.5714	S	0.0221	(0.58, 0.52)-(0.48, 0.50)	DataPointRange(24274 - 24275)	0.0221	0.10	4.37	8.06
2161	269.6045	F	0.1658	(0.46, 0.49)		DataPointRange(24276 - 24290)
2162	269.7589	S	0.0443	(0.46, 0.48)-(0.25, 0.23)	DataPointRange(24291 - 24294)	0.0443	0.28	6.38	10.11
2163	269.8145	F	0.1545	(0.26, 0.20)		DataPointRange(24295 - 24308)
2164	269.9690	End					DataPoint(24309)
2165	269.9580	S	0.0333	(0.26, 0.20)-(0.16, 0.14)	DataPointRange(24309 - 24311)	0.0333	0.11	3.41	4.50
2166	270.0021	F	0.5303	(0.17, 0.15)		DataPointRange(24312 - 24359)
2167	270.3116	t1n3s.bmp				DataPoint(24340)
2168	270.3666	Start					DataPoint(24345)
2169	270.5213	S	0.0111	(0.18, 0.16)-(0.19, 0.16)	DataPointRange(24360 - 24360)	0.0111	0.02	1.37	1.37
2170	270.5434	F	0.1547	(0.24, 0.15)		DataPointRange(24361 - 24374)
2171	270.6871	S	0.0220	(0.24, 0.17)-(0.30, 0.15)	DataPointRange(24375 - 24376)	0.0220	0.06	2.65	5.16
2172	270.7203	F	0.1547	(0.32, 0.16)		DataPointRange(24377 - 24390)
2173	270.8635	S	0.0222	(0.32, 0.16)-(0.42, 0.17)	DataPointRange(24391 - 24392)	0.0222	0.10	4.42	5.58
2174	270.8967	F	0.1659	(0.45, 0.16)		DataPointRange(24393 - 24407)
2175	271.0516	S	0.0332	(0.45, 0.17)-(0.56, 0.15)	DataPointRange(24408 - 24410)	0.0332	0.11	3.45	5.99
2176	271.0958	F	0.1773	(0.58, 0.16)		DataPointRange(24411 - 24426)
2177	271.2615	S	0.0221	(0.58, 0.17)-(0.63, 0.17)	DataPointRange(24427 - 24428)	0.0221	0.05	2.36	3.73
2178	271.2946	F	0.1768	(0.64, 0.16)		DataPointRange(24429 - 24444)
2179	271.4600	S	0.0665	(0.64, 0.15)-(0.17, 0.23)	DataPointRange(24445 - 24450)	0.0665	0.47	7.06	21.58
2180	271.5379	F	0.2537	(0.18, 0.23)		DataPointRange(24451 - 24473)
2181	271.7807	S	0.0223	(0.17, 0.23)-(0.27, 0.21)	DataPointRange(24474 - 24475)	0.0223	0.10	4.42	5.76
2182	271.8138	F	0.2098	(0.28, 0.23)		DataPointRange(24476 - 24494)
2183	272.0125	S	0.0332	(0.28, 0.23)-(0.44, 0.25)	DataPointRange(24495 - 24497)	0.0332	0.17	5.02	8.01
2184	272.0566	F	0.2543	(0.47, 0.24)		DataPointRange(24498 - 24517)
2185	272.2998	S	0.0221	(0.47, 0.23)-(0.55, 0.24)	DataPointRange(24518 - 24519)	0.0221	0.07	3.29	5.02
2186	272.3334	F	0.2094	(0.55, 0.24)		DataPointRange(24520 - 24538)
2187	272.5318	S	0.0330	(0.54, 0.24)-(0.68, 0.25)	DataPointRange(24539 - 24541)	0.0330	0.14	4.26	6.29
2188	272.5760	F	0.3091	(0.70, 0.24)		DataPointRange(24542 - 24569)
2189	272.8740	S	0.0558	(0.70, 0.24)-(0.26, 0.29)	DataPointRange(24570 - 24574)	0.0558	0.45	8.02	12.54
2190	272.9405	F	0.2982	(0.22, 0.30)		DataPointRange(24575 - 24601)
2191	273.2277	S	0.0219	(0.23, 0.31)-(0.28, 0.31)	DataPointRange(24602 - 24603)	0.0219	0.05	2.38	4.94
2192	273.2607	F	0.1988	(0.32, 0.31)		DataPointRange(24604 - 24621)
2193	273.4486	S	0.0221	(0.32, 0.32)-(0.39, 0.32)	DataPointRange(24622 - 24623)	0.0221	0.07	3.26	4.42
2194	273.4817	F	0.1989	(0.40, 0.32)		DataPointRange(24624 - 24641)
2195	273.6696	S	0.0331	(0.39, 0.33)-(0.51, 0.32)	DataPointRange(24642 - 24644)	0.0331	0.12	3.56	5.19
2196	273.7139	F	0.2319	(0.53, 0.32)		DataPointRange(24645 - 24665)
2197	273.9346	S	0.0332	(0.53, 0.32)-(0.63, 0.33)	DataPointRange(24666 - 24668)	0.0332	0.10	3.01	6.53
2198	273.9790	F	0.2316	(0.65, 0.32)		DataPointRange(24669 - 24689)
2199	274.1995	S	0.0221	(0.65, 0.34)-(0.73, 0.32)	DataPointRange(24690 - 24691)	0.0221	0.08	3.74	4.14
2200	274.2327	F	0.3426	(0.74, 0.32)		DataPointRange(24692 - 24722)
2201	274.5644	S	0.0662	(0.73, 0.32)-(0.26, 0.38)	DataPointRange(24723 - 24728)	0.0662	0.47	7.12	10.82
2202	274.6415	F	0.1657	(0.23, 0.39)		DataPointRange(24729 - 24743)
2203	274.7961	S	0.0111	(0.22, 0.38)-(0.21, 0.39)	DataPointRange(24744 - 24744)	0.0111	0.02	1.70	1.70
2204	274.8185	F	0.1766	(0.17, 0.42)		DataPointRange(24745 - 24760)
2205	274.9843	S	0.0333	(0.18, 0.43)-(0.30, 0.42)	DataPointRange(24761 - 24763)	0.0333	0.12	3.58	5.92
2206	275.0283	F	0.2096	(0.33, 0.41)		DataPointRange(24764 - 24782)
2207	275.2270	S	0.0224	(0.33, 0.41)-(0.38, 0.41)	DataPointRange(24783 - 24784)	0.0224	0.06	2.58	4.53
2208	275.2602	F	0.4750	(0.42, 0.40)		DataPointRange(24785 - 24827)
2209	275.7240	S	0.0220	(0.42, 0.39)-(0.33, 0.32)	DataPointRange(24828 - 24829)	0.0220	0.10	4.68	8.21
2210	275.7572	F	0.2649	(0.31, 0.29)		DataPointRange(24830 - 24853)
2211	276.0111	S	0.0332	(0.32, 0.29)-(0.23, 0.21)	DataPointRange(24854 - 24856)	0.0332	0.11	3.38	5.85
2212	276.0553	F	0.1882	(0.21, 0.18)		DataPointRange(24857 - 24873)
2213	276.2322	S	0.0113	(0.20, 0.17)-(0.23, 0.17)	DataPointRange(24874 - 24874)	0.0113	0.03	2.51	2.51
2214	276.2542	F	0.6642	(0.27, 0.16)		DataPointRange(24875 - 24933)
2215	276.5085	End					DataPoint(24898)
2216	276.8507	aa1n1.bmp				DataPoint(24929)
2217	276.8948	S	0.0372	(0.27, 0.17)-(0.22, 0.16)	DataPointRange(24934 - 24935)	0.0372	0.05	1.33	3.92
2218	276.9389	F	0.1991	(0.20, 0.15)		DataPointRange(24936 - 24953)
2219	276.9502	Start					DataPoint(24937)
2220	277.1271	S	0.0223	(0.19, 0.16)-(0.27, 0.14)	DataPointRange(24954 - 24955)	0.0223	0.08	3.59	3.74
2221	277.1603	F	0.2095	(0.27, 0.15)		DataPointRange(24956 - 24974)
2222	277.3586	S	0.0332	(0.28, 0.15)-(0.41, 0.15)	DataPointRange(24975 - 24977)	0.0332	0.13	3.85	6.84
2223	277.4029	F	0.1327	(0.44, 0.16)		DataPointRange(24978 - 24989)
2224	277.5246	S	0.0110	(0.44, 0.16)-(0.44, 0.16)	DataPointRange(24990 - 24990)	0.0110	0.01	0.47	0.47
2225	277.5469	F	0.0108	(0.40, 0.19)		DataPointRange(24991 - 24991)
2226	277.5469	S	0.0108	(0.40, 0.19)-(0.44, 0.16)	DataPointRange(24992 - 24992)	0.0108	0.05	4.17	4.17
2227	277.5687	F	0.0442	(0.44, 0.15)		DataPointRange(24993 - 24996)
2228	277.6018	S	0.0333	(0.44, 0.16)-(0.54, 0.16)	DataPointRange(24997 - 24999)	0.0333	0.10	2.88	4.66
2229	277.6464	F	0.2872	(0.55, 0.17)		DataPointRange(25000 - 25025)
2230	277.9223	S	0.0224	(0.55, 0.15)-(0.60, 0.16)	DataPointRange(25026 - 25027)	0.0224	0.05	2.17	4.03
2231	277.9555	F	0.3865	(0.61, 0.16)		DataPointRange(25028 - 25062)
2232	278.3310	S	0.0110	(0.59, 0.17)-(0.61, 0.14)	DataPointRange(25063 - 25063)	0.0110	0.03	2.30	2.30
2233	278.3531	F	0.0772	(0.65, 0.16)		DataPointRange(25064 - 25070)
2234	278.4194	S	0.0662	(0.65, 0.17)-(0.19, 0.20)	DataPointRange(25071 - 25076)	0.0662	0.46	7.00	11.88
2235	278.4964	F	0.3207	(0.18, 0.22)		DataPointRange(25077 - 25105)
2236	278.8060	S	0.0222	(0.18, 0.21)-(0.25, 0.23)	DataPointRange(25106 - 25107)	0.0222	0.08	3.51	4.73
2237	278.8394	F	0.1764	(0.25, 0.24)		DataPointRange(25108 - 25123)
2238	279.0049	S	0.0331	(0.25, 0.24)-(0.40, 0.25)	DataPointRange(25124 - 25126)	0.0331	0.15	4.57	7.04
2239	279.0491	F	0.2760	(0.44, 0.25)		DataPointRange(25127 - 25151)
2240	279.3139	S	0.0331	(0.44, 0.25)-(0.53, 0.24)	DataPointRange(25152 - 25154)	0.0331	0.10	2.88	3.96
2241	279.3580	F	0.1879	(0.56, 0.24)		DataPointRange(25155 - 25171)
2242	279.5348	S	0.0221	(0.57, 0.23)-(0.65, 0.24)	DataPointRange(25172 - 25173)	0.0221	0.09	4.03	5.97
2243	279.5681	F	0.2320	(0.68, 0.25)		DataPointRange(25174 - 25194)
2244	279.7891	S	0.0663	(0.68, 0.25)-(0.17, 0.31)	DataPointRange(25195 - 25200)	0.0663	0.51	7.72	11.99
2245	279.8666	F	0.2539	(0.18, 0.34)		DataPointRange(25201 - 25223)
2246	280.1095	S	0.0221	(0.19, 0.33)-(0.24, 0.33)	DataPointRange(25224 - 25225)	0.0221	0.06	2.57	4.81
2247	280.1426	F	0.2209	(0.25, 0.34)		DataPointRange(25226 - 25245)
2248	280.3525	S	0.0332	(0.24, 0.32)-(0.37, 0.32)	DataPointRange(25246 - 25248)	0.0332	0.14	4.12	8.00
2249	280.3968	F	0.2318	(0.42, 0.34)		DataPointRange(25249 - 25269)
2250	280.6174	S	0.0331	(0.43, 0.35)-(0.58, 0.32)	DataPointRange(25270 - 25272)	0.0331	0.15	4.66	6.52
2251	280.6617	F	0.6075	(0.58, 0.33)		DataPointRange(25273 - 25327)
2252	281.2581	S	0.0222	(0.58, 0.33)-(0.64, 0.33)	DataPointRange(25328 - 25329)	0.0222	0.07	2.93	4.76
2253	281.2915	F	0.3424	(0.66, 0.33)		DataPointRange(25330 - 25360)
2254	281.6228	S	0.0442	(0.66, 0.32)-(0.44, 0.33)	DataPointRange(25361 - 25364)	0.0442	0.22	5.03	7.65
2255	281.6779	F	0.2431	(0.44, 0.34)		DataPointRange(25365 - 25386)
2256	281.9099	S	0.0550	(0.43, 0.34)-(0.19, 0.17)	DataPointRange(25387 - 25391)	0.0550	0.27	4.99	8.25
2257	281.9321	End					DataPoint(25388)
2258	281.9763	F	0.4969	(0.21, 0.17)		DataPointRange(25392 - 25436)
2259	282.3295	aa1n18.bmp				DataPoint(25424)
2260	282.4071	Start					DataPoint(25431)
2261	282.4622	S	0.0110	(0.21, 0.16)-(0.17, 0.15)	DataPointRange(25437 - 25437)	0.0110	0.04	3.67	3.67
2262	282.4846	F	0.1434	(0.17, 0.14)		DataPointRange(25438 - 25450)
2263	282.6174	S	0.0436	(0.16, 0.13)-(0.35, 0.16)	DataPointRange(25451 - 25454)	0.0436	0.19	4.37	8.34
2264	282.6722	F	0.2096	(0.37, 0.16)		DataPointRange(25455 - 25473)
2265	282.8707	S	0.0445	(0.38, 0.16)-(0.55, 0.16)	DataPointRange(25474 - 25477)	0.0445	0.18	3.93	6.12
2266	282.9260	F	0.2431	(0.56, 0.16)		DataPointRange(25478 - 25499)
2267	283.1580	S	0.0226	(0.56, 0.16)-(0.61, 0.16)	DataPointRange(25500 - 25501)	0.0226	0.05	2.23	3.74
2268	283.1913	F	0.0994	(0.63, 0.17)		DataPointRange(25502 - 25510)
2269	283.2801	S	0.0880	(0.64, 0.19)-(1.86, -0.31)	DataPointRange(25511 - 25518)	0.0880	1.28	14.55	255.67
2270	283.3794	F	0.0107	(1.87, -0.24)		DataPointRange(25519 - 25519)
2271	283.3794	S	0.0771	(1.87, -0.24)-(0.77, 0.18)	DataPointRange(25520 - 25526)	0.0771	1.14	14.76	118.52
2272	283.4674	F	0.1658	(0.79, 0.19)		DataPointRange(25527 - 25541)
2273	283.6223	S	0.0661	(0.80, 0.19)-(0.19, 0.24)	DataPointRange(25542 - 25547)	0.0661	0.61	9.18	12.72
2274	283.6994	F	0.3312	(0.19, 0.25)		DataPointRange(25548 - 25577)
2275	284.0196	S	0.0223	(0.19, 0.24)-(0.30, 0.25)	DataPointRange(25578 - 25579)	0.0223	0.11	4.90	7.56
2276	284.0528	F	0.2652	(0.33, 0.25)		DataPointRange(25580 - 25603)
2277	284.3072	S	0.0218	(0.32, 0.24)-(0.23, 0.26)	DataPointRange(25604 - 25605)	0.0218	0.10	4.45	5.65
2278	284.3401	F	0.1546	(0.24, 0.25)		DataPointRange(25606 - 25619)
2279	284.4836	S	0.0333	(0.24, 0.23)-(0.38, 0.26)	DataPointRange(25620 - 25622)	0.0333	0.14	4.26	5.64
2280	284.5279	F	0.2210	(0.38, 0.26)		DataPointRange(25623 - 25642)
2281	284.7378	S	0.0223	(0.38, 0.26)-(0.47, 0.24)	DataPointRange(25643 - 25644)	0.0223	0.09	4.01	7.52
2282	284.7712	F	0.2756	(0.53, 0.25)		DataPointRange(25645 - 25669)
2283	285.0358	S	0.0336	(0.53, 0.26)-(0.66, 0.25)	DataPointRange(25670 - 25672)	0.0336	0.13	3.93	5.72
2284	285.0802	F	0.3094	(0.67, 0.24)		DataPointRange(25673 - 25700)
2285	285.3783	S	0.0558	(0.68, 0.23)-(0.28, 0.34)	DataPointRange(25701 - 25705)	0.0558	0.41	7.42	11.37
2286	285.4448	F	0.4309	(0.24, 0.33)		DataPointRange(25706 - 25744)
2287	285.8646	S	0.0334	(0.23, 0.33)-(0.33, 0.32)	DataPointRange(25745 - 25747)	0.0334	0.10	3.06	5.21
2288	285.9088	F	0.3644	(0.35, 0.33)		DataPointRange(25748 - 25780)
2289	286.2624	S	0.0441	(0.36, 0.33)-(0.58, 0.35)	DataPointRange(25781 - 25784)	0.0441	0.22	5.06	8.23
2290	286.3175	F	0.2319	(0.59, 0.34)		DataPointRange(25785 - 25805)
2291	286.5383	S	0.0223	(0.59, 0.34)-(0.53, 0.34)	DataPointRange(25806 - 25807)	0.0223	0.07	2.93	4.53
2292	286.5716	F	0.2871	(0.54, 0.33)		DataPointRange(25808 - 25833)
2293	286.8475	S	0.0223	(0.55, 0.33)-(0.61, 0.33)	DataPointRange(25834 - 25835)	0.0223	0.06	2.87	4.71
2294	286.8808	F	0.2870	(0.62, 0.33)		DataPointRange(25836 - 25861)
2295	287.1567	S	0.0331	(0.63, 0.33)-(0.74, 0.34)	DataPointRange(25862 - 25864)	0.0331	0.11	3.44	5.95
2296	287.2009	F	0.3204	(0.78, 0.33)		DataPointRange(25865 - 25893)
2297	287.5104	S	0.0776	(0.78, 0.35)-(0.13, 0.30)	DataPointRange(25894 - 25900)	0.0776	0.65	8.41	31.52
2298	287.5988	F	0.0110	(0.26, 0.25)		DataPointRange(25901 - 25901)
2299	287.5988	S	0.0331	(0.26, 0.25)-(0.24, 0.42)	DataPointRange(25902 - 25904)	0.0331	0.13	3.86	9.65
2300	287.6430	F	0.1986	(0.23, 0.42)		DataPointRange(25905 - 25922)
2301	287.8308	S	0.0223	(0.23, 0.43)-(0.17, 0.45)	DataPointRange(25923 - 25924)	0.0223	0.06	2.58	5.00
2302	287.8639	F	0.2429	(0.15, 0.44)		DataPointRange(25925 - 25946)
2303	288.0959	S	0.0331	(0.15, 0.45)-(0.30, 0.45)	DataPointRange(25947 - 25949)	0.0331	0.15	4.64	6.71
2304	288.1400	F	0.3761	(0.32, 0.42)		DataPointRange(25950 - 25983)
2305	288.5044	S	0.0444	(0.32, 0.43)-(0.51, 0.43)	DataPointRange(25984 - 25987)	0.0444	0.19	4.34	8.31
2306	288.5597	F	0.3207	(0.54, 0.42)		DataPointRange(25988 - 26016)
2307	288.8693	S	0.0329	(0.53, 0.42)-(0.68, 0.41)	DataPointRange(26017 - 26019)	0.0329	0.14	4.29	5.54
2308	288.9134	F	0.2761	(0.69, 0.42)		DataPointRange(26020 - 26044)
2309	289.1784	S	0.0335	(0.70, 0.42)-(0.56, 0.41)	DataPointRange(26045 - 26047)	0.0335	0.13	3.98	6.52
2310	289.2225	F	0.1655	(0.57, 0.42)		DataPointRange(26048 - 26062)
2311	289.3770	S	0.0553	(0.58, 0.42)-(0.18, 0.52)	DataPointRange(26063 - 26067)	0.0553	0.40	7.28	9.86
2312	289.4435	F	0.3093	(0.18, 0.53)		DataPointRange(26068 - 26095)
2313	289.7418	S	0.0332	(0.17, 0.51)-(0.33, 0.53)	DataPointRange(26096 - 26098)	0.0332	0.16	4.82	7.59
2314	289.7860	F	0.2871	(0.33, 0.53)		DataPointRange(26099 - 26124)
2315	290.0620	S	0.0332	(0.34, 0.52)-(0.49, 0.51)	DataPointRange(26125 - 26127)	0.0332	0.15	4.52	7.49
2316	290.1067	F	0.3638	(0.53, 0.53)		DataPointRange(26128 - 26160)
2317	290.4595	S	0.0332	(0.53, 0.53)-(0.70, 0.49)	DataPointRange(26161 - 26163)	0.0332	0.17	5.18	6.22
2318	290.5040	F	0.2655	(0.71, 0.51)		DataPointRange(26164 - 26187)
2319	290.7579	S	0.0222	(0.70, 0.52)-(0.78, 0.50)	DataPointRange(26188 - 26189)	0.0222	0.08	3.64	5.89
2320	290.7912	F	0.4195	(0.81, 0.51)		DataPointRange(26190 - 26227)
2321	291.1999	S	0.0662	(0.81, 0.51)-(0.26, 0.65)	DataPointRange(26228 - 26233)	0.0662	0.56	8.40	11.99
2322	291.2770	F	0.1327	(0.26, 0.64)		DataPointRange(26234 - 26245)
2323	291.3988	S	0.0219	(0.27, 0.65)-(0.17, 0.65)	DataPointRange(26246 - 26247)	0.0219	0.10	4.49	7.61
2324	291.4320	F	0.1433	(0.15, 0.65)		DataPointRange(26248 - 26260)
2325	291.5646	S	0.1433	(0.14, 0.64)-(0.28, 0.60)	DataPointRange(26261 - 26273)	0.1433	0.14	1.00	23.47
2326	291.7187	F	0.0445	(0.31, 0.56)		DataPointRange(26274 - 26277)
2327	291.7519	S	0.0113	(0.32, 0.54)-(0.30, 0.55)	DataPointRange(26278 - 26278)	0.0113	0.02	1.49	1.49
2328	291.7739	F	0.1218	(0.31, 0.60)		DataPointRange(26279 - 26289)
2329	291.8846	S	0.0220	(0.32, 0.61)-(0.41, 0.58)	DataPointRange(26290 - 26291)	0.0220	0.09	4.07	8.03
2330	291.9178	F	0.3427	(0.45, 0.61)		DataPointRange(26292 - 26322)
2331	292.2492	S	0.0330	(0.44, 0.63)-(0.62, 0.60)	DataPointRange(26323 - 26325)	0.0330	0.18	5.48	7.93
2332	292.2937	F	0.2978	(0.65, 0.62)		DataPointRange(26326 - 26352)
2333	292.5805	S	0.0330	(0.63, 0.61)-(0.77, 0.65)	DataPointRange(26353 - 26355)	0.0330	0.14	4.29	6.35
2334	292.6247	F	0.3426	(0.77, 0.61)		DataPointRange(26356 - 26386)
2335	292.9566	S	0.0107	(0.75, 0.60)-(0.75, 0.62)	DataPointRange(26387 - 26387)	0.0107	0.01	1.20	1.20
2336	292.9783	F	0.1545	(0.76, 0.61)		DataPointRange(26388 - 26401)
2337	293.1218	S	0.0335	(0.76, 0.60)-(0.64, 0.57)	DataPointRange(26402 - 26404)	0.0335	0.13	3.76	5.77
2338	293.1659	F	0.0221	(0.64, 0.59)		DataPointRange(26405 - 26406)
2339	293.1769	S	0.0111	(0.65, 0.60)-(0.63, 0.57)	DataPointRange(26407 - 26407)	0.0111	0.03	2.96	2.96
2340	293.1990	F	0.0223	(0.62, 0.55)		DataPointRange(26408 - 26409)
2341	293.2102	S	0.0220	(0.62, 0.55)-(0.59, 0.50)	DataPointRange(26410 - 26411)	0.0220	0.05	2.26	4.25
2342	293.2433	F	0.0111	(0.57, 0.48)		DataPointRange(26412 - 26412)
2343	293.2433	S	0.0774	(0.57, 0.48)-(0.66, 0.64)	DataPointRange(26413 - 26419)	0.0774	0.15	1.99	11.65
2344	293.3317	F	0.0221	(0.68, 0.57)		DataPointRange(26420 - 26421)
2345	293.3428	S	0.0110	(0.68, 0.58)-(0.67, 0.53)	DataPointRange(26422 - 26422)	0.0110	0.04	3.79	3.79
2346	293.3650	F	0.2430	(0.66, 0.60)		DataPointRange(26423 - 26444)
2347	293.5968	S	0.0332	(0.66, 0.59)-(0.48, 0.48)	DataPointRange(26445 - 26447)	0.0332	0.19	5.79	8.70
2348	293.6412	F	0.1873	(0.47, 0.43)		DataPointRange(26448 - 26464)
2349	293.7404	End					DataPoint(26457)
2350	293.8177	S	0.0440	(0.46, 0.42)-(0.25, 0.27)	DataPointRange(26465 - 26468)	0.0440	0.24	5.35	10.25
2351	293.8729	F	0.2541	(0.26, 0.27)		DataPointRange(26469 - 26491)
2352	294.1160	S	0.0221	(0.26, 0.27)-(0.18, 0.23)	DataPointRange(26492 - 26493)	0.0221	0.09	3.86	7.76
2353	294.1381	t1n1s.bmp				DataPoint(26493)
2354	294.1510	F	0.1305	(0.17, 0.20)		DataPointRange(26494 - 26505)
2355	294.1932	Start					DataPoint(26498)
2356	294.2705	S	0.0110	(0.17, 0.21)-(0.17, 0.19)	DataPointRange(26506 - 26506)	0.0110	0.02	1.43	1.43
2357	294.2926	F	0.1552	(0.16, 0.15)		DataPointRange(26507 - 26520)
2358	294.4363	S	0.0221	(0.16, 0.16)-(0.25, 0.16)	DataPointRange(26521 - 26522)	0.0221	0.09	4.04	5.54
2359	294.4696	F	0.6628	(0.26, 0.15)		DataPointRange(26523 - 26582)
2360	295.1213	S	0.0220	(0.26, 0.15)-(0.18, 0.17)	DataPointRange(26583 - 26584)	0.0220	0.08	3.63	6.97
2361	295.1544	F	0.5631	(0.16, 0.16)		DataPointRange(26585 - 26635)
2362	295.7065	S	0.0337	(0.15, 0.18)-(0.28, 0.15)	DataPointRange(26636 - 26638)	0.0337	0.13	3.75	5.38
2363	295.7510	F	0.2315	(0.30, 0.16)		DataPointRange(26639 - 26659)
2364	295.9715	S	0.0222	(0.30, 0.14)-(0.36, 0.17)	DataPointRange(26660 - 26661)	0.0222	0.07	3.02	5.22
2365	296.0049	F	0.1765	(0.40, 0.17)		DataPointRange(26662 - 26677)
2366	296.1708	S	0.0218	(0.42, 0.17)-(0.49, 0.16)	DataPointRange(26678 - 26679)	0.0218	0.08	3.45	4.74
2367	296.2039	F	0.1986	(0.51, 0.17)		DataPointRange(26680 - 26697)
2368	296.3914	S	0.0223	(0.52, 0.17)-(0.57, 0.16)	DataPointRange(26698 - 26699)	0.0223	0.05	2.02	5.08
2369	296.4246	F	0.2873	(0.59, 0.16)		DataPointRange(26700 - 26725)
2370	296.7010	S	0.0550	(0.57, 0.17)-(0.15, 0.24)	DataPointRange(26726 - 26730)	0.0550	0.43	7.79	12.29
2371	296.7676	F	0.2424	(0.16, 0.25)		DataPointRange(26731 - 26752)
2372	296.9990	S	0.0220	(0.16, 0.27)-(0.25, 0.23)	DataPointRange(26753 - 26754)	0.0220	0.09	4.02	6.45
2373	297.0321	F	0.1877	(0.27, 0.24)		DataPointRange(26755 - 26771)
2374	297.2088	S	0.0223	(0.27, 0.24)-(0.40, 0.24)	DataPointRange(26772 - 26773)	0.0223	0.13	5.96	7.89
2375	297.2420	F	0.2432	(0.44, 0.25)		DataPointRange(26774 - 26795)
2376	297.4742	S	0.0328	(0.43, 0.24)-(0.58, 0.24)	DataPointRange(26796 - 26798)	0.0328	0.15	4.50	7.43
2377	297.5180	F	0.2102	(0.60, 0.24)		DataPointRange(26799 - 26817)
2378	297.7171	S	0.0332	(0.59, 0.24)-(0.75, 0.24)	DataPointRange(26818 - 26820)	0.0332	0.16	4.81	6.00
2379	297.7618	F	0.4414	(0.77, 0.23)		DataPointRange(26821 - 26860)
2380	298.1919	S	0.0667	(0.77, 0.23)-(0.24, 0.33)	DataPointRange(26861 - 26866)	0.0667	0.53	8.01	10.92
2381	298.2692	F	0.1217	(0.22, 0.36)		DataPointRange(26867 - 26877)
2382	298.3800	S	0.0109	(0.22, 0.37)-(0.20, 0.37)	DataPointRange(26878 - 26878)	0.0109	0.02	1.79	1.79
2383	298.4021	F	0.2761	(0.16, 0.36)		DataPointRange(26879 - 26903)
2384	298.6670	S	0.0112	(0.17, 0.36)-(0.17, 0.34)	DataPointRange(26904 - 26904)	0.0112	0.01	1.07	1.07
2385	298.6890	F	0.1661	(0.22, 0.34)		DataPointRange(26905 - 26919)
2386	298.8437	S	0.0333	(0.22, 0.33)-(0.34, 0.33)	DataPointRange(26920 - 26922)	0.0333	0.12	3.68	5.61
2387	298.8878	F	0.2856	(0.37, 0.33)		DataPointRange(26923 - 26947)
2388	299.1535	S	0.0224	(0.39, 0.32)-(0.48, 0.31)	DataPointRange(26948 - 26949)	0.0224	0.09	4.19	32.22
2389	299.1864	F	0.1432	(0.49, 0.32)		DataPointRange(26950 - 26962)
2390	299.3186	S	0.0220	(0.49, 0.32)-(0.56, 0.33)	DataPointRange(26963 - 26964)	0.0220	0.07	3.06	5.07
2391	299.3519	F	0.1879	(0.56, 0.34)		DataPointRange(26965 - 26981)
2392	299.5286	S	0.0442	(0.55, 0.33)-(0.77, 0.32)	DataPointRange(26982 - 26985)	0.0442	0.22	5.03	6.47
2393	299.5843	F	0.2094	(0.77, 0.32)		DataPointRange(26986 - 27004)
2394	299.7830	S	0.0329	(0.76, 0.33)-(0.84, 0.35)	DataPointRange(27005 - 27007)	0.0329	0.08	2.39	3.63
2395	299.8269	F	0.2762	(0.85, 0.33)		DataPointRange(27008 - 27032)
2396	300.0918	S	0.0444	(0.84, 0.31)-(0.59, 0.25)	DataPointRange(27033 - 27036)	0.0444	0.25	5.70	9.14
2397	300.1470	F	0.3756	(0.57, 0.24)		DataPointRange(27037 - 27070)
2398	300.5119	S	0.2322	(0.56, 0.25)-(0.62, 0.30)	DataPointRange(27071 - 27091)	0.2322	0.07	0.28	236.52
2399	300.7548	F	0.0223	(0.61, 0.26)		DataPointRange(27092 - 27093)
2400	300.7657	S	0.0114	(0.60, 0.25)-(0.62, 0.29)	DataPointRange(27094 - 27094)	0.0114	0.04	3.34	3.34
2401	300.7880	F	0.2983	(0.63, 0.31)		DataPointRange(27095 - 27121)
2402	301.0749	S	0.0334	(0.63, 0.31)-(0.50, 0.26)	DataPointRange(27122 - 27124)	0.0334	0.13	3.94	7.56
2403	301.1193	F	0.5302	(0.48, 0.24)		DataPointRange(27125 - 27172)
2404	301.6384	S	0.0441	(0.47, 0.24)-(0.67, 0.33)	DataPointRange(27173 - 27176)	0.0441	0.21	4.87	6.08
2405	301.6937	F	0.1325	(0.66, 0.31)		DataPointRange(27177 - 27188)
2406	301.8150	S	0.0221	(0.66, 0.30)-(0.78, 0.34)	DataPointRange(27189 - 27190)	0.0221	0.12	5.36	7.87
2407	301.8482	F	0.1988	(0.81, 0.33)		DataPointRange(27191 - 27208)
2408	302.0360	S	0.0664	(0.81, 0.32)-(0.24, 0.18)	DataPointRange(27209 - 27214)	0.0664	0.58	8.80	38.36
2409	302.1132	F	0.5412	(0.24, 0.19)		DataPointRange(27215 - 27263)
2410	302.1464	End					DataPoint(27218)
2411	302.4999	aa1n20s.bmp				DataPoint(27250)
2412	302.6213	Start					DataPoint(27261)
2413	302.6435	S	0.0109	(0.23, 0.18)-(0.21, 0.18)	DataPointRange(27264 - 27264)	0.0109	0.01	1.32	1.32
2414	302.6657	F	0.1437	(0.19, 0.16)		DataPointRange(27265 - 27277)
2415	302.7983	S	0.0222	(0.19, 0.17)-(0.27, 0.17)	DataPointRange(27278 - 27279)	0.0222	0.08	3.59	5.84
2416	302.8321	F	0.1980	(0.30, 0.16)		DataPointRange(27280 - 27297)
2417	303.0193	S	0.0219	(0.30, 0.15)-(0.40, 0.15)	DataPointRange(27298 - 27299)	0.0219	0.09	4.28	5.93
2418	303.0522	F	0.1989	(0.40, 0.15)		DataPointRange(27300 - 27317)
2419	303.2401	S	0.0221	(0.39, 0.16)-(0.47, 0.16)	DataPointRange(27318 - 27319)	0.0221	0.08	3.42	4.61
2420	303.2731	F	0.1878	(0.47, 0.17)		DataPointRange(27320 - 27336)
2421	303.4498	S	0.0444	(0.47, 0.16)-(0.64, 0.16)	DataPointRange(27337 - 27340)	0.0444	0.17	3.74	6.66
2422	303.5052	F	0.1883	(0.65, 0.17)		DataPointRange(27341 - 27357)
2423	303.6819	S	0.0221	(0.64, 0.15)-(0.74, 0.16)	DataPointRange(27358 - 27359)	0.0221	0.10	4.41	5.68
2424	303.7149	F	0.1989	(0.76, 0.16)		DataPointRange(27360 - 27377)
2425	303.9026	S	0.0665	(0.75, 0.18)-(0.21, 0.21)	DataPointRange(27378 - 27383)	0.0665	0.54	8.16	10.95
2426	303.9800	F	0.2762	(0.20, 0.25)		DataPointRange(27384 - 27408)
2427	304.2452	S	0.0333	(0.21, 0.24)-(0.32, 0.24)	DataPointRange(27409 - 27411)	0.0333	0.10	3.13	5.24
2428	304.2898	F	0.2320	(0.34, 0.24)		DataPointRange(27412 - 27432)
2429	304.5103	S	0.0333	(0.35, 0.24)-(0.49, 0.24)	DataPointRange(27433 - 27435)	0.0333	0.14	4.10	6.65
2430	304.5549	F	0.2425	(0.49, 0.24)		DataPointRange(27436 - 27457)
2431	304.7866	S	0.0331	(0.49, 0.26)-(0.63, 0.23)	DataPointRange(27458 - 27460)	0.0331	0.14	4.37	7.53
2432	304.8307	F	0.5967	(0.64, 0.24)		DataPointRange(27461 - 27514)
2433	305.4163	S	0.0221	(0.64, 0.22)-(0.70, 0.24)	DataPointRange(27515 - 27516)	0.0221	0.06	2.80	5.20
2434	305.4494	F	0.1327	(0.74, 0.23)		DataPointRange(27517 - 27528)
2435	305.5710	S	0.0221	(0.75, 0.23)-(0.80, 0.23)	DataPointRange(27529 - 27530)	0.0221	0.05	2.42	3.72
2436	305.6041	F	0.2319	(0.83, 0.24)		DataPointRange(27531 - 27551)
2437	305.8249	S	0.0773	(0.83, 0.24)-(0.30, 0.30)	DataPointRange(27552 - 27558)	0.0773	0.53	6.90	10.82
2438	305.9134	F	0.1213	(0.30, 0.32)		DataPointRange(27559 - 27569)
2439	306.0237	S	0.0333	(0.30, 0.29)-(0.18, 0.34)	DataPointRange(27570 - 27572)	0.0333	0.13	3.78	6.83
2440	306.0680	F	0.2209	(0.17, 0.35)		DataPointRange(27573 - 27592)
2441	306.2780	S	0.0442	(0.18, 0.36)-(0.36, 0.35)	DataPointRange(27593 - 27596)	0.0442	0.19	4.19	6.98
2442	306.3332	F	0.3091	(0.37, 0.33)		DataPointRange(27597 - 27624)
2443	306.6312	S	0.0222	(0.36, 0.33)-(0.45, 0.32)	DataPointRange(27625 - 27626)	0.0222	0.09	3.84	6.60
2444	306.6645	F	0.1768	(0.48, 0.32)		DataPointRange(27627 - 27642)
2445	306.8303	S	0.0219	(0.50, 0.32)-(0.58, 0.32)	DataPointRange(27643 - 27644)	0.0219	0.08	3.70	7.25
2446	306.8633	F	0.2649	(0.61, 0.33)		DataPointRange(27645 - 27668)
2447	307.1171	S	0.0111	(0.60, 0.33)-(0.64, 0.34)	DataPointRange(27669 - 27669)	0.0111	0.04	3.31	3.31
2448	307.1394	F	0.1216	(0.65, 0.34)		DataPointRange(27670 - 27680)
2449	307.2499	S	0.0663	(0.64, 0.35)-(0.20, 0.41)	DataPointRange(27681 - 27686)	0.0663	0.44	6.70	11.25
2450	307.3270	F	0.2436	(0.21, 0.40)		DataPointRange(27687 - 27708)
2451	307.5593	S	0.0222	(0.21, 0.40)-(0.26, 0.41)	DataPointRange(27709 - 27710)	0.0222	0.05	2.26	4.36
2452	307.5923	F	0.2649	(0.27, 0.42)		DataPointRange(27711 - 27734)
2453	307.8463	S	0.0220	(0.28, 0.40)-(0.23, 0.43)	DataPointRange(27735 - 27736)	0.0220	0.06	2.59	3.33
2454	307.8795	F	0.2981	(0.23, 0.43)		DataPointRange(27737 - 27763)
2455	308.1667	S	0.0220	(0.24, 0.41)-(0.29, 0.43)	DataPointRange(27764 - 27765)	0.0220	0.05	2.18	3.90
2456	308.2001	F	0.2758	(0.30, 0.41)		DataPointRange(27766 - 27790)
2457	308.4653	S	0.0439	(0.29, 0.39)-(0.47, 0.42)	DataPointRange(27791 - 27794)	0.0439	0.18	4.16	7.33
2458	308.5202	F	0.2432	(0.47, 0.41)		DataPointRange(27795 - 27816)
2459	308.7521	S	0.0330	(0.46, 0.41)-(0.57, 0.42)	DataPointRange(27817 - 27819)	0.0330	0.12	3.59	6.08
2460	308.7963	F	0.3314	(0.58, 0.42)		DataPointRange(27820 - 27849)
2461	309.1166	S	0.0220	(0.57, 0.42)-(0.62, 0.40)	DataPointRange(27850 - 27851)	0.0220	0.06	2.67	3.61
2462	309.1497	F	0.1766	(0.63, 0.41)		DataPointRange(27852 - 27867)
2463	309.3153	S	0.0110	(0.63, 0.41)-(0.62, 0.39)	DataPointRange(27868 - 27868)	0.0110	0.02	2.09	2.09
2464	309.3376	F	0.1876	(0.62, 0.33)		DataPointRange(27869 - 27885)
2465	309.5142	S	0.0333	(0.62, 0.35)-(0.69, 0.44)	DataPointRange(27886 - 27888)	0.0333	0.10	3.07	5.34
2466	309.5588	F	0.1210	(0.70, 0.44)		DataPointRange(27889 - 27899)
2467	309.6689	S	0.0773	(0.71, 0.46)-(0.16, 0.57)	DataPointRange(27900 - 27906)	0.0773	0.55	7.18	12.92
2468	309.7575	F	0.2098	(0.16, 0.56)		DataPointRange(27907 - 27925)
2469	309.9561	S	0.0221	(0.16, 0.58)-(0.22, 0.53)	DataPointRange(27926 - 27927)	0.0221	0.07	3.03	5.21
2470	309.9897	F	0.1692	(0.25, 0.53)		DataPointRange(27928 - 27942)
2471	310.1438	S	0.0333	(0.26, 0.55)-(0.38, 0.53)	DataPointRange(27943 - 27945)	0.0333	0.12	3.61	9.81
2472	310.1882	F	0.2318	(0.41, 0.52)		DataPointRange(27946 - 27966)
2473	310.4090	S	0.0331	(0.42, 0.53)-(0.58, 0.52)	DataPointRange(27967 - 27969)	0.0331	0.17	4.99	7.83
2474	310.4534	F	0.3091	(0.63, 0.50)		DataPointRange(27970 - 27997)
2475	310.7515	S	0.0221	(0.62, 0.50)-(0.67, 0.51)	DataPointRange(27998 - 27999)	0.0221	0.05	2.20	3.37
2476	310.7848	F	0.1326	(0.69, 0.51)		DataPointRange(28000 - 28011)
2477	310.9059	S	0.0664	(0.69, 0.50)-(0.15, 1.34)	DataPointRange(28012 - 28017)	0.0664	0.83	12.52	118.16
2478	310.9837	F	0.0106	(0.16, 1.38)		DataPointRange(28018 - 28018)
2479	310.9837	S	0.0548	(0.16, 1.38)-(1.86, -0.32)	DataPointRange(28019 - 28023)	0.0548	2.12	38.69	193.16
2480	311.0497	F	0.0110	(1.86, -0.36)		DataPointRange(28024 - 28024)
2481	311.0497	S	0.1436	(1.86, -0.36)-(0.57, 0.48)	DataPointRange(28025 - 28037)	0.1436	1.44	10.00	276.33
2482	311.2042	F	0.0332	(0.58, 0.43)		DataPointRange(28038 - 28040)
2483	311.2263	S	0.0225	(0.58, 0.42)-(0.58, 0.50)	DataPointRange(28041 - 28042)	0.0225	0.06	2.62	2.76
2484	311.2594	F	0.1550	(0.58, 0.51)		DataPointRange(28043 - 28056)
2485	311.4031	S	0.0113	(0.58, 0.51)-(0.61, 0.49)	DataPointRange(28057 - 28057)	0.0113	0.03	2.82	2.82
2486	311.4253	F	0.2982	(0.66, 0.52)		DataPointRange(28058 - 28084)
2487	311.7125	S	0.0883	(0.66, 0.52)-(1.87, -0.24)	DataPointRange(28085 - 28092)	0.0883	1.33	15.08	294.10
2488	311.8122	F	0.0108	(1.86, -0.36)		DataPointRange(28093 - 28093)
2489	311.8122	S	0.0108	(1.86, -0.36)-(1.87, -0.22)	DataPointRange(28094 - 28094)	0.0108	0.11	10.36	10.36
2490	311.8342	F	0.0331	(1.87, -0.22)		DataPointRange(28095 - 28097)
2491	311.8561	S	0.2211	(1.87, -0.22)-(0.57, 0.40)	DataPointRange(28098 - 28117)	0.2211	1.39	6.27	274.80
2492	312.0882	F	0.0221	(0.58, 0.40)		DataPointRange(28118 - 28119)
2493	312.0989	S	0.0222	(0.58, 0.38)-(0.59, 0.45)	DataPointRange(28120 - 28121)	0.0222	0.05	2.45	6.34
2494	312.1322	F	0.0993	(0.59, 0.47)		DataPointRange(28122 - 28130)
2495	312.2205	S	0.0110	(0.59, 0.47)-(0.55, 0.47)	DataPointRange(28131 - 28131)	0.0110	0.04	3.38	3.38
2496	312.2429	F	0.1546	(0.52, 0.48)		DataPointRange(28132 - 28145)
2497	312.3864	S	0.0332	(0.52, 0.49)-(0.66, 0.52)	DataPointRange(28146 - 28148)	0.0332	0.14	4.26	6.95
2498	312.4305	F	0.3204	(0.65, 0.52)		DataPointRange(28149 - 28177)
2499	312.7399	S	0.0441	(0.66, 0.51)-(0.42, 0.50)	DataPointRange(28178 - 28181)	0.0441	0.24	5.43	11.26
2500	312.7951	F	0.1215	(0.41, 0.51)		DataPointRange(28182 - 28192)
2501	312.9057	S	0.0550	(0.42, 0.52)-(0.23, 0.25)	DataPointRange(28193 - 28197)	0.0550	0.28	5.01	8.33
2502	312.9719	F	0.1215	(0.24, 0.22)		DataPointRange(28198 - 28208)
2503	313.0380	End					DataPoint(28204)
2504	313.0822	S	0.0224	(0.24, 0.23)-(0.21, 0.16)	DataPointRange(28209 - 28210)	0.0224	0.06	2.53	4.80
2505	313.1156	F	0.4967	(0.19, 0.15)		DataPointRange(28211 - 28255)
2506	313.4248	t1n2.bmp					DataPoint(28239)
2507	313.4908	Start					DataPoint(28245)
2508	313.6014	S	0.0221	(0.18, 0.17)-(0.27, 0.16)	DataPointRange(28256 - 28257)	0.0221	0.09	3.97	5.12
2509	313.6345	F	0.1548	(0.28, 0.16)		DataPointRange(28258 - 28271)
2510	313.7782	S	0.0333	(0.28, 0.15)-(0.43, 0.14)	DataPointRange(28272 - 28274)	0.0333	0.15	4.54	8.58
2511	313.8223	F	0.1992	(0.46, 0.16)		DataPointRange(28275 - 28292)
2512	314.0101	S	0.0223	(0.47, 0.16)-(0.56, 0.17)	DataPointRange(28293 - 28294)	0.0223	0.09	4.05	4.18
2513	314.0434	F	0.1986	(0.59, 0.17)		DataPointRange(28295 - 28312)
2514	314.2310	S	0.0554	(0.59, 0.16)-(0.20, 0.25)	DataPointRange(28313 - 28317)	0.0554	0.39	7.13	10.15
2515	314.2977	F	0.3200	(0.19, 0.24)		DataPointRange(28318 - 28346)
2516	314.6067	S	0.0110	(0.19, 0.25)-(0.22, 0.24)	DataPointRange(28347 - 28347)	0.0110	0.03	2.81	2.81
2517	314.6288	F	0.2872	(0.27, 0.25)		DataPointRange(28348 - 28373)
2518	314.9050	S	0.0220	(0.26, 0.24)-(0.39, 0.25)	DataPointRange(28374 - 28375)	0.0220	0.13	5.96	7.11
2519	314.9382	F	0.4086	(0.44, 0.26)		DataPointRange(28376 - 28412)
2520	315.3357	S	0.0111	(0.44, 0.24)-(0.48, 0.25)	DataPointRange(28413 - 28413)	0.0111	0.04	3.31	3.31
2521	315.3578	F	0.2210	(0.51, 0.26)		DataPointRange(28414 - 28433)
2522	315.5678	S	0.0441	(0.53, 0.27)-(0.23, 0.34)	DataPointRange(28434 - 28437)	0.0441	0.30	6.89	9.93
2523	315.6232	F	0.2536	(0.21, 0.34)		DataPointRange(28438 - 28460)
2524	315.8658	S	0.0224	(0.20, 0.33)-(0.29, 0.33)	DataPointRange(28461 - 28462)	0.0224	0.08	3.77	4.92
2525	315.8990	F	0.2100	(0.29, 0.34)		DataPointRange(28463 - 28481)
2526	316.0980	S	0.0219	(0.29, 0.34)-(0.34, 0.33)	DataPointRange(28482 - 28483)	0.0219	0.05	2.15	4.22
2527	316.1311	F	0.1988	(0.37, 0.34)		DataPointRange(28484 - 28501)
2528	316.3187	S	0.0331	(0.38, 0.34)-(0.53, 0.35)	DataPointRange(28502 - 28504)	0.0331	0.15	4.51	6.14
2529	316.3631	F	0.1987	(0.55, 0.35)		DataPointRange(28505 - 28522)
2530	316.5508	S	0.0335	(0.54, 0.35)-(0.64, 0.35)	DataPointRange(28523 - 28525)	0.0335	0.10	3.06	4.81
2531	316.5950	F	0.2871	(0.69, 0.35)		DataPointRange(28526 - 28551)
2532	316.8713	S	0.0551	(0.69, 0.35)-(0.27, 0.39)	DataPointRange(28552 - 28556)	0.0551	0.42	7.66	11.62
2533	316.9372	F	0.1880	(0.24, 0.42)		DataPointRange(28557 - 28573)
2534	317.1146	S	0.0218	(0.24, 0.42)-(0.19, 0.44)	DataPointRange(28574 - 28575)	0.0218	0.05	2.17	4.48
2535	317.1475	F	0.1985	(0.18, 0.45)		DataPointRange(28576 - 28593)
2536	317.3349	S	0.0333	(0.18, 0.45)-(0.32, 0.43)	DataPointRange(28594 - 28596)	0.0333	0.14	4.27	7.57
2537	317.3794	F	0.5303	(0.34, 0.43)		DataPointRange(28597 - 28644)
2538	317.8984	S	0.0113	(0.34, 0.44)-(0.37, 0.42)	DataPointRange(28645 - 28645)	0.0113	0.04	3.16	3.16
2539	317.9204	F	0.4419	(0.39, 0.42)		DataPointRange(28646 - 28685)
2540	318.3512	S	0.0333	(0.39, 0.41)-(0.47, 0.40)	DataPointRange(28686 - 28688)	0.0333	0.09	2.61	3.70
2541	318.3956	F	0.2207	(0.48, 0.40)		DataPointRange(28689 - 28708)
2542	318.6055	S	0.0108	(0.48, 0.40)-(0.47, 0.39)	DataPointRange(28709 - 28709)	0.0108	0.02	1.46	1.46
2543	318.6275	F	0.2652	(0.44, 0.42)		DataPointRange(28710 - 28733)
2544	318.8814	S	0.0333	(0.43, 0.41)-(0.39, 0.33)	DataPointRange(28734 - 28736)	0.0333	0.07	2.19	4.25
2545	318.9256	F	0.2651	(0.39, 0.27)		DataPointRange(28737 - 28760)
2546	319.1797	S	0.0223	(0.39, 0.27)-(0.28, 0.21)	DataPointRange(28761 - 28762)	0.0223	0.12	5.44	7.00
2547	319.2128	F	0.9389	(0.24, 0.18)		DataPointRange(28763 - 28847)
2548	319.6215	End					DataPoint(28800)
2549	319.9749	aa1n5s.bmp				DataPoint(28832)
2550	320.1074	Start					DataPoint(28844)
2551	320.1407	S	0.0110	(0.23, 0.17)-(0.19, 0.16)	DataPointRange(28848 - 28848)	0.0110	0.04	3.53	3.53
2552	320.1630	F	0.1327	(0.19, 0.15)		DataPointRange(28849 - 28860)
2553	320.2843	S	0.0221	(0.19, 0.15)-(0.26, 0.16)	DataPointRange(28861 - 28862)	0.0221	0.08	3.46	5.56
2554	320.3173	F	0.2210	(0.29, 0.15)		DataPointRange(28863 - 28882)
2555	320.5275	S	0.0333	(0.29, 0.17)-(0.41, 0.17)	DataPointRange(28883 - 28885)	0.0333	0.12	3.61	5.37
2556	320.5715	F	0.2319	(0.44, 0.16)		DataPointRange(28886 - 28906)
2557	320.7925	S	0.0550	(0.44, 0.17)-(0.44, 0.24)	DataPointRange(28907 - 28911)	0.0550	0.05	0.96	12.30
2558	320.8587	F	0.0110	(0.46, 0.26)		DataPointRange(28912 - 28912)
2559	320.8587	S	0.0219	(0.46, 0.26)-(0.46, 0.20)	DataPointRange(28913 - 28914)	0.0219	0.05	2.27	3.98
2560	320.8921	F	0.2428	(0.49, 0.17)		DataPointRange(28915 - 28936)
2561	321.1241	S	0.0217	(0.49, 0.17)-(0.60, 0.17)	DataPointRange(28937 - 28938)	0.0217	0.11	5.18	7.54
2562	321.1587	F	0.3297	(0.64, 0.16)		DataPointRange(28939 - 28968)
2563	321.4774	S	0.0221	(0.65, 0.17)-(0.71, 0.17)	DataPointRange(28969 - 28970)	0.0221	0.06	2.68	4.25
2564	321.5106	F	0.1989	(0.73, 0.16)		DataPointRange(28971 - 28988)
2565	321.6985	S	0.0661	(0.73, 0.17)-(0.17, 0.22)	DataPointRange(28989 - 28994)	0.0661	0.56	8.51	12.07
2566	321.7756	F	0.2434	(0.17, 0.27)		DataPointRange(28995 - 29016)
2567	322.0077	S	0.0221	(0.16, 0.27)-(0.27, 0.25)	DataPointRange(29017 - 29018)	0.0221	0.11	4.88	6.01
2568	322.0409	F	0.2316	(0.27, 0.26)		DataPointRange(29019 - 29039)
2569	322.2615	S	0.0441	(0.28, 0.26)-(0.43, 0.24)	DataPointRange(29040 - 29043)	0.0441	0.15	3.41	6.37
2570	322.3169	F	0.2429	(0.46, 0.25)		DataPointRange(29044 - 29065)
2571	322.5487	S	0.0221	(0.46, 0.25)-(0.54, 0.26)	DataPointRange(29066 - 29067)	0.0221	0.08	3.55	5.10
2572	322.5818	F	0.3316	(0.53, 0.25)		DataPointRange(29068 - 29097)
2573	322.9023	S	0.0446	(0.52, 0.27)-(0.70, 0.25)	DataPointRange(29098 - 29101)	0.0446	0.18	4.00	5.89
2574	322.9577	F	0.2760	(0.69, 0.24)		DataPointRange(29102 - 29126)
2575	323.2227	S	0.0220	(0.70, 0.24)-(0.80, 0.24)	DataPointRange(29127 - 29128)	0.0220	0.10	4.68	6.05
2576	323.2559	F	0.1437	(0.84, 0.24)		DataPointRange(29129 - 29141)
2577	323.3885	S	0.0221	(0.84, 0.24)-(0.73, 0.23)	DataPointRange(29142 - 29143)	0.0221	0.11	4.93	7.87
2578	323.4220	F	0.2540	(0.70, 0.23)		DataPointRange(29144 - 29166)
2579	323.6647	S	0.0330	(0.70, 0.23)-(0.82, 0.25)	DataPointRange(29167 - 29169)	0.0330	0.12	3.69	6.41
2580	323.7086	F	0.2212	(0.87, 0.24)		DataPointRange(29170 - 29189)
2581	323.9186	S	0.0332	(0.88, 0.25)-(0.75, 0.23)	DataPointRange(29190 - 29192)	0.0332	0.13	3.85	6.77
2582	323.9629	F	0.2650	(0.77, 0.25)		DataPointRange(29193 - 29216)
2583	324.2167	S	0.0222	(0.77, 0.24)-(0.86, 0.25)	DataPointRange(29217 - 29218)	0.0222	0.09	3.95	4.91
2584	324.2499	F	0.0886	(0.87, 0.24)		DataPointRange(29219 - 29226)
2585	324.3273	S	0.0777	(0.87, 0.25)-(0.02, 0.17)	DataPointRange(29227 - 29233)	0.0777	0.86	11.01	23.99
2586	324.4158	F	0.0110	(-0.02, 0.20)		DataPointRange(29234 - 29234)
2587	324.4158	S	0.0331	(-0.02, 0.20)-(0.15, 0.38)	DataPointRange(29235 - 29237)	0.0331	0.22	6.64	16.17
2588	324.4597	F	0.1770	(0.16, 0.39)		DataPointRange(29238 - 29253)
2589	324.6254	S	0.0332	(0.16, 0.36)-(0.31, 0.35)	DataPointRange(29254 - 29256)	0.0332	0.15	4.58	7.28
2590	324.6696	F	0.2875	(0.35, 0.34)		DataPointRange(29257 - 29282)
2591	324.9460	S	0.0439	(0.36, 0.35)-(0.55, 0.34)	DataPointRange(29283 - 29286)	0.0439	0.19	4.26	6.62
2592	325.0015	F	0.8168	(0.56, 0.34)		DataPointRange(29287 - 29360)
2593	325.8074	S	0.0331	(0.55, 0.33)-(0.73, 0.35)	DataPointRange(29361 - 29363)	0.0331	0.17	5.23	8.30
2594	325.8515	F	0.2872	(0.77, 0.33)		DataPointRange(29364 - 29389)
2595	326.1277	S	0.0443	(0.78, 0.32)-(0.47, 0.28)	DataPointRange(29390 - 29393)	0.0443	0.31	7.07	11.25
2596	326.1830	F	0.2322	(0.48, 0.27)		DataPointRange(29394 - 29414)
2597	326.4041	S	0.0220	(0.47, 0.27)-(0.41, 0.26)	DataPointRange(29415 - 29416)	0.0220	0.06	2.71	5.51
2598	326.4372	F	0.2098	(0.38, 0.26)		DataPointRange(29417 - 29435)
2599	326.6359	S	0.0331	(0.38, 0.27)-(0.26, 0.19)	DataPointRange(29436 - 29438)	0.0331	0.13	3.95	5.89
2600	326.6801	F	0.1766	(0.26, 0.20)		DataPointRange(29439 - 29454)
2601	326.8458	S	0.0109	(0.26, 0.20)-(0.26, 0.17)	DataPointRange(29455 - 29455)	0.0109	0.02	2.29	2.29
2602	326.8678	F	0.2542	(0.30, 0.16)		DataPointRange(29456 - 29478)
2603	327.1108	S	0.0220	(0.30, 0.16)-(0.39, 0.16)	DataPointRange(29479 - 29480)	0.0220	0.09	4.02	7.47
2604	327.1439	F	0.2766	(0.41, 0.16)		DataPointRange(29481 - 29505)
2605	327.1771	End					DataPoint(29484)
2606	327.4090	S	0.0222	(0.42, 0.15)-(0.33, 0.16)	DataPointRange(29506 - 29507)	0.0222	0.09	3.90	7.55
2607	327.4423	F	0.2540	(0.30, 0.16)		DataPointRange(29508 - 29530)
2608	327.6744	aa1n11.bmp				DataPoint(29529)
2609	327.6857	S	0.0216	(0.30, 0.15)-(0.20, 0.13)	DataPointRange(29531 - 29532)	0.0216	0.09	4.36	5.09
2610	327.7186	Start					DataPoint(29533)
2611	327.7186	F	0.2207	(0.20, 0.15)		DataPointRange(29533 - 29552)
2612	327.9282	S	0.0221	(0.19, 0.15)-(0.29, 0.17)	DataPointRange(29553 - 29554)	0.0221	0.10	4.68	5.68
2613	327.9614	F	0.1438	(0.32, 0.16)		DataPointRange(29555 - 29567)
2614	328.0941	S	0.0221	(0.31, 0.16)-(0.38, 0.14)	DataPointRange(29568 - 29569)	0.0221	0.07	3.04	5.63
2615	328.1272	F	0.1767	(0.43, 0.16)		DataPointRange(29570 - 29585)
2616	328.2930	S	0.0224	(0.43, 0.16)-(0.50, 0.17)	DataPointRange(29586 - 29587)	0.0224	0.07	3.23	4.79
2617	328.3260	F	0.2761	(0.51, 0.16)		DataPointRange(29588 - 29612)
2618	328.5912	S	0.0332	(0.51, 0.18)-(0.63, 0.15)	DataPointRange(29613 - 29615)	0.0332	0.12	3.70	4.86
2619	328.6357	F	0.3752	(0.66, 0.16)		DataPointRange(29616 - 29649)
2620	328.9997	S	0.0221	(0.67, 0.16)-(0.57, 0.16)	DataPointRange(29650 - 29651)	0.0221	0.09	4.27	7.55
2621	329.0328	F	0.2872	(0.55, 0.16)		DataPointRange(29652 - 29677)
2622	329.3093	S	0.0439	(0.55, 0.17)-(0.75, 0.14)	DataPointRange(29678 - 29681)	0.0439	0.20	4.65	7.84
2623	329.3642	F	0.2100	(0.76, 0.16)		DataPointRange(29682 - 29700)
2624	329.5632	S	0.0883	(0.77, 0.16)-(0.16, 0.26)	DataPointRange(29701 - 29708)	0.0883	0.61	6.97	20.75
2625	329.6625	F	0.1991	(0.15, 0.26)		DataPointRange(29709 - 29726)
2626	329.8505	S	0.0223	(0.16, 0.28)-(0.22, 0.23)	DataPointRange(29727 - 29728)	0.0223	0.08	3.51	4.81
2627	329.8836	F	0.1549	(0.23, 0.25)		DataPointRange(29729 - 29742)
2628	330.0271	S	0.0221	(0.23, 0.25)-(0.32, 0.25)	DataPointRange(29743 - 29744)	0.0221	0.09	4.02	4.35
2629	330.0602	F	0.2650	(0.34, 0.25)		DataPointRange(29745 - 29768)
2630	330.3144	S	0.0331	(0.33, 0.24)-(0.47, 0.24)	DataPointRange(29769 - 29771)	0.0331	0.14	4.16	4.58
2631	330.3584	F	0.2430	(0.48, 0.24)		DataPointRange(29772 - 29793)
2632	330.5903	S	0.0331	(0.48, 0.25)-(0.62, 0.26)	DataPointRange(29794 - 29796)	0.0331	0.14	4.32	6.35
2633	330.6346	F	0.2433	(0.64, 0.25)		DataPointRange(29797 - 29818)
2634	330.8668	S	0.0331	(0.63, 0.25)-(0.72, 0.27)	DataPointRange(29819 - 29821)	0.0331	0.09	2.73	4.54
2635	330.9109	F	0.2981	(0.74, 0.25)		DataPointRange(29822 - 29848)
2636	331.1979	S	0.0220	(0.75, 0.26)-(0.80, 0.25)	DataPointRange(29849 - 29850)	0.0220	0.05	2.44	4.81
2637	331.2312	F	0.3091	(0.80, 0.25)		DataPointRange(29851 - 29878)
2638	331.5295	S	0.0329	(0.80, 0.24)-(0.59, 0.22)	DataPointRange(29879 - 29881)	0.0329	0.21	6.44	9.05
2639	331.5734	F	0.2654	(0.57, 0.24)		DataPointRange(29882 - 29905)
2640	331.8280	S	0.1674	(0.58, 0.24)-(0.52, 0.30)	DataPointRange(29906 - 29920)	0.1674	0.07	0.45	65.67
2641	332.0046	F	0.2868	(0.53, 0.25)		DataPointRange(29921 - 29946)
2642	332.2804	S	0.0110	(0.53, 0.24)-(0.49, 0.24)	DataPointRange(29947 - 29947)	0.0110	0.04	3.75	3.75
2643	332.3025	F	0.5746	(0.44, 0.25)		DataPointRange(29948 - 29999)
2644	332.5125	End					DataPoint(29967)
2645	332.8662	S	0.0331	(0.44, 0.23)-(0.29, 0.23)	DataPointRange(30000 - 30002)	0.0331	0.15	4.57	6.84
2646	332.9103	F	0.1375	(0.29, 0.22)		DataPointRange(30003 - 30013)
2647	332.9653	aa1n12.bmp				DataPoint(30008)
2648	333.0363	S	0.0173	(0.28, 0.22)-(0.20, 0.19)	DataPointRange(30014 - 30015)	0.0173	0.09	4.94	13.91
2649	333.0536	Start					DataPoint(30015)
2650	333.0646	F	0.1547	(0.17, 0.17)		DataPointRange(30016 - 30029)
2651	333.2083	S	0.0224	(0.17, 0.19)-(0.23, 0.16)	DataPointRange(30030 - 30031)	0.0224	0.07	3.09	4.06
2652	333.2415	F	0.1766	(0.23, 0.16)		DataPointRange(30032 - 30047)
2653	333.4072	S	0.0221	(0.23, 0.16)-(0.31, 0.18)	DataPointRange(30048 - 30049)	0.0221	0.08	3.67	5.90
2654	333.4402	F	0.2100	(0.32, 0.17)		DataPointRange(30050 - 30068)
2655	333.6392	S	0.0220	(0.31, 0.16)-(0.41, 0.16)	DataPointRange(30069 - 30070)	0.0220	0.10	4.47	5.48
2656	333.6723	F	0.3315	(0.42, 0.16)		DataPointRange(30071 - 30100)
2657	333.9928	S	0.0219	(0.43, 0.17)-(0.50, 0.16)	DataPointRange(30101 - 30102)	0.0219	0.07	2.99	3.75
2658	334.0260	F	0.1876	(0.52, 0.16)		DataPointRange(30103 - 30119)
2659	334.2026	S	0.0332	(0.52, 0.15)-(0.68, 0.16)	DataPointRange(30120 - 30122)	0.0332	0.15	4.60	7.30
2660	334.2469	F	0.2318	(0.70, 0.16)		DataPointRange(30123 - 30143)
2661	334.4675	S	0.0223	(0.69, 0.16)-(0.76, 0.16)	DataPointRange(30144 - 30145)	0.0223	0.07	3.09	6.17
2662	334.5007	F	0.0664	(0.79, 0.15)		DataPointRange(30146 - 30151)
2663	334.5563	S	0.0879	(0.78, 0.16)-(0.17, 0.28)	DataPointRange(30152 - 30159)	0.0879	0.62	7.03	11.83
2664	334.6556	F	0.3534	(0.18, 0.27)		DataPointRange(30160 - 30191)
2665	334.9980	S	0.0221	(0.17, 0.27)-(0.24, 0.23)	DataPointRange(30192 - 30193)	0.0221	0.07	3.16	5.00
2666	335.0309	F	0.1657	(0.25, 0.25)		DataPointRange(30194 - 30208)
2667	335.1855	S	0.0332	(0.24, 0.24)-(0.38, 0.24)	DataPointRange(30209 - 30211)	0.0332	0.14	4.11	7.36
2668	335.2297	F	0.2321	(0.42, 0.24)		DataPointRange(30212 - 30232)
2669	335.4506	S	0.0221	(0.43, 0.26)-(0.52, 0.23)	DataPointRange(30233 - 30234)	0.0221	0.09	4.28	6.40
2670	335.4842	F	0.2095	(0.53, 0.25)		DataPointRange(30235 - 30253)
2671	335.6826	S	0.0554	(0.53, 0.25)-(0.18, 0.33)	DataPointRange(30254 - 30258)	0.0554	0.36	6.48	9.26
2672	335.7493	F	0.2870	(0.18, 0.33)		DataPointRange(30259 - 30284)
2673	336.0253	S	0.0884	(0.19, 0.34)-(1.03, 1.74)	DataPointRange(30285 - 30292)	0.0884	1.35	15.26	175.05
2674	336.1244	F	0.0110	(1.00, 1.74)		DataPointRange(30293 - 30293)
2675	336.1244	S	0.1547	(1.00, 1.74)-(0.22, 0.43)	DataPointRange(30294 - 30307)	0.1547	1.26	8.11	283.70
2676	336.2902	F	0.0222	(0.21, 0.38)		DataPointRange(30308 - 30309)
2677	336.3012	S	0.0112	(0.21, 0.39)-(0.23, 0.33)	DataPointRange(30310 - 30310)	0.0112	0.05	4.40	4.40
2678	336.3233	F	0.0333	(0.24, 0.31)		DataPointRange(30311 - 30313)
2679	336.3456	S	0.0110	(0.25, 0.30)-(0.26, 0.35)	DataPointRange(30314 - 30314)	0.0110	0.03	3.02	3.02
2680	336.3677	F	0.1544	(0.24, 0.36)		DataPointRange(30315 - 30328)
2681	336.5111	S	0.0223	(0.25, 0.35)-(0.30, 0.34)	DataPointRange(30329 - 30330)	0.0223	0.06	2.51	3.77
2682	336.5444	F	0.1328	(0.33, 0.35)		DataPointRange(30331 - 30342)
2683	336.6657	S	0.0332	(0.34, 0.35)-(0.42, 0.34)	DataPointRange(30343 - 30345)	0.0332	0.09	2.60	4.76
2684	336.7099	F	0.2430	(0.45, 0.34)		DataPointRange(30346 - 30367)
2685	336.9420	S	0.0330	(0.45, 0.33)-(0.59, 0.34)	DataPointRange(30368 - 30370)	0.0330	0.14	4.26	6.64
2686	336.9861	F	0.1438	(0.61, 0.34)		DataPointRange(30371 - 30383)
2687	337.1188	S	0.0111	(0.61, 0.35)-(0.58, 0.33)	DataPointRange(30384 - 30384)	0.0111	0.03	3.04	3.04
2688	337.1409	F	0.1436	(0.54, 0.34)		DataPointRange(30385 - 30397)
2689	337.2735	S	0.0439	(0.55, 0.33)-(0.70, 0.38)	DataPointRange(30398 - 30401)	0.0439	0.16	3.65	6.04
2690	337.3286	F	0.1546	(0.72, 0.35)		DataPointRange(30402 - 30415)
2691	337.4721	S	0.0665	(0.71, 0.34)-(0.18, 0.47)	DataPointRange(30416 - 30421)	0.0665	0.54	8.15	13.17
2692	337.5494	F	0.4198	(0.17, 0.46)		DataPointRange(30422 - 30459)
2693	337.9583	S	0.0332	(0.17, 0.46)-(0.33, 0.45)	DataPointRange(30460 - 30462)	0.0332	0.16	4.91	7.66
2694	338.0025	F	0.2539	(0.34, 0.44)		DataPointRange(30463 - 30485)
2695	338.2455	S	0.0219	(0.35, 0.46)-(0.40, 0.44)	DataPointRange(30486 - 30487)	0.0219	0.06	2.63	5.95
2696	338.2784	F	0.2100	(0.43, 0.44)		DataPointRange(30488 - 30506)
2697	338.4775	S	0.0329	(0.42, 0.46)-(0.56, 0.43)	DataPointRange(30507 - 30509)	0.0329	0.14	4.27	6.98
2698	338.5215	F	0.2322	(0.61, 0.44)		DataPointRange(30510 - 30530)
2699	338.7428	S	0.0222	(0.60, 0.42)-(0.69, 0.45)	DataPointRange(30531 - 30532)	0.0222	0.09	4.22	8.50
2700	338.7758	F	0.2318	(0.71, 0.43)		DataPointRange(30533 - 30553)
2701	338.9966	S	0.0664	(0.70, 0.43)-(0.19, 0.55)	DataPointRange(30554 - 30559)	0.0664	0.52	7.85	12.23
2702	339.0738	F	0.1991	(0.20, 0.54)		DataPointRange(30560 - 30577)
2703	339.2618	S	0.0219	(0.18, 0.53)-(0.14, 0.57)	DataPointRange(30578 - 30579)	0.0219	0.06	2.60	6.26
2704	339.2949	F	0.2653	(0.16, 0.56)		DataPointRange(30580 - 30603)
2705	339.5489	S	0.0222	(0.16, 0.59)-(0.23, 0.55)	DataPointRange(30604 - 30605)	0.0222	0.07	3.24	5.81
2706	339.5819	F	0.7625	(0.25, 0.55)		DataPointRange(30606 - 30674)
2707	340.3331	S	0.0442	(0.23, 0.55)-(0.46, 0.46)	DataPointRange(30675 - 30678)	0.0442	0.25	5.57	7.99
2708	340.3885	F	0.3316	(0.49, 0.44)		DataPointRange(30679 - 30708)
2709	340.7088	S	0.1767	(0.49, 0.44)-(0.61, 0.38)	DataPointRange(30709 - 30724)	0.1767	0.12	0.70	31.22
2710	340.8965	F	0.1766	(0.60, 0.39)		DataPointRange(30725 - 30740)
2711	341.0624	S	0.0439	(0.60, 0.41)-(0.35, 0.29)	DataPointRange(30741 - 30744)	0.0439	0.27	6.08	7.91
2712	341.1176	F	0.1435	(0.32, 0.27)		DataPointRange(30745 - 30757)
2713	341.2500	S	0.0330	(0.32, 0.28)-(0.22, 0.22)	DataPointRange(30758 - 30760)	0.0330	0.11	3.42	6.32
2714	341.2941	F	0.2653	(0.21, 0.18)		DataPointRange(30761 - 30784)
2715	341.5488	S	0.0106	(0.19, 0.18)-(0.24, 0.16)	DataPointRange(30785 - 30785)	0.0106	0.05	4.34	4.34
2716	341.5705	F	0.7402	(0.26, 0.15)		DataPointRange(30786 - 30852)
2717	341.6036	End					DataPoint(30789)
2718	341.9791	aa1n16s.bmp				DataPoint(30823)
2719	342.1117	Start					DataPoint(30835)
2720	342.2993	S	0.0330	(0.25, 0.15)-(0.40, 0.15)	DataPointRange(30853 - 30855)	0.0330	0.15	4.43	7.75
2721	342.3440	F	0.2537	(0.45, 0.15)		DataPointRange(30856 - 30878)
2722	342.5866	S	0.0222	(0.46, 0.16)-(0.54, 0.16)	DataPointRange(30879 - 30880)	0.0222	0.08	3.57	5.69
2723	342.6198	F	0.2207	(0.55, 0.16)		DataPointRange(30881 - 30900)
2724	342.8295	S	0.0333	(0.54, 0.17)-(0.43, 0.15)	DataPointRange(30901 - 30903)	0.0333	0.11	3.24	6.07
2725	342.8743	F	0.2205	(0.42, 0.16)		DataPointRange(30904 - 30923)
2726	343.0838	S	0.0439	(0.43, 0.15)-(0.60, 0.16)	DataPointRange(30924 - 30927)	0.0439	0.17	3.97	7.30
2727	343.1390	F	0.2073	(0.60, 0.16)		DataPointRange(30928 - 30937)
2728	343.3375	S	0.0225	(0.59, 0.16)-(0.71, 0.17)	DataPointRange(30938 - 30940)	0.0225	0.12	5.54	33.15
2729	343.3707	F	0.1326	(0.73, 0.17)		DataPointRange(30941 - 30952)
2730	343.4924	S	0.0666	(0.74, 0.16)-(0.19, 0.23)	DataPointRange(30953 - 30958)	0.0666	0.55	8.31	11.91
2731	343.5698	F	0.2760	(0.21, 0.24)		DataPointRange(30959 - 30983)
2732	343.8350	S	0.0221	(0.20, 0.25)-(0.27, 0.24)	DataPointRange(30984 - 30985)	0.0221	0.07	3.02	5.76
2733	343.8681	F	0.2321	(0.29, 0.24)		DataPointRange(30986 - 31006)
2734	344.0893	S	0.0327	(0.30, 0.25)-(0.45, 0.26)	DataPointRange(31007 - 31009)	0.0327	0.15	4.72	5.97
2735	344.1332	F	0.0661	(0.46, 0.25)		DataPointRange(31010 - 31015)
2736	344.1886	S	0.5107	(0.47, 0.24)-(0.65, 0.25)	DataPointRange(31016 - 31017)	0.5107	0.18	0.35	2.12
2737	344.8184	F	0.0437	(0.64, 0.26)		DataPointRange(31018 - 31022)
2738	344.8510	S	0.0111	(0.64, 0.26)-(0.66, 0.27)	DataPointRange(31023 - 31023)	0.0111	0.02	1.57	1.57
2739	344.8731	F	0.2209	(0.70, 0.25)		DataPointRange(31024 - 31043)
2740	345.0830	S	0.0662	(0.71, 0.25)-(0.20, 0.31)	DataPointRange(31044 - 31049)	0.0662	0.51	7.72	11.31
2741	345.1602	F	0.1660	(0.20, 0.30)		DataPointRange(31050 - 31064)
2742	345.3152	S	0.0110	(0.20, 0.30)-(0.17, 0.31)	DataPointRange(31065 - 31065)	0.0110	0.02	2.25	2.25
2743	345.3375	F	0.1433	(0.16, 0.34)		DataPointRange(31066 - 31078)
2744	345.4697	S	0.0220	(0.16, 0.34)-(0.22, 0.32)	DataPointRange(31079 - 31080)	0.0220	0.06	2.84	5.21
2745	345.5028	F	0.2098	(0.27, 0.34)		DataPointRange(31081 - 31099)
2746	345.7018	S	0.0440	(0.26, 0.34)-(0.46, 0.33)	DataPointRange(31100 - 31103)	0.0440	0.20	4.59	7.18
2747	345.7567	F	0.6849	(0.47, 0.32)		DataPointRange(31104 - 31165)
2748	346.4306	S	0.0441	(0.47, 0.33)-(0.65, 0.34)	DataPointRange(31166 - 31169)	0.0441	0.18	4.02	5.44
2749	346.4858	F	0.1108	(0.65, 0.35)		DataPointRange(31170 - 31179)
2750	346.5853	S	0.1769	(0.65, 0.37)-(0.72, 0.31)	DataPointRange(31180 - 31195)	0.1769	0.08	0.47	161.61
2751	346.7730	F	0.0225	(0.74, 0.32)		DataPointRange(31196 - 31197)
2752	346.7844	S	0.0219	(0.74, 0.30)-(0.74, 0.37)	DataPointRange(31198 - 31199)	0.0219	0.05	2.24	5.54
2753	346.8173	F	0.2100	(0.73, 0.36)		DataPointRange(31200 - 31218)
2754	347.0162	S	0.0219	(0.73, 0.36)-(0.78, 0.36)	DataPointRange(31219 - 31220)	0.0219	0.05	2.48	4.96
2755	347.0492	F	0.0884	(0.78, 0.35)		DataPointRange(31221 - 31228)
2756	347.1266	S	0.0664	(0.78, 0.34)-(0.21, 0.45)	DataPointRange(31229 - 31234)	0.0664	0.58	8.68	12.21
2757	347.2040	F	0.2871	(0.20, 0.45)		DataPointRange(31235 - 31260)
2758	347.4801	S	0.0219	(0.19, 0.46)-(0.28, 0.43)	DataPointRange(31261 - 31262)	0.0219	0.09	4.15	4.84
2759	347.5131	F	0.1990	(0.28, 0.42)		DataPointRange(31263 - 31280)
2760	347.7009	S	0.0441	(0.29, 0.41)-(0.50, 0.41)	DataPointRange(31281 - 31284)	0.0441	0.21	4.86	7.48
2761	347.7562	F	0.4970	(0.51, 0.42)		DataPointRange(31285 - 31329)
2762	348.2422	S	0.0222	(0.51, 0.45)-(0.60, 0.44)	DataPointRange(31330 - 31331)	0.0222	0.09	3.95	6.04
2763	348.2753	F	0.1548	(0.60, 0.43)		DataPointRange(31332 - 31345)
2764	348.4190	S	0.0220	(0.60, 0.43)-(0.70, 0.41)	DataPointRange(31346 - 31347)	0.0220	0.10	4.64	6.44
2765	348.4524	F	0.2536	(0.69, 0.42)		DataPointRange(31348 - 31370)
2766	348.6950	S	0.0332	(0.70, 0.41)-(0.79, 0.44)	DataPointRange(31371 - 31373)	0.0332	0.09	2.85	5.47
2767	348.7392	F	0.0112	(0.81, 0.33)		DataPointRange(31374 - 31374)
2768	348.7392	S	0.2105	(0.81, 0.33)-(0.84, 0.41)	DataPointRange(31375 - 31393)	0.2105	0.06	0.31	289.43
2769	348.9603	F	0.0223	(0.82, 0.39)		DataPointRange(31394 - 31395)
2770	348.9713	S	0.0221	(0.81, 0.37)-(0.84, 0.44)	DataPointRange(31396 - 31397)	0.0221	0.05	2.36	4.73
2771	349.0044	F	0.2541	(0.83, 0.43)		DataPointRange(31398 - 31420)
2772	349.2475	S	0.0110	(0.82, 0.41)-(0.84, 0.42)	DataPointRange(31421 - 31421)	0.0110	0.02	1.85	1.85
2773	349.2694	F	0.4199	(0.86, 0.44)		DataPointRange(31422 - 31459)
2774	349.6783	S	0.0110	(0.86, 0.45)-(0.84, 0.43)	DataPointRange(31460 - 31460)	0.0110	0.03	2.54	2.54
2775	349.7003	F	0.4528	(0.80, 0.42)		DataPointRange(31461 - 31501)
2776	350.1422	S	0.0441	(0.81, 0.41)-(0.56, 0.35)	DataPointRange(31502 - 31505)	0.0441	0.25	5.77	8.50
2777	350.1972	F	0.1438	(0.55, 0.36)		DataPointRange(31506 - 31518)
2778	350.3190	End					DataPoint(31517)
2779	350.3300	S	0.0444	(0.56, 0.37)-(0.39, 0.32)	DataPointRange(31519 - 31522)	0.0444	0.17	3.90	7.37
2780	350.3852	F	0.1655	(0.36, 0.29)		DataPointRange(31523 - 31537)
2781	350.5397	S	0.0442	(0.38, 0.29)-(0.21, 0.25)	DataPointRange(31538 - 31541)	0.0442	0.18	3.97	6.94
2782	350.5950	F	0.2430	(0.22, 0.23)		DataPointRange(31542 - 31563)
2783	350.7056	aa1n11s.bmp				DataPoint(31552)
2784	350.7828	Start					DataPoint(31559)
2785	350.8270	S	0.0110	(0.23, 0.22)-(0.19, 0.19)	DataPointRange(31564 - 31564)	0.0110	0.04	3.88	3.88
2786	350.8491	F	0.1546	(0.19, 0.14)		DataPointRange(31565 - 31578)
2787	350.9927	S	0.0220	(0.18, 0.15)-(0.25, 0.16)	DataPointRange(31579 - 31580)	0.0220	0.07	3.13	4.96
2788	351.0258	F	0.2210	(0.30, 0.16)		DataPointRange(31581 - 31600)
2789	351.2378	S	0.0201	(0.30, 0.17)-(0.41, 0.16)	DataPointRange(31601 - 31602)	0.0201	0.11	5.28	7.19
2790	351.2687	F	0.5414	(0.44, 0.16)		DataPointRange(31603 - 31651)
2791	351.7993	S	0.0220	(0.45, 0.16)-(0.53, 0.17)	DataPointRange(31652 - 31653)	0.0220	0.08	3.71	4.59
2792	351.8324	F	0.4086	(0.58, 0.18)		DataPointRange(31654 - 31690)
2793	352.2300	S	0.0219	(0.59, 0.18)-(0.66, 0.16)	DataPointRange(31691 - 31692)	0.0219	0.07	3.20	3.71
2794	352.2630	F	0.3317	(0.69, 0.17)		DataPointRange(31693 - 31722)
2795	352.5833	S	0.0114	(0.70, 0.17)-(0.74, 0.17)	DataPointRange(31723 - 31723)	0.0114	0.04	3.29	3.29
2796	352.6053	F	0.1658	(0.74, 0.17)		DataPointRange(31724 - 31738)
2797	352.7604	S	0.1432	(0.75, 0.19)-(0.20, 0.27)	DataPointRange(31739 - 31751)	0.1432	0.56	3.89	22.01
2798	352.9148	F	0.3203	(0.19, 0.27)		DataPointRange(31752 - 31780)
2799	353.2240	S	0.0333	(0.19, 0.27)-(0.32, 0.27)	DataPointRange(31781 - 31783)	0.0333	0.13	4.00	7.12
2800	353.2682	F	0.1989	(0.36, 0.26)		DataPointRange(31784 - 31801)
2801	353.4561	S	0.0110	(0.36, 0.27)-(0.32, 0.26)	DataPointRange(31802 - 31802)	0.0110	0.04	3.85	3.85
2802	353.4782	F	0.4085	(0.31, 0.26)		DataPointRange(31803 - 31839)
2803	353.8757	S	0.0331	(0.31, 0.25)-(0.46, 0.27)	DataPointRange(31840 - 31842)	0.0331	0.15	4.59	8.04
2804	353.9201	F	0.1766	(0.47, 0.25)		DataPointRange(31843 - 31858)
2805	354.0860	S	0.0329	(0.47, 0.26)-(0.62, 0.25)	DataPointRange(31859 - 31861)	0.0329	0.14	4.32	7.69
2806	354.1298	F	0.2430	(0.66, 0.25)		DataPointRange(31862 - 31883)
2807	354.3617	S	0.0111	(0.66, 0.25)-(0.70, 0.26)	DataPointRange(31884 - 31884)	0.0111	0.04	3.30	3.30
2808	354.3838	F	0.1879	(0.73, 0.26)		DataPointRange(31885 - 31901)
2809	354.5606	S	0.0223	(0.74, 0.26)-(0.79, 0.24)	DataPointRange(31902 - 31903)	0.0223	0.06	2.82	3.97
2810	354.5937	F	0.3537	(0.80, 0.24)		DataPointRange(31904 - 31935)
2811	354.9363	S	0.0444	(0.80, 0.25)-(0.53, 0.25)	DataPointRange(31936 - 31939)	0.0444	0.27	6.05	9.35
2812	354.9914	F	0.2319	(0.51, 0.25)		DataPointRange(31940 - 31960)
2813	355.2125	S	0.2207	(0.51, 0.27)-(0.43, 0.21)	DataPointRange(31961 - 31980)	0.2207	0.09	0.41	243.30
2814	355.4445	F	0.0218	(0.46, 0.20)		DataPointRange(31981 - 31982)
2815	355.4553	S	0.0110	(0.47, 0.20)-(0.46, 0.23)	DataPointRange(31983 - 31983)	0.0110	0.02	2.24	2.24
2816	355.4779	F	0.3420	(0.47, 0.25)		DataPointRange(31984 - 32014)
2817	355.8088	S	0.0334	(0.47, 0.23)-(0.58, 0.25)	DataPointRange(32015 - 32017)	0.0334	0.11	3.28	5.74
2818	355.8530	F	0.2429	(0.61, 0.25)		DataPointRange(32018 - 32039)
2819	356.0849	S	0.0333	(0.60, 0.25)-(0.46, 0.21)	DataPointRange(32040 - 32042)	0.0333	0.14	4.33	7.74
2820	356.1292	F	0.1877	(0.42, 0.20)		DataPointRange(32043 - 32059)
2821	356.3061	S	0.0440	(0.42, 0.20)-(0.23, 0.14)	DataPointRange(32060 - 32063)	0.0440	0.19	4.35	7.91
2822	356.3610	F	0.8948	(0.23, 0.15)		DataPointRange(32064 - 32142)
2823	356.5820	End					DataPoint(32084)
2824	356.9687	aa1n15.bmp				DataPoint(32119)
2825	357.0570	Start					DataPoint(32127)
2826	357.2448	S	0.0222	(0.18, 0.15)-(0.25, 0.15)	DataPointRange(32143 - 32144)	0.0222	0.07	3.13	4.76
2827	357.2779	F	0.1990	(0.28, 0.17)		DataPointRange(32145 - 32162)
2828	357.4656	S	0.0334	(0.28, 0.16)-(0.43, 0.18)	DataPointRange(32163 - 32165)	0.0334	0.15	4.51	8.05
2829	357.5100	F	0.1876	(0.46, 0.17)		DataPointRange(32166 - 32182)
2830	357.6868	S	0.0551	(0.49, 0.13)-(0.60, 0.59)	DataPointRange(32183 - 32187)	0.0551	0.36	6.62	11.40
2831	357.7531	F	0.0108	(0.57, 0.62)		DataPointRange(32188 - 32188)
2832	357.7531	S	0.0664	(0.57, 0.62)-(0.58, 0.19)	DataPointRange(32189 - 32194)	0.0664	0.32	4.78	7.64
2833	357.8304	F	0.3423	(0.59, 0.17)		DataPointRange(32195 - 32225)
2834	358.1616	S	0.0332	(0.59, 0.17)-(0.73, 0.17)	DataPointRange(32226 - 32228)	0.0332	0.14	4.25	5.76
2835	358.2060	F	0.2540	(0.74, 0.17)		DataPointRange(32229 - 32251)
2836	358.4487	S	0.0668	(0.74, 0.16)-(0.20, 0.23)	DataPointRange(32252 - 32257)	0.0668	0.54	8.11	12.21
2837	358.5261	F	0.3316	(0.18, 0.25)		DataPointRange(32258 - 32287)
2838	358.8469	S	0.0332	(0.19, 0.22)-(0.39, 0.25)	DataPointRange(32288 - 32290)	0.0332	0.21	6.18	8.38
2839	358.8908	F	0.3313	(0.42, 0.24)		DataPointRange(32291 - 32320)
2840	359.2110	S	0.0222	(0.43, 0.24)-(0.37, 0.24)	DataPointRange(32321 - 32322)	0.0222	0.05	2.39	3.78
2841	359.2445	F	0.1765	(0.33, 0.25)		DataPointRange(32323 - 32338)
2842	359.4101	S	0.0439	(0.34, 0.26)-(0.52, 0.23)	DataPointRange(32339 - 32342)	0.0439	0.19	4.24	7.80
2843	359.4652	F	0.1546	(0.56, 0.24)		DataPointRange(32343 - 32356)
2844	359.6089	S	0.0219	(0.56, 0.24)-(0.66, 0.24)	DataPointRange(32357 - 32358)	0.0219	0.10	4.43	5.06
2845	359.6422	F	0.1431	(0.66, 0.24)		DataPointRange(32359 - 32371)
2846	359.7743	S	0.0332	(0.65, 0.25)-(0.77, 0.24)	DataPointRange(32372 - 32374)	0.0332	0.11	3.39	6.41
2847	359.8185	F	0.4750	(0.82, 0.24)		DataPointRange(32375 - 32417)
2848	360.2825	S	0.0995	(0.85, 0.24)-(0.15, 0.32)	DataPointRange(32418 - 32426)	0.0995	0.70	7.02	11.74
2849	360.3930	F	0.2983	(0.15, 0.34)		DataPointRange(32427 - 32453)
2850	360.6801	S	0.0442	(0.14, 0.34)-(0.31, 0.32)	DataPointRange(32454 - 32457)	0.0442	0.17	3.78	6.90
2851	360.7357	F	0.2538	(0.33, 0.33)		DataPointRange(32458 - 32480)
2852	360.9784	S	0.0331	(0.34, 0.33)-(0.45, 0.34)	DataPointRange(32481 - 32483)	0.0331	0.11	3.31	7.23
2853	361.0226	F	0.1768	(0.45, 0.33)		DataPointRange(32484 - 32499)
2854	361.1883	S	0.0552	(0.46, 0.33)-(0.64, 0.35)	DataPointRange(32500 - 32504)	0.0552	0.18	3.34	6.07
2855	361.2546	F	0.3315	(0.66, 0.34)		DataPointRange(32505 - 32534)
2856	361.5748	S	0.0113	(0.64, 0.35)-(0.62, 0.33)	DataPointRange(32535 - 32535)	0.0113	0.03	2.51	2.51
2857	361.5972	F	0.2206	(0.60, 0.34)		DataPointRange(32536 - 32555)
2858	361.8068	S	0.0444	(0.59, 0.33)-(0.82, 0.35)	DataPointRange(32556 - 32559)	0.0444	0.23	5.19	6.50
2859	361.8625	F	0.3199	(0.83, 0.35)		DataPointRange(32560 - 32588)
2860	362.1714	S	0.0773	(0.82, 0.35)-(-0.92, -0.00)	DataPointRange(32589 - 32595)	0.0773	1.76	22.70	130.49
2861	362.2599	F	0.0109	(0.44, 0.56)		DataPointRange(32596 - 32596)
2862	362.2599	S	0.1991	(0.44, 0.56)-(0.83, 0.31)	DataPointRange(32597 - 32614)	0.1991	0.44	2.19	243.45
2863	362.4696	F	0.0223	(0.82, 0.31)		DataPointRange(32615 - 32616)
2864	362.4807	S	0.0112	(0.82, 0.30)-(0.83, 0.33)	DataPointRange(32617 - 32617)	0.0112	0.02	1.92	1.92
2865	362.5028	F	0.2541	(0.83, 0.36)		DataPointRange(32618 - 32640)
2866	362.7458	S	0.0446	(0.83, 0.35)-(0.68, 0.24)	DataPointRange(32641 - 32644)	0.0446	0.18	4.00	5.73
2867	362.8011	F	0.1987	(0.66, 0.23)		DataPointRange(32645 - 32662)
2868	362.9892	S	0.0549	(0.67, 0.24)-(0.95, -0.06)	DataPointRange(32663 - 32667)	0.0549	0.36	6.49	11.20
2869	363.0555	F	0.1654	(0.95, -0.07)		DataPointRange(32668 - 32682)
2870	363.2098	S	0.2653	(0.94, -0.07)-(-1.47, 0.75)	DataPointRange(32683 - 32706)	0.2653	2.49	9.38	228.73
2871	363.4860	F	0.0335	(-1.46, 0.74)		DataPointRange(32707 - 32709)
2872	363.5080	S	0.1215	(-1.47, 0.74)-(-1.47, 0.76)	DataPointRange(32710 - 32720)	0.1215	0.02	0.16	21.69
2873	363.6408	F	0.0220	(-1.46, 0.72)		DataPointRange(32721 - 32722)
2874	363.6516	S	0.0775	(-1.47, 0.81)-(-1.49, 0.97)	DataPointRange(32723 - 32729)	0.0775	0.12	1.51	41.51
2875	363.7400	F	0.0109	(-1.45, 0.67)		DataPointRange(32730 - 32730)
2876	363.7400	S	0.0109	(-1.45, 0.67)-(-1.50, 0.96)	DataPointRange(32731 - 32731)	0.0109	0.22	20.51	20.51
2877	363.7622	F	0.0110	(-1.48, 0.83)		DataPointRange(32732 - 32732)
2878	363.7622	S	0.0442	(-1.48, 0.83)-(-1.50, 0.95)	DataPointRange(32733 - 32736)	0.0442	0.09	2.11	18.11
2879	363.8174	F	0.0109	(-1.18, 1.15)		DataPointRange(32737 - 32737)
2880	363.8174	S	0.0440	(-1.18, 1.15)-(-1.47, 0.84)	DataPointRange(32738 - 32741)	0.0440	0.37	8.50	31.97
2881	363.8725	F	0.0223	(-1.48, 0.78)		DataPointRange(32742 - 32743)
2882	363.8837	S	0.0111	(-1.50, 0.86)-(-1.47, 0.73)	DataPointRange(32744 - 32744)	0.0111	0.10	9.39	9.39
2883	363.9057	F	0.0221	(-1.48, 0.87)		DataPointRange(32745 - 32746)
2884	363.9168	S	0.0551	(-1.46, 0.76)-(-1.50, 0.86)	DataPointRange(32747 - 32751)	0.0551	0.08	1.44	13.58
2885	363.9831	F	0.0109	(-1.50, 0.97)		DataPointRange(32752 - 32752)
2886	363.9831	S	0.0330	(-1.50, 0.97)-(-1.43, 0.71)	DataPointRange(32753 - 32755)	0.0330	0.21	6.30	22.84
2887	364.0272	F	0.0110	(-1.49, 0.87)		DataPointRange(32756 - 32756)
2888	364.0272	S	0.0110	(-1.49, 0.87)-(-1.44, 0.67)	DataPointRange(32757 - 32757)	0.0110	0.16	14.51	14.51
2889	364.0494	F	0.0666	(-1.44, 0.66)		DataPointRange(32758 - 32763)
2890	364.1045	S	0.0115	(-1.45, 0.66)-(-1.45, 0.70)	DataPointRange(32764 - 32764)	0.0115	0.03	2.41	2.41
2891	364.1267	F	0.0109	(-1.17, 1.10)		DataPointRange(32765 - 32765)
2892	364.1267	S	0.0109	(-1.17, 1.10)-(-1.45, 0.71)	DataPointRange(32766 - 32766)	0.0109	0.41	37.52	37.52
2893	364.1489	F	0.0329	(-1.45, 0.70)		DataPointRange(32767 - 32769)
2894	364.1707	S	0.0111	(-1.44, 0.71)-(-1.44, 0.71)	DataPointRange(32770 - 32770)	0.0111	0.00	0.42	0.42
2895	364.1928	F	0.0110	(-1.43, 0.65)		DataPointRange(32771 - 32771)
2896	364.1928	S	0.0110	(-1.43, 0.65)-(-1.44, 0.73)	DataPointRange(32772 - 32772)	0.0110	0.06	5.68	5.68
2897	364.2150	F	0.0111	(-1.48, 0.93)		DataPointRange(32773 - 32773)
2898	364.2150	S	0.0111	(-1.48, 0.93)-(-1.45, 0.72)	DataPointRange(32774 - 32774)	0.0111	0.16	13.96	13.96
2899	364.2370	F	0.0114	(-1.43, 0.64)		DataPointRange(32775 - 32775)
2900	364.2370	S	0.0222	(-1.43, 0.64)-(-1.44, 0.72)	DataPointRange(32776 - 32777)	0.0222	0.06	2.93	9.48
2901	364.2702	F	0.0112	(-1.50, 0.97)		DataPointRange(32778 - 32778)
2902	364.2702	S	0.0220	(-1.50, 0.97)-(-1.44, 0.70)	DataPointRange(32779 - 32780)	0.0220	0.21	9.59	19.47
2903	364.3034	F	0.0112	(-1.44, 0.75)		DataPointRange(32781 - 32781)
2904	364.3034	S	0.0112	(-1.44, 0.75)-(-1.44, 0.70)	DataPointRange(32782 - 32782)	0.0112	0.03	2.95	2.95
2905	364.3256	F	0.0220	(-1.43, 0.70)		DataPointRange(32783 - 32784)
2906	364.3367	S	0.0551	(-1.43, 0.72)-(-1.49, 0.99)	DataPointRange(32785 - 32789)	0.0551	0.22	3.91	22.01
2907	364.4027	F	0.0112	(-1.33, 0.56)		DataPointRange(32790 - 32790)
2908	364.4027	S	0.0331	(-1.33, 0.56)-(-1.44, 0.65)	DataPointRange(32791 - 32793)	0.0331	0.13	4.00	30.39
2909	364.4470	F	0.0109	(-1.48, 0.85)		DataPointRange(32794 - 32794)
2910	364.4470	S	0.0442	(-1.48, 0.85)-(-1.46, 0.84)	DataPointRange(32795 - 32798)	0.0442	0.02	0.52	20.86
2911	364.5022	F	0.0115	(-1.49, 0.98)		DataPointRange(32799 - 32799)
2912	364.5022	S	0.0115	(-1.49, 0.98)-(-1.46, 0.81)	DataPointRange(32800 - 32800)	0.0115	0.13	11.24	11.24
2913	364.5242	F	0.0225	(-1.45, 0.71)		DataPointRange(32801 - 32802)
2914	364.5354	S	0.0330	(-1.47, 0.76)-(-1.45, 0.60)	DataPointRange(32803 - 32805)	0.0330	0.12	3.61	3.86
2915	364.5796	F	0.0108	(-1.48, 0.77)		DataPointRange(32806 - 32806)
2916	364.5796	S	0.0108	(-1.48, 0.77)-(-1.44, 0.63)	DataPointRange(32807 - 32807)	0.0108	0.11	10.33	10.33
2917	364.6015	F	0.0114	(-1.32, 0.52)		DataPointRange(32808 - 32808)
2918	364.6015	S	0.0223	(-1.32, 0.52)-(-1.46, 0.71)	DataPointRange(32809 - 32810)	0.0223	0.20	9.12	15.63
2919	364.6346	F	0.0112	(-1.48, 0.87)		DataPointRange(32811 - 32811)
2920	364.6346	S	0.0554	(-1.48, 0.87)-(-1.47, 0.69)	DataPointRange(32812 - 32816)	0.0554	0.13	2.38	19.93
2921	364.7011	F	0.0219	(-1.48, 0.81)		DataPointRange(32817 - 32818)
2922	364.7123	S	0.0217	(-1.45, 0.67)-(-1.46, 0.75)	DataPointRange(32819 - 32820)	0.0217	0.06	2.90	16.68
2923	364.7452	End					DataPoint(32821)
2924	364.7452	F	0.0110	(-1.47, 0.71)		DataPointRange(32821 - 32821)
2925	364.7452	S	0.0772	(-1.47, 0.71)-(0.39, 0.20)	DataPointRange(32822 - 32828)	0.0772	1.91	24.69	232.34
2926	364.8335	F	0.2099	(0.41, 0.20)		DataPointRange(32829 - 32847)
2927	365.0323	S	0.0332	(0.41, 0.21)-(0.26, 0.19)	DataPointRange(32848 - 32850)	0.0332	0.15	4.64	7.97
2928	365.0766	F	0.1769	(0.24, 0.19)		DataPointRange(32851 - 32866)
2929	365.0987	aa1n3.bmp				DataPoint(32853)
2930	365.1430	Start					DataPoint(32857)
2931	365.2426	S	0.0219	(0.25, 0.19)-(0.18, 0.13)	DataPointRange(32867 - 32868)	0.0219	0.08	3.51	6.30
2932	365.2758	F	0.1874	(0.17, 0.13)		DataPointRange(32869 - 32885)
2933	365.4522	S	0.0222	(0.17, 0.13)-(0.24, 0.12)	DataPointRange(32886 - 32887)	0.0222	0.06	2.81	5.57
2934	365.4853	F	0.2098	(0.28, 0.14)		DataPointRange(32888 - 32906)
2935	365.6840	S	0.0221	(0.29, 0.13)-(0.37, 0.14)	DataPointRange(32907 - 32908)	0.0221	0.08	3.49	4.98
2936	365.7172	F	0.2320	(0.39, 0.15)		DataPointRange(32909 - 32929)
2937	365.9382	S	0.0337	(0.39, 0.15)-(0.47, 0.17)	DataPointRange(32930 - 32932)	0.0337	0.08	2.52	5.90
2938	365.9824	F	0.1327	(0.50, 0.16)		DataPointRange(32933 - 32944)
2939	366.1041	S	0.0552	(0.49, 0.17)-(0.54, 0.42)	DataPointRange(32945 - 32949)	0.0552	0.19	3.44	19.92
2940	366.1707	F	0.0107	(0.64, 0.44)		DataPointRange(32950 - 32950)
2941	366.1707	S	0.0438	(0.64, 0.44)-(0.64, 0.15)	DataPointRange(32951 - 32954)	0.0438	0.22	5.04	14.69
2942	366.2254	F	0.2982	(0.65, 0.18)		DataPointRange(32955 - 32981)
2943	366.5125	S	0.0223	(0.66, 0.18)-(0.72, 0.16)	DataPointRange(32982 - 32983)	0.0223	0.06	2.92	3.76
2944	366.5457	F	0.2652	(0.74, 0.16)		DataPointRange(32984 - 33007)
2945	366.7999	S	0.0774	(0.75, 0.17)-(0.20, 0.27)	DataPointRange(33008 - 33014)	0.0774	0.55	7.06	12.19
2946	366.8884	F	0.3756	(0.21, 0.25)		DataPointRange(33015 - 33048)
2947	367.2526	S	0.0332	(0.21, 0.26)-(0.35, 0.25)	DataPointRange(33049 - 33051)	0.0332	0.14	4.16	6.30
2948	367.2968	F	0.5306	(0.37, 0.26)		DataPointRange(33052 - 33099)
2949	367.8161	S	0.0220	(0.36, 0.27)-(0.46, 0.28)	DataPointRange(33100 - 33101)	0.0220	0.10	4.48	6.16
2950	367.8493	F	0.2983	(0.51, 0.26)		DataPointRange(33102 - 33128)
2951	368.1365	S	0.0332	(0.52, 0.25)-(0.65, 0.27)	DataPointRange(33129 - 33131)	0.0332	0.13	3.91	6.53
2952	368.1806	F	0.2101	(0.67, 0.26)		DataPointRange(33132 - 33150)
2953	368.3794	S	0.0221	(0.67, 0.25)-(0.73, 0.25)	DataPointRange(33151 - 33152)	0.0221	0.06	2.51	4.54
2954	368.4128	F	0.0993	(0.76, 0.25)		DataPointRange(33153 - 33161)
2955	368.5011	S	0.0332	(0.76, 0.26)-(0.59, 0.24)	DataPointRange(33162 - 33164)	0.0332	0.17	5.19	8.36
2956	368.5453	F	0.1323	(0.60, 0.25)		DataPointRange(33165 - 33176)
2957	368.6665	S	0.0111	(0.60, 0.25)-(0.58, 0.27)	DataPointRange(33177 - 33177)	0.0111	0.02	2.22	2.22
2958	368.6887	F	0.2652	(0.53, 0.26)		DataPointRange(33178 - 33201)
2959	368.9428	S	0.0446	(0.52, 0.26)-(0.72, 0.24)	DataPointRange(33202 - 33205)	0.0446	0.20	4.59	7.61
2960	368.9980	F	0.2652	(0.75, 0.24)		DataPointRange(33206 - 33229)
2961	369.2524	S	0.0219	(0.75, 0.25)-(0.80, 0.24)	DataPointRange(33230 - 33231)	0.0219	0.05	2.32	4.81
2962	369.2851	F	0.1326	(0.79, 0.25)		DataPointRange(33232 - 33243)
2963	369.4068	S	0.0662	(0.79, 0.25)-(0.22, 0.34)	DataPointRange(33244 - 33249)	0.0662	0.58	8.75	13.23
2964	369.4843	F	0.2870	(0.21, 0.35)		DataPointRange(33250 - 33275)
2965	369.7603	S	0.0329	(0.20, 0.34)-(0.33, 0.33)	DataPointRange(33276 - 33278)	0.0329	0.13	3.93	5.59
2966	369.8044	F	0.3425	(0.34, 0.34)		DataPointRange(33279 - 33309)
2967	370.1359	S	0.0445	(0.34, 0.34)-(0.54, 0.33)	DataPointRange(33310 - 33313)	0.0445	0.20	4.42	7.28
2968	370.1911	F	0.2982	(0.57, 0.34)		DataPointRange(33314 - 33340)
2969	370.4787	S	0.0218	(0.57, 0.34)-(0.62, 0.34)	DataPointRange(33341 - 33342)	0.0218	0.05	2.35	4.76
2970	370.5113	F	0.1439	(0.65, 0.33)		DataPointRange(33343 - 33355)
2971	370.6440	S	0.0222	(0.65, 0.33)-(0.71, 0.34)	DataPointRange(33356 - 33357)	0.0222	0.06	2.60	5.25
2972	370.6772	F	0.1768	(0.75, 0.33)		DataPointRange(33358 - 33373)
2973	370.8432	S	0.2648	(0.76, 0.28)-(0.84, 0.32)	DataPointRange(33374 - 33397)	0.2648	0.09	0.33	249.57
2974	371.1188	F	0.0226	(0.83, 0.29)		DataPointRange(33398 - 33399)
2975	371.1300	S	0.0114	(0.83, 0.30)-(0.83, 0.31)	DataPointRange(33400 - 33400)	0.0114	0.01	0.59	0.59
2976	371.1521	F	0.2983	(0.82, 0.36)		DataPointRange(33401 - 33427)
2977	371.4394	S	0.0666	(0.81, 0.37)-(0.26, 0.45)	DataPointRange(33428 - 33433)	0.0666	0.55	8.33	12.65
2978	371.5166	F	0.1326	(0.25, 0.43)		DataPointRange(33434 - 33445)
2979	371.6382	S	0.0222	(0.25, 0.44)-(0.16, 0.44)	DataPointRange(33446 - 33447)	0.0222	0.08	3.83	5.54
2980	371.6717	F	0.2093	(0.16, 0.46)		DataPointRange(33448 - 33466)
2981	371.8700	S	0.0332	(0.17, 0.46)-(0.33, 0.44)	DataPointRange(33467 - 33469)	0.0332	0.16	4.91	5.69
2982	371.9142	F	0.2319	(0.34, 0.42)		DataPointRange(33470 - 33490)
2983	372.1351	S	0.0221	(0.35, 0.43)-(0.43, 0.41)	DataPointRange(33491 - 33492)	0.0221	0.08	3.83	5.67
2984	372.1683	F	0.1657	(0.44, 0.41)		DataPointRange(33493 - 33507)
2985	372.3229	S	0.0332	(0.44, 0.41)-(0.22, 0.50)	DataPointRange(33508 - 33510)	0.0332	0.23	6.99	11.31
2986	372.3673	F	0.2651	(0.18, 0.56)		DataPointRange(33511 - 33534)
2987	372.6212	S	0.0220	(0.18, 0.57)-(0.27, 0.56)	DataPointRange(33535 - 33536)	0.0220	0.08	3.79	5.97
2988	372.6543	F	0.2762	(0.27, 0.53)		DataPointRange(33537 - 33561)
2989	372.9196	S	0.0218	(0.28, 0.54)-(0.37, 0.53)	DataPointRange(33562 - 33563)	0.0218	0.10	4.46	7.32
2990	372.9524	F	0.7183	(0.41, 0.53)		DataPointRange(33564 - 33628)
2991	373.6596	S	0.0221	(0.44, 0.52)-(0.33, 0.48)	DataPointRange(33629 - 33630)	0.0221	0.12	5.22	7.40
2992	373.6927	F	0.3978	(0.32, 0.47)		DataPointRange(33631 - 33666)
2993	374.0794	S	0.1987	(0.30, 0.42)-(0.40, 0.45)	DataPointRange(33667 - 33684)	0.1987	0.10	0.48	100.12
2994	374.2894	F	0.1657	(0.42, 0.45)		DataPointRange(33685 - 33699)
2995	374.4439	S	0.0222	(0.44, 0.45)-(0.39, 0.42)	DataPointRange(33700 - 33701)	0.0222	0.05	2.32	3.52
2996	374.4771	F	0.2982	(0.38, 0.38)		DataPointRange(33702 - 33728)
2997	374.5102	End					DataPoint(33705)
2998	374.7644	S	0.0439	(0.38, 0.37)-(0.28, 0.22)	DataPointRange(33729 - 33732)	0.0439	0.15	3.48	5.69
2999	374.8196	F	0.2096	(0.27, 0.21)		DataPointRange(33733 - 33751)
3000	374.8745	t1n9s.bmp				DataPoint(33738)
3001	374.9077	Start					DataPoint(33741)
3002	375.0181	S	0.0221	(0.28, 0.19)-(0.20, 0.15)	DataPointRange(33752 - 33753)	0.0221	0.08	3.84	6.27
3003	375.0515	F	0.3645	(0.18, 0.13)		DataPointRange(33754 - 33786)
3004	375.4049	S	0.0331	(0.17, 0.12)-(0.33, 0.16)	DataPointRange(33787 - 33789)	0.0331	0.16	4.75	6.84
3005	375.4489	F	0.2209	(0.37, 0.16)		DataPointRange(33790 - 33809)
3006	375.6589	S	0.0331	(0.37, 0.15)-(0.47, 0.16)	DataPointRange(33810 - 33812)	0.0331	0.10	2.99	4.70
3007	375.7030	F	0.1880	(0.48, 0.17)		DataPointRange(33813 - 33829)
3008	375.8799	S	0.0220	(0.49, 0.18)-(0.55, 0.17)	DataPointRange(33830 - 33831)	0.0220	0.06	2.57	3.57
3009	375.9133	F	0.2648	(0.56, 0.17)		DataPointRange(33832 - 33855)
3010	376.1669	S	0.0443	(0.56, 0.16)-(0.73, 0.15)	DataPointRange(33856 - 33859)	0.0443	0.17	3.78	6.02
3011	376.2224	F	0.2870	(0.73, 0.16)		DataPointRange(33860 - 33885)
3012	376.4983	S	0.0221	(0.74, 0.17)-(0.81, 0.16)	DataPointRange(33886 - 33887)	0.0221	0.07	3.36	6.30
3013	376.5315	F	0.2210	(0.84, 0.14)		DataPointRange(33888 - 33907)
3014	376.7416	S	0.0664	(0.84, 0.15)-(0.37, 0.25)	DataPointRange(33908 - 33913)	0.0664	0.48	7.20	11.79
3015	376.8191	F	0.1323	(0.33, 0.26)		DataPointRange(33914 - 33925)
3016	376.9403	S	0.0335	(0.34, 0.25)-(0.18, 0.25)	DataPointRange(33926 - 33928)	0.0335	0.16	4.73	7.70
3017	376.9846	F	0.3644	(0.15, 0.27)		DataPointRange(33929 - 33961)
3018	377.3379	S	0.0111	(0.16, 0.26)-(0.15, 0.27)	DataPointRange(33962 - 33962)	0.0111	0.02	1.47	1.47
3019	377.3601	F	0.1767	(0.12, 0.27)		DataPointRange(33963 - 33978)
3020	377.5256	S	0.0333	(0.11, 0.26)-(0.21, 0.25)	DataPointRange(33979 - 33981)	0.0333	0.10	3.07	5.66
3021	377.5698	F	0.3207	(0.25, 0.26)		DataPointRange(33982 - 34010)
3022	377.8792	S	0.0332	(0.26, 0.26)-(0.40, 0.26)	DataPointRange(34011 - 34013)	0.0332	0.14	4.27	7.50
3023	377.9235	F	0.2209	(0.41, 0.25)		DataPointRange(34014 - 34033)
3024	378.1333	S	0.0442	(0.41, 0.25)-(0.59, 0.25)	DataPointRange(34034 - 34037)	0.0442	0.19	4.21	7.53
3025	378.1886	F	0.2761	(0.62, 0.26)		DataPointRange(34038 - 34062)
3026	378.4538	S	0.0219	(0.62, 0.26)-(0.71, 0.27)	DataPointRange(34063 - 34064)	0.0219	0.09	3.97	6.83
3027	378.4867	F	0.4531	(0.74, 0.26)		DataPointRange(34065 - 34105)
3028	378.9288	S	0.1655	(0.75, 0.27)-(0.64, 0.23)	DataPointRange(34106 - 34120)	0.1655	0.12	0.70	196.34
3029	379.1054	F	0.0224	(0.66, 0.21)		DataPointRange(34121 - 34122)
3030	379.1166	S	0.0112	(0.65, 0.20)-(0.66, 0.26)	DataPointRange(34123 - 34123)	0.0112	0.04	3.48	3.48
3031	379.1385	F	0.4970	(0.66, 0.26)		DataPointRange(34124 - 34168)
3032	379.6244	S	0.0221	(0.67, 0.24)-(0.55, 0.25)	DataPointRange(34169 - 34170)	0.0221	0.11	5.19	6.31
3033	379.6575	F	0.1437	(0.55, 0.25)		DataPointRange(34171 - 34183)
3034	379.7900	S	0.0222	(0.55, 0.25)-(0.50, 0.24)	DataPointRange(34184 - 34185)	0.0222	0.05	2.45	4.36
3035	379.8232	F	0.2433	(0.49, 0.26)		DataPointRange(34186 - 34207)
3036	380.0558	S	0.0217	(0.48, 0.25)-(0.43, 0.20)	DataPointRange(34208 - 34209)	0.0217	0.07	3.02	4.45
3037	380.0885	F	0.2651	(0.42, 0.16)		DataPointRange(34210 - 34233)
3038	380.3423	S	0.0224	(0.41, 0.15)-(0.52, 0.16)	DataPointRange(34234 - 34235)	0.0224	0.11	4.89	6.30
3039	380.3757	F	0.2982	(0.57, 0.17)		DataPointRange(34236 - 34262)
3040	380.6629	S	0.0441	(0.57, 0.17)-(0.73, 0.24)	DataPointRange(34263 - 34266)	0.0441	0.17	3.87	5.71
3041	380.7184	F	0.3199	(0.74, 0.24)		DataPointRange(34267 - 34295)
3042	380.7954	End					DataPoint(34274)
3043	381.0275	S	0.0554	(0.74, 0.24)-(0.37, 0.25)	DataPointRange(34296 - 34300)	0.0554	0.37	6.68	8.75
3044	381.0938	F	0.2096	(0.35, 0.26)		DataPointRange(34301 - 34319)
3045	381.2261	t1n8.bmp					DataPoint(34313)
3046	381.2924	S	0.0331	(0.35, 0.25)-(0.19, 0.19)	DataPointRange(34320 - 34322)	0.0331	0.17	5.02	8.29
3047	381.3144	Start					DataPoint(34321)
3048	381.3366	F	0.1879	(0.18, 0.15)		DataPointRange(34323 - 34339)
3049	381.5135	S	0.0219	(0.17, 0.15)-(0.23, 0.16)	DataPointRange(34340 - 34341)	0.0219	0.06	2.78	4.75
3050	381.5464	F	0.2100	(0.27, 0.17)		DataPointRange(34342 - 34360)
3051	381.7452	S	0.0112	(0.26, 0.18)-(0.30, 0.16)	DataPointRange(34361 - 34361)	0.0112	0.04	3.82	3.82
3052	381.7673	F	0.2321	(0.34, 0.17)		DataPointRange(34362 - 34382)
3053	381.9884	S	0.0442	(0.34, 0.18)-(0.51, 0.17)	DataPointRange(34383 - 34386)	0.0442	0.17	3.93	5.87
3054	382.0437	F	0.2324	(0.54, 0.18)		DataPointRange(34387 - 34407)
3055	382.2646	S	0.0220	(0.54, 0.19)-(0.60, 0.16)	DataPointRange(34408 - 34409)	0.0220	0.06	2.72	4.82
3056	382.2978	F	0.2872	(0.62, 0.17)		DataPointRange(34410 - 34435)
3057	382.5739	S	0.0335	(0.62, 0.17)-(0.75, 0.17)	DataPointRange(34436 - 34438)	0.0335	0.14	4.06	5.51
3058	382.6181	F	0.3206	(0.76, 0.17)		DataPointRange(34439 - 34467)
3059	382.9274	S	0.0221	(0.76, 0.17)-(0.83, 0.17)	DataPointRange(34468 - 34469)	0.0221	0.07	3.23	5.58
3060	382.9606	F	0.1212	(0.84, 0.16)		DataPointRange(34470 - 34480)
3061	383.0711	S	0.0769	(0.84, 0.17)-(0.22, 0.25)	DataPointRange(34481 - 34487)	0.0769	0.62	8.11	15.92
3062	383.1592	F	0.1657	(0.21, 0.27)		DataPointRange(34488 - 34502)
3063	383.3139	S	0.0224	(0.21, 0.28)-(0.17, 0.29)	DataPointRange(34503 - 34504)	0.0224	0.05	2.20	4.22
3064	383.3470	F	0.2542	(0.16, 0.27)		DataPointRange(34505 - 34527)
3065	383.5899	S	0.0445	(0.15, 0.27)-(0.38, 0.27)	DataPointRange(34528 - 34531)	0.0445	0.22	4.96	7.40
3066	383.6452	F	0.2874	(0.41, 0.26)		DataPointRange(34532 - 34557)
3067	383.9214	S	0.0333	(0.42, 0.24)-(0.54, 0.26)	DataPointRange(34558 - 34560)	0.0333	0.12	3.72	6.58
3068	383.9658	F	0.2760	(0.55, 0.26)		DataPointRange(34561 - 34585)
3069	384.2308	S	0.0220	(0.55, 0.25)-(0.46, 0.26)	DataPointRange(34586 - 34587)	0.0220	0.09	4.10	6.36
3070	384.2643	F	0.1657	(0.43, 0.25)		DataPointRange(34588 - 34602)
3071	384.4187	S	0.0332	(0.43, 0.25)-(0.54, 0.25)	DataPointRange(34603 - 34605)	0.0332	0.11	3.37	8.04
3072	384.4633	F	0.3088	(0.58, 0.25)		DataPointRange(34606 - 34633)
3073	384.7610	S	0.0333	(0.58, 0.26)-(0.70, 0.24)	DataPointRange(34634 - 34636)	0.0333	0.12	3.65	6.29
3074	384.8053	F	0.1548	(0.73, 0.24)		DataPointRange(34637 - 34650)
3075	384.9490	S	0.0662	(0.74, 0.25)-(0.22, 0.35)	DataPointRange(34651 - 34656)	0.0662	0.52	7.87	10.83
3076	385.0267	F	0.3751	(0.22, 0.37)		DataPointRange(34657 - 34690)
3077	385.3910	S	0.0218	(0.22, 0.37)-(0.31, 0.31)	DataPointRange(34691 - 34692)	0.0218	0.10	4.50	6.99
3078	385.4244	F	0.1980	(0.33, 0.34)		DataPointRange(34693 - 34710)
3079	385.6115	S	0.0330	(0.33, 0.35)-(0.16, 0.40)	DataPointRange(34711 - 34713)	0.0330	0.17	5.08	7.45
3080	385.6559	F	0.2098	(0.15, 0.38)		DataPointRange(34714 - 34732)
3081	385.8546	S	0.0553	(0.15, 0.39)-(0.46, 0.36)	DataPointRange(34733 - 34737)	0.0553	0.31	5.65	9.74
3082	385.9209	F	0.2098	(0.48, 0.36)		DataPointRange(34738 - 34756)
3083	386.1197	S	0.0221	(0.49, 0.36)-(0.58, 0.32)	DataPointRange(34757 - 34758)	0.0221	0.10	4.50	7.81
3084	386.1530	F	0.3423	(0.62, 0.36)		DataPointRange(34759 - 34789)
3085	386.4843	S	0.0110	(0.60, 0.36)-(0.58, 0.36)	DataPointRange(34790 - 34790)	0.0110	0.02	1.60	1.60
3086	386.5063	F	0.1767	(0.57, 0.34)		DataPointRange(34791 - 34806)
3087	386.6720	S	0.0110	(0.57, 0.36)-(0.57, 0.37)	DataPointRange(34807 - 34807)	0.0110	0.01	0.88	0.88
3088	386.6941	F	0.0109	(0.51, 0.19)		DataPointRange(34808 - 34808)
3089	386.6941	S	0.0441	(0.51, 0.19)-(0.78, -0.08)	DataPointRange(34809 - 34812)	0.0441	0.34	7.70	120.39
3090	386.7495	F	0.0108	(0.76, -0.06)		DataPointRange(34813 - 34813)
3091	386.7495	S	0.1543	(0.76, -0.06)-(0.49, 0.31)	DataPointRange(34814 - 34827)	0.1543	0.39	2.51	243.02
3092	386.9153	F	0.0217	(0.49, 0.28)		DataPointRange(34828 - 34829)
3093	386.9258	S	0.0112	(0.48, 0.26)-(0.50, 0.30)	DataPointRange(34830 - 34830)	0.0112	0.04	3.20	3.20
3094	386.9483	F	0.2208	(0.50, 0.32)		DataPointRange(34831 - 34850)
3095	387.1582	S	0.0219	(0.50, 0.30)-(0.41, 0.29)	DataPointRange(34851 - 34852)	0.0219	0.09	4.00	6.38
3096	387.1910	F	0.5194	(0.40, 0.28)		DataPointRange(34853 - 34899)
3097	387.6993	S	0.0220	(0.40, 0.29)-(0.48, 0.31)	DataPointRange(34900 - 34901)	0.0220	0.08	3.68	5.08
3098	387.7325	F	0.3425	(0.52, 0.33)		DataPointRange(34902 - 34932)
3099	388.0639	S	0.0221	(0.52, 0.33)-(0.57, 0.35)	DataPointRange(34933 - 34934)	0.0221	0.05	2.37	4.06
3100	388.0969	F	0.2319	(0.58, 0.35)		DataPointRange(34935 - 34955)
3101	388.3178	S	0.0331	(0.59, 0.34)-(0.43, 0.35)	DataPointRange(34956 - 34958)	0.0331	0.15	4.67	7.78
3102	388.3620	F	0.1877	(0.43, 0.32)		DataPointRange(34959 - 34975)
3103	388.5389	S	0.0439	(0.42, 0.33)-(0.26, 0.22)	DataPointRange(34976 - 34979)	0.0439	0.18	4.11	8.10
3104	388.5938	F	0.6298	(0.25, 0.19)		DataPointRange(34980 - 35036)
3105	388.7153	End					DataPoint(34991)
3106	389.2124	S	0.0333	(0.25, 0.19)-(0.14, 0.15)	DataPointRange(35037 - 35039)	0.0333	0.11	3.34	5.06
3107	389.2457	aa1n2.bmp				DataPoint(35039)
3108	389.2589	F	0.1748	(0.16, 0.15)		DataPointRange(35040 - 35055)
3109	389.4006	Start					DataPoint(35053)
3110	389.4224	S	0.0221	(0.15, 0.16)-(0.25, 0.16)	DataPointRange(35056 - 35057)	0.0221	0.10	4.38	6.70
3111	389.4556	F	0.1987	(0.27, 0.17)		DataPointRange(35058 - 35075)
3112	389.6432	S	0.0331	(0.29, 0.16)-(0.40, 0.18)	DataPointRange(35076 - 35078)	0.0331	0.11	3.47	7.00
3113	389.6874	F	0.2652	(0.42, 0.19)		DataPointRange(35079 - 35102)
3114	389.9416	S	0.0222	(0.43, 0.19)-(0.52, 0.19)	DataPointRange(35103 - 35104)	0.0222	0.09	3.99	5.11
3115	389.9748	F	0.2207	(0.52, 0.18)		DataPointRange(35105 - 35124)
3116	390.1846	S	0.0220	(0.51, 0.19)-(0.61, 0.19)	DataPointRange(35125 - 35126)	0.0220	0.09	4.21	4.32
3117	390.2177	F	0.2430	(0.62, 0.18)		DataPointRange(35127 - 35148)
3118	390.4496	S	0.0111	(0.61, 0.18)-(0.65, 0.19)	DataPointRange(35149 - 35149)	0.0111	0.04	3.57	3.57
3119	390.4718	F	0.1216	(0.68, 0.18)		DataPointRange(35150 - 35160)
3120	390.5824	S	0.0660	(0.69, 0.17)-(0.22, 0.24)	DataPointRange(35161 - 35166)	0.0660	0.47	7.13	11.10
3121	390.6598	F	0.3202	(0.21, 0.25)		DataPointRange(35167 - 35195)
3122	390.9688	S	0.0222	(0.21, 0.26)-(0.31, 0.24)	DataPointRange(35196 - 35197)	0.0222	0.10	4.56	5.99
3123	391.0020	F	0.2098	(0.32, 0.25)		DataPointRange(35198 - 35216)
3124	391.2008	S	0.0220	(0.32, 0.26)-(0.42, 0.26)	DataPointRange(35217 - 35218)	0.0220	0.10	4.59	5.83
3125	391.2339	F	0.2101	(0.43, 0.27)		DataPointRange(35219 - 35237)
3126	391.4328	S	0.0223	(0.43, 0.27)-(0.53, 0.26)	DataPointRange(35238 - 35239)	0.0223	0.10	4.52	5.67
3127	391.4659	F	0.2876	(0.57, 0.27)		DataPointRange(35240 - 35265)
3128	391.7422	S	0.0222	(0.57, 0.25)-(0.62, 0.26)	DataPointRange(35266 - 35267)	0.0222	0.05	2.41	5.39
3129	391.7754	F	0.1878	(0.65, 0.26)		DataPointRange(35268 - 35284)
3130	391.9521	S	0.0221	(0.65, 0.26)-(0.73, 0.25)	DataPointRange(35285 - 35286)	0.0221	0.08	3.80	5.21
3131	391.9853	F	0.0992	(0.74, 0.26)		DataPointRange(35287 - 35295)
3132	392.0734	S	0.0778	(0.73, 0.28)-(0.19, 0.35)	DataPointRange(35296 - 35302)	0.0778	0.55	7.09	11.09
3133	392.1618	F	0.2320	(0.16, 0.36)		DataPointRange(35303 - 35323)
3134	392.3829	S	0.0219	(0.16, 0.37)-(0.23, 0.35)	DataPointRange(35324 - 35325)	0.0219	0.07	3.01	6.03
3135	392.4159	F	0.1547	(0.28, 0.34)		DataPointRange(35326 - 35339)
3136	392.5594	S	0.0222	(0.28, 0.35)-(0.39, 0.35)	DataPointRange(35340 - 35341)	0.0222	0.11	5.08	6.84
3137	392.5927	F	0.2208	(0.41, 0.33)		DataPointRange(35342 - 35361)
3138	392.8025	S	0.0332	(0.40, 0.34)-(0.59, 0.35)	DataPointRange(35362 - 35364)	0.0332	0.19	5.72	8.40
3139	392.8470	F	0.2205	(0.61, 0.34)		DataPointRange(35365 - 35384)
3140	393.0565	S	0.0332	(0.61, 0.35)-(0.74, 0.35)	DataPointRange(35385 - 35387)	0.0332	0.13	3.99	6.10
3141	393.1008	F	0.2099	(0.79, 0.34)		DataPointRange(35388 - 35406)
3142	393.2998	S	0.0109	(0.80, 0.35)-(0.77, 0.35)	DataPointRange(35407 - 35407)	0.0109	0.02	2.20	2.20
3143	393.3219	F	0.0442	(0.74, 0.33)		DataPointRange(35408 - 35411)
3144	393.3550	S	0.0111	(0.75, 0.33)-(0.74, 0.37)	DataPointRange(35412 - 35412)	0.0111	0.03	2.70	2.70
3145	393.3772	F	0.0110	(0.76, 0.28)		DataPointRange(35413 - 35413)
3146	393.3772	S	0.0881	(0.76, 0.28)-(-0.93, 0.18)	DataPointRange(35414 - 35421)	0.0881	1.69	19.22	195.60
3147	393.4767	F	0.0107	(-0.94, 0.18)		DataPointRange(35422 - 35422)
3148	393.4767	S	0.0218	(-0.94, 0.18)-(1.86, -0.36)	DataPointRange(35423 - 35424)	0.0218	2.82	129.49	252.76
3149	393.5096	F	0.0110	(1.87, -0.22)		DataPointRange(35425 - 35425)
3150	393.5096	S	0.0110	(1.87, -0.22)-(1.86, -0.36)	DataPointRange(35426 - 35426)	0.0110	0.11	10.19	10.19
3151	393.5317	F	0.0220	(1.86, -0.36)		DataPointRange(35427 - 35428)
3152	393.5430	S	0.1102	(1.86, -0.36)-(0.88, 0.32)	DataPointRange(35429 - 35438)	0.1102	1.10	9.96	119.30
3153	393.6642	F	0.0115	(0.87, 0.30)		DataPointRange(35439 - 35439)
3154	393.6642	S	0.0222	(0.87, 0.30)-(0.89, 0.38)	DataPointRange(35440 - 35441)	0.0222	0.07	3.06	5.90
3155	393.6974	F	0.0444	(0.88, 0.38)		DataPointRange(35442 - 35445)
3156	393.7306	S	0.0772	(0.88, 0.38)-(0.23, 0.45)	DataPointRange(35446 - 35452)	0.0772	0.66	8.54	12.30
3157	393.8188	F	0.2216	(0.20, 0.45)		DataPointRange(35453 - 35472)
3158	394.0319	S	0.0085	(0.21, 0.43)-(0.20, 0.45)	DataPointRange(35473 - 35473)	0.0085	0.02	1.81	1.81
3159	394.0507	F	0.0442	(0.20, 0.47)		DataPointRange(35474 - 35477)
3160	394.0839	S	0.0223	(0.20, 0.47)-(0.26, 0.42)	DataPointRange(35478 - 35479)	0.0223	0.07	3.06	6.12
3161	394.1171	F	0.3314	(0.29, 0.43)		DataPointRange(35480 - 35509)
3162	394.4374	S	0.0552	(0.31, 0.43)-(1.87, -0.26)	DataPointRange(35510 - 35514)	0.0552	1.64	29.74	239.94
3163	394.5041	F	0.0106	(1.87, -0.29)		DataPointRange(35515 - 35515)
3164	394.5041	S	0.1321	(1.87, -0.29)-(0.36, 0.35)	DataPointRange(35516 - 35527)	0.1321	1.59	12.02	113.58
3165	394.6472	F	0.0224	(0.36, 0.30)		DataPointRange(35528 - 35529)
3166	394.6583	S	0.0113	(0.36, 0.30)-(0.37, 0.36)	DataPointRange(35530 - 35530)	0.0113	0.04	3.50	3.50
3167	394.6806	F	0.1548	(0.38, 0.36)		DataPointRange(35531 - 35544)
3168	394.8241	S	0.0113	(0.37, 0.39)-(0.35, 0.35)	DataPointRange(35545 - 35545)	0.0113	0.04	3.16	3.16
3169	394.8461	F	0.2651	(0.34, 0.34)		DataPointRange(35546 - 35569)
3170	395.1002	S	0.0335	(0.33, 0.33)-(0.30, 0.23)	DataPointRange(35570 - 35572)	0.0335	0.08	2.53	5.07
3171	395.1445	F	0.1986	(0.30, 0.20)		DataPointRange(35573 - 35590)
3172	395.3324	S	0.0328	(0.30, 0.19)-(0.15, 0.16)	DataPointRange(35591 - 35593)	0.0328	0.15	4.54	7.03
3173	395.3764	F	0.7290	(0.16, 0.15)		DataPointRange(35594 - 35659)
3174	395.5531	End					DataPoint(35610)
3175	395.9618	t1n6s.bmp				DataPoint(35647)
3176	396.0170	Start					DataPoint(35652)
3177	396.0944	S	0.0330	(0.16, 0.16)-(0.26, 0.15)	DataPointRange(35660 - 35662)	0.0330	0.11	3.21	4.62
3178	396.1384	F	0.2214	(0.26, 0.15)		DataPointRange(35663 - 35682)
3179	396.3485	S	0.0219	(0.27, 0.15)-(0.33, 0.17)	DataPointRange(35683 - 35684)	0.0219	0.06	2.70	4.56
3180	396.3815	F	0.2876	(0.33, 0.17)		DataPointRange(35685 - 35710)
3181	396.6579	S	0.0112	(0.33, 0.16)-(0.37, 0.16)	DataPointRange(35711 - 35711)	0.0112	0.04	3.34	3.34
3182	396.6800	F	0.1878	(0.38, 0.17)		DataPointRange(35712 - 35728)
3183	396.8569	S	0.0332	(0.39, 0.16)-(0.52, 0.18)	DataPointRange(35729 - 35731)	0.0332	0.13	3.92	6.92
3184	396.9008	F	0.0111	(0.54, 0.18)		DataPointRange(35732 - 35732)
3185	396.9008	S	0.0111	(0.54, 0.18)-(0.53, 0.18)	DataPointRange(35733 - 35733)	0.0111	0.01	0.57	0.57
3186	396.9233	F	0.0331	(0.50, 0.21)		DataPointRange(35734 - 35736)
3187	396.9449	S	0.0115	(0.49, 0.22)-(0.54, 0.18)	DataPointRange(35737 - 35737)	0.0115	0.06	4.98	4.98
3188	396.9670	F	0.2985	(0.54, 0.18)		DataPointRange(35738 - 35764)
3189	397.2544	S	0.0222	(0.53, 0.18)-(0.60, 0.16)	DataPointRange(35765 - 35766)	0.0222	0.07	2.96	4.47
3190	397.2875	F	0.1544	(0.62, 0.17)		DataPointRange(35767 - 35780)
3191	397.4310	S	0.0554	(0.61, 0.17)-(0.21, 0.23)	DataPointRange(35781 - 35785)	0.0554	0.40	7.24	11.02
3192	397.4972	F	0.3869	(0.18, 0.24)		DataPointRange(35786 - 35820)
3193	397.8730	S	0.0111	(0.19, 0.23)-(0.21, 0.24)	DataPointRange(35821 - 35821)	0.0111	0.02	1.79	1.79
3194	397.8950	F	0.4085	(0.25, 0.26)		DataPointRange(35822 - 35858)
3195	398.2925	S	0.0331	(0.23, 0.27)-(0.36, 0.25)	DataPointRange(35859 - 35861)	0.0331	0.14	4.11	6.31
3196	398.3368	F	0.3093	(0.38, 0.25)		DataPointRange(35862 - 35889)
3197	398.6352	S	0.0329	(0.37, 0.25)-(0.51, 0.26)	DataPointRange(35890 - 35892)	0.0329	0.14	4.16	6.95
3198	398.6796	F	0.1763	(0.53, 0.26)		DataPointRange(35893 - 35908)
3199	398.8453	S	0.0519	(0.53, 0.27)-(0.62, 0.25)	DataPointRange(35909 - 35911)	0.0519	0.09	1.76	6.18
3200	398.9039	F	0.2062	(0.63, 0.24)		DataPointRange(35912 - 35930)
3201	399.0991	S	0.0553	(0.64, 0.26)-(0.20, 0.35)	DataPointRange(35931 - 35935)	0.0553	0.44	7.95	11.69
3202	399.1651	F	0.4418	(0.19, 0.35)		DataPointRange(35936 - 35975)
3203	399.5959	S	0.0110	(0.20, 0.36)-(0.17, 0.32)	DataPointRange(35976 - 35976)	0.0110	0.04	4.02	4.02
3204	399.6181	F	0.1877	(0.15, 0.30)		DataPointRange(35977 - 35993)
3205	399.7948	S	0.0110	(0.14, 0.30)-(0.17, 0.31)	DataPointRange(35994 - 35994)	0.0110	0.03	2.57	2.57
3206	399.8169	F	0.2102	(0.20, 0.36)		DataPointRange(35995 - 36013)
3207	400.0163	S	0.0439	(0.21, 0.37)-(0.41, 0.34)	DataPointRange(36014 - 36017)	0.0439	0.20	4.65	10.77
3208	400.0708	F	0.3540	(0.42, 0.35)		DataPointRange(36018 - 36049)
3209	400.4136	S	0.0330	(0.42, 0.34)-(0.31, 0.29)	DataPointRange(36050 - 36052)	0.0330	0.11	3.45	6.61
3210	400.4576	F	0.1767	(0.30, 0.27)		DataPointRange(36053 - 36068)
3211	400.6232	S	0.0223	(0.30, 0.29)-(0.22, 0.27)	DataPointRange(36069 - 36070)	0.0223	0.09	3.87	6.18
3212	400.6564	F	0.1876	(0.20, 0.26)		DataPointRange(36071 - 36087)
3213	400.8330	S	0.0777	(0.21, 0.25)-(1.86, -0.36)	DataPointRange(36088 - 36094)	0.0777	1.71	21.98	169.88
3214	400.9216	F	0.0772	(1.86, -0.33)		DataPointRange(36095 - 36101)
3215	400.9878	S	0.1435	(1.86, -0.36)-(0.33, 0.34)	DataPointRange(36102 - 36114)	0.1435	1.62	11.26	193.07
3216	401.1426	F	0.0441	(0.33, 0.28)		DataPointRange(36115 - 36118)
3217	401.1757	S	0.0110	(0.32, 0.26)-(0.34, 0.29)	DataPointRange(36119 - 36119)	0.0110	0.03	2.58	2.58
3218	401.1977	F	0.1545	(0.35, 0.34)		DataPointRange(36120 - 36133)
3219	401.3414	S	0.0332	(0.34, 0.35)-(0.47, 0.35)	DataPointRange(36134 - 36136)	0.0332	0.13	3.98	7.15
3220	401.3854	F	0.2431	(0.46, 0.34)		DataPointRange(36137 - 36158)
3221	401.6175	S	0.0331	(0.45, 0.34)-(0.31, 0.25)	DataPointRange(36159 - 36161)	0.0331	0.16	4.88	7.46
3222	401.6617	F	0.1988	(0.30, 0.23)		DataPointRange(36162 - 36179)
3223	401.8494	S	0.0225	(0.29, 0.22)-(0.24, 0.17)	DataPointRange(36180 - 36181)	0.0225	0.06	2.74	3.63
3224	401.8827	F	0.8284	(0.25, 0.17)		DataPointRange(36182 - 36256)
3225	402.0703	End					DataPoint(36199)
3226	402.5011	t1n5s.bmp				DataPoint(36238)
3227	402.5894	Start					DataPoint(36246)
3228	402.7004	S	0.0219	(0.25, 0.19)-(0.31, 0.17)	DataPointRange(36257 - 36258)	0.0219	0.06	2.89	4.79
3229	402.7333	F	0.2319	(0.30, 0.17)		DataPointRange(36259 - 36279)
3230	402.9540	S	0.0332	(0.31, 0.17)-(0.43, 0.17)	DataPointRange(36280 - 36282)	0.0332	0.12	3.61	5.41
3231	402.9984	F	0.2648	(0.45, 0.18)		DataPointRange(36283 - 36306)
3232	403.2522	S	0.0220	(0.45, 0.18)-(0.51, 0.19)	DataPointRange(36307 - 36308)	0.0220	0.06	2.79	4.28
3233	403.2854	F	0.2652	(0.55, 0.19)		DataPointRange(36309 - 36332)
3234	403.5395	S	0.0222	(0.55, 0.20)-(0.61, 0.19)	DataPointRange(36333 - 36334)	0.0222	0.06	2.89	4.94
3235	403.5726	F	0.2542	(0.64, 0.18)		DataPointRange(36335 - 36357)
3236	403.8156	S	0.0220	(0.64, 0.16)-(0.71, 0.16)	DataPointRange(36358 - 36359)	0.0220	0.07	3.04	5.65
3237	403.8487	F	0.1988	(0.75, 0.17)		DataPointRange(36360 - 36377)
3238	404.0366	S	0.0220	(0.76, 0.16)-(0.83, 0.18)	DataPointRange(36378 - 36379)	0.0220	0.07	3.16	4.90
3239	404.0696	F	0.1992	(0.82, 0.18)		DataPointRange(36380 - 36397)
3240	404.2578	S	0.0665	(0.83, 0.18)-(0.22, 0.25)	DataPointRange(36398 - 36403)	0.0665	0.61	9.21	14.63
3241	404.3349	F	0.1655	(0.20, 0.26)		DataPointRange(36404 - 36418)
3242	404.4894	S	0.0110	(0.20, 0.27)-(0.16, 0.27)	DataPointRange(36419 - 36419)	0.0110	0.04	3.79	3.79
3243	404.5116	F	0.2099	(0.13, 0.27)		DataPointRange(36420 - 36438)
3244	404.7105	S	0.0331	(0.13, 0.27)-(0.28, 0.26)	DataPointRange(36439 - 36441)	0.0331	0.15	4.58	7.63
3245	404.7549	F	0.2647	(0.30, 0.27)		DataPointRange(36442 - 36465)
3246	405.0085	S	0.0111	(0.29, 0.24)-(0.33, 0.26)	DataPointRange(36466 - 36466)	0.0111	0.04	3.35	3.35
3247	405.0307	F	0.2432	(0.35, 0.26)		DataPointRange(36467 - 36488)
3248	405.2628	S	0.0332	(0.35, 0.25)-(0.50, 0.26)	DataPointRange(36489 - 36491)	0.0332	0.15	4.52	6.79
3249	405.3069	F	0.1435	(0.51, 0.27)		DataPointRange(36492 - 36504)
3250	405.4395	S	0.0664	(0.52, 0.27)-(-0.20, 1.76)	DataPointRange(36505 - 36510)	0.0664	1.33	20.06	71.96
3251	405.5172	F	0.0106	(-0.20, 1.92)		DataPointRange(36511 - 36511)
3252	405.5172	S	0.0991	(-0.20, 1.92)-(0.59, 0.18)	DataPointRange(36512 - 36520)	0.0991	1.52	15.36	60.66
3253	405.6272	F	0.0113	(0.57, 0.14)		DataPointRange(36521 - 36521)
3254	405.6272	S	0.0113	(0.57, 0.14)-(0.61, 0.21)	DataPointRange(36522 - 36522)	0.0113	0.06	5.14	5.14
3255	405.6497	F	0.1986	(0.59, 0.26)		DataPointRange(36523 - 36540)
3256	405.8372	S	0.0221	(0.58, 0.26)-(0.68, 0.27)	DataPointRange(36541 - 36542)	0.0221	0.09	4.24	4.40
3257	405.8702	F	0.1216	(0.68, 0.27)		DataPointRange(36543 - 36553)
3258	405.9811	S	0.0549	(0.68, 0.27)-(0.23, 0.33)	DataPointRange(36554 - 36558)	0.0549	0.45	8.12	10.35
3259	406.0475	F	0.3751	(0.21, 0.35)		DataPointRange(36559 - 36592)
3260	406.4117	S	0.0220	(0.21, 0.36)-(0.27, 0.33)	DataPointRange(36593 - 36594)	0.0220	0.06	2.93	5.83
3261	406.4452	F	0.2426	(0.30, 0.35)		DataPointRange(36595 - 36616)
3262	406.6770	S	0.0440	(0.29, 0.34)-(0.58, 0.29)	DataPointRange(36617 - 36620)	0.0440	0.29	6.68	8.99
3263	406.7319	F	0.2873	(0.63, 0.29)		DataPointRange(36621 - 36646)
3264	407.0084	S	0.0108	(0.63, 0.29)-(0.62, 0.31)	DataPointRange(36647 - 36647)	0.0108	0.02	1.83	1.83
3265	407.0302	F	0.3315	(0.57, 0.35)		DataPointRange(36648 - 36677)
3266	407.3506	S	0.0442	(0.55, 0.35)-(0.77, 0.37)	DataPointRange(36678 - 36681)	0.0442	0.22	4.96	8.21
3267	407.4058	F	0.2431	(0.78, 0.35)		DataPointRange(36682 - 36703)
3268	407.6383	S	0.0106	(0.78, 0.34)-(0.75, 0.34)	DataPointRange(36704 - 36704)	0.0106	0.03	2.97	2.97
3269	407.6601	F	0.2869	(0.71, 0.34)		DataPointRange(36705 - 36730)
3270	407.9360	S	0.0773	(0.71, 0.33)-(0.81, 0.33)	DataPointRange(36731 - 36737)	0.0773	0.10	1.33	8.88
3271	408.0244	F	0.0111	(0.80, 0.32)		DataPointRange(36738 - 36738)
3272	408.0244	S	0.0111	(0.80, 0.32)-(0.77, 0.31)	DataPointRange(36739 - 36739)	0.0111	0.03	2.52	2.52
3273	408.0466	F	0.0772	(0.75, 0.35)		DataPointRange(36740 - 36746)
3274	408.1128	S	0.0662	(0.74, 0.36)-(0.24, 0.45)	DataPointRange(36747 - 36752)	0.0662	0.51	7.70	13.28
3275	408.1901	F	0.1765	(0.21, 0.48)		DataPointRange(36753 - 36768)
3276	408.3556	S	0.0110	(0.22, 0.51)-(0.17, 0.48)	DataPointRange(36769 - 36769)	0.0110	0.05	4.54	4.54
3277	408.3777	F	0.1877	(0.18, 0.47)		DataPointRange(36770 - 36786)
3278	408.5545	S	0.0331	(0.17, 0.48)-(0.34, 0.43)	DataPointRange(36787 - 36789)	0.0331	0.17	5.19	7.31
3279	408.5985	F	0.1997	(0.36, 0.43)		DataPointRange(36790 - 36807)
3280	408.7866	S	0.0222	(0.37, 0.43)-(0.43, 0.44)	DataPointRange(36808 - 36809)	0.0222	0.06	2.53	4.30
3281	408.8197	F	0.2320	(0.45, 0.43)		DataPointRange(36810 - 36830)
3282	409.0408	S	0.0326	(0.46, 0.44)-(0.59, 0.40)	DataPointRange(36831 - 36833)	0.0326	0.14	4.24	8.71
3283	409.0846	F	0.2543	(0.61, 0.42)		DataPointRange(36834 - 36856)
3284	409.3282	S	0.0329	(0.63, 0.44)-(0.76, 0.45)	DataPointRange(36857 - 36859)	0.0329	0.14	4.13	5.80
3285	409.3718	F	0.3316	(0.75, 0.43)		DataPointRange(36860 - 36889)
3286	409.6928	S	0.0217	(0.75, 0.42)-(0.80, 0.42)	DataPointRange(36890 - 36891)	0.0217	0.05	2.38	5.36
3287	409.7254	F	0.1327	(0.82, 0.43)		DataPointRange(36892 - 36903)
3288	409.8470	S	0.1109	(0.80, 0.43)-(0.14, 0.43)	DataPointRange(36904 - 36913)	0.1109	0.66	5.98	24.83
3289	409.9685	F	0.0112	(0.13, 0.43)		DataPointRange(36914 - 36914)
3290	409.9685	S	0.0223	(0.13, 0.43)-(0.19, 0.56)	DataPointRange(36915 - 36916)	0.0223	0.12	5.33	8.70
3291	410.0017	F	0.3425	(0.20, 0.57)		DataPointRange(36917 - 36947)
3292	410.3331	S	0.0331	(0.20, 0.58)-(0.39, 0.56)	DataPointRange(36948 - 36950)	0.0331	0.19	5.65	9.64
3293	410.3773	F	0.4085	(0.40, 0.54)		DataPointRange(36951 - 36987)
3294	410.7749	S	0.0220	(0.40, 0.54)-(0.45, 0.53)	DataPointRange(36988 - 36989)	0.0220	0.05	2.46	4.43
3295	410.8080	F	0.3534	(0.48, 0.52)		DataPointRange(36990 - 37021)
3296	411.1505	S	0.0219	(0.49, 0.53)-(0.43, 0.52)	DataPointRange(37022 - 37023)	0.0219	0.07	3.16	5.79
3297	411.1840	F	0.1652	(0.39, 0.48)		DataPointRange(37024 - 37038)
3298	411.3382	S	0.0446	(0.39, 0.47)-(0.26, 0.36)	DataPointRange(37039 - 37042)	0.0446	0.16	3.53	5.81
3299	411.3935	F	0.1437	(0.27, 0.33)		DataPointRange(37043 - 37055)
3300	411.5260	S	0.0441	(0.28, 0.33)-(0.22, 0.16)	DataPointRange(37056 - 37059)	0.0441	0.13	3.06	5.80
3301	411.5810	F	0.5757	(0.23, 0.15)		DataPointRange(37060 - 37111)
3302	411.6476	End					DataPoint(37066)
3303	412.0783	aa1n4.bmp				DataPoint(37105)
3304	412.1512	S	0.0155	(0.23, 0.15)-(0.15, 0.17)	DataPointRange(37112 - 37113)	0.0155	0.08	5.17	5.75
3305	412.1779	F	0.2208	(0.15, 0.15)		DataPointRange(37114 - 37133)
3306	412.1887	Start					DataPoint(37115)
3307	412.3876	S	0.0331	(0.16, 0.14)-(0.31, 0.16)	DataPointRange(37134 - 37136)	0.0331	0.15	4.48	6.92
3308	412.4318	F	0.2538	(0.34, 0.17)		DataPointRange(37137 - 37159)
3309	412.6747	S	0.0109	(0.34, 0.17)-(0.36, 0.18)	DataPointRange(37160 - 37160)	0.0109	0.02	2.02	2.02
3310	412.6968	F	0.1437	(0.41, 0.17)		DataPointRange(37161 - 37173)
3311	412.8293	S	0.1878	(0.41, 0.18)-(0.53, 0.16)	DataPointRange(37174 - 37190)	0.1878	0.13	0.69	257.83
3312	413.0283	F	0.0112	(0.50, 0.11)		DataPointRange(37191 - 37191)
3313	413.0283	S	0.0329	(0.50, 0.11)-(0.53, 0.19)	DataPointRange(37192 - 37194)	0.0329	0.07	2.13	10.79
3314	413.0725	F	0.1988	(0.53, 0.19)		DataPointRange(37195 - 37212)
3315	413.2603	S	0.0221	(0.52, 0.18)-(0.60, 0.19)	DataPointRange(37213 - 37214)	0.0221	0.08	3.72	5.58
3316	413.2935	F	0.2318	(0.59, 0.18)		DataPointRange(37215 - 37235)
3317	413.5143	S	0.0332	(0.58, 0.17)-(0.70, 0.17)	DataPointRange(37236 - 37238)	0.0332	0.12	3.59	6.47
3318	413.5586	F	0.2870	(0.74, 0.16)		DataPointRange(37239 - 37264)
3319	413.8345	S	0.0222	(0.73, 0.16)-(0.81, 0.16)	DataPointRange(37265 - 37266)	0.0222	0.08	3.64	4.97
3320	413.8677	F	0.1658	(0.82, 0.16)		DataPointRange(37267 - 37281)
3321	414.0224	S	0.2430	(0.81, 0.16)-(0.11, 0.21)	DataPointRange(37282 - 37303)	0.2430	0.70	2.89	185.23
3322	414.2763	F	0.0111	(0.10, 0.14)		DataPointRange(37304 - 37304)
3323	414.2763	S	0.0223	(0.10, 0.14)-(0.12, 0.27)	DataPointRange(37305 - 37306)	0.0223	0.09	4.19	6.98
3324	414.3096	F	0.1879	(0.13, 0.28)		DataPointRange(37307 - 37323)
3325	414.4864	S	0.0221	(0.14, 0.27)-(0.25, 0.28)	DataPointRange(37324 - 37325)	0.0221	0.11	4.87	7.14
3326	414.5196	F	0.2208	(0.24, 0.26)		DataPointRange(37326 - 37345)
3327	414.7297	S	0.0219	(0.25, 0.27)-(0.31, 0.27)	DataPointRange(37346 - 37347)	0.0219	0.06	2.91	4.50
3328	414.7628	F	0.1990	(0.34, 0.26)		DataPointRange(37348 - 37365)
3329	414.9503	S	0.0222	(0.34, 0.24)-(0.43, 0.25)	DataPointRange(37366 - 37367)	0.0222	0.10	4.32	7.65
3330	414.9835	F	0.2319	(0.48, 0.26)		DataPointRange(37368 - 37388)
3331	415.2043	S	0.0332	(0.48, 0.25)-(0.61, 0.26)	DataPointRange(37389 - 37391)	0.0332	0.13	3.84	5.14
3332	415.2486	F	0.2434	(0.65, 0.25)		DataPointRange(37392 - 37413)
3333	415.4804	S	0.0222	(0.65, 0.24)-(0.61, 0.25)	DataPointRange(37414 - 37415)	0.0222	0.05	2.08	4.39
3334	415.5137	F	0.2429	(0.59, 0.25)		DataPointRange(37416 - 37437)
3335	415.7456	S	0.0223	(0.59, 0.26)-(0.70, 0.28)	DataPointRange(37438 - 37439)	0.0223	0.11	4.75	6.39
3336	415.7789	F	0.0662	(0.70, 0.25)		DataPointRange(37440 - 37445)
3337	415.8342	S	0.0109	(0.70, 0.24)-(0.70, 0.25)	DataPointRange(37446 - 37446)	0.0109	0.00	0.07	0.07
3338	415.8563	F	0.0110	(0.65, 0.31)		DataPointRange(37447 - 37447)
3339	415.8563	S	0.0110	(0.65, 0.31)-(0.70, 0.25)	DataPointRange(37448 - 37448)	0.0110	0.06	5.85	5.85
3340	415.8783	F	0.0552	(0.70, 0.24)		DataPointRange(37449 - 37453)
3341	415.9225	S	0.0662	(0.71, 0.24)-(0.19, 0.34)	DataPointRange(37454 - 37459)	0.0662	0.52	7.90	12.11
3342	415.9994	F	0.3211	(0.17, 0.36)		DataPointRange(37460 - 37488)
3343	416.3091	S	0.0222	(0.18, 0.36)-(0.24, 0.34)	DataPointRange(37489 - 37490)	0.0222	0.06	2.89	5.23
3344	416.3423	F	0.2427	(0.25, 0.35)		DataPointRange(37491 - 37512)
3345	416.5740	S	0.0221	(0.26, 0.36)-(0.20, 0.36)	DataPointRange(37513 - 37514)	0.0221	0.06	2.52	4.89
3346	416.6073	F	0.1326	(0.21, 0.35)		DataPointRange(37515 - 37526)
3347	416.7289	S	0.0330	(0.21, 0.35)-(0.35, 0.35)	DataPointRange(37527 - 37529)	0.0330	0.15	4.47	8.31
3348	416.7730	F	0.2649	(0.41, 0.34)		DataPointRange(37530 - 37553)
3349	417.0269	S	0.0225	(0.42, 0.34)-(0.50, 0.35)	DataPointRange(37554 - 37555)	0.0225	0.08	3.74	6.81
3350	417.0600	F	0.2544	(0.51, 0.35)		DataPointRange(37556 - 37578)
3351	417.3031	S	0.0222	(0.51, 0.35)-(0.61, 0.35)	DataPointRange(37579 - 37580)	0.0222	0.11	4.74	6.26
3352	417.3364	F	0.2210	(0.64, 0.35)		DataPointRange(37581 - 37600)
3353	417.5463	S	0.0329	(0.65, 0.37)-(0.78, 0.35)	DataPointRange(37601 - 37603)	0.0329	0.13	4.06	6.65
3354	417.5903	F	0.3757	(0.80, 0.35)		DataPointRange(37604 - 37637)
3355	417.9552	S	0.0770	(0.80, 0.35)-(0.18, 0.43)	DataPointRange(37638 - 37644)	0.0770	0.62	8.11	12.89
3356	418.0435	F	0.3201	(0.18, 0.45)		DataPointRange(37645 - 37673)
3357	418.3524	S	0.0334	(0.18, 0.46)-(0.34, 0.43)	DataPointRange(37674 - 37676)	0.0334	0.17	5.00	7.54
3358	418.3968	F	0.2100	(0.36, 0.42)		DataPointRange(37677 - 37695)
3359	418.5957	S	0.0332	(0.36, 0.43)-(0.47, 0.43)	DataPointRange(37696 - 37698)	0.0332	0.11	3.19	5.79
3360	418.6403	F	0.2425	(0.48, 0.42)		DataPointRange(37699 - 37720)
3361	418.8723	S	0.0327	(0.48, 0.42)-(0.61, 0.42)	DataPointRange(37721 - 37723)	0.0327	0.13	3.97	5.79
3362	418.9160	F	0.2538	(0.64, 0.43)		DataPointRange(37724 - 37746)
3363	419.1590	S	0.0218	(0.63, 0.41)-(0.69, 0.41)	DataPointRange(37747 - 37748)	0.0218	0.06	2.65	5.22
3364	419.1920	F	0.3425	(0.74, 0.42)		DataPointRange(37749 - 37779)
3365	419.5235	S	0.0221	(0.74, 0.42)-(0.79, 0.41)	DataPointRange(37780 - 37781)	0.0221	0.05	2.25	4.26
3366	419.5568	F	0.0882	(0.80, 0.43)		DataPointRange(37782 - 37789)
3367	419.6339	S	0.2430	(0.80, 0.43)-(0.15, 0.52)	DataPointRange(37790 - 37811)	0.2430	0.66	2.71	243.90
3368	419.8879	F	0.0447	(0.15, 0.49)		DataPointRange(37812 - 37815)
3369	419.9211	S	0.0115	(0.14, 0.48)-(0.15, 0.52)	DataPointRange(37816 - 37816)	0.0115	0.03	2.86	2.86
3370	419.9434	F	0.1551	(0.16, 0.56)		DataPointRange(37817 - 37830)
3371	420.0870	S	0.0331	(0.16, 0.57)-(0.25, 0.52)	DataPointRange(37831 - 37833)	0.0331	0.11	3.18	6.60
3372	420.1310	F	0.3977	(0.24, 0.52)		DataPointRange(37834 - 37869)
3373	420.5176	S	0.0331	(0.24, 0.56)-(0.34, 0.49)	DataPointRange(37870 - 37872)	0.0331	0.11	3.46	5.59
3374	420.5619	F	0.2209	(0.35, 0.52)		DataPointRange(37873 - 37892)
3375	420.7718	S	0.0223	(0.35, 0.53)-(0.42, 0.51)	DataPointRange(37893 - 37894)	0.0223	0.08	3.39	6.40
3376	420.8049	F	0.2651	(0.46, 0.51)		DataPointRange(37895 - 37918)
3377	421.0588	S	0.0112	(0.47, 0.49)-(0.50, 0.52)	DataPointRange(37919 - 37919)	0.0112	0.04	3.47	3.47
3378	421.0809	F	0.4527	(0.51, 0.53)		DataPointRange(37920 - 37958)
3379	421.5226	S	0.0224	(0.52, 0.52)-(0.56, 0.52)	DataPointRange(37959 - 37960)	0.0224	0.05	2.05	3.11
3380	421.5559	F	0.2763	(0.62, 0.53)		DataPointRange(37961 - 37985)
3381	421.8211	S	0.0331	(0.63, 0.54)-(0.48, 0.40)	DataPointRange(37986 - 37988)	0.0331	0.18	5.42	7.17
3382	421.8652	F	0.3203	(0.48, 0.42)		DataPointRange(37989 - 38017)
3383	422.1747	S	0.0329	(0.48, 0.43)-(0.59, 0.53)	DataPointRange(38018 - 38020)	0.0329	0.13	3.86	5.68
3384	422.2187	F	0.2216	(0.60, 0.54)		DataPointRange(38021 - 38040)
3385	422.4288	S	0.0548	(0.59, 0.54)-(0.36, 0.27)	DataPointRange(38041 - 38045)	0.0548	0.30	5.53	10.19
3386	422.4948	F	0.1325	(0.37, 0.25)		DataPointRange(38046 - 38057)
3387	422.6163	S	0.0332	(0.37, 0.26)-(0.26, 0.17)	DataPointRange(38058 - 38060)	0.0332	0.12	3.70	6.48
3388	422.6605	F	0.5633	(0.24, 0.16)		DataPointRange(38061 - 38111)
3389	422.7158	End					DataPoint(38066)
3390	423.0582	t1n6.bmp					DataPoint(38097)
3391	423.1135	Start					DataPoint(38102)
3392	423.2128	S	0.0110	(0.23, 0.18)-(0.22, 0.16)	DataPointRange(38112 - 38112)	0.0110	0.02	1.93	1.93
3393	423.2353	F	0.1433	(0.19, 0.15)		DataPointRange(38113 - 38125)
3394	423.3676	S	0.0222	(0.20, 0.15)-(0.24, 0.15)	DataPointRange(38126 - 38127)	0.0222	0.05	2.17	3.86
3395	423.4011	F	0.2980	(0.28, 0.17)		DataPointRange(38128 - 38154)
3396	423.6877	S	0.0332	(0.29, 0.18)-(0.43, 0.18)	DataPointRange(38155 - 38157)	0.0332	0.14	4.26	8.16
3397	423.7322	F	0.3534	(0.47, 0.18)		DataPointRange(38158 - 38189)
3398	424.0748	S	0.0219	(0.47, 0.17)-(0.52, 0.16)	DataPointRange(38190 - 38191)	0.0219	0.05	2.28	4.46
3399	424.1078	F	0.4417	(0.57, 0.18)		DataPointRange(38192 - 38231)
3400	424.5385	S	0.1765	(0.57, 0.17)-(0.60, 0.17)	DataPointRange(38232 - 38247)	0.1765	0.03	0.14	26.56
3401	424.7262	F	0.1546	(0.61, 0.19)		DataPointRange(38248 - 38261)
3402	424.8697	S	0.0225	(0.61, 0.20)-(0.71, 0.18)	DataPointRange(38262 - 38263)	0.0225	0.09	4.19	6.57
3403	424.9029	F	0.2432	(0.74, 0.18)		DataPointRange(38264 - 38285)
3404	425.1350	S	0.0220	(0.75, 0.18)-(0.80, 0.17)	DataPointRange(38286 - 38287)	0.0220	0.05	2.29	4.90
3405	425.2268	F	0.1731	(0.81, 0.17)		DataPointRange(38288 - 38303)
3406	425.3890	S	0.0773	(0.81, 0.18)-(0.17, 0.29)	DataPointRange(38304 - 38310)	0.0773	0.65	8.42	13.65
3407	425.4782	F	0.4187	(0.16, 0.28)		DataPointRange(38311 - 38348)
3408	425.8858	S	0.0331	(0.17, 0.28)-(0.29, 0.28)	DataPointRange(38349 - 38351)	0.0331	0.12	3.69	7.52
3409	425.9301	F	0.1991	(0.31, 0.26)		DataPointRange(38352 - 38369)
3410	426.1181	S	0.0225	(0.31, 0.24)-(0.40, 0.24)	DataPointRange(38370 - 38371)	0.0225	0.10	4.32	7.25
3411	426.1512	F	0.2210	(0.41, 0.25)		DataPointRange(38372 - 38391)
3412	426.3610	S	0.0333	(0.42, 0.25)-(0.57, 0.26)	DataPointRange(38392 - 38394)	0.0333	0.16	4.72	7.72
3413	426.4057	F	0.2868	(0.60, 0.25)		DataPointRange(38395 - 38420)
3414	426.6816	S	0.0223	(0.60, 0.25)-(0.69, 0.25)	DataPointRange(38421 - 38422)	0.0223	0.09	3.92	5.76
3415	426.7146	F	0.1877	(0.69, 0.25)		DataPointRange(38423 - 38439)
3416	426.8913	S	0.0552	(0.69, 0.25)-(0.27, 0.34)	DataPointRange(38440 - 38444)	0.0552	0.42	7.59	11.97
3417	426.9573	F	0.1882	(0.25, 0.35)		DataPointRange(38445 - 38461)
3418	427.1347	S	0.0216	(0.25, 0.33)-(0.19, 0.35)	DataPointRange(38462 - 38463)	0.0216	0.06	2.80	5.99
3419	427.2228	F	0.0998	(0.17, 0.37)		DataPointRange(38464 - 38472)
3420	427.3109	S	0.1110	(0.17, 0.39)-(1.87, -0.26)	DataPointRange(38473 - 38482)	0.1110	1.77	15.97	188.23
3421	427.4326	F	0.0111	(1.87, -0.26)		DataPointRange(38483 - 38483)
3422	427.4326	S	0.0886	(1.87, -0.26)-(0.22, 0.33)	DataPointRange(38484 - 38491)	0.0886	1.71	19.30	187.03
3423	427.5320	F	0.0225	(0.22, 0.30)		DataPointRange(38492 - 38493)
3424	427.5431	S	0.0114	(0.22, 0.29)-(0.23, 0.35)	DataPointRange(38494 - 38494)	0.0114	0.04	3.54	3.54
3425	427.5653	F	0.2983	(0.23, 0.34)		DataPointRange(38495 - 38521)
3426	427.8523	S	0.0221	(0.23, 0.33)-(0.28, 0.35)	DataPointRange(38522 - 38523)	0.0221	0.05	2.44	4.57
3427	427.8857	F	0.1985	(0.27, 0.38)		DataPointRange(38524 - 38541)
3428	428.0730	S	0.0222	(0.27, 0.38)-(0.22, 0.28)	DataPointRange(38542 - 38543)	0.0222	0.09	4.20	4.36
3429	428.1062	F	0.5525	(0.22, 0.25)		DataPointRange(38544 - 38593)
3430	428.1284	End					DataPoint(38546)
3431	428.4711	aa1n12s.bmp				DataPoint(38577)
3432	428.5370	Start					DataPoint(38583)
3433	428.6476	S	0.0111	(0.21, 0.23)-(0.21, 0.19)	DataPointRange(38594 - 38594)	0.0111	0.03	2.71	2.71
3434	428.6698	F	0.1765	(0.21, 0.17)		DataPointRange(38595 - 38610)
3435	428.8355	S	0.0220	(0.21, 0.16)-(0.26, 0.16)	DataPointRange(38611 - 38612)	0.0220	0.05	2.44	5.41
3436	428.8685	F	0.5744	(0.34, 0.17)		DataPointRange(38613 - 38663)
3437	429.4319	S	0.0110	(0.37, 0.17)-(0.40, 0.16)	DataPointRange(38664 - 38664)	0.0110	0.04	3.37	3.37
3438	429.4543	F	0.2539	(0.44, 0.17)		DataPointRange(38665 - 38687)
3439	429.6970	S	0.0332	(0.44, 0.17)-(0.56, 0.17)	DataPointRange(38688 - 38690)	0.0332	0.11	3.44	5.74
3440	429.7413	F	0.2651	(0.61, 0.18)		DataPointRange(38691 - 38714)
3441	429.9953	S	0.0226	(0.62, 0.18)-(0.68, 0.17)	DataPointRange(38715 - 38716)	0.0226	0.07	2.89	3.98
3442	430.0284	F	0.2317	(0.70, 0.17)		DataPointRange(38717 - 38737)
3443	430.2494	S	0.0663	(0.70, 0.15)-(0.18, 0.26)	DataPointRange(38738 - 38743)	0.0663	0.53	7.95	10.91
3444	430.3264	F	0.2320	(0.17, 0.27)		DataPointRange(38744 - 38764)
3445	430.5478	S	0.0220	(0.17, 0.28)-(0.22, 0.26)	DataPointRange(38765 - 38766)	0.0220	0.04	2.01	4.47
3446	430.5806	F	0.2987	(0.24, 0.25)		DataPointRange(38767 - 38793)
3447	430.8681	S	0.0112	(0.25, 0.25)-(0.23, 0.26)	DataPointRange(38794 - 38794)	0.0112	0.02	2.01	2.01
3448	430.8901	F	0.2982	(0.21, 0.26)		DataPointRange(38795 - 38821)
3449	431.1773	S	0.0443	(0.21, 0.26)-(0.38, 0.26)	DataPointRange(38822 - 38825)	0.0443	0.17	3.83	6.91
3450	431.2324	F	0.2099	(0.40, 0.25)		DataPointRange(38826 - 38844)
3451	431.4312	S	0.0221	(0.40, 0.25)-(0.47, 0.25)	DataPointRange(38845 - 38846)	0.0221	0.07	3.25	5.45
3452	431.4644	F	0.2652	(0.51, 0.25)		DataPointRange(38847 - 38870)
3453	431.7185	S	0.0995	(0.51, 0.26)-(1.86, -0.36)	DataPointRange(38871 - 38879)	0.0995	1.42	14.28	263.15
3454	431.8291	F	0.0221	(1.86, -0.34)		DataPointRange(38880 - 38881)
3455	431.8405	S	0.1432	(1.86, -0.36)-(0.47, 0.24)	DataPointRange(38882 - 38894)	0.1432	1.46	10.17	202.60
3456	431.9945	F	0.0884	(0.50, 0.27)		DataPointRange(38895 - 38902)
3457	432.0723	S	0.0218	(0.51, 0.26)-(0.57, 0.27)	DataPointRange(38903 - 38904)	0.0218	0.05	2.49	5.33
3458	432.1054	F	0.1765	(0.60, 0.26)		DataPointRange(38905 - 38920)
3459	432.2712	S	0.0439	(0.60, 0.25)-(0.22, 0.37)	DataPointRange(38921 - 38924)	0.0439	0.39	8.80	12.18
3460	432.3262	F	0.3092	(0.19, 0.36)		DataPointRange(38925 - 38952)
3461	432.6243	S	0.0220	(0.18, 0.36)-(0.25, 0.35)	DataPointRange(38953 - 38954)	0.0220	0.07	3.00	4.73
3462	432.6575	F	0.1656	(0.27, 0.35)		DataPointRange(38955 - 38969)
3463	432.8121	S	0.0441	(0.28, 0.34)-(0.45, 0.34)	DataPointRange(38970 - 38973)	0.0441	0.17	3.91	8.11
3464	432.8678	F	0.3088	(0.47, 0.34)		DataPointRange(38974 - 39001)
3465	433.1654	S	0.0332	(0.47, 0.34)-(0.59, 0.33)	DataPointRange(39002 - 39004)	0.0332	0.12	3.69	6.44
3466	433.2096	F	0.2653	(0.63, 0.35)		DataPointRange(39005 - 39028)
3467	433.4642	S	0.0217	(0.63, 0.34)-(0.69, 0.34)	DataPointRange(39029 - 39030)	0.0217	0.06	2.93	5.45
3468	433.4970	F	0.2539	(0.71, 0.34)		DataPointRange(39031 - 39053)
3469	433.7400	S	0.0663	(0.72, 0.32)-(0.22, 0.46)	DataPointRange(39054 - 39059)	0.0663	0.51	7.65	13.35
3470	433.8175	F	0.4637	(0.18, 0.45)		DataPointRange(39060 - 39101)
3471	434.2702	S	0.0223	(0.18, 0.45)-(0.29, 0.43)	DataPointRange(39102 - 39103)	0.0223	0.10	4.70	6.81
3472	434.3033	F	0.2980	(0.31, 0.43)		DataPointRange(39104 - 39130)
3473	434.5903	S	0.0220	(0.31, 0.43)-(0.40, 0.40)	DataPointRange(39131 - 39132)	0.0220	0.09	4.13	6.36
3474	434.6234	F	0.3317	(0.42, 0.42)		DataPointRange(39133 - 39162)
3475	434.9440	S	0.0221	(0.41, 0.42)-(0.53, 0.42)	DataPointRange(39163 - 39164)	0.0221	0.11	5.20	6.99
3476	434.9773	F	0.2318	(0.56, 0.44)		DataPointRange(39165 - 39185)
3477	435.1981	S	0.0225	(0.57, 0.42)-(0.68, 0.44)	DataPointRange(39186 - 39187)	0.0225	0.11	4.93	6.43
3478	435.2313	F	0.2212	(0.68, 0.42)		DataPointRange(39188 - 39207)
3479	435.4411	S	0.0221	(0.67, 0.42)-(0.61, 0.43)	DataPointRange(39208 - 39209)	0.0221	0.06	2.55	4.77
3480	435.4743	F	0.2428	(0.58, 0.43)		DataPointRange(39210 - 39231)
3481	435.7060	S	0.0331	(0.58, 0.40)-(0.70, 0.40)	DataPointRange(39232 - 39234)	0.0331	0.12	3.69	6.21
3482	435.7502	F	0.1769	(0.72, 0.42)		DataPointRange(39235 - 39250)
3483	435.9163	S	0.0661	(0.71, 0.41)-(0.20, 0.52)	DataPointRange(39251 - 39256)	0.0661	0.52	7.83	11.42
3484	435.9934	F	0.1877	(0.21, 0.54)		DataPointRange(39257 - 39273)
3485	436.1700	S	0.0221	(0.21, 0.54)-(0.15, 0.54)	DataPointRange(39274 - 39275)	0.0221	0.07	3.02	6.37
3486	436.2033	F	0.2651	(0.15, 0.56)		DataPointRange(39276 - 39299)
3487	436.4574	S	0.0219	(0.16, 0.55)-(0.24, 0.53)	DataPointRange(39300 - 39301)	0.0219	0.08	3.53	6.46
3488	436.4904	F	0.4641	(0.22, 0.53)		DataPointRange(39302 - 39343)
3489	436.9435	S	0.0550	(0.21, 0.52)-(0.26, 0.26)	DataPointRange(39344 - 39348)	0.0550	0.20	3.69	5.36
3490	437.0099	F	0.1985	(0.27, 0.24)		DataPointRange(39349 - 39366)
3491	437.1974	S	0.0220	(0.27, 0.23)-(0.22, 0.18)	DataPointRange(39367 - 39368)	0.0220	0.07	3.10	5.54
3492	437.2305	F	0.8063	(0.22, 0.17)		DataPointRange(39369 - 39440)
3493	437.3632	End					DataPoint(39381)
3494	437.7166	aa1n19s.bmp				DataPoint(39413)
3495	437.8932	Start					DataPoint(39428)
3496	438.0258	S	0.0331	(0.20, 0.16)-(0.31, 0.17)	DataPointRange(39441 - 39443)	0.0331	0.12	3.54	5.66
3497	438.0699	F	0.2653	(0.32, 0.16)		DataPointRange(39444 - 39467)
3498	438.3242	S	0.0224	(0.31, 0.17)-(0.40, 0.16)	DataPointRange(39468 - 39469)	0.0224	0.09	4.10	5.46
3499	438.3574	F	0.3644	(0.42, 0.17)		DataPointRange(39470 - 39502)
3500	438.7109	S	0.0440	(0.42, 0.17)-(0.21, 0.17)	DataPointRange(39503 - 39506)	0.0440	0.21	4.69	8.56
3501	438.7661	F	0.1545	(0.21, 0.16)		DataPointRange(39507 - 39520)
3502	438.9100	S	0.0327	(0.21, 0.18)-(0.38, 0.16)	DataPointRange(39521 - 39523)	0.0327	0.17	5.10	6.36
3503	438.9535	F	0.2548	(0.41, 0.17)		DataPointRange(39524 - 39546)
3504	439.1968	S	0.0222	(0.41, 0.17)-(0.35, 0.17)	DataPointRange(39547 - 39548)	0.0222	0.06	2.75	5.91
3505	439.2298	F	0.2320	(0.35, 0.17)		DataPointRange(39549 - 39569)
3506	439.4508	S	0.0332	(0.35, 0.15)-(0.49, 0.16)	DataPointRange(39570 - 39572)	0.0332	0.14	4.32	7.16
3507	439.4950	F	0.3865	(0.52, 0.16)		DataPointRange(39573 - 39607)
3508	439.8705	S	0.0332	(0.51, 0.16)-(0.64, 0.16)	DataPointRange(39608 - 39610)	0.0332	0.13	3.92	5.79
3509	439.9147	F	0.3204	(0.65, 0.17)		DataPointRange(39611 - 39639)
3510	440.2240	S	0.0667	(0.65, 0.16)-(0.20, 0.16)	DataPointRange(39640 - 39645)	0.0667	0.45	6.67	16.23
3511	440.3018	F	0.2093	(0.27, 0.16)		DataPointRange(39646 - 39664)
3512	440.5005	S	0.0219	(0.27, 0.19)-(0.38, 0.16)	DataPointRange(39665 - 39666)	0.0219	0.11	5.10	6.15
3513	440.5336	F	0.1766	(0.43, 0.17)		DataPointRange(39667 - 39682)
3514	440.6992	S	0.0335	(0.43, 0.16)-(0.63, 0.19)	DataPointRange(39683 - 39685)	0.0335	0.20	5.97	8.36
3515	440.7433	F	0.2541	(0.67, 0.18)		DataPointRange(39686 - 39708)
3516	440.9862	S	0.0222	(0.68, 0.18)-(0.76, 0.18)	DataPointRange(39709 - 39710)	0.0222	0.08	3.52	6.92
3517	441.0194	F	0.1878	(0.80, 0.17)		DataPointRange(39711 - 39727)
3518	441.1960	S	0.0774	(0.81, 0.18)-(0.18, 0.25)	DataPointRange(39728 - 39734)	0.0774	0.63	8.10	15.87
3519	441.2843	F	0.1328	(0.19, 0.24)		DataPointRange(39735 - 39746)
3520	441.4060	S	0.0221	(0.20, 0.27)-(0.16, 0.26)	DataPointRange(39747 - 39748)	0.0221	0.04	2.00	3.58
3521	441.4392	F	0.1879	(0.15, 0.26)		DataPointRange(39749 - 39765)
3522	441.6159	S	0.0221	(0.15, 0.26)-(0.26, 0.25)	DataPointRange(39766 - 39767)	0.0221	0.11	4.95	8.09
3523	441.6492	F	0.2100	(0.29, 0.26)		DataPointRange(39768 - 39786)
3524	441.8479	S	0.0331	(0.29, 0.25)-(0.43, 0.25)	DataPointRange(39787 - 39789)	0.0331	0.13	4.07	7.11
3525	441.8922	F	0.3312	(0.45, 0.26)		DataPointRange(39790 - 39819)
3526	442.2123	S	0.0221	(0.44, 0.27)-(0.56, 0.27)	DataPointRange(39820 - 39821)	0.0221	0.11	5.02	6.83
3527	442.2455	F	0.1656	(0.59, 0.26)		DataPointRange(39822 - 39836)
3528	442.4001	S	0.0221	(0.58, 0.26)-(0.53, 0.27)	DataPointRange(39837 - 39838)	0.0221	0.05	2.47	5.87
3529	442.4333	F	0.2762	(0.50, 0.26)		DataPointRange(39839 - 39863)
3530	442.6985	S	0.0331	(0.51, 0.26)-(0.65, 0.26)	DataPointRange(39864 - 39866)	0.0331	0.14	4.23	6.04
3531	442.7428	F	0.3313	(0.64, 0.26)		DataPointRange(39867 - 39896)
3532	443.0631	S	0.0330	(0.64, 0.27)-(0.78, 0.26)	DataPointRange(39897 - 39899)	0.0330	0.13	4.02	6.88
3533	443.1071	F	0.2539	(0.79, 0.26)		DataPointRange(39900 - 39922)
3534	443.3502	S	0.0662	(0.79, 0.26)-(0.23, 0.34)	DataPointRange(39923 - 39928)	0.0662	0.56	8.52	11.10
3535	443.4273	F	0.4532	(0.22, 0.36)		DataPointRange(39929 - 39969)
3536	443.8693	S	0.0222	(0.22, 0.37)-(0.33, 0.36)	DataPointRange(39970 - 39971)	0.0222	0.11	5.08	6.96
3537	443.9025	F	0.3203	(0.36, 0.35)		DataPointRange(39972 - 40000)
3538	444.2119	S	0.0334	(0.36, 0.36)-(0.49, 0.37)	DataPointRange(40001 - 40003)	0.0334	0.13	3.85	7.20
3539	444.2559	F	0.1438	(0.51, 0.35)		DataPointRange(40004 - 40016)
3540	444.3886	S	0.1655	(0.52, 0.36)-(0.70, 0.40)	DataPointRange(40017 - 40031)	0.1655	0.18	1.08	80.05
3541	444.5651	F	0.0115	(0.71, 0.40)		DataPointRange(40032 - 40032)
3542	444.5651	S	0.0115	(0.71, 0.40)-(0.69, 0.35)	DataPointRange(40033 - 40033)	0.0115	0.04	3.50	3.50
3543	444.5872	F	0.3313	(0.66, 0.35)		DataPointRange(40034 - 40063)
3544	444.9075	S	0.0221	(0.65, 0.34)-(0.70, 0.36)	DataPointRange(40064 - 40065)	0.0221	0.05	2.41	3.53
3545	444.9407	F	0.3203	(0.71, 0.35)		DataPointRange(40066 - 40094)
3546	445.2499	S	0.0773	(0.71, 0.34)-(0.19, 0.45)	DataPointRange(40095 - 40101)	0.0773	0.53	6.87	12.00
3547	445.3384	F	0.2539	(0.18, 0.43)		DataPointRange(40102 - 40124)
3548	445.5813	S	0.0220	(0.18, 0.43)-(0.24, 0.45)	DataPointRange(40125 - 40126)	0.0220	0.07	3.05	4.61
3549	445.6144	F	0.3979	(0.25, 0.44)		DataPointRange(40127 - 40162)
3550	446.0012	S	0.0441	(0.25, 0.45)-(0.42, 0.44)	DataPointRange(40163 - 40166)	0.0441	0.17	3.96	6.94
3551	446.0566	F	0.2980	(0.44, 0.43)		DataPointRange(40167 - 40193)
3552	446.3435	S	0.0222	(0.43, 0.42)-(0.49, 0.43)	DataPointRange(40194 - 40195)	0.0222	0.06	2.79	5.37
3553	446.3768	F	0.2541	(0.52, 0.43)		DataPointRange(40196 - 40218)
3554	446.6196	S	0.0220	(0.52, 0.42)-(0.58, 0.45)	DataPointRange(40219 - 40220)	0.0220	0.07	3.07	5.27
3555	446.6528	F	0.1770	(0.60, 0.42)		DataPointRange(40221 - 40236)
3556	446.8186	S	0.0333	(0.59, 0.40)-(0.71, 0.45)	DataPointRange(40237 - 40239)	0.0333	0.13	3.78	6.14
3557	446.8632	F	0.1323	(0.71, 0.44)		DataPointRange(40240 - 40251)
3558	446.9845	S	0.0775	(0.71, 0.44)-(0.19, 0.52)	DataPointRange(40252 - 40258)	0.0775	0.53	6.82	10.28
3559	447.0729	F	0.3313	(0.16, 0.55)		DataPointRange(40259 - 40288)
3560	447.3930	S	0.0332	(0.16, 0.55)-(0.25, 0.54)	DataPointRange(40289 - 40291)	0.0332	0.09	2.85	4.53
3561	447.4373	F	0.2099	(0.26, 0.54)		DataPointRange(40292 - 40310)
3562	447.6360	S	0.0223	(0.27, 0.58)-(0.37, 0.56)	DataPointRange(40311 - 40312)	0.0223	0.10	4.44	8.36
3563	447.6693	F	0.2873	(0.40, 0.54)		DataPointRange(40313 - 40338)
3564	447.9453	S	0.0333	(0.39, 0.55)-(0.57, 0.52)	DataPointRange(40339 - 40341)	0.0333	0.18	5.43	8.38
3565	447.9899	F	0.3200	(0.60, 0.52)		DataPointRange(40342 - 40370)
3566	448.2990	S	0.0771	(0.60, 0.52)-(1.87, -0.24)	DataPointRange(40371 - 40377)	0.0771	1.39	18.07	186.22
3567	448.3876	F	0.0108	(1.87, -0.22)		DataPointRange(40378 - 40378)
3568	448.3876	S	0.0108	(1.87, -0.22)-(1.87, -0.23)	DataPointRange(40379 - 40379)	0.0108	0.01	1.13	1.13
3569	448.4095	F	0.0110	(1.87, -0.29)		DataPointRange(40380 - 40380)
3570	448.4095	S	0.0110	(1.87, -0.29)-(1.87, -0.22)	DataPointRange(40381 - 40381)	0.0110	0.05	4.83	4.83
3571	448.4314	F	0.0111	(1.87, -0.22)		DataPointRange(40382 - 40382)
3572	448.4314	S	0.0224	(1.87, -0.22)-(1.87, -0.29)	DataPointRange(40383 - 40384)	0.0224	0.05	2.42	4.81
3573	448.4646	F	0.0111	(1.86, -0.32)		DataPointRange(40385 - 40385)
3574	448.4646	S	0.1215	(1.86, -0.32)-(0.41, 0.50)	DataPointRange(40386 - 40396)	0.1215	1.58	13.03	134.49
3575	448.5971	F	0.0227	(0.41, 0.50)		DataPointRange(40397 - 40398)
3576	448.6082	S	0.0116	(0.42, 0.52)-(0.40, 0.45)	DataPointRange(40399 - 40399)	0.0116	0.05	4.37	4.37
3577	448.6303	F	0.0224	(0.39, 0.45)		DataPointRange(40400 - 40401)
3578	448.6413	S	0.0114	(0.40, 0.45)-(0.40, 0.49)	DataPointRange(40402 - 40402)	0.0114	0.03	2.74	2.74
3579	448.6634	F	0.1106	(0.42, 0.52)		DataPointRange(40403 - 40412)
3580	448.7630	S	0.0437	(0.43, 0.51)-(0.64, 0.50)	DataPointRange(40413 - 40416)	0.0437	0.21	4.76	8.11
3581	448.8181	F	0.2872	(0.65, 0.51)		DataPointRange(40417 - 40442)
3582	449.0942	S	0.0441	(0.66, 0.50)-(0.40, 0.33)	DataPointRange(40443 - 40446)	0.0441	0.29	6.57	10.78
3583	449.1498	F	0.1653	(0.39, 0.32)		DataPointRange(40447 - 40461)
3584	449.3041	S	0.0332	(0.39, 0.33)-(0.29, 0.23)	DataPointRange(40462 - 40464)	0.0332	0.13	3.79	6.18
3585	449.3483	F	0.1545	(0.29, 0.22)		DataPointRange(40465 - 40478)
3586	449.4917	S	0.0111	(0.30, 0.22)-(0.27, 0.20)	DataPointRange(40479 - 40479)	0.0111	0.03	2.53	2.53
3587	449.5140	F	0.9389	(0.25, 0.17)		DataPointRange(40480 - 40564)
3588	449.9004	End					DataPoint(40515)
3589	450.2981	t1n10.bmp				DataPoint(40551)
3590	450.3313	Start					DataPoint(40554)
3591	450.4419	S	0.0110	(0.25, 0.16)-(0.24, 0.16)	DataPointRange(40565 - 40565)	0.0110	0.02	1.57	1.57
3592	450.4641	F	0.1768	(0.21, 0.16)		DataPointRange(40566 - 40581)
3593	450.6297	S	0.0331	(0.21, 0.18)-(0.30, 0.17)	DataPointRange(40582 - 40584)	0.0331	0.09	2.73	5.17
3594	450.6739	F	0.2981	(0.32, 0.17)		DataPointRange(40585 - 40611)
3595	450.9609	S	0.0330	(0.33, 0.17)-(0.45, 0.17)	DataPointRange(40612 - 40614)	0.0330	0.13	3.90	6.66
3596	451.0053	F	0.2986	(0.47, 0.18)		DataPointRange(40615 - 40641)
3597	451.2924	S	0.0220	(0.48, 0.17)-(0.56, 0.18)	DataPointRange(40642 - 40643)	0.0220	0.09	3.92	5.22
3598	451.3255	F	0.2211	(0.57, 0.17)		DataPointRange(40644 - 40663)
3599	451.5358	S	0.0217	(0.57, 0.18)-(0.67, 0.17)	DataPointRange(40664 - 40665)	0.0217	0.10	4.65	6.40
3600	451.5686	F	0.2429	(0.68, 0.17)		DataPointRange(40666 - 40687)
3601	451.8004	S	0.0220	(0.68, 0.17)-(0.76, 0.19)	DataPointRange(40688 - 40689)	0.0220	0.08	3.44	5.77
3602	451.8336	F	0.2651	(0.77, 0.17)		DataPointRange(40690 - 40713)
3603	452.0876	S	0.0111	(0.77, 0.18)-(0.80, 0.18)	DataPointRange(40714 - 40714)	0.0111	0.03	3.04	3.04
3604	452.1096	F	0.1104	(0.82, 0.17)		DataPointRange(40715 - 40724)
3605	452.2089	S	0.0664	(0.82, 0.16)-(0.26, 0.22)	DataPointRange(40725 - 40730)	0.0664	0.56	8.42	12.10
3606	452.2864	F	0.1659	(0.24, 0.24)		DataPointRange(40731 - 40745)
3607	452.4412	S	0.0111	(0.25, 0.24)-(0.22, 0.26)	DataPointRange(40746 - 40746)	0.0111	0.03	3.08	3.08
3608	452.4631	F	0.2432	(0.18, 0.26)		DataPointRange(40747 - 40768)
3609	452.6957	S	0.0333	(0.18, 0.26)-(0.29, 0.26)	DataPointRange(40769 - 40771)	0.0333	0.11	3.35	4.57
3610	452.7394	F	0.2542	(0.29, 0.27)		DataPointRange(40772 - 40794)
3611	452.9826	S	0.0221	(0.28, 0.26)-(0.38, 0.27)	DataPointRange(40795 - 40796)	0.0221	0.10	4.62	6.45
3612	453.0155	F	0.2208	(0.38, 0.26)		DataPointRange(40797 - 40816)
3613	453.2254	S	0.0219	(0.38, 0.26)-(0.46, 0.27)	DataPointRange(40817 - 40818)	0.0219	0.08	3.76	4.92
3614	453.2584	F	0.1549	(0.46, 0.26)		DataPointRange(40819 - 40832)
3615	453.4022	S	0.0222	(0.47, 0.26)-(0.52, 0.25)	DataPointRange(40833 - 40834)	0.0222	0.06	2.59	5.34
3616	453.4353	F	0.1768	(0.58, 0.26)		DataPointRange(40835 - 40850)
3617	453.6011	S	0.0227	(0.58, 0.23)-(0.66, 0.28)	DataPointRange(40851 - 40852)	0.0227	0.09	3.78	5.48
3618	453.6344	F	0.2097	(0.68, 0.26)		DataPointRange(40853 - 40871)
3619	453.8331	S	0.0661	(0.68, 0.27)-(-0.98, -0.09)	DataPointRange(40872 - 40877)	0.0661	1.68	25.46	234.08
3620	453.9102	F	0.0114	(-0.96, -0.04)		DataPointRange(40878 - 40878)
3621	453.9102	S	0.1442	(-0.96, -0.04)-(0.83, 0.22)	DataPointRange(40879 - 40891)	0.1442	1.80	12.50	263.63
3622	454.0649	F	0.0111	(0.84, 0.20)		DataPointRange(40892 - 40892)
3623	454.0649	S	0.0225	(0.84, 0.20)-(0.82, 0.26)	DataPointRange(40893 - 40894)	0.0225	0.05	2.07	4.77
3624	454.0982	F	0.1875	(0.82, 0.26)		DataPointRange(40895 - 40911)
3625	454.2748	S	0.0661	(0.82, 0.25)-(0.23, 0.33)	DataPointRange(40912 - 40917)	0.0661	0.59	8.86	11.41
3626	454.3521	F	0.1546	(0.24, 0.33)		DataPointRange(40918 - 40931)
3627	454.4956	S	0.0226	(0.24, 0.34)-(0.17, 0.35)	DataPointRange(40932 - 40933)	0.0226	0.07	3.00	6.10
3628	454.5289	F	0.3205	(0.18, 0.37)		DataPointRange(40934 - 40962)
3629	454.8382	S	0.0222	(0.18, 0.37)-(0.24, 0.33)	DataPointRange(40963 - 40964)	0.0222	0.06	2.79	5.34
3630	454.8713	F	0.1545	(0.27, 0.34)		DataPointRange(40965 - 40978)
3631	455.0150	S	0.0328	(0.27, 0.34)-(0.20, 0.27)	DataPointRange(40979 - 40981)	0.0328	0.09	2.70	4.31
3632	455.0590	F	1.0938	(0.19, 0.22)		DataPointRange(40982 - 41080)
3633	455.2359	End					DataPoint(40998)
3634	455.6003	aa1n14s.bmp				DataPoint(41031)
3635	455.6665	Start					DataPoint(41037)
3636	456.1417	S	0.0220	(0.19, 0.23)-(0.13, 0.16)	DataPointRange(41081 - 41082)	0.0220	0.08	3.70	4.75
3637	456.1751	F	0.2648	(0.14, 0.15)		DataPointRange(41083 - 41106)
3638	456.4288	S	0.0331	(0.14, 0.17)-(0.27, 0.18)	DataPointRange(41107 - 41109)	0.0331	0.13	3.87	6.30
3639	456.4730	F	0.2542	(0.29, 0.18)		DataPointRange(41110 - 41132)
3640	456.7161	S	0.0111	(0.30, 0.17)-(0.32, 0.17)	DataPointRange(41133 - 41133)	0.0111	0.02	1.42	1.42
3641	456.7384	F	0.2098	(0.34, 0.18)		DataPointRange(41134 - 41152)
3642	456.9376	S	0.0217	(0.34, 0.19)-(0.45, 0.17)	DataPointRange(41153 - 41154)	0.0217	0.11	5.24	6.04
3643	456.9703	F	0.2537	(0.48, 0.18)		DataPointRange(41155 - 41177)
3644	457.2131	S	0.0221	(0.49, 0.19)-(0.55, 0.17)	DataPointRange(41178 - 41179)	0.0221	0.06	2.87	4.42
3645	457.2462	F	0.2215	(0.56, 0.18)		DataPointRange(41180 - 41199)
3646	457.4562	S	0.0115	(0.56, 0.19)-(0.57, 0.16)	DataPointRange(41200 - 41200)	0.0115	0.02	1.77	1.77
3647	457.4783	F	0.2542	(0.61, 0.18)		DataPointRange(41201 - 41223)
3648	457.7212	S	0.0331	(0.61, 0.19)-(0.72, 0.18)	DataPointRange(41224 - 41226)	0.0331	0.11	3.23	4.70
3649	457.7659	F	0.2535	(0.76, 0.18)		DataPointRange(41227 - 41249)
3650	458.0084	S	0.0110	(0.76, 0.16)-(0.80, 0.17)	DataPointRange(41250 - 41250)	0.0110	0.04	3.60	3.60
3651	458.0305	F	0.1655	(0.83, 0.17)		DataPointRange(41251 - 41265)
3652	458.1851	S	0.0775	(0.83, 0.17)-(0.22, 0.22)	DataPointRange(41266 - 41272)	0.0775	0.61	7.93	11.24
3653	458.2736	F	0.1437	(0.20, 0.23)		DataPointRange(41273 - 41285)
3654	458.4061	S	0.0221	(0.20, 0.24)-(0.15, 0.24)	DataPointRange(41286 - 41287)	0.0221	0.05	2.05	4.53
3655	458.4391	F	0.1552	(0.16, 0.27)		DataPointRange(41288 - 41301)
3656	458.5829	S	0.0329	(0.16, 0.27)-(0.29, 0.24)	DataPointRange(41302 - 41304)	0.0329	0.14	4.13	6.18
3657	458.6272	F	0.2429	(0.31, 0.26)		DataPointRange(41305 - 41326)
3658	458.8592	S	0.0219	(0.32, 0.26)-(0.38, 0.26)	DataPointRange(41327 - 41328)	0.0219	0.06	2.91	6.14
3659	458.8924	F	0.2979	(0.39, 0.26)		DataPointRange(41329 - 41355)
3660	459.1793	S	0.0330	(0.40, 0.26)-(0.55, 0.27)	DataPointRange(41356 - 41358)	0.0330	0.14	4.33	6.38
3661	459.2240	F	0.2867	(0.56, 0.26)		DataPointRange(41359 - 41384)
3662	459.4998	S	0.0109	(0.56, 0.24)-(0.58, 0.26)	DataPointRange(41385 - 41385)	0.0109	0.03	2.67	2.67
3663	459.5222	F	0.3087	(0.63, 0.26)		DataPointRange(41386 - 41413)
3664	459.8201	S	0.0329	(0.63, 0.28)-(0.73, 0.26)	DataPointRange(41414 - 41416)	0.0329	0.09	2.86	4.45
3665	459.8640	F	0.2098	(0.75, 0.25)		DataPointRange(41417 - 41435)
3666	460.0630	S	0.2214	(0.74, 0.26)-(0.21, 0.29)	DataPointRange(41436 - 41455)	0.2214	0.54	2.42	32.01
3667	460.2951	F	0.3093	(0.18, 0.36)		DataPointRange(41456 - 41483)
3668	460.5934	S	0.0331	(0.16, 0.36)-(0.27, 0.35)	DataPointRange(41484 - 41486)	0.0331	0.11	3.38	5.96
3669	460.6374	F	0.3202	(0.28, 0.36)		DataPointRange(41487 - 41515)
3670	460.9466	S	0.0332	(0.29, 0.37)-(0.46, 0.34)	DataPointRange(41516 - 41518)	0.0332	0.17	5.12	7.40
3671	460.9908	F	0.2209	(0.46, 0.34)		DataPointRange(41519 - 41538)
3672	461.2007	S	0.0222	(0.47, 0.34)-(0.56, 0.32)	DataPointRange(41539 - 41540)	0.0222	0.09	4.03	4.43
3673	461.2339	F	0.2542	(0.57, 0.34)		DataPointRange(41541 - 41563)
3674	461.4770	S	0.1878	(0.58, 0.38)-(0.65, 0.37)	DataPointRange(41564 - 41580)	0.1878	0.07	0.36	289.36
3675	461.6764	F	0.1430	(0.65, 0.33)		DataPointRange(41581 - 41593)
3676	461.8087	S	0.0549	(0.66, 0.36)-(0.20, 0.42)	DataPointRange(41594 - 41598)	0.0549	0.46	8.31	13.23
3677	461.8749	F	0.1542	(0.20, 0.46)		DataPointRange(41599 - 41612)
3678	462.0180	S	0.0221	(0.20, 0.49)-(0.14, 0.48)	DataPointRange(41613 - 41614)	0.0221	0.06	2.68	4.27
3679	462.0513	F	0.1548	(0.14, 0.45)		DataPointRange(41615 - 41628)
3680	462.1949	S	0.0553	(0.14, 0.45)-(0.37, 0.44)	DataPointRange(41629 - 41633)	0.0553	0.23	4.21	9.27
3681	462.2614	F	0.3096	(0.40, 0.43)		DataPointRange(41634 - 41661)
3682	462.5597	S	0.0446	(0.40, 0.42)-(0.65, 0.41)	DataPointRange(41662 - 41665)	0.0446	0.25	5.52	10.90
3683	462.6148	F	0.2982	(0.66, 0.42)		DataPointRange(41666 - 41692)
3684	462.9020	S	0.0331	(0.66, 0.42)-(0.79, 0.44)	DataPointRange(41693 - 41695)	0.0331	0.13	3.85	6.43
3685	462.9463	F	0.5632	(0.82, 0.44)		DataPointRange(41696 - 41746)
3686	463.4432	End					DataPoint(41741)
3687	463.4987	S	0.0550	(0.81, 0.46)-(-1.10, 0.02)	DataPointRange(41747 - 41751)	0.0550	1.94	35.26	227.74
3688	463.5651	F	0.0107	(-1.14, 0.02)		DataPointRange(41752 - 41752)
3689	463.5651	S	0.0548	(-1.14, 0.02)-(1.86, -0.31)	DataPointRange(41753 - 41757)	0.0548	3.01	54.97	279.55
3690	463.6315	F	0.0106	(-1.18, -0.08)		DataPointRange(41758 - 41758)
3691	463.6315	S	0.0106	(-1.18, -0.08)-(1.86, -0.32)	DataPointRange(41759 - 41759)	0.0106	3.04	287.05	287.05
3692	463.6532	F	0.0113	(-1.24, -0.22)		DataPointRange(41760 - 41760)
3693	463.6532	S	0.0113	(-1.24, -0.22)-(1.86, -0.32)	DataPointRange(41761 - 41761)	0.0113	3.10	274.29	274.29
3694	463.6753	F	0.0108	(1.86, -0.31)		DataPointRange(41762 - 41762)
3695	463.6753	S	0.1102	(1.86, -0.31)-(0.35, 0.30)	DataPointRange(41763 - 41772)	0.1102	1.58	14.31	192.11
3696	463.7855	aa1n5.bmp				DataPoint(41772)
3697	463.7978	F	0.1426	(0.40, 0.34)		DataPointRange(41773 - 41784)
3698	463.8517	Start					DataPoint(41777)
3699	463.9293	S	0.0443	(0.40, 0.36)-(0.17, 0.25)	DataPointRange(41785 - 41788)	0.0443	0.24	5.38	10.16
3700	463.9844	F	0.1548	(0.20, 0.24)		DataPointRange(41789 - 41802)
3701	464.1278	S	0.0114	(0.20, 0.23)-(0.20, 0.19)	DataPointRange(41803 - 41803)	0.0114	0.03	2.38	2.38
3702	464.1500	F	0.1769	(0.21, 0.17)		DataPointRange(41804 - 41819)
3703	464.3159	S	0.0225	(0.21, 0.18)-(0.30, 0.18)	DataPointRange(41820 - 41821)	0.0225	0.09	4.16	5.54
3704	464.3490	F	0.3535	(0.30, 0.18)		DataPointRange(41822 - 41853)
3705	464.6915	S	0.1657	(0.31, 0.19)-(0.41, 0.22)	DataPointRange(41854 - 41868)	0.1657	0.11	0.64	148.22
3706	464.8685	F	0.1544	(0.41, 0.21)		DataPointRange(41869 - 41882)
3707	465.0118	S	0.0332	(0.40, 0.21)-(0.32, 0.19)	DataPointRange(41883 - 41885)	0.0332	0.08	2.29	4.92
3708	465.0558	F	0.3428	(0.31, 0.19)		DataPointRange(41886 - 41916)
3709	465.3873	S	0.0113	(0.31, 0.17)-(0.26, 0.19)	DataPointRange(41917 - 41917)	0.0113	0.04	3.76	3.76
3710	465.4094	F	0.2321	(0.26, 0.19)		DataPointRange(41918 - 41938)
3711	465.6307	S	0.0218	(0.26, 0.20)-(0.35, 0.17)	DataPointRange(41939 - 41940)	0.0218	0.10	4.43	6.27
3712	465.6639	F	0.1657	(0.40, 0.20)		DataPointRange(41941 - 41955)
3713	465.8182	S	0.0114	(0.40, 0.19)-(0.40, 0.20)	DataPointRange(41956 - 41956)	0.0114	0.01	0.71	0.71
3714	465.8402	F	0.0111	(0.35, 0.25)		DataPointRange(41957 - 41957)
3715	465.8402	S	0.0111	(0.35, 0.25)-(0.40, 0.19)	DataPointRange(41958 - 41958)	0.0111	0.07	6.66	6.66
3716	465.8627	F	0.0992	(0.41, 0.19)		DataPointRange(41959 - 41967)
3717	465.9507	S	0.0333	(0.42, 0.19)-(0.32, 0.17)	DataPointRange(41968 - 41970)	0.0333	0.10	3.11	4.72
3718	465.9954	F	0.0878	(0.32, 0.17)		DataPointRange(41971 - 41978)
3719	466.0722	S	0.0110	(0.32, 0.17)-(0.32, 0.17)	DataPointRange(41979 - 41979)	0.0110	0.00	0.30	0.30
3720	466.0942	F	0.0112	(0.26, 0.23)		DataPointRange(41980 - 41980)
3721	466.0942	S	0.0112	(0.26, 0.23)-(0.33, 0.16)	DataPointRange(41981 - 41981)	0.0112	0.08	7.29	7.29
3722	466.1163	F	0.0777	(0.32, 0.17)		DataPointRange(41982 - 41988)
3723	466.1826	S	0.0331	(0.33, 0.16)-(0.46, 0.17)	DataPointRange(41989 - 41991)	0.0331	0.13	4.03	7.12
3724	466.2272	F	0.2868	(0.47, 0.17)		DataPointRange(41992 - 42017)
3725	466.5029	S	0.0111	(0.47, 0.17)-(0.49, 0.18)	DataPointRange(42018 - 42018)	0.0111	0.02	1.90	1.90
3726	466.5251	F	0.0110	(0.43, 0.25)		DataPointRange(42019 - 42019)
3727	466.5251	S	0.0110	(0.43, 0.25)-(0.48, 0.18)	DataPointRange(42020 - 42020)	0.0110	0.07	6.67	6.67
3728	466.5471	F	0.0331	(0.48, 0.19)		DataPointRange(42021 - 42023)
3729	466.5691	S	0.0111	(0.47, 0.19)-(0.47, 0.16)	DataPointRange(42024 - 42024)	0.0111	0.02	1.64	1.64
3730	466.5913	F	0.0111	(0.43, 0.21)		DataPointRange(42025 - 42025)
3731	466.5913	S	0.0335	(0.43, 0.21)-(0.61, 0.16)	DataPointRange(42026 - 42028)	0.0335	0.19	5.58	6.86
3732	466.6354	F	0.2548	(0.64, 0.17)		DataPointRange(42029 - 42051)
3733	466.8788	S	0.0331	(0.65, 0.18)-(0.77, 0.18)	DataPointRange(42052 - 42054)	0.0331	0.12	3.74	5.52
3734	466.9229	F	0.1436	(0.76, 0.16)		DataPointRange(42055 - 42067)
3735	467.0560	S	0.0662	(0.76, 0.15)-(0.27, 0.24)	DataPointRange(42068 - 42073)	0.0662	0.49	7.43	12.01
3736	467.1327	F	0.1546	(0.23, 0.27)		DataPointRange(42074 - 42085)
3737	467.2765	S	0.0220	(0.24, 0.29)-(0.15, 0.27)	DataPointRange(42086 - 42087)	0.0220	0.09	3.97	6.00
3738	467.3097	F	0.1986	(0.15, 0.28)		DataPointRange(42088 - 42105)
3739	467.4969	S	0.0221	(0.14, 0.29)-(0.26, 0.26)	DataPointRange(42106 - 42107)	0.0221	0.12	5.57	8.08
3740	467.5301	F	0.2100	(0.30, 0.26)		DataPointRange(42108 - 42126)
3741	467.7290	S	0.0446	(0.29, 0.23)-(0.45, 0.26)	DataPointRange(42127 - 42130)	0.0446	0.16	3.53	5.84
3742	467.7843	F	0.4088	(0.45, 0.26)		DataPointRange(42131 - 42167)
3743	468.1821	S	0.0330	(0.44, 0.26)-(0.55, 0.25)	DataPointRange(42168 - 42170)	0.0330	0.10	3.17	5.22
3744	468.2264	F	0.1434	(0.57, 0.27)		DataPointRange(42171 - 42183)
3745	468.3587	S	0.0331	(0.56, 0.28)-(0.66, 0.27)	DataPointRange(42184 - 42186)	0.0331	0.10	3.17	5.43
3746	468.4033	F	0.1872	(0.67, 0.26)		DataPointRange(42187 - 42203)
3747	468.5794	S	0.0111	(0.68, 0.26)-(0.68, 0.27)	DataPointRange(42204 - 42204)	0.0111	0.01	0.63	0.63
3748	468.6017	F	0.0109	(0.62, 0.30)		DataPointRange(42205 - 42205)
3749	468.6017	S	0.0109	(0.62, 0.30)-(0.68, 0.27)	DataPointRange(42206 - 42206)	0.0109	0.07	6.37	6.37
3750	468.6237	F	0.1439	(0.68, 0.26)		DataPointRange(42207 - 42219)
3751	468.7564	S	0.0332	(0.67, 0.25)-(0.80, 0.26)	DataPointRange(42220 - 42222)	0.0332	0.13	3.92	5.12
3752	468.8006	F	0.3535	(0.83, 0.26)		DataPointRange(42223 - 42254)
3753	469.1432	S	0.0773	(0.83, 0.27)-(0.40, 0.32)	DataPointRange(42255 - 42258)	0.0773	0.43	5.56	34.66
3754	469.2316	F	0.1213	(0.41, 0.32)		DataPointRange(42259 - 42269)
3755	469.3419	S	0.0442	(0.41, 0.32)-(0.17, 0.35)	DataPointRange(42270 - 42273)	0.0442	0.24	5.47	8.95
3756	469.3971	F	0.3092	(0.18, 0.35)		DataPointRange(42274 - 42301)
3757	469.6956	S	0.0331	(0.17, 0.36)-(0.30, 0.34)	DataPointRange(42302 - 42304)	0.0331	0.13	3.98	6.09
3758	469.7396	F	0.2540	(0.32, 0.34)		DataPointRange(42305 - 42327)
3759	469.9825	S	0.0222	(0.33, 0.34)-(0.40, 0.34)	DataPointRange(42328 - 42329)	0.0222	0.06	2.84	5.15
3760	470.0158	F	0.1656	(0.43, 0.34)		DataPointRange(42330 - 42344)
3761	470.1702	S	0.0222	(0.44, 0.34)-(0.52, 0.34)	DataPointRange(42345 - 42346)	0.0222	0.08	3.63	5.39
3762	470.2034	F	0.1765	(0.54, 0.34)		DataPointRange(42347 - 42362)
3763	470.3690	S	0.0109	(0.55, 0.36)-(0.54, 0.37)	DataPointRange(42363 - 42363)	0.0109	0.01	0.80	0.80
3764	470.3912	F	0.0110	(0.48, 0.20)		DataPointRange(42364 - 42364)
3765	470.3912	S	0.0773	(0.48, 0.20)-(1.87, -0.23)	DataPointRange(42365 - 42371)	0.0773	1.42	18.43	288.39
3766	470.4797	F	0.0220	(1.87, -0.23)		DataPointRange(42372 - 42373)
3767	470.4911	S	0.1101	(1.86, -0.23)-(0.65, 0.33)	DataPointRange(42374 - 42383)	0.1101	1.28	11.67	214.69
3768	470.6123	F	0.0114	(0.61, 0.29)		DataPointRange(42384 - 42384)
3769	470.6123	S	0.0331	(0.61, 0.29)-(0.65, 0.36)	DataPointRange(42385 - 42387)	0.0331	0.07	2.09	5.50
3770	470.6568	F	0.2426	(0.65, 0.35)		DataPointRange(42388 - 42409)
3771	470.8883	S	0.0333	(0.65, 0.33)-(0.78, 0.37)	DataPointRange(42410 - 42412)	0.0333	0.14	4.14	5.48
3772	470.9326	F	0.4086	(0.79, 0.35)		DataPointRange(42413 - 42448)
3773	471.3301	S	0.1435	(0.80, 0.38)-(1.87, -0.23)	DataPointRange(42449 - 42461)	0.1435	1.16	8.07	153.04
3774	471.4846	F	0.0115	(1.87, -0.28)		DataPointRange(42462 - 42462)
3775	471.4846	S	0.1216	(1.87, -0.28)-(0.53, 0.38)	DataPointRange(42463 - 42473)	0.1216	1.42	11.71	184.93
3776	471.6173	F	0.0444	(0.54, 0.34)		DataPointRange(42474 - 42477)
3777	471.6504	S	0.0113	(0.53, 0.34)-(0.55, 0.35)	DataPointRange(42478 - 42478)	0.0113	0.02	1.79	1.79
3778	471.6724	F	0.0663	(0.54, 0.40)		DataPointRange(42479 - 42484)
3779	471.7280	S	0.0769	(0.55, 0.40)-(0.53, 1.19)	DataPointRange(42485 - 42491)	0.0769	0.59	7.68	13.84
3780	471.8159	F	0.0664	(0.52, 1.18)		DataPointRange(42492 - 42497)
3781	471.8712	S	0.0111	(0.53, 1.20)-(0.53, 1.17)	DataPointRange(42498 - 42498)	0.0111	0.02	2.03	2.03
3782	471.8935	F	0.0552	(0.52, 1.16)		DataPointRange(42499 - 42503)
3783	471.9376	S	0.0334	(0.52, 1.17)-(0.51, 1.37)	DataPointRange(42504 - 42506)	0.0334	0.15	4.50	8.93
3784	471.9820	F	0.4639	(0.51, 1.31)		DataPointRange(42507 - 42548)
3785	472.4347	S	0.0995	(0.53, 1.28)-(1.86, -0.36)	DataPointRange(42549 - 42557)	0.0995	1.81	18.17	197.03
3786	472.5454	F	0.0221	(1.86, -0.35)		DataPointRange(42558 - 42559)
3787	472.5569	S	0.1325	(1.86, -0.36)-(0.21, 0.31)	DataPointRange(42560 - 42571)	0.1325	1.72	13.02	212.02
3788	472.7000	F	0.0224	(0.24, 0.30)		DataPointRange(42572 - 42573)
3789	472.7109	S	0.0221	(0.23, 0.28)-(0.24, 0.34)	DataPointRange(42574 - 42575)	0.0221	0.05	2.06	4.95
3790	472.7442	F	0.1435	(0.25, 0.36)		DataPointRange(42576 - 42588)
3791	472.8104	End					DataPoint(42582)
3792	472.8764	S	0.0331	(0.24, 0.35)-(0.15, 0.23)	DataPointRange(42589 - 42591)	0.0331	0.12	3.74	5.61
3793	472.9207	F	0.2432	(0.15, 0.21)		DataPointRange(42592 - 42613)
3794	473.1531	S	0.0739	(0.15, 0.22)-(0.21, 0.18)	DataPointRange(42614 - 42615)	0.0739	0.07	0.91	1.01
3795	473.2270	aa1n8.bmp				DataPoint(42615)
3796	473.2425	F	0.1646	(0.22, 0.20)		DataPointRange(42616 - 42630)
3797	473.2853	Start					DataPoint(42620)
3798	473.3958	S	0.0113	(0.22, 0.21)-(0.21, 0.19)	DataPointRange(42631 - 42631)	0.0113	0.02	1.39	1.39
3799	473.4178	F	0.2764	(0.19, 0.17)		DataPointRange(42632 - 42656)
3800	473.6830	S	0.0112	(0.19, 0.17)-(0.15, 0.16)	DataPointRange(42657 - 42657)	0.0112	0.03	2.98	2.98
3801	473.7052	F	0.1878	(0.13, 0.15)		DataPointRange(42658 - 42674)
3802	473.8819	S	0.0441	(0.12, 0.16)-(0.32, 0.17)	DataPointRange(42675 - 42678)	0.0441	0.20	4.49	5.71
3803	473.9375	F	0.1319	(0.33, 0.17)		DataPointRange(42679 - 42690)
3804	474.0585	S	0.0222	(0.33, 0.16)-(0.42, 0.18)	DataPointRange(42691 - 42692)	0.0222	0.09	4.22	7.59
3805	474.0917	F	0.2873	(0.44, 0.17)		DataPointRange(42693 - 42718)
3806	474.3680	S	0.0110	(0.44, 0.18)-(0.46, 0.16)	DataPointRange(42719 - 42719)	0.0110	0.03	2.63	2.63
3807	474.3901	F	0.1656	(0.50, 0.17)		DataPointRange(42720 - 42734)
3808	474.5448	S	0.0331	(0.50, 0.18)-(0.63, 0.18)	DataPointRange(42735 - 42737)	0.0331	0.12	3.76	7.26
3809	474.5888	F	0.2098	(0.65, 0.17)		DataPointRange(42738 - 42756)
3810	474.7876	S	0.0332	(0.64, 0.16)-(0.76, 0.17)	DataPointRange(42757 - 42759)	0.0332	0.12	3.58	5.93
3811	474.8324	F	0.1543	(0.79, 0.17)		DataPointRange(42760 - 42773)
3812	474.9755	S	0.0770	(0.78, 0.16)-(0.18, 0.23)	DataPointRange(42774 - 42780)	0.0770	0.60	7.82	13.86
3813	475.0639	F	0.2868	(0.20, 0.24)		DataPointRange(42781 - 42806)
3814	475.3399	S	0.0330	(0.21, 0.27)-(0.33, 0.24)	DataPointRange(42807 - 42809)	0.0330	0.13	3.81	6.88
3815	475.3838	F	0.4200	(0.37, 0.24)		DataPointRange(42810 - 42847)
3816	475.7928	S	0.0110	(0.38, 0.25)-(0.34, 0.25)	DataPointRange(42848 - 42848)	0.0110	0.04	3.37	3.37
3817	475.8149	F	0.1768	(0.34, 0.25)		DataPointRange(42849 - 42864)
3818	475.9806	S	0.0331	(0.34, 0.24)-(0.50, 0.24)	DataPointRange(42865 - 42867)	0.0331	0.15	4.64	7.83
3819	476.0251	F	0.2538	(0.52, 0.24)		DataPointRange(42868 - 42890)
3820	476.2677	S	0.0224	(0.53, 0.25)-(0.59, 0.23)	DataPointRange(42891 - 42892)	0.0224	0.06	2.84	6.19
3821	476.3009	F	0.2542	(0.60, 0.25)		DataPointRange(42893 - 42915)
3822	476.5442	S	0.0551	(0.60, 0.25)-(0.24, 0.32)	DataPointRange(42916 - 42920)	0.0551	0.36	6.58	11.85
3823	476.6103	F	0.4639	(0.21, 0.34)		DataPointRange(42921 - 42962)
3824	477.0633	S	0.0109	(0.21, 0.36)-(0.17, 0.35)	DataPointRange(42963 - 42963)	0.0109	0.04	3.76	3.76
3825	477.0851	F	0.1987	(0.17, 0.35)		DataPointRange(42964 - 42981)
3826	477.2728	S	0.0441	(0.17, 0.34)-(0.37, 0.35)	DataPointRange(42982 - 42985)	0.0441	0.20	4.53	8.15
3827	477.3280	F	0.0997	(0.42, 0.34)		DataPointRange(42986 - 42994)
3828	477.4166	S	0.1218	(0.42, 0.35)-(1.87, -0.23)	DataPointRange(42995 - 43005)	0.1218	1.50	12.35	231.68
3829	477.5496	F	0.0104	(-1.00, -0.36)		DataPointRange(43006 - 43006)
3830	477.5496	S	0.1100	(-1.00, -0.36)-(0.69, 0.27)	DataPointRange(43007 - 43016)	0.1100	1.75	15.95	275.95
3831	477.6709	F	0.1541	(0.69, 0.28)		DataPointRange(43017 - 43030)
3832	477.8140	S	0.0442	(0.68, 0.28)-(0.39, 0.34)	DataPointRange(43031 - 43034)	0.0442	0.30	6.68	10.31
3833	477.8693	F	0.2319	(0.35, 0.34)		DataPointRange(43035 - 43055)
3834	478.0902	S	0.0110	(0.35, 0.32)-(0.34, 0.36)	DataPointRange(43056 - 43056)	0.0110	0.03	2.95	2.95
3835	478.1125	F	0.0219	(0.35, 0.37)		DataPointRange(43057 - 43058)
3836	478.1233	S	0.0332	(0.35, 0.35)-(0.45, 0.33)	DataPointRange(43059 - 43061)	0.0332	0.10	3.04	4.37
3837	478.1675	F	0.4309	(0.45, 0.34)		DataPointRange(43062 - 43100)
3838	478.5874	S	0.0220	(0.47, 0.34)-(0.41, 0.34)	DataPointRange(43101 - 43102)	0.0220	0.05	2.35	3.35
3839	478.6206	F	0.1215	(0.37, 0.35)		DataPointRange(43103 - 43113)
3840	478.7313	S	0.0440	(0.37, 0.34)-(0.16, 0.42)	DataPointRange(43114 - 43117)	0.0440	0.22	5.04	7.95
3841	478.7863	F	0.2872	(0.17, 0.43)		DataPointRange(43118 - 43143)
3842	479.0626	S	0.0219	(0.16, 0.45)-(0.22, 0.44)	DataPointRange(43144 - 43145)	0.0219	0.07	3.05	5.96
3843	479.0957	F	0.1987	(0.26, 0.44)		DataPointRange(43146 - 43163)
3844	479.2834	S	0.0226	(0.26, 0.45)-(0.18, 0.47)	DataPointRange(43164 - 43165)	0.0226	0.09	3.84	5.71
3845	479.3165	F	0.3094	(0.15, 0.46)		DataPointRange(43166 - 43193)
3846	479.6147	S	0.0331	(0.14, 0.48)-(0.30, 0.46)	DataPointRange(43194 - 43196)	0.0331	0.16	4.76	8.21
3847	479.6590	F	0.3097	(0.32, 0.44)		DataPointRange(43197 - 43224)
3848	479.9574	S	0.0332	(0.32, 0.44)-(0.45, 0.42)	DataPointRange(43225 - 43227)	0.0332	0.13	3.81	5.80
3849	480.0019	F	0.4303	(0.46, 0.43)		DataPointRange(43228 - 43266)
3850	480.4210	S	0.0331	(0.46, 0.45)-(0.61, 0.44)	DataPointRange(43267 - 43269)	0.0331	0.15	4.61	6.98
3851	480.4654	F	0.2654	(0.63, 0.44)		DataPointRange(43270 - 43293)
3852	480.7193	S	0.0444	(0.63, 0.43)-(0.79, 0.41)	DataPointRange(43294 - 43297)	0.0444	0.15	3.41	6.04
3853	480.7745	F	0.1658	(0.79, 0.43)		DataPointRange(43298 - 43312)
3854	480.9298	S	0.0548	(0.79, 0.44)-(0.20, 0.26)	DataPointRange(43313 - 43317)	0.0548	0.60	11.00	24.19
3855	480.9960	F	0.0106	(0.14, 0.29)		DataPointRange(43318 - 43318)
3856	480.9960	S	0.0330	(0.14, 0.29)-(0.29, 0.46)	DataPointRange(43319 - 43321)	0.0330	0.20	6.21	11.72
3857	481.0396	F	0.3869	(0.29, 0.45)		DataPointRange(43322 - 43356)
3858	481.4154	S	0.0330	(0.29, 0.44)-(0.13, 0.58)	DataPointRange(43357 - 43359)	0.0330	0.19	5.70	8.86
3859	481.4597	F	0.1873	(0.12, 0.58)		DataPointRange(43360 - 43376)
3860	481.6361	S	0.0224	(0.11, 0.57)-(0.19, 0.55)	DataPointRange(43377 - 43378)	0.0224	0.08	3.49	5.42
3861	481.6693	F	0.2546	(0.19, 0.55)		DataPointRange(43379 - 43401)
3862	481.9124	S	0.0778	(0.19, 0.54)-(0.75, 0.45)	DataPointRange(43402 - 43408)	0.0778	0.56	7.26	11.86
3863	482.0008	F	0.1767	(0.76, 0.45)		DataPointRange(43409 - 43424)
3864	482.1665	S	0.0557	(0.77, 0.47)-(0.35, 0.50)	DataPointRange(43425 - 43429)	0.0557	0.42	7.59	10.53
3865	482.2328	F	0.1767	(0.35, 0.53)		DataPointRange(43430 - 43445)
3866	482.3986	S	0.0220	(0.35, 0.51)-(0.45, 0.54)	DataPointRange(43446 - 43447)	0.0220	0.10	4.69	8.27
3867	482.4316	F	0.2321	(0.46, 0.53)		DataPointRange(43448 - 43468)
3868	482.6525	S	0.0222	(0.46, 0.54)-(0.55, 0.54)	DataPointRange(43469 - 43470)	0.0222	0.09	4.00	6.61
3869	482.6857	F	0.2210	(0.54, 0.54)		DataPointRange(43471 - 43490)
3870	482.8953	S	0.0114	(0.54, 0.54)-(0.56, 0.52)	DataPointRange(43491 - 43491)	0.0114	0.02	2.14	2.14
3871	482.9177	F	0.1768	(0.59, 0.52)		DataPointRange(43492 - 43507)
3872	483.0835	S	0.0996	(0.59, 0.54)-(0.25, 0.41)	DataPointRange(43508 - 43516)	0.0996	0.36	3.58	11.56
3873	483.1938	F	0.3977	(0.27, 0.41)		DataPointRange(43517 - 43552)
3874	483.5809	S	0.0216	(0.27, 0.42)-(0.36, 0.42)	DataPointRange(43553 - 43554)	0.0216	0.09	4.24	6.71
3875	483.6135	F	0.3315	(0.34, 0.43)		DataPointRange(43555 - 43584)
3876	483.9336	S	0.0114	(0.34, 0.44)-(0.38, 0.45)	DataPointRange(43585 - 43585)	0.0114	0.04	3.53	3.53
3877	483.9557	F	0.2099	(0.42, 0.43)		DataPointRange(43586 - 43604)
3878	484.1656	End					DataPoint(43605)
3879	484.1582	S	0.0295	(0.43, 0.43)-(0.26, 0.28)	DataPointRange(43605 - 43607)	0.0295	0.20	6.82	10.41
3880	484.1988	F	0.1879	(0.27, 0.25)		DataPointRange(43608 - 43624)
3881	484.3756	S	0.0222	(0.27, 0.25)-(0.20, 0.21)	DataPointRange(43625 - 43626)	0.0222	0.08	3.45	5.79
3882	484.4088	F	0.4639	(0.19, 0.18)		DataPointRange(43627 - 43668)
3883	484.5414	aa1n6s.bmp				DataPoint(43639)
3884	484.5965	Start					DataPoint(43644)
3885	484.8616	S	0.0111	(0.20, 0.18)-(0.23, 0.17)	DataPointRange(43669 - 43669)	0.0111	0.03	2.50	2.50
3886	484.8836	F	0.2099	(0.27, 0.17)		DataPointRange(43670 - 43688)
3887	485.0825	S	0.0772	(0.28, 0.18)-(0.36, 0.10)	DataPointRange(43689 - 43695)	0.0772	0.10	1.32	22.87
3888	485.1711	F	0.0108	(0.33, 0.09)		DataPointRange(43696 - 43696)
3889	485.1711	S	0.0219	(0.33, 0.09)-(0.40, 0.17)	DataPointRange(43697 - 43698)	0.0219	0.09	4.06	6.31
3890	485.2044	F	0.2206	(0.40, 0.17)		DataPointRange(43699 - 43718)
3891	485.4139	S	0.0222	(0.39, 0.17)-(0.51, 0.18)	DataPointRange(43719 - 43720)	0.0222	0.12	5.25	6.86
3892	485.4471	F	0.4752	(0.56, 0.17)		DataPointRange(43721 - 43763)
3893	485.9113	S	0.0555	(0.57, 0.18)-(0.20, 0.23)	DataPointRange(43764 - 43768)	0.0555	0.37	6.69	11.24
3894	485.9773	F	0.2653	(0.18, 0.24)		DataPointRange(43769 - 43792)
3895	486.2315	S	0.0221	(0.19, 0.25)-(0.24, 0.25)	DataPointRange(43793 - 43794)	0.0221	0.05	2.31	4.92
3896	486.2647	F	0.2541	(0.27, 0.25)		DataPointRange(43795 - 43817)
3897	486.5078	S	0.0110	(0.27, 0.24)-(0.29, 0.26)	DataPointRange(43818 - 43818)	0.0110	0.03	2.73	2.73
3898	486.5298	F	0.2432	(0.32, 0.26)		DataPointRange(43819 - 43840)
3899	486.7622	S	0.0333	(0.32, 0.25)-(0.43, 0.24)	DataPointRange(43841 - 43843)	0.0333	0.11	3.30	5.67
3900	486.8059	F	0.2981	(0.46, 0.26)		DataPointRange(43844 - 43870)
3901	487.0929	S	0.0220	(0.46, 0.26)-(0.53, 0.25)	DataPointRange(43871 - 43872)	0.0220	0.07	3.26	6.55
3902	487.1259	F	0.1440	(0.54, 0.26)		DataPointRange(43873 - 43882)
3903	487.2588	S	0.0335	(0.53, 0.23)-(0.62, 0.27)	DataPointRange(43883 - 43885)	0.0335	0.09	2.76	3.70
3904	487.3029	F	0.0664	(0.63, 0.26)		DataPointRange(43886 - 43891)
3905	487.3582	S	0.0667	(0.63, 0.26)-(0.20, 0.32)	DataPointRange(43892 - 43897)	0.0667	0.43	6.49	11.89
3906	487.4357	F	0.3203	(0.20, 0.33)		DataPointRange(43898 - 43926)
3907	487.7450	S	0.0219	(0.20, 0.33)-(0.27, 0.34)	DataPointRange(43927 - 43928)	0.0219	0.07	3.22	4.69
3908	487.7779	F	0.2099	(0.26, 0.35)		DataPointRange(43929 - 43947)
3909	487.9769	S	0.0330	(0.27, 0.37)-(0.39, 0.36)	DataPointRange(43948 - 43950)	0.0330	0.13	3.84	6.93
3910	488.0212	F	0.2758	(0.42, 0.35)		DataPointRange(43951 - 43975)
3911	488.2862	S	0.0108	(0.42, 0.35)-(0.47, 0.33)	DataPointRange(43976 - 43976)	0.0108	0.05	4.91	4.91
3912	488.3080	F	0.7182	(0.50, 0.34)		DataPointRange(43977 - 44041)
3913	489.0152	S	0.0222	(0.50, 0.33)-(0.42, 0.29)	DataPointRange(44042 - 44043)	0.0222	0.08	3.63	7.54
3914	489.0484	F	0.1547	(0.42, 0.29)		DataPointRange(44044 - 44056)
3915	489.1925	S	0.0215	(0.41, 0.28)-(0.34, 0.27)	DataPointRange(44057 - 44058)	0.0215	0.08	3.51	4.35
3916	489.2251	F	0.1990	(0.33, 0.26)		DataPointRange(44059 - 44076)
3917	489.4126	S	0.0221	(0.33, 0.25)-(0.27, 0.20)	DataPointRange(44077 - 44078)	0.0221	0.07	3.24	5.48
3918	489.4457	F	0.3097	(0.30, 0.19)		DataPointRange(44079 - 44106)
3919	489.7442	S	0.0445	(0.30, 0.19)-(0.47, 0.29)	DataPointRange(44107 - 44110)	0.0445	0.19	4.24	7.38
3920	489.7996	F	0.3423	(0.47, 0.28)		DataPointRange(44111 - 44141)
3921	489.8547	End					DataPoint(44116)
3922	490.1308	S	0.0442	(0.47, 0.29)-(0.33, 0.25)	DataPointRange(44142 - 44145)	0.0442	0.14	3.07	8.24
3923	490.1860	F	0.0335	(0.35, 0.25)		DataPointRange(44146 - 44148)
3924	490.2081	aa1n15s.bmp				DataPoint(44148)
3925	490.2081	S	0.0331	(0.36, 0.25)-(0.27, 0.21)	DataPointRange(44149 - 44151)	0.0331	0.09	2.84	4.56
3926	490.2526	F	0.1430	(0.26, 0.22)		DataPointRange(44152 - 44162)
3927	490.3295	Start					DataPoint(44157)
3928	490.3847	S	0.0223	(0.26, 0.21)-(0.20, 0.19)	DataPointRange(44163 - 44164)	0.0223	0.06	2.80	5.52
3929	490.4178	F	0.1878	(0.18, 0.17)		DataPointRange(44165 - 44181)
3930	490.5947	S	0.0220	(0.18, 0.16)-(0.27, 0.16)	DataPointRange(44182 - 44183)	0.0220	0.09	4.19	4.74
3931	490.6277	F	0.1879	(0.29, 0.17)		DataPointRange(44184 - 44200)
3932	490.8046	S	0.0110	(0.29, 0.18)-(0.34, 0.16)	DataPointRange(44201 - 44201)	0.0110	0.04	4.03	4.03
3933	490.8269	F	0.2651	(0.37, 0.17)		DataPointRange(44202 - 44225)
3934	491.0809	S	0.0329	(0.36, 0.17)-(0.48, 0.18)	DataPointRange(44226 - 44228)	0.0329	0.12	3.62	5.83
3935	491.1250	F	0.2320	(0.52, 0.17)		DataPointRange(44229 - 44249)
3936	491.3459	S	0.0111	(0.52, 0.17)-(0.56, 0.17)	DataPointRange(44250 - 44250)	0.0111	0.04	3.24	3.24
3937	491.3681	F	0.1324	(0.59, 0.17)		DataPointRange(44251 - 44262)
3938	491.4893	S	0.0112	(0.59, 0.17)-(0.58, 0.17)	DataPointRange(44263 - 44263)	0.0112	0.01	0.60	0.60
3939	491.5114	F	0.0110	(0.55, 0.23)		DataPointRange(44264 - 44264)
3940	491.5114	S	0.0110	(0.55, 0.23)-(0.59, 0.17)	DataPointRange(44265 - 44265)	0.0110	0.06	5.45	5.45
3941	491.5334	F	0.0553	(0.59, 0.17)		DataPointRange(44266 - 44270)
3942	491.5777	S	0.0222	(0.59, 0.17)-(0.64, 0.15)	DataPointRange(44271 - 44272)	0.0222	0.06	2.55	3.88
3943	491.6109	F	0.2984	(0.64, 0.16)		DataPointRange(44273 - 44299)
3944	491.8987	S	0.0331	(0.64, 0.17)-(0.74, 0.17)	DataPointRange(44300 - 44302)	0.0331	0.10	3.04	6.64
3945	491.9425	F	0.5197	(0.77, 0.17)		DataPointRange(44303 - 44349)
3946	492.4506	S	0.0116	(0.76, 0.16)-(0.78, 0.18)	DataPointRange(44350 - 44350)	0.0116	0.02	1.98	1.98
3947	492.4727	F	0.0886	(0.81, 0.16)		DataPointRange(44351 - 44358)
3948	492.5498	S	0.0774	(0.82, 0.15)-(0.21, 0.23)	DataPointRange(44359 - 44365)	0.0774	0.62	8.01	12.69
3949	492.6380	F	0.2548	(0.17, 0.27)		DataPointRange(44366 - 44388)
3950	492.8814	S	0.0332	(0.17, 0.28)-(0.32, 0.26)	DataPointRange(44389 - 44391)	0.0332	0.14	4.36	7.03
3951	492.9255	F	0.3313	(0.36, 0.25)		DataPointRange(44392 - 44421)
3952	493.2458	S	0.0222	(0.37, 0.25)-(0.45, 0.25)	DataPointRange(44422 - 44423)	0.0222	0.09	3.94	5.11
3953	493.2790	F	0.2207	(0.48, 0.25)		DataPointRange(44424 - 44443)
3954	493.4892	S	0.0331	(0.48, 0.23)-(0.63, 0.28)	DataPointRange(44444 - 44446)	0.0331	0.15	4.62	6.55
3955	493.5329	F	0.1656	(0.67, 0.25)		DataPointRange(44447 - 44461)
3956	493.6877	S	0.0108	(0.66, 0.23)-(0.67, 0.26)	DataPointRange(44462 - 44462)	0.0108	0.02	2.18	2.18
3957	493.7096	F	0.0110	(0.60, 0.32)		DataPointRange(44463 - 44463)
3958	493.7096	S	0.0110	(0.60, 0.32)-(0.67, 0.24)	DataPointRange(44464 - 44464)	0.0110	0.09	8.28	8.28
3959	493.7316	F	0.0774	(0.67, 0.25)		DataPointRange(44465 - 44471)
3960	493.7979	S	0.0331	(0.66, 0.25)-(0.77, 0.24)	DataPointRange(44472 - 44474)	0.0331	0.11	3.36	6.01
3961	493.8422	F	0.1550	(0.77, 0.25)		DataPointRange(44475 - 44488)
3962	493.9860	S	0.0221	(0.77, 0.26)-(0.81, 0.25)	DataPointRange(44489 - 44490)	0.0221	0.05	2.05	3.26
3963	494.0191	F	0.1991	(0.84, 0.25)		DataPointRange(44491 - 44508)
3964	494.2069	S	0.0662	(0.83, 0.24)-(0.28, 0.32)	DataPointRange(44509 - 44514)	0.0662	0.56	8.39	12.09
3965	494.2846	F	0.1434	(0.24, 0.35)		DataPointRange(44515 - 44527)
3966	494.4172	S	0.0217	(0.24, 0.36)-(0.16, 0.35)	DataPointRange(44528 - 44529)	0.0217	0.08	3.85	6.19
3967	494.4502	F	0.3090	(0.15, 0.36)		DataPointRange(44530 - 44557)
3968	494.7485	S	0.0436	(0.14, 0.37)-(0.33, 0.34)	DataPointRange(44558 - 44561)	0.0436	0.19	4.44	7.31
3969	494.8031	F	0.2211	(0.39, 0.34)		DataPointRange(44562 - 44581)
3970	495.0132	S	0.0221	(0.40, 0.34)-(0.49, 0.36)	DataPointRange(44582 - 44583)	0.0221	0.09	3.91	5.67
3971	495.0465	F	0.2319	(0.51, 0.35)		DataPointRange(44584 - 44604)
3972	495.2674	S	0.0220	(0.52, 0.35)-(0.57, 0.34)	DataPointRange(44605 - 44606)	0.0220	0.04	2.01	4.64
3973	495.3006	F	0.1436	(0.58, 0.34)		DataPointRange(44607 - 44619)
3974	495.4331	S	0.0331	(0.58, 0.34)-(0.42, 0.36)	DataPointRange(44620 - 44622)	0.0331	0.16	4.72	8.73
3975	495.4773	F	0.1436	(0.40, 0.35)		DataPointRange(44623 - 44635)
3976	495.6099	S	0.0550	(0.39, 0.35)-(0.79, 0.37)	DataPointRange(44636 - 44640)	0.0550	0.40	7.19	10.24
3977	495.6764	F	0.5299	(0.79, 0.36)		DataPointRange(44641 - 44688)
3978	496.1952	S	0.0111	(0.79, 0.35)-(0.82, 0.34)	DataPointRange(44689 - 44689)	0.0111	0.03	2.76	2.76
3979	496.2174	F	0.1767	(0.83, 0.34)		DataPointRange(44690 - 44705)
3980	496.3830	S	0.1547	(0.84, 0.36)-(0.75, 0.27)	DataPointRange(44706 - 44719)	0.1547	0.11	0.72	17.83
3981	496.5488	F	0.1767	(0.75, 0.30)		DataPointRange(44720 - 44735)
3982	496.7144	S	0.0226	(0.76, 0.31)-(0.84, 0.35)	DataPointRange(44736 - 44737)	0.0226	0.08	3.54	5.95
3983	496.7476	F	0.3312	(0.86, 0.34)		DataPointRange(44738 - 44767)
3984	497.0676	S	0.0443	(0.84, 0.34)-(0.59, 0.28)	DataPointRange(44768 - 44771)	0.0443	0.26	5.79	11.99
3985	497.1229	F	0.3979	(0.59, 0.28)		DataPointRange(44772 - 44804)
3986	497.5097	S	0.0332	(0.59, 0.26)-(0.46, 0.25)	DataPointRange(44805 - 44807)	0.0332	0.13	3.99	6.57
3987	497.5539	F	0.2098	(0.45, 0.23)		DataPointRange(44808 - 44826)
3988	497.7525	S	0.0333	(0.44, 0.25)-(0.26, 0.19)	DataPointRange(44827 - 44829)	0.0333	0.18	5.40	8.81
3989	497.7973	F	0.2427	(0.25, 0.20)		DataPointRange(44830 - 44851)
3990	498.0294	S	0.0106	(0.24, 0.20)-(0.29, 0.17)	DataPointRange(44852 - 44852)	0.0106	0.05	4.97	4.97
3991	498.0509	F	0.6185	(0.30, 0.17)		DataPointRange(44853 - 44908)
3992	498.1614	End					DataPoint(44863)
3993	498.5149	aa1n13.bmp				DataPoint(44895)
3994	498.6140	Start					DataPoint(44904)
3995	498.6583	S	0.0220	(0.30, 0.17)-(0.21, 0.17)	DataPointRange(44909 - 44910)	0.0220	0.09	4.07	4.90
3996	498.6915	F	0.1769	(0.19, 0.17)		DataPointRange(44911 - 44926)
3997	498.8573	S	0.0332	(0.18, 0.17)-(0.33, 0.17)	DataPointRange(44927 - 44929)	0.0332	0.15	4.42	6.82
3998	498.9016	F	0.2208	(0.36, 0.17)		DataPointRange(44930 - 44949)
3999	499.1115	S	0.0109	(0.35, 0.17)-(0.40, 0.19)	DataPointRange(44950 - 44950)	0.0109	0.05	4.47	4.47
4000	499.1334	F	0.2319	(0.45, 0.18)		DataPointRange(44951 - 44971)
4001	499.3543	S	0.0221	(0.44, 0.18)-(0.50, 0.17)	DataPointRange(44972 - 44973)	0.0221	0.06	2.73	4.86
4002	499.3876	F	0.2431	(0.52, 0.18)		DataPointRange(44974 - 44995)
4003	499.6195	S	0.0222	(0.52, 0.18)-(0.64, 0.18)	DataPointRange(44996 - 44997)	0.0222	0.12	5.43	6.58
4004	499.6528	F	0.2208	(0.68, 0.18)		DataPointRange(44998 - 45017)
4005	499.8626	S	0.0225	(0.68, 0.18)-(0.73, 0.17)	DataPointRange(45018 - 45019)	0.0225	0.05	2.40	3.59
4006	499.8957	F	0.2760	(0.77, 0.18)		DataPointRange(45020 - 45044)
4007	500.1606	S	0.0665	(0.76, 0.19)-(0.21, 0.26)	DataPointRange(45045 - 45050)	0.0665	0.56	8.41	11.78
4008	500.2380	F	0.1438	(0.23, 0.26)		DataPointRange(45051 - 45063)
4009	500.3706	S	0.0221	(0.22, 0.25)-(0.14, 0.23)	DataPointRange(45064 - 45065)	0.0221	0.09	3.94	6.08
4010	500.4037	F	0.2211	(0.15, 0.26)		DataPointRange(45066 - 45085)
4011	500.6137	S	0.0331	(0.15, 0.28)-(0.28, 0.26)	DataPointRange(45086 - 45088)	0.0331	0.13	4.08	6.90
4012	500.6580	F	0.2980	(0.30, 0.26)		DataPointRange(45089 - 45115)
4013	500.9453	S	0.0107	(0.30, 0.26)-(0.28, 0.26)	DataPointRange(45116 - 45116)	0.0107	0.02	1.69	1.69
4014	500.9671	F	0.1877	(0.26, 0.25)		DataPointRange(45117 - 45133)
4015	501.1443	S	0.0327	(0.26, 0.27)-(0.37, 0.25)	DataPointRange(45134 - 45136)	0.0327	0.11	3.38	18.76
4016	501.1882	F	0.2543	(0.41, 0.25)		DataPointRange(45137 - 45159)
4017	501.4310	S	0.0220	(0.41, 0.25)-(0.51, 0.25)	DataPointRange(45160 - 45161)	0.0220	0.10	4.70	6.01
4018	501.4643	F	0.4750	(0.55, 0.26)		DataPointRange(45162 - 45204)
4019	501.9283	S	0.0330	(0.59, 0.26)-(0.71, 0.28)	DataPointRange(45205 - 45207)	0.0330	0.13	3.84	5.64
4020	501.9725	F	0.2983	(0.75, 0.27)		DataPointRange(45208 - 45234)
4021	502.2596	S	0.0112	(0.75, 0.27)-(0.78, 0.26)	DataPointRange(45235 - 45235)	0.0112	0.04	3.18	3.18
4022	502.2817	F	0.1218	(0.80, 0.26)		DataPointRange(45236 - 45246)
4023	502.3921	S	0.0991	(0.79, 0.25)-(0.09, 0.22)	DataPointRange(45247 - 45255)	0.0991	0.69	7.01	23.05
4024	502.5026	F	0.0108	(0.12, 0.24)		DataPointRange(45256 - 45256)
4025	502.5026	S	0.0218	(0.12, 0.24)-(0.17, 0.37)	DataPointRange(45257 - 45258)	0.0218	0.11	5.12	10.13
4026	502.5357	F	0.2540	(0.17, 0.37)		DataPointRange(45259 - 45281)
4027	502.7787	S	0.0224	(0.17, 0.38)-(0.27, 0.36)	DataPointRange(45282 - 45283)	0.0224	0.10	4.56	6.90
4028	502.8120	F	0.1988	(0.28, 0.35)		DataPointRange(45284 - 45301)
4029	502.9995	S	0.0444	(0.28, 0.34)-(0.48, 0.37)	DataPointRange(45302 - 45305)	0.0444	0.21	4.67	7.59
4030	503.0549	F	0.1989	(0.52, 0.35)		DataPointRange(45306 - 45323)
4031	503.2427	S	0.0111	(0.51, 0.34)-(0.47, 0.36)	DataPointRange(45324 - 45324)	0.0111	0.04	3.84	3.84
4032	503.2652	F	0.3418	(0.47, 0.36)		DataPointRange(45325 - 45355)
4033	503.5960	S	0.0330	(0.47, 0.34)-(0.57, 0.34)	DataPointRange(45356 - 45358)	0.0330	0.10	3.01	5.19
4034	503.6402	F	0.4421	(0.60, 0.34)		DataPointRange(45359 - 45398)
4035	504.0712	S	0.0554	(0.61, 0.35)-(0.49, 0.27)	DataPointRange(45399 - 45403)	0.0554	0.14	2.52	11.24
4036	504.1375	F	0.0218	(0.52, 0.27)		DataPointRange(45404 - 45405)
4037	504.1485	S	0.0221	(0.53, 0.26)-(0.54, 0.33)	DataPointRange(45406 - 45407)	0.0221	0.05	2.40	5.55
4038	504.1816	F	0.4637	(0.53, 0.34)		DataPointRange(45408 - 45449)
4039	504.6342	S	0.0332	(0.53, 0.34)-(0.37, 0.27)	DataPointRange(45450 - 45452)	0.0332	0.17	5.13	8.00
4040	504.6785	F	0.8176	(0.35, 0.25)		DataPointRange(45453 - 45526)
4041	505.4854	S	0.0438	(0.34, 0.25)-(0.16, 0.19)	DataPointRange(45527 - 45530)	0.0438	0.19	4.33	6.90
4042	505.5402	F	0.2208	(0.15, 0.20)		DataPointRange(45531 - 45550)
4043	505.7502	S	0.0217	(0.15, 0.21)-(0.20, 0.16)	DataPointRange(45551 - 45552)	0.0217	0.07	3.06	4.88
4044	505.7831	F	0.6076	(0.21, 0.16)		DataPointRange(45553 - 45607)
4045	505.8051	End					DataPoint(45555)
4046	506.1811	aa1n17s.bmp				DataPoint(45589)
4047	506.2252	Start					DataPoint(45593)
4048	506.3797	S	0.0221	(0.21, 0.17)-(0.29, 0.18)	DataPointRange(45608 - 45609)	0.0221	0.08	3.66	4.30
4049	506.4130	F	0.2323	(0.30, 0.17)		DataPointRange(45610 - 45630)
4050	506.6339	S	0.0332	(0.30, 0.18)-(0.47, 0.17)	DataPointRange(45631 - 45633)	0.0332	0.17	5.13	5.88
4051	506.6786	F	0.2533	(0.48, 0.18)		DataPointRange(45634 - 45656)
4052	506.9209	S	0.0332	(0.48, 0.20)-(0.56, 0.19)	DataPointRange(45657 - 45659)	0.0332	0.08	2.51	3.73
4053	506.9651	F	0.2176	(0.57, 0.17)		DataPointRange(45660 - 45677)
4054	507.1530	S	0.0442	(0.56, 0.17)-(0.69, 0.17)	DataPointRange(45678 - 45680)	0.0442	0.12	2.80	12.45
4055	507.2084	F	0.2209	(0.71, 0.17)		DataPointRange(45681 - 45700)
4056	507.4180	S	0.0336	(0.71, 0.17)-(0.81, 0.18)	DataPointRange(45701 - 45703)	0.0336	0.09	2.79	4.32
4057	507.4624	F	0.2651	(0.81, 0.17)		DataPointRange(45704 - 45727)
4058	507.7163	S	0.0667	(0.83, 0.20)-(-0.90, -0.15)	DataPointRange(45728 - 45733)	0.0667	1.74	26.16	218.22
4059	507.7938	F	0.0223	(-0.93, -0.18)		DataPointRange(45734 - 45735)
4060	507.8048	S	0.0219	(-0.92, -0.14)-(1.87, -0.21)	DataPointRange(45736 - 45737)	0.0219	2.79	127.42	268.76
4061	507.8379	F	0.0220	(1.87, -0.21)		DataPointRange(45738 - 45739)
4062	507.8493	S	0.0991	(1.87, -0.21)-(0.84, 0.19)	DataPointRange(45740 - 45748)	0.0991	1.07	10.81	244.61
4063	507.9592	F	0.0331	(0.83, 0.15)		DataPointRange(45749 - 45751)
4064	507.9815	S	0.0108	(0.83, 0.15)-(0.83, 0.19)	DataPointRange(45752 - 45752)	0.0108	0.03	2.81	2.81
4065	508.0034	F	0.1328	(0.84, 0.21)		DataPointRange(45753 - 45764)
4066	508.1249	S	0.0775	(0.84, 0.20)-(0.19, 0.30)	DataPointRange(45765 - 45771)	0.0775	0.65	8.39	12.81
4067	508.2137	F	0.4082	(0.17, 0.29)		DataPointRange(45772 - 45808)
4068	508.6111	S	0.0334	(0.15, 0.30)-(0.28, 0.28)	DataPointRange(45809 - 45811)	0.0334	0.13	3.94	5.75
4069	508.6553	F	0.2873	(0.30, 0.26)		DataPointRange(45812 - 45837)
4070	508.9315	S	0.0221	(0.32, 0.25)-(0.40, 0.25)	DataPointRange(45838 - 45839)	0.0221	0.08	3.68	5.36
4071	508.9647	F	0.1765	(0.41, 0.27)		DataPointRange(45840 - 45855)
4072	509.1301	S	0.0220	(0.41, 0.27)-(0.50, 0.27)	DataPointRange(45856 - 45857)	0.0220	0.08	3.81	5.42
4073	509.1633	F	0.1109	(0.52, 0.26)		DataPointRange(45858 - 45867)
4074	509.2629	S	0.0993	(0.53, 0.27)-(0.66, 0.27)	DataPointRange(45868 - 45876)	0.0993	0.13	1.29	7.45
4075	509.3735	F	0.2208	(0.66, 0.27)		DataPointRange(45877 - 45896)
4076	509.5832	S	0.0221	(0.67, 0.27)-(0.73, 0.26)	DataPointRange(45897 - 45898)	0.0221	0.07	2.98	5.75
4077	509.6164	F	0.1988	(0.75, 0.27)		DataPointRange(45899 - 45916)
4078	509.8042	S	0.0441	(0.76, 0.28)-(0.28, 0.36)	DataPointRange(45917 - 45920)	0.0441	0.48	10.89	21.93
4079	509.8598	F	0.0109	(0.32, 0.33)		DataPointRange(45921 - 45921)
4080	509.8598	S	0.0109	(0.32, 0.33)-(0.26, 0.36)	DataPointRange(45922 - 45922)	0.0109	0.06	5.86	5.86
4081	509.8815	F	0.1658	(0.25, 0.34)		DataPointRange(45923 - 45937)
4082	510.0364	S	0.0218	(0.25, 0.34)-(0.20, 0.34)	DataPointRange(45938 - 45939)	0.0218	0.06	2.66	5.26
4083	510.0698	F	0.3529	(0.18, 0.37)		DataPointRange(45940 - 45971)
4084	510.4116	S	0.0226	(0.19, 0.37)-(0.27, 0.36)	DataPointRange(45972 - 45973)	0.0226	0.08	3.42	5.59
4085	510.4448	F	0.2763	(0.29, 0.36)		DataPointRange(45974 - 45998)
4086	510.7099	S	0.0662	(0.29, 0.34)-(0.71, 0.28)	DataPointRange(45999 - 46004)	0.0662	0.42	6.38	9.62
4087	510.7875	F	0.2646	(0.72, 0.27)		DataPointRange(46005 - 46028)
4088	511.0410	S	0.2320	(0.72, 0.27)-(0.24, 0.36)	DataPointRange(46029 - 46049)	0.2320	0.48	2.06	156.41
4089	511.2840	F	0.0445	(0.26, 0.33)		DataPointRange(46050 - 46053)
4090	511.3172	S	0.0113	(0.25, 0.31)-(0.27, 0.37)	DataPointRange(46054 - 46054)	0.0113	0.05	4.38	4.38
4091	511.3393	F	0.2212	(0.27, 0.37)		DataPointRange(46055 - 46074)
4092	511.5494	S	0.0221	(0.27, 0.37)-(0.35, 0.31)	DataPointRange(46075 - 46076)	0.0221	0.09	4.16	4.87
4093	511.5825	F	0.1989	(0.37, 0.30)		DataPointRange(46077 - 46094)
4094	511.7704	S	0.0442	(0.38, 0.29)-(0.27, 0.17)	DataPointRange(46095 - 46098)	0.0442	0.14	3.20	5.83
4095	511.8260	F	0.4745	(0.28, 0.20)		DataPointRange(46099 - 46141)
4096	511.9361	End					DataPoint(46109)
4097	512.2898	S	0.0437	(0.28, 0.19)-(0.49, 0.52)	DataPointRange(46142 - 46145)	0.0437	0.32	7.41	10.29
4098	512.3445	F	0.6186	(0.49, 0.51)		DataPointRange(46146 - 46201)
